# Neuroimaging Feature Terminology: A Controlled Terminology for the Annotation of Brain Imaging Features

**DOI:** 10.3233/JAD-161148

**Published:** 2017-08-14

**Authors:** Anandhi Iyappan, Erfan Younesi, Alberto Redolfi, Henri Vrooman, Shashank Khanna, Giovanni B. Frisoni, Martin Hofmann-Apitius, for the Alzheimer’s Disease Neuroimaging Initiative

**Affiliations:** a Department of Bioinformatics, Fraunhofer Institute for Algorithms and Scientific Computing (SCAI), Schloss Birlinghoven, Sankt Augustin, Germany; b Rheinische Friedrich-Wilhelms-Universität Bonn, Bonn-Aachen International Center for Information Technology, Bonn, Germany; c Laboratory of Epidemiology and Neuroimaging, IRCCS San Giovanni di Dio Fatebenefratelli, Brescia, Italy; d Departments of Radiology and Medical Informatics, Biomedical Imaging Group Rotterdam, Erasmus MC University Medical Center, The Netherlands; e Memory Clinic and Laboratoire de Neuroimagerie du Vieillissement (LANVIE), University Hospitals and University of Geneva, Geneva, Switzerland

**Keywords:** Alzheimer’s disease, annotation, brain, neuroimaging, terminology

## Abstract

Ontologies and terminologies are used for interoperability of knowledge and data in a standard manner among interdisciplinary research groups. Existing imaging ontologies capture general aspects of the imaging domain as a whole such as methodological concepts or calibrations of imaging instruments. However, none of the existing ontologies covers the diagnostic features measured by imaging technologies in the context of neurodegenerative diseases. Therefore, the Neuro-Imaging Feature Terminology (NIFT) was developed to organize the knowledge domain of measured brain features in association with neurodegenerative diseases by imaging technologies. The purpose is to identify quantitative imaging biomarkers that can be extracted from multi-modal brain imaging data. This terminology attempts to cover measured features and parameters in brain scans relevant to disease progression. In this paper, we demonstrate the systematic retrieval of measured indices from literature and how the extracted knowledge can be further used for disease modeling that integrates neuroimaging features with molecular processes.

## INTRODUCTION

Brain imaging technologies have revolutionized the way that neurodegenerative diseases, such as Alzheimer’s disease (AD), are diagnosed and tracked. Since the human brain is largely inaccessible for direct sampling, neuroimaging provides an alternative for measuring *in vivo* structural and functional features that can be used as biomarkers of disease onset and progression. The quantitative nature of imaging biomarkers, their potential to assess disease-modifying effects, and the ability to monitor the safety of candidate drugs all make neuroimaging readouts an extensively used, measurable endpoint in clinical trials for neurodegenerative diseases [1]. Parameters and features that are clinically measured using neuroimaging biomarkers reflect biological or pathological changes underlying disease in the brain of patients; for example, positron emission tomography (PET) imaging measures the load of amyloid-β (Aβ) neuritic plaques through the uptake and binding of a particular radioligand in the living brain [2]. But such measurements at the clinical level are often disconnected from their underlying mechanistic causes, on one hand, and from their corresponding patient clinical tests, on the other hand.

The importance of neuroimaging in the new era of systems neurology is highlighted by its pivotal role in linking clinical readouts to underlying mechanistic changes [3]. Thus, there is a need for integrative approaches that enable multiscale modeling of both biological and clinical data with the aim of bridging the translational gap [4]. The first step toward this goal is, however, the collection and standardization of disparate and scattered data and knowledge across many resources available for research. Among several efforts in this direction, ApiNATOMY correlates brain imaging diagnostics to affected anatomical regions of the brain through the Foundational Model of Anatomy ontology (FMA) [5]; the OntoNeuroLOG ontology covers the domain of imaging datasets and their processing methods [6]; RadLex provides a lexicon of terms relevant to diagnostic and interventional radiology [7]; and the quantitative imaging biomarker ontology (QIBO) represents a series of heterogeneous concepts across several fields including imaging physics, contrast agents, biology, and quantitation techniques [8]. Neuroimaging Data Model and Taskforce (NIDM) facilitates the exchange of large publication corpus and other relevant metadata such as provenance information related to the neuroimaging research for establishing the reproducibility of research experiments as well as overcoming the challenge of data sharing (http://nidm.nidash.org/specs/nidm-overview.html).

In parallel to the generation of imaging datasets, an increasing amount of imaging information is published within literature articles which often report on measured features in patients with AD [9, 10]. Such studies typically try to correlate neuroimaging readouts with defined disease stages or subtypes. As an example, Whitwell and co-workers utilized magnetic resonance imaging (MRI) in patients with atypical variants of AD and were able to categorize these patient groups, based on measuring patterns of atrophy in medial temporal and cortical grey matter, into hippocampal sparing AD, limbic predominant AD, and typical AD subtypes [11]. This example clearly shows the importance of harvesting neuroimaging feature information from literature not only for monitoring critical imaging findings but also for stratification of patients based on their diagnostic status.

To this end, UMLS metathesaurus vocabularies were used to annotate and index radiology journal figure captions from more than 9000 articles for image information retrieval [12]. Similarly, RadLex was applied to the biomedical imaging literature and annotated more than 385,000 figures with RadLex terms [13]. However, when the National Cancer Institute Thesaurus (NCIT), Radiology Lexicon (RadLex; http://www.radlex.org/), Systemized Nomenclature of Medicine (SNOMED-CT), and International Classification of Diseases (ICD-9-CM) were evaluated for retrieval of radiology reports containing critical imaging findings, it was found that no single terminology is optimal for retrieving radiology reports with critical findings [14].

Biomedical terminologies and ontologies have proven their role in namespace harmonization and mediation of semantic interoperability in numerous examples [15]. One of the main application domains of shared semantics (ontologies and terminologies) lies in metadata annotation as well as data integration and knowledge retrieval [16]. The neuroimaging community has not yet come up with a consensus for commonly used and shared metadata. However, over the past decades, many initiatives have made their primary data publicly available [17]. Out of those, the Alzheimer Disease Neuroimaging Initiative (ADNI) (http://adni.loni.usc.edu/) and Parkinson’s Progression Markers Initiative (PPMI) (http://www.ppmi-info.org/) have gained increasing momentum for creating an impact on data sharing across the scientific community. Despite the ongoing efforts, the significant lack of structural and semantic interoperability impedes the momentum of data sharing [18] and lack of an established framework hampers the merging of imaging data from other resources [8]. The heterogeneous data such as cortical thickness or neuropsychological assessments of individual patients that are stored in ADNI/PPMI datasets do not follow a standard nomenclature, which makes them difficult to interpret or use for validation.

Motivated by the obvious need for a terminology that enables a systematic representation and retrieval of features derived from neuroimaging techniques, we aimed at developing a Neuro-Imaging Feature Terminology (NIFT) to capture and organize the knowledge domain of structural and functional brain features as measured and represented by neuroimaging technologies in the domain of neurodegeneration. In this study, we demonstrate the value of NIFT for the identification and extraction of neuroimaging features in both Medline abstracts and full-text publications in the context of neurodegenerative disease pathology. We also demonstrate the applicability of NIFT for the annotation of imaging readouts in MRI and CT scans. Furthermore, we go beyond retrieval and annotation of imaging concepts by providing an example of how extracted neuroimaging features can be utilized for mechanistic modeling of disease pathology.

## MATERIALS AND METHODS

The NIFT terminology is built based on a hierarchical knowledge representation system by organizing higher level concepts as root nodes followed by specific sub-classes organized under them; however, it is not an ontology as it uses simple hierarchical relationships but is capable of being leveraged to an ontology in the future. However, to be leveraged to an ontology, NIFT should undergo major changes in the current hierarchical structure based on ontology formalism definitions. NIFT in its current form provides a first substrate for the scientific community to elaborate its conceptual complexity and structure. The Protégé OWL editor was used to build this hierarchical terminology (http://protege.stanford.edu/). This terminology was constructed using the OWL language for two reasons: firstly, the hierarchical edition and annotation of concepts in OWL language facilitates creation of such a granular terminology: and secondly, the OWL format of NIFT ensures the interoperability of the terminology file. The concepts that are included under this terminology were examined by experts from the clinical research domain.

### Generation of NIFT

The NIFT terminology concepts were gathered by collecting and reading relevant publications, e-books, websites, and medical blogs related to imaging in neurodegeneration. Following the initial literature search, we also adapted some concepts from already published, highly relevant ontologies such as QIBO [19] and Radlex. Ontologies such as QIBO and Radlex had well-structured concepts such as Imaging Techniques and Imaging Agents, which were contextually relevant for the development of NIFT. Essential entities used in the ADNI (http://adni.loni.usc.edu/) were also included in our terminology system.

Consequently, we enriched the NIFT with measured biomarkers obtained from the Biomedical Imaging Group Rotterdam (BIGR) pipeline, UMC Rotterdam [20] and neuGRID platform (http://www.neugrid4you.eu). The BIGR pipeline consists of six image processing pipelines such as FreeSurfer (http://freesurfer.net/), BIGR Tissue Segmentation [21], BIGR hippocampus segmentation [22], BIGR SAMSco [23], BIGR diffusion imaging pipeline [24], and Human Connectome Mapper [25]. The neuGRID platform consists of three image processing pipelines including: FreeSurfer, Adaboost, and SPMgrid. This platform was used to extract measured imaging indices to be added to the terminology.

For the sake of covering brain-specific anatomical structures in NIFT, we made use of the Brain Region & Cell Type Terminology (BRCT) which was initially developed to capture a wide range of key concepts representing human brain neuroanatomical structures and integrate their corresponding cell types (http://bioportal.bioontology.org/ontologies/BRCT). Alzheimer Disease Ontology [26] was also re-used to enrich NIFT. Pathway concepts were derived from the pathway Terminology System, that was developed with the intention to support the extraction of pathway information specific to the neurodegenerative disease domain [27].

Upon completion, this terminology system was reviewed by a clinical imaging expert team (Professor Frisoni’s team at the University Hospitals Geneva) which further improved the quality and relevance of the classification.

### Natural language processing (NLP)-based assessment of NIFT performance

In order to assess the relevancy of the NIFT terminology, we compared the performance of our terminology with the two already well-established imaging ontologies, QIBO and Biomedical Image Ontology (BIM) [28]. This comparison was performed at the level of terminologies found in those ontologies as they claim to capture the knowledge of image annotations and imaging biomarkers, respectively. To perform this, we used an NLP–based approach and ran the PDF tagger over the previously selected full-text publications (PMC1, PMC2, PMC3, and PMC4) using all the three ontology/terminology systems and validated the retrieval of the maximum annotation of terms specific to neuroimaging domain. The validation of the terminology using PDF tagger was performed using the formula:

$$\mathrm{mjk}=\;\sum_{i=1}^{\mathrm{nk}}ai$$

where a, frequency of single term; j, document number; mj, overall frequency in document j; nk, number of items in dictionary k; k, dictionary number.

This index sums up the recall of relevant terms captured using the relevant terminology over all the terms found in the document. This sum gives an overall count of different concepts and terms captured from the given document.

This analysis was done to demonstrate the usability of NIFT in extracting relevant context from publications of interest.

### Correlating clinical diagnosis with imaging features for staging AD

For bridging the clinical indices with imaging readouts, we systematically harvested relevant publications using the query “(((([NeuroimagingFeature]) AND [MeSH Disease “Alzheimer Disease”]) AND [Alzheimer Ontology: “Cognitive tests”]) AND [Organism: “Homo sapiens”]) AND [BRCT]” in SCAIView.

### Retrieval and mining figure captions and full-text from PubMed

Following the curation and further refinement of the terminology, NIFT was integrated into our in-house literature mining environment SCAIView [29]. SCAIView enables the users to efficiently retrieve context specific articles from the literature using standardized terminologies and ontologies. NIFT in its SCAIView integrated form can be freely accessed using this link (http://academia.scaiview.com/academia//).

We performed an overall coverage analysis of NIFT by running it over figure captions and full-text articles using SCAIView. For this, we converted the OWL file into a dictionary (.syn) file using a java program. The resulting dictionary was incorporated in ProMiner, which is a rule-based entity recognition system [30]. The hierarchical structure of the OWL file was converted into an XML tree so that NIFT can be navigated within the SCAIView environment and faceted search becomes feasible. The ProMiner program was subsequently run over the five figure captions which were enriched with imaging indices from PubMed articles and four full-text publications from PubMed Central (PMC), which generated an abstract with markup of the terms specific for NIFT.

We also performed an analysis of full-text publications using a special PDF tagger (http://publica.fraunhofer.de/eprints/urn_nbn_de_0011-n-936860.pdf). In order to perform this task, we chose four full-text publications, which were relevant to the neurodegenerative context as well as reported imaging findings namely PMC1 [31], PMC2 [32], PMC3 [33], and PMC4 [34]. The PDF tagger was run over these publications for validation of the coverage of NIFT and results were stored in a dedicated directory. The PDF tagger first makes use of the documents in the directory as an input to create a term list from all the PDF files. Following the complete annotation of the PDF files, an output file was automatically generated with the original PDF file containing additional annotations highlighted through markup of terms.

### Annotation of image scans using NIFT

In order to annotate brain scans with NIFT, we chose three groups of patients with different diagnostic features, namely: ADNI_016_S_4952 Control (CN), ADNI_002_S_4171 Mild Cognitive Impairment (MCI), and ADNI_003_S_4136 AD from the ADNI dataset (http://adni.loni.usc.edu/). ADNI is a large-scale, multicenter study which has been structured to develop molecular, clinical, and biochemical biomarkers from longitudinal patient data for early detection of AD. We processed PET (F18-AV-45 and FDG [18]) and T13D MPRAGE scans using the neuGRID platform with different pipelines, such as: SPMgrid to detect hypo-metabolism as well as amyloid burden; Freesurfer to highlight cortical thickness measurements and subcortical morphological differences; and Adaboost to quantify the hippocampal differences among the three diagnostic groups. The morphological changes observed from the patient scans were further annotated manually using the NIFT terminology.

### Mechanistic modeling of image-derived indices in the context of AD

Yet another important aspect of this paper is to identify the role of molecular mechanisms, which bring about clear diagnostic outcomes captured by imaging techniques. For this purpose, we generated the following query in SCAIView: “(([Neuroimaging Feature]) AND [MeSH Disease: “Alzheimer Disease”]) AND [Organism: “Homo sapiens”]” and filtered for Human Genes/Proteins. Next, we developed a “global map” of brain-region image-derived features along with molecular readouts, such as genes linked to a neuroimaging feature. We studied mechanisms of hippocampal atrophy in detail at molecular and cellular level. This knowledge was transformed into a cause-and-effect model using Biological Expression Language (BEL) platform (http://openbel.org/). BEL is a platform for representing causal and correlative relationships from biological context in a computer readable form. Then, we performed a high-resolution modelling of the mechanism underlying hippocampal atrophy. The outcome of this analysis will be further discussed in the Results section.

## RESULTS

Often, literature resources misclassify an imaging technique as a biomarker while many others denote the derived indices as a biomarker. Owing to this, NIFT was constructed to represent, integrate, and harmonize heterogeneous knowledge across the domain of imaging biomarkers in the context of neurodegeneration.

### Structure and content

NIFT comprises of 7 major classes namely Algorithms, Brain Region, Clinical indices, Clinical trial information, Imaging technique, Measured Feature, and finally Radiopharmaceutical compound. There are in total 1,221 terms in NIFT. The root concepts of NIFT include

(i) Algorithms which contains 4 children nodes namely: Image acquisition, MR-image analysis, PET-image analysis, and Post-processing algorithm. This concept contains all the brain imaging features that are automatically detected using various imaging pipelines such as FreeSurfer.
(ii) The second root concept in NIFT is Clinical Indices which has two children concepts namely AD and Parkinson’s disease. This includes all the genetic, proteomic biomarkers mentioned in the literature for AD and Parkinson’s disease.
(iii) The third root concept is Clinical trial information which contains three children concepts namely adverse effects observed in patients with neurodegeneration, neuropsychological assessments and scores such as Mini-Mental Status Examination score, Alzheimer’s Disease Assessment Scale-Cog test, and clock draw test to name a few.
(iv) The fourth root concept of NIFT is Imaging Technique. This contains 7 children concepts, each of them represents the different imaging techniques used to study the various structural and functional dimensionality of the brain.
(v) The fifth root concept consists of measured features. This concept covers a wide range of “observable indicators” that determine the state of the brain and disease progression observed using various imaging techniques. This concept includes structural features such as cortical thickness, cerebral atrophy and functional features such as glucose metabolism, blood oxygenation level dependent signal.
(vi) The last root concept consists of radiopharmaceutical compounds. This concept contains all the radioactive tracers that are induced in the brain to diagnose dysfunction.

**Fig.1:**
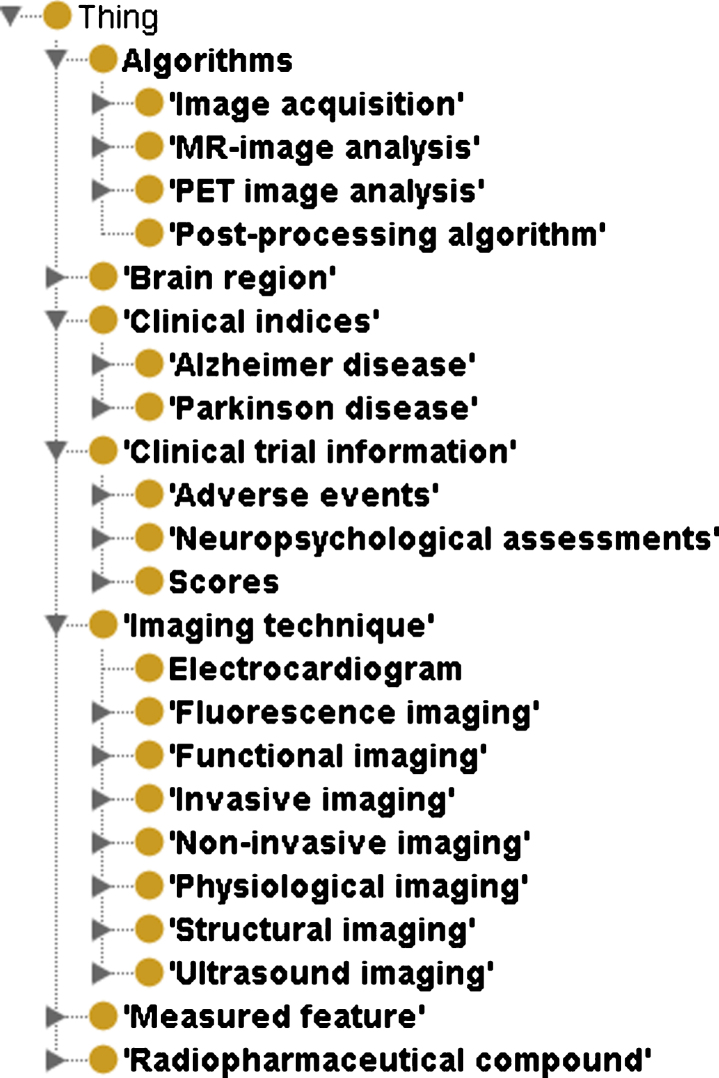
Hierarchical structure of NIFT as visualized in the Protégé OWL Editor. This figure depicts the higher level concepts the terminology namely Algorithms, Brain Region, Clinical Indices, Clinical trial information, Imaging Technique, Measured Feature, and Radiopharmaceutical compound.

NIFT is available in OWL format and can be accessed from the following link (https://www.scai.fraunhofer.de/en/business-research-areas/bioinformatics/downloads.html). The hierarchical structure of NIFT is illustrated in Fig. 1.

### NIFT evaluation

**Fig.2:**
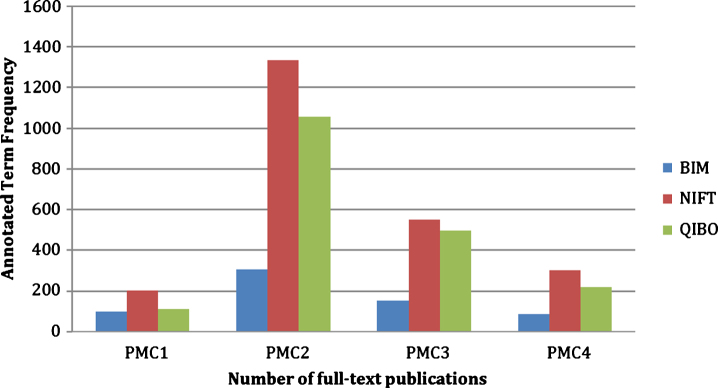
Cross-validation of NIFT terminology against QIBO and BIM. The figure illustrates the evaluation of NIFT by comparing the term relevancy from NIFT, QIBO, and BIM against four full-text PubMed Central articles (PMC1, PMC2, PMC3, and PMC4).

The content evaluation of NIFT in comparison to QIBO and BIM ontologies showed that NIFT performed comparatively better than QIBO and significantly better than BIM in capturing relevant terminology (see Fig. 2). For the first document (PMC1), we found 97 relevant terms annotated by BIM, 204 with NIFT, and 113 with QIBO. The second document (PMC2) was annotated with 308 relevant terms by BIM, 1334 terms by NIFT, and 1056 terms by QIBO. The third document (PMC3) retrieved 153 terms for BIM, 552 terms for NIFT, and 495 terms for QIBO. The fourth and final document (PMC4) retrieved 87 terms for BIM, 303 terms for NIFT, and 217 terms for QIBO. The PMC documents can be found in the Supplementary File 1.

The usability of well-annotated terminology systems can only be considered useful if they are applicable to relevant research. To assess the applicability of NIFT, we have studied the role of image derived indices for diagnosis and how they complement the clinical assessments for better disease prognosis. One of our aims was to establish a (plausible) bridge between clinical, imaging, and cognitive tests which is not only multi-modal, but should enable disease sub-type identification and classification. Our hope is that linking imaging with anatomical as well as diagnostic readouts in AD can help to gain better insight into disease progression and thereby provide more accurate diagnoses.

However, imaging-derived indices information is often scattered throughout the largely unstructured scientific literature, which needs to be analyzed in a systematic manner. Using NIFT, we retrieved 4,029 publications. Out of the 4,029 publications, we filtered 1000 documents that contained at least one quantitative imaging feature, one neuropsychological test at clinic, and a diagnosed stage of AD in corresponding patients (see the query in the Methods section). To exclude false positive documents, we manually curated all the 1000 publications and we found 101 articles that were relevant. For manual curation of documents, we followed a 3-step procedure which are as following:

(i) Only those articles that had informative relationship between neuropsychological assessment and radiological finding were considered for further analysis.
(ii) Articles that only had information about either neuropsychological assessment or radiological findings were not considered for further analysis
(iii) Articles that contained both neuropsychological assessment as well as radiological finding but did not have any meaningful relationship between them, were not considered for further analysis.

The resulting overview shows a pattern based on which imaging technologies and measured features derived from these technologies can be used to categorize the underlying clinical manifestations of patients and thereby links clinical and imaging readouts with the stage of the patient (see Table 1). The overall relation between the quantitative imaging feature, psychological feature, and diagnosis can be found in mentary File 2.

**Table 1:** Mapping imaging derived features to clinical diagnosis and stage in Alzheimer’s disease

PMID	IMAGING	IMAGING FEATURE	RELATION	NEUROPSYCHOLOGICAL	BRAIN REGION	DIAGNOSIS
	TECHNIQUE			ASSESSMENTS
18468705	MRI	Medial Temporal Atrophy	Positive correlation	Mean visual rating scale (5.39)	Temporal lobe	Late AD
18468705	MRI	Medial Temporal Atrophy	Positive correlation	Mean visual rating scale (2.16)	Temporal lobe	Vascular dementia
18468705	MRI	Medial Temporal Atrophy	Positive correlation	Mean visual rating scale (0.56)	Temporal lobe	Healthy
11594917	MRI	Hippocampal atrophy	Correlation	Neuropsychological test (*r* > 0.5)	Hippocampus	Late AD
2061508	DTI	White matter integrity,	Associated	Clinical dementia rating (0.5)	Brain, white matter	Mild AD, EOAD
		brain atrophy
23880336	MRI	Cortical thinning	Associated	Montgomery Asberg Depression	Cortex	Mild AD, LBD
				Rating Scale
12834197	MRI	Hippocampal atrophy,	Associated	CDR (1 and 2)	Hippocampus	Probable AD
		corpus callosum atrophy			Corpus callosum
12834197	MRI	Hippocampal atrophy	Associated	CDR (0.5)	Hippocampus	Mild AD
18400396	MRI	Cortical thinning	Associated	CDR (0.5)	Cortex	Pre-dementia

MRI, magnetic resonance imaging; DTI, diffusion tensor imaging; CDR, Clinical Dementia Rating; AD, Alzheimer’s disease; EOAD, early onset Alzheimer’s disease; LBD, Lewy body disease.

We also conducted a systematical analysis of the heterogeneous imaging techniques and readouts and combining them with the anatomical correlates and clinical endpoints. According to our analysis (as seen in Table 1), the medial temporal atrophy (MTA) as such is a common phenomenon observed in all the three diagnostic classes; however, they could be better distinguished as AD when the MTA score is the highest (5.39). Similarly, the atrophy could be classified as vascular dementia when the MTA score is 2.16 and in case of healthy patients, MTA could still occur, but with a very minimal score (0.56).

### Analysis of literature for neuroimaging features

#### Mining image captions from literature

**Fig.3:**
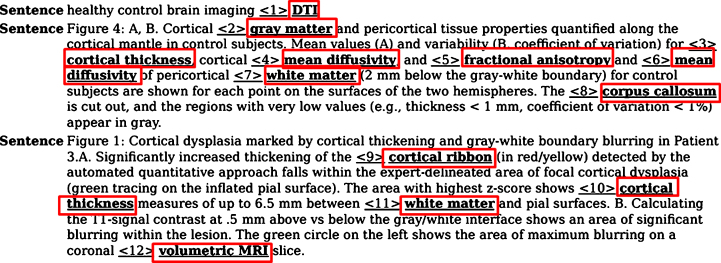
Annotation of an assembly of figure captions with NIFT terminology. This figure showcases the figure captions extracted from publications using NIFT terminology. The red box indicates the NIFT terms present in the figure captions.

Although a fair amount of information on the image-derived findings is usually reported in the abstract of publications, specific features and interpretations gained from brain imaging experiments are often described in the caption of imaging figures that accompany the abstract text in PubMed. We, therefore, tested the relevance and performance of NIFT by applying it to a text mining scenario for analysis of figure legends extracted from publications. A typical example of figure captions annotated using NIFT terms that were extracted from PubMed abstracts is shown in Fig. 3. This figure highlights important quantitative biomarkers such as cortical ribbon which occurs due to the hyperintensity of the cortex observed in patients with early MCI and AD. This radiological sign can be detected using a diffusion tensor imaging technique and fractional anisotropy which is an important measure that demonstrates the connectivity of the brain as well as the tissue characteristics such as myelination and fiber density.

#### Mining full-text publications

**Fig.4:**
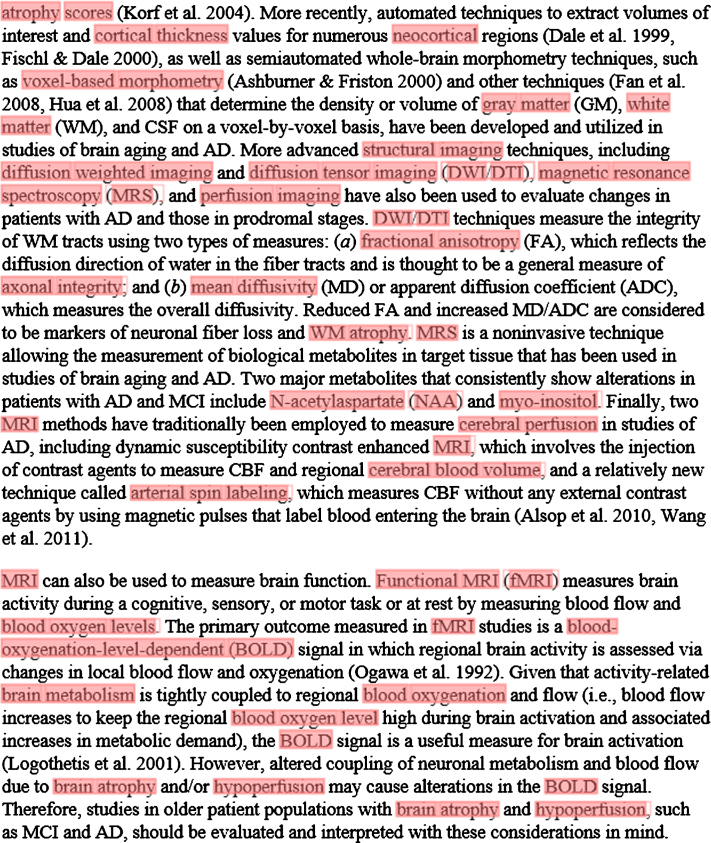
Annotation of a section of a full-text article using the NIFT terminology. The ProMiner tagger was used to identify NIFT terms in full text; matching terms are marked up in red.

In a separate experiment, we annotated a large corpus of full-text publications in order to examine the coverage of NIFT. A typical example for the automated annotation of a section of a full-text publication is shown in Fig. 4. This figure highlights the coverage of the NIFT terms from the full-text publication which includes neuropsychological assessments, brain regions, imaging technique as well as imaging biomarkers. This application demonstrates the usability of NIFT in mining context-specific, full-text publications in the field of neurodegeneration. Retrieval of context-specific, full-text publications can further be used to build a gold-standard corpus in the neurodegeneration domain for generation of novel hypotheses.

#### Annotation of image scans using NIFT

**Fig.5:**
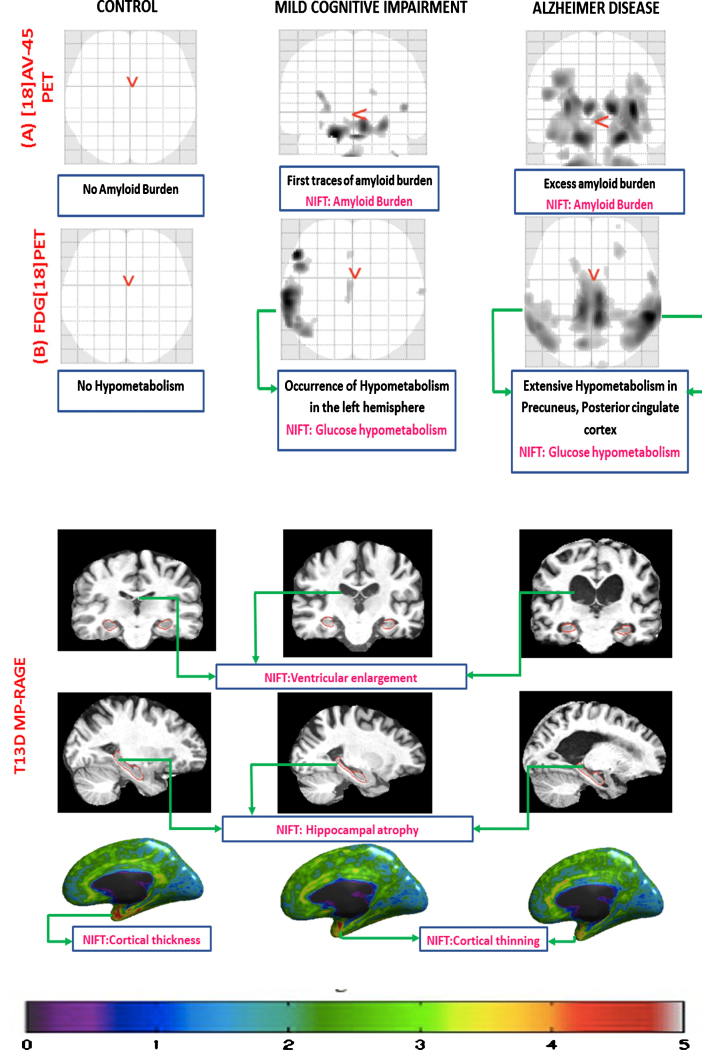
Manual annotation of brain image scans using NIFT. This figure represents different biomarkers captured using three different imaging techniques in control, mild cognitive impairment (MCI), and AD respectively. A) [18] AV-45 PET scan: this figure captures the increased amount of amyloid burden (*p*-value threshold 0.001; voxel extend 10; smoothing kernel [8-8-8]) during the disease progression across CN, MCI, and AD, respectively. B) FDG [18] PET: this figure captures no hypometabolism in control, increased hypometabolic pattern in case of MCI, and extensive hypometabolic topography in the temporo-parietal regions, precuneus, and posterior cingulate cortex (*p*-value threshold 0.001; voxel extend 10; smoothing kernel [8-8-8]). C) T13D MP-RAGE: the first row of the figure demonstrates the progressive ventricular enlargement among control, MCI, and AD respectively. The second row represents progressive hippocampal atrophy across control, MCI, and AD. The third row represents progressive cortical shrinkage in the temporal-parietal lobe, posterior cingulate and precuneus area.

In a separate experiment, we tested, to what extend NIFT terms are suitable for the annotation of primary neuroimaging data (brain scans). Figure 5 depicts the comparison between control, MCI, and AD patient brain scans based on: the amyloid burden through [18] AV45-PET, the regional pattern of hypo-metabolism through FDG-PET, and hippocampal volumetry as well as cortical thickness through T13D MP-RAGE brain scans. The top part of the figure shows the amyloid burden and the hypometabolic clusters across the different brain regions. As it can be seen in Fig. 5, the control does not have any amyloid deposit cluster and no hypometabolism detected, while in MCI, hypometabolic pattern starts to appear in the left hemisphere and more extensively in AD. The expected hypometabolic topography spread across the temporo-parietal regions, precuneus, and posterior cingulate cortex. All the patient-derived image scans can be found in Supplementary File 3.

### Mechanistic modeling of imaging features in the context of AD pathology

**Fig.6:**
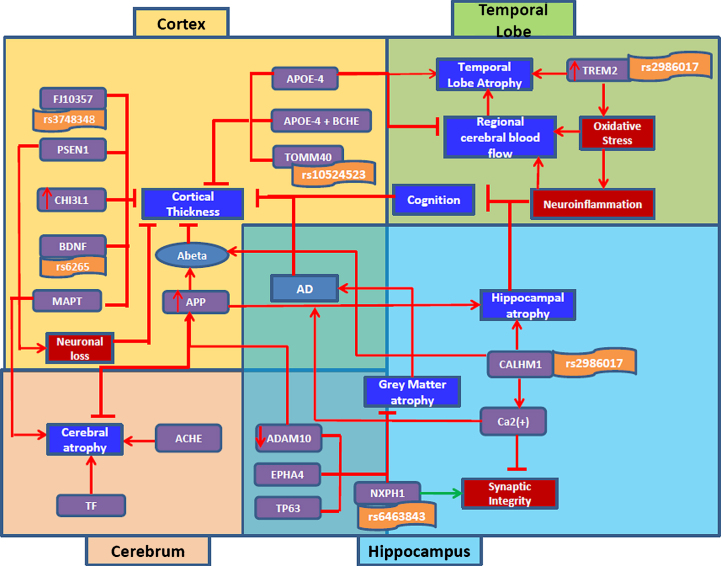
Integrative view of literature-derived associations between molecular and clinical indices in AD through image-derived features. This figure illustrates the complex interaction of genetic players playing a causative/protective role in underlying disease pathology through neuroimaging indices. Top left part of the figure key genetic factors that play a role in shrinking of the cortex eventually leading to AD; top right part of the figure consists of genes involved in neuro-inflammation and temporal lobe atrophy; Bottom left part of the figure displays genes involved in cerebral atrophy; bottom right part consists of genes playing a role in hippocampal and gray matter atrophy. The red color symbol (-|) indicates perturbation of a gene. The red color arrow indicates the function of a gene in disease condition. The green arrow represents the normal process.

Generating links between molecular entities and imaging modalities, even if very demanding and complex, could provide interesting insights into the disease progression as well as help to raise our understanding of the underlying pathology. On that note, we tried to establish that link by querying our in-house SCAIView tool for genes/proteins relevant to imaging features (See Methods section). We retrieved 1,853 gene/protein entities, out of which we identified the top 20 entities confined to interesting brain regions such as cortex, hippocampus, temporal lobe,and cerebrum. Using these entities, we produced the ‘global map’ of genes and imaging features (See Fig. 6). We also inferred from this model that these top ranking genes play a role in cortical thickness, hippocampal atrophy, temporal lobe atrophy, grey matter atrophy and cerebral atrophy, as follows.

### Cortical thickness

Our systematic analysis of the literature revealed that many key players contributed to thinning of the cortex, which is a strong indicator of AD progression. In the following, we demonstrate lines of evidence about factors causally involved in or correlated with cortical thinning and exemplify their corresponding BEL codes:

• Increased expression of APP results in the accumulation of Aβ, which affects the thinning of the cortex [35, 36].

p(HGNC:APP) ->a(“Amyloid beta-Peptides”)

a(“Amyloid beta-Peptides”) –a(NIFT: “Cortical thinning”)

a(NIFT: “Cortical thinning”) ->path(MESHD: “Alzheimer Disease”)

• Increased expression of CHI3L1, a gene responsible for inflammatory response [37, 38], was found to be correlated with cortical thickness [39].

p(HGNC:CHI3L1) ->bp(GO:“inflammatory response”)

bp(GO:“inflammatory response”) negativeCorrelation a(NIFT: “Cortical thickness”)

• PSEN1 was found to cause neuronal loss [40, 41], which results in the shrinkage of the cortex due to neuronal injury [42, 43].

p(HGNC:PSEN1) ->bp (GO:“neuronal loss”)

bp(GO:“neuronal loss”) ->a(NIFT: “Cortical thinning”)

• Well-known genes such as APOE4 along with APOE4 and BCHE carriers contributed to the structural alteration of the cortex, resulting in cortical thinning [44–47].

p(HGNC:APOE) ->a(NIFT: “Cortical thinning”)

p(HGNC:BCHE) ->a(NIFT: “Cortical thinning”)

• Some genes can be linked to cortical thinning through genetics approaches: genes such as FJ10357 [48], TOMM40 [49], and BDNF [50, 51] play a protective role in preserving the structure of the cortex, however, their genetic alteration results in cortical thinning–rs3748348, rs10524523 and rs6265, respectively.

p(HGNC: FJ10357) -> a(NIFT: “Cortical thickness”)

g(dbSNP: rs3748348) – a(NIFT: “Cortical thinning”)

p(HGNC: TOMM40) -| a(NIFT: “Cortical thickness”)

g(dbSNP: rs10524523) – a(NIFT: “Cortical thinning”)

p(HGNC: BDNF) ->a(NIFT: “Cortical thickness”)

g(dbSNP: rs6265) – a(NIFT: “Cortical thinning”)

### Temporal lobe atrophy

We investigated two genes, APOE *ɛ*4 and TREM2, which mainly contribute to the atrophy of temporal lobes. TREM2 is an inflammatory response gene predominantly found in microglia [52, 53]. They are known to enhance phagocytosis as well as maintaining cytokine production so that inflammatory responses can be triggered by TREM-1, a novel receptor expressed on neutrophils and monocytes [54, 55]. However, the genetic mutation of TREM2, rs75932628, causes the atrophy of temporal lobes through enhancing oxidative stress [56], which in turn causes the reduction of cerebral blood flow leading to reduction in regular supply of oxygen, glucose, and other nutrients to the temporal lobe, and finally the shrinkage of the temporal lobe [57, 58]. On the other hand, increased expression of APOE *ɛ*e4 allele affects the flow of cerebral blood, further contributing to atrophy [59, 60].

### Hippocampal atrophy

Through our work, we identified an interesting gene, CALHM1 which was known to regulate Aβ clearance [61] through the activation of insulin-degrading enzyme [62]. However, a genetic mutation by rs2986017 results in (i) loss of hippocampal neurons further causing atrophy as well as (ii) increased Aβ levels and altered calcium homeostasis which could result in reduced synaptic integrity and mitochondrial dysfunction [63].

### Grey matter atrophy

Here, we identified a gene namely NXPH1, which was found to play a role in adhesion of dendrites and axons and maintaining synaptic integrity. However, the mutation of the gene, rs6463843, affects the synaptic integrity and results in loss of grey matter density leading to atrophy [64]. Apart from that, EPHA4 was also found to play a protective role in the glial glutamate transport that ultimately regulates hippocampal function as well as the maintenance of grey matter density [65, 66].

### Cerebral atrophy

Cerebral atrophy was found to be regulated by two key players— Transferrin as well as ACHE. Transferrin was found to play a significant role in iron homeostasis [67]. However, the alteration of the gene could result in iron overload which causes damage to the cerebral structure, ultimately leading to cerebral atrophy [68, 69]. On the other hand, the altered function of ACHE in the cholinergic system could result in the loss of cerebral neurons leading to cerebral atrophy [70]. The computer readable BEL version of this model is found in Supplementary File 4.

## DISCUSSION

A “biomarker” is an accurately measured medical sign that indicates the medical state of the patient. However, in the field of imaging, this term is often misinterpreted due to the lack of standardization of terminology and concepts. Often, literature resources misclassify an imaging technique as a biomarker while many others denote the derived indices as a biomarker [71, 72]. Owing to this, NIFT was constructed to represent, integrate, and harmonize heterogeneous knowledge across the domain of imaging biomarkers in the context of neurodegeneration. NIFT serves as a single resource of the standard terminology that describes the domain of neuroimaging biomarkers in a hierarchical manner and has been designed to capture relevant image-derived features (“indices”) with high specificity and granularity. As shown by our analysis, what distinguishes NIFT from other existing resources is the inclusion of various concepts ranging from algorithms that automate the process of measuring features to radiological tracers that help in revealing functional alterations of the brain. Such a standard reference terminology has the potential not only to support organization and exchange of imaging information among neurologists and clinical researchers but also to provide a useful tool for annotation of brain scan metadata as detection of meta-information in brain scans helps inferring neuroanatomical relationships present in imaging data [73]. With such an inventory, it is indeed possible to automatically extend the annotation of scans by incorporating NIFT in image annotation tools. Since NIFT combines specificity and granularity of imaging features in the context of neurology knowledge domain, users can intuitively navigate through different levels of concept granularity within a search engine and for instance, perform faceted search in literature mining environments.

With respect to the contextual specificity, as benchmark analysis of NIFT against two other highly domain-specific, relevant terminologies showed the overall granularity of medical relevant terms and cognitive tests in NIFT was comparably high, making NIFT a reference terminology resource specific to neuroimaging. The applicability of NIFT could be extended toward information retrieval and extraction. As demonstrated earlier, using NIFT for literature mining improves retrieval of the relevant, informative neuroimaging publications and supports curation and extraction of captured information from unstructured text. In the presence of other terminology sources, powerful filtering for faceted searches can be implemented. For instance, we can combine NIFT with HypothesisFinder [74] to systematically harvest speculative statements linked to imaging features; or combination of NIFT terms with ADO terms will allow us to systematically harvest factual statements that link imaging readouts to aspects of AD progression in literature; and finally, we also have the possibility of mining “shared imaging features” amongst other diseases by making use of the already integrated Parkinson Disease Ontology [75] and Multiple Sclerosis Ontology [76]. This could lead to domain specific imaging feature identification across disease scales.

Importantly, the usage of NIFT is not limited to information retrieval and extraction. Since the major mission of the neuroscience community currently is to bridge the gap between molecular mechanisms and imaging readouts, NIFT can be used to address this challenge by bringing context to computational modeling efforts. To demonstrate this possibility, we showed how NIFT serves as a valuable resource to support mechanistic modeling of complex AD pathomechanisms. As highlighted in Fig. 6, this high resolution mechanistic model captures novel genetic players such as CALHM1, NXPH1, and ADAM10, which cause hippocampal atrophy through neuronal loss. Here, we identified the various roles played by CALHM1 in AD pathology, ranging from controlling cytosolic Ca(2+) concentrations and Aβ levels to increased oxidative stress through glutamatergic neurotransmission inhibition [77, 78]. Similarly, another two novel genetic biomarkers were CHI3L1 and CAND1. CHI3L1, a protein that encodes YKL-40, was found to be associated with cortical thinning and was found to play a role in neuroinflammatory response. They were found to play a role in cell morphology and behavior; however, their association with susceptibility to AD has only been recently studied [79].

The current neuropathological studies on AD suggest that the clinical onset of the disease goes decades before the formation of neurofibrillary tangles and Aβ plaques [80, 81]. This brings up the need for heterogeneous measurable indicators that can aid systematic tracing of alternative patterns of disease progression. ADNI have positioned themselves as pioneers in assembling patient records with cognitive and longitudinal assessments along with genetic and fluid sample measures. This interesting combination of measured metadata could provide unique insights into measurable signs before the expected onset of the disease. However, the challenge still remains to identify those patterns at an earlier stage through the use of combinatorial features. Furthermore, we foresee the option to perform systematic association studies in the literature between SNPs and mutations on one side and imaging features on the other side (Iyappan et al., in preparation). The multi-level association between genetic factors and clinical readouts can be directly used for modeling and mining across scales in the neurology and psychiatry field. A first attempt of demonstrating such systematic harvesting approach is the association of imaging features with cognition readouts (refer to Table 1). Such associations lead to comprehensive analysis of imaging features correlating with cognition.

To the best state of our knowledge, NIFT is the first reference compendium, which apprehends the various aspects of the derived quantitative measures from neuroimaging scans. We invite the scientific community to contribute to edition and enrichment of NIFT so that it can be leveraged to the level of a formal ontology in future.

### Conclusion

To our knowledge, there have been little efforts invested so far in the direction of standardizing and capturing observable clinical imaging features, particularly in the neurology domain. Through this work, we attempted to bridge “omics” and imaging/clinical level data. This type of integration across scales is often regarded as the “holy grail” of integrative modeling and mining. Future approaches should be able to represent and model the disease progression in a longitudinal model by integrating molecular processes and imaging features over time, provided that longitudinal data capture would be extended to other omics data types beside imaging. For this purpose, we obviously need trajectories. Currently, the BEL modeling framework does not deliver this time dimension. However, we are working towards the extension of BEL by a time dimension. A long term perspective of this extension is the vision of a virtual patient cohort that comprises several such longitudinal “trajectories” representing the dynamics of important imaging features. The link between imaging and genetics will be a cornerstone for the construction of the virtual cohort; the generation of a “virtual dementia cohort” has recently been made a task in IMI-project AETIONOMY and we will see first results of the simulation of entire trials based on a “virtual dementia cohort” in the near future. The imaging derived features captured through NIFT will have a major role in that “virtual dementia cohort”. We believe it would be desirable to generate a “metadata atlas” of the brain populated with NIFT concepts. Such an atlas could serve as a template for qualitative models that integrate imaging features from different, heterogeneous studies.

## Supplementary Material

Supplementary file 1

Supplementary file 2

Supplementary file 3

Supplementary file 4
