# Supplementary material for: Neuroimaging Feature Terminology: A Controlled Terminology for the Annotation of Brain Imaging Features
Source: J Alzheimers Dis. 2017 Aug 14;59(4):1153–69. doi: 10.3233/JAD-161148 (PMC5611802; doi:10.3233/JAD-161148)
Supplement: Supplementary file 1 [file jad-59-jad161148-s001.zip › Supplementary_File1/Neuroimaging and Other Biomarkers for Alzheimer's Disease.pdf]

NIH-PA Author Manuscript

NIH-PA Author Manuscript

NIH-PA Author Manuscript

NIH Public Access
Author Manuscript
Annu Rev Clin Psychol. Author manuscript; available in PMC 2014 March 15.

Published in final edited form as:
Annu Rev Clin Psychol. 2013 ; 9: 621–648. doi:10.1146/annurev-clinpsy-050212-185535.

Neuroimaging and Other Biomarkers for Alzheimer's Disease: The Changing Landscape of Early Detection

Shannon L. Risacher and **Andrew J. Saykin**
Center for Neuroimaging, Department of Radiology and Imaging Sciences, Indiana University School of Medicine, Indianapolis, Indiana 46202; srisache@iupui.edu, asaykin@iupui.edu

Abstract

The goal of this review is to provide an overview of biomarkers for Alzheimer's disease (AD), with emphasis on neuroimaging and cerebrospinal fluid (CSF) biomarkers. We first review biomarker changes in patients with late-onset AD, including findings from studies using structural and functional magnetic resonance imaging (MRI), advanced MRI techniques (diffusion tensor imaging, magnetic resonance spectroscopy, perfusion), positron emission tomography with fluorodeoxyglucose, amyloid tracers, and other neurochemical tracers, and CSF protein levels. Next, we evaluate findings from these biomarkers in preclinical and prodromal stages of AD including mild cognitive impairment (MCI) and pre-MCI conditions conferring elevated risk. We then discuss related findings in patients with dominantly inherited AD. We conclude with a discussion of the current theoretical framework for the role of biomarkers in AD and emergent directions for AD biomarker research.

Keywords

mild cognitive impairment (MCI); preclinical Alzheimer's disease (AD); magnetic resonance imaging (MRI); functional MRI (fMRI); positron emission tomography (PET); cerebrospinal fluid (CSF); genetics

INTRODUCTION

Alzheimer's disease (AD) is the most common age-related neurodegenerative disease. Characterized by progressive cognitive decline and dementia, AD affects more than 5 million individuals in the United States, most aged 65 or older, and that number is expected to more than triple by 2050 (Alzheimer's Assoc. 2012). Currently no treatments effectively target the underlying pathology associated with AD. Research and evaluation of potential therapeutic targets are ongoing, and it is hoped that effective therapies that ameliorate or prevent the effects of AD will be developed.

The goal of this article is to review current findings regarding the role of neuroimaging and other biological markers (biomarkers) in AD, especially in the prodromal stages of disease, such as mild cognitive impairment (MCI) and preclinical AD. In each section, structural and functional magnetic resonance imaging (MRI) and positron emission tomography (PET) techniques and nonimaging biomarkers [i.e., cerebrospinal fluid (CSF) measures] are discussed. First, we briefly review the findings in patients with late-onset AD. However,

Copyright © 2013 by Annual Reviews. All rights reserved

DISCLOSURE STATEMENT The authors are not aware of any affiliations, memberships, funding, or financial holdings that might be perceived as affecting the objectivity of this review.

NIH-PA Author Manuscript
NIH-PA Author Manuscript
NIH-PA Author Manuscript

because the emphasis of this review is on early indicators of AD, the majority of this review focuses on findings in patients in the prodromal stages of AD. Thus, the next two sections discuss neuroimaging and nonimaging biomarkers in patients with MCI and in preclinical AD samples, respectively. Next, we examine findings from biomarker studies in patients with dominantly inherited AD. Then, we explore the current theoretical framework for the role of biomarkers in AD research that was proposed by Jack et al. (2010b). Finally, we discuss new and future directions in the field of AD biomarkers. We emphasize findings from large studies where possible, including consortium imaging studies such as the Alzheimer's Disease Neuroimaging Initiative (ADNI). ADNI is designed to evaluate the role of longitudinal neuroimaging measures (MRI, PET), CSF and blood-based protein measures, neuropsychological and clinical testing, and genetic variation in detecting AD-related biological changes, especially in prodromal stages, and predicting future disease progression. Further information about ADNI can be found at http://www.adni-info.org and in recent reviews (Aisen et al. 2010; Beckett et al. 2010; Jack et al. 2010a; Jagust et al. 2010; Saykin et al. 2010; Trojanowski et al. 2010; Weiner et al. 2010, 2011). Results from other large collaborative studies of AD, such as the Australian Imaging, Biomarker & Lifestyle Flagship Study of Ageing and the Dominantly Inherited Alzheimer Network, are also included, as they provide data from large samples about the utility of biomarkers, cognitive measures, lifestyle factors, and genetics in the detection and development of AD.

Neuropathology of AD

AD is characterized by two neuropathological hallmarks: amyloid plaques and neurofibrillary tangles (NFTs). Amyloid plaques are extracellular aggregations of the amyloid-beta (Aβ) peptide that are found throughout the brain of AD patients. Recent studies suggest that the accumulation of Aβ in late-onset Alzheimer's disease (LOAD) may result primarily from imbalanced or ineffective Aβ clearance rather than excess formation (Mawuenyega et al. 2010, Sperling et al. 2011). Aβ oligomers are thought to be a major neurotoxic species in the brains of patients with AD (Walsh & Selkoe 2007). Ultimately, Aβ oligomers and soluble fragments become large fibrils, which further aggregate to form insoluble deposits in the extracellular space, including small diffuse plaques and dense core plaques, which are a hallmark of AD neuropathology. Insoluble Aβ plaques may also be neurotoxic, although the mechanisms by which this occurs are still not fully understood. NFTs result from the hyperphosphorylation of the microtubule-associated protein tau. Hyperphosphorylated tau undergoes a conformational change preventing normal binding to microtubules, resulting in destabilization of axons, impairment of axonal transport, axonal degeneration, neuron dysfunction, and ultimately cell death. In addition, the hyperphosphorylated tau forms insoluble filamentous structures that combine to create paired helical filaments, a key component of the NFTs seen in the brains of patients with AD (Iqbal & Grundke-Iqbal 2008). The underlying cause of this abnormal hyperphosphorylation is currently unknown but is likely due to a disruption in the balance between the kinases and phosphatases regulating tau phosphorylation (Iqbal & Grundke-Iqbal 2008).

The temporal relationship and direct link between amyloid plaques and NFTs are not completely elucidated at this time. Current theories suggest that amyloid plaque formation precedes NFTs, with amyloid accumulation occurring during a long preclinical period lasting years to decades (Jack et al. 2010b) (Figure 1). However, the neurodegeneration, synaptic loss, and cognitive symptoms seen in AD patients are more strongly associated with the formation and extent of NFTs than with amyloid plaque deposition (Bennett et al. 2004). The current framework of AD development suggests that amyloid accumulation initiates a pathological cascade resulting in the formation of NFTs and other pathologies (Jack et al. 2010b). The formation of NFTs and toxic Aβ species (e.g., oligomers), as well as

NIH-PA Author Manuscript NIH-PA Author Manuscript NIH-PA Author Manuscript

the initiation of other apoptotic cascades, leads to widespread neuronal injury and death and, thus, the clinical symptoms associated with the disease.

The biochemical processes involved in AD development ultimately converge upon widespread cell death and neuronal loss, likely through apoptosis. The stages of neurodegeneration in AD are described in detail by Braak and colleagues (1993). The first regions of the brain to show neuronal loss associated with AD are in the medial temporal lobe (MTL), including the entorhinal cortex (EC), hippocampus, amygdala, and parahippocampal cortex. Additionally, extensive degeneration of the cholinergic innervations to the neocortex from the basal nucleus of Meynert and the medial septal nucleus occurs early in the disease process (Braak et al. 1993). The next stage of degeneration usually involves neuronal loss in the cerebral cortex, particularly in the lateral temporal and medial parietal cortices, followed by atrophy of the lateral parietal and frontal lobes. By the time a patient has reached a diagnosis of AD, neurodegeneration is usually found throughout the neocortex and subcortical regions, with significant atrophy of the temporal, parietal, and frontal cortices, but relative sparing of the primary occipital cortex and primary sensory-motor regions (Braak et al. 1993).

Clinical Symptoms and Diagnosis

The earliest clinical symptoms associated with AD are a direct result of the brain regions to first degenerate, namely the MTL. Memory impairments, particularly in episodic and semantic domains, as well as deficits in language and executive functioning are common symptoms early in the disease course (McKhann et al. 2011). Patients with mild AD also show a significant impairment in daily functioning with disruption or cessation of the ability to perform complex tasks associated with general life (e.g., balancing a checkbook, workplace performance, and social activities). In moderate to severe AD, patients may show an inability to function independently, with difficulties in even simple daily tasks, such as feeding and dressing.

Clinicians and researchers have recently updated the AD diagnostic criteria for use in clinical practice and research (McKhann et al. 2011). Currently, the diagnosis of AD is made clinically, based on cognition and the relative impact of impairments on daily activities. However, biomarkers (e.g., CSF protein levels, neuroimaging) may be used to rule out other causes of dementia (e.g., vascular) and to support the AD diagnosis in cases with unclear or atypical presentations. Attempts to diagnose AD at an earlier stage have led to the development of a clinical syndrome termed mild cognitive impairment (MCI) (Petersen et al. 1999). Recently, new criteria for diagnosis of MCI in clinical and research settings have been published (Albert et al. 2011). A patient must have the following symptoms to receive a clinical diagnosis of MCI: (*a*) concern regarding a change in cognition (by the patient, an informant, and/or a skilled clinician); (*b*) impairment in one or more cognitive domains (memory, executive function, attention, visuospatial, language) greater than expected for age and education level; (*c*) preservation of independence in functional activities, meaning a patient may show mild problems performing complex functional tasks but they should be able to generally maintain their independence with minimal aid or assistance; and (*d*) changes should be sufficiently mild so that there is no significant impairment in occupational or social functioning (i.e., not demented). Currently, biomarkers (e.g., CSF protein levels, neuroimaging) are used in the diagnosis of MCI only in clinical settings to rule out alternative etiologies of cognitive impairment. However, diagnosis of MCI in research settings may include the use of biomarkers to support the underlying cause of the MCI and to predict the type and rate of future disease progression. Patients with MCI typically show deficits in cognition that fall between 1 and 2 standard deviations below age- and education-adjusted and culturally appropriate normative levels (Albert et al. 2011).

NIH-PA Author Manuscript NIH-PA Author Manuscript NIH-PA Author Manuscript

More recently, researchers have proposed dividing MCI into an earlier stage [early MCI (E-MCI)] and a later stage [late MCI (L-MCI)], with E-MCI patients showing a 1–1.5 standard deviation cognitive deficit and L-MCI showing a 1.5–2 standard deviation deficit. This classification has only recently been introduced, and future studies will help to elucidate differences between these MCI subgroups. The most common presentation of MCI features memory impairment (amnestic MCI). Executive impairments are also commonly reported and can co-occur with other cognitive deficits (multidomain MCI) (Albert et al. 2011). Amnestic MCI is widely considered to be a prodromal form of AD, as nearly 10% to 15% of amnestic MCI patients convert to probable AD each year, relative to only 1% to 2% of the general elderly population (Petersen et al. 1999).

Recently, researchers and clinicians have been attempting to detect AD-related changes and predict progression even earlier than MCI (e.g., pre-MCI or preclinical AD). A recent article presents a conceptual framework for identifying preclinical AD patients (Sperling et al. 2011). This framework was designed to categorize and conceptualize the presence of AD pathology in seemingly healthy older adults. Approximately 20% to 40% of older adults show extensive amyloid pathology with minimal or no clinical AD symptoms (Mintun et al. 2006, Sperling et al. 2011). A time lag of 10 to 15 years between the initial development of AD pathology and the emergence of AD clinical symptoms is thought to be common (Figure 1). Thus, cognitively normal participants with AD pathology are considered to be at increased risk for progression to clinical AD. Other researchers define preclinical AD patients using other characteristics, such as subjective reports of cognitive changes (i.e., cognitive complaints), *APOE* genotype, and/or family history of dementia (Dik et al. 2001, Saykin et al. 2006). All of these factors have been shown to be linked with increased risk for future progression to AD (Corder et al. 1993, Jessen et al. 2010, Reisberg et al. 2010).

Biomarkers of AD

Early detection of AD is an important goal because future treatments will likely target disease prevention or slowing of AD development rather than reversal of AD-related neuronal damage. Therefore, these interventions would likely be maximally effective in the prodromal stages of the disease (Figure 1). Sensitive and specific biomarkers of AD are needed to detect patients in the early and preclinical stages of AD, to effectively monitor and predict disease progression, and to provide differential diagnostic information for an accurate diagnosis. Biomarkers are also important for clinical trials of new pharmaceutical interventions to enrich the sample with patients most likely to progress to AD and monitor the outcome of new treatments. Levels of AD-related proteins (e.g., $A\beta_{40}$, $A\beta_{42}$, total tau, phosphorylated tau) measured from the CSF are commonly used as biomarkers in AD research (de Leon et al. 2007, Shaw et al. 2009). Neuroimaging provides an excellent noninvasive set of methods for measuring in vivo AD pathophysiology and brain atrophy associated with MCI and AD, as well as for predicting disease progression, even in patients with relatively minor or no cognitive impairments (e.g., preclinical AD patients) (de Leon et al. 2007, Jack et al. 2010b, Weiner et al. 2011).

Neuroimaging Biomarkers

The two types of neuroimaging most commonly used as AD biomarkers include MRI and PET. The most widely used neuroimaging technique to investigate structural changes and neurodegeneration associated with AD in vivo is structural MRI. A number of studies have investigated differences between AD patients, MCI patients, and age-matched healthy controls (HCs) on measures of global and local brain volume, tissue morphology, and rate of atrophy using both manually applied and automated techniques. Historically, manually applied techniques were used to extract volumetric and morphometric characteristics, including manual tracing of regions of interest (Jack et al. 1997) and medial temporal

NIH-PA Author Manuscript NIH-PA Author Manuscript NIH-PA Author Manuscript

atrophy scores (Korf et al. 2004). More recently, automated techniques to extract volumes of interest and cortical thickness values for numerous neocortical regions (Dale et al. 1999, Fischl & Dale 2000), as well as semiautomated whole-brain morphometry techniques, such as voxel-based morphometry (Ashburner & Friston 2000) and other techniques (Fan et al. 2008, Hua et al. 2008) that determine the density or volume of gray matter (GM), white matter (WM), and CSF on a voxel-by-voxel basis, have been developed and utilized in studies of brain aging and AD. More advanced structural imaging techniques, including diffusion weighted imaging and diffusion tensor imaging (DWI/DTI), magnetic resonance spectroscopy (MRS), and perfusion imaging have also been used to evaluate changes in patients with AD and those in prodromal stages. DWI/DTI techniques measure the integrity of WM tracts using two types of measures: (*a*) fractional anisotropy (FA), which reflects the diffusion direction of water in the fiber tracts and is thought to be a general measure of axonal integrity; and (*b*) mean diffusivity (MD) or apparent diffusion coefficient (ADC), which measures the overall diffusivity. Reduced FA and increased MD/ADC are considered to be markers of neuronal fiber loss and WM atrophy. MRS is a noninvasive technique allowing the measurement of biological metabolites in target tissue that has been used in studies of brain aging and AD. Two major metabolites that consistently show alterations in patients with AD and MCI include N-acetylaspartate (NAA) and myo-inositol. Finally, two MRI methods have traditionally been employed to measure cerebral perfusion in studies of AD, including dynamic susceptibility contrast enhanced MRI, which involves the injection of contrast agents to measure CBF and regional cerebral blood volume, and a relatively new technique called arterial spin labeling, which measures CBF without any external contrast agents by using magnetic pulses that label blood entering the brain (Alsop et al. 2010, Wang et al. 2011).

MRI can also be used to measure brain function. Functional MRI (fMRI) measures brain activity during a cognitive, sensory, or motor task or at rest by measuring blood flow and blood oxygen levels. The primary outcome measured in fMRI studies is a blood-oxygenation-level-dependent (BOLD) signal in which regional brain activity is assessed via changes in local blood flow and oxygenation (Ogawa et al. 1992). Given that activity-related brain metabolism is tightly coupled to regional blood oxygenation and flow (i.e., blood flow increases to keep the regional blood oxygen level high during brain activation and associated increases in metabolic demand), the BOLD signal is a useful measure for brain activation (Logothetis et al. 2001). However, altered coupling of neuronal metabolism and blood flow due to brain atrophy and/or hypoperfusion may cause alterations in the BOLD signal. Therefore, studies in older patient populations with brain atrophy and hypoperfusion, such as MCI and AD, should be evaluated and interpreted with these considerations in mind. fMRI studies in patients with MCI and AD often measure brain activity during cognitive tests, particularly in memory, executive function, and language domains. In addition to estimates of regional task-related brain activity, quantification of brain networks can provide a unique measure of brain activity. Techniques for quantifying brain connectivity from fMRI data have recently been developed and applied in studies of brain aging during functional activation (i.e., during performance of tasks) as well as during resting or task-free states (Greicius et al. 2003).

PET uses radiolabeled ligands to measure metabolic and neurochemical processes in vivo. In AD research, two types of PET ligands are primarily utilized: (*a*) $[^{18}F]$fluorodeoxyglucose (FDG), which measures brain metabolism (Jagust et al. 2010, Langbaum et al. 2009); and (*b*) amyloid tracers, including $[^{11}C]$Pittsburgh compound B (PiB) and $[^{18}F]$Florbetapir, which bind to fibrillar amyloid plaques (Klunk et al. 2004, Vandenberghe et al. 2010, Wong et al. 2010). The most commonly used amyloid tracer in studies of AD and MCI patients to date is $[^{11}C]$PiB, which has been shown to bind to both neuritic and diffuse plaques (Klunk et al. 2005) as well as to cerebral amyloid (Johnson et al. 2007) within the vasculature. The

NIH-PA Author Manuscript

NIH-PA Author Manuscript

NIH-PA Author Manuscript

primary signal measured in studies with [^{11}C]PiB is the result of specific binding to amyloid plaques, with a high correlation between the anatomical distribution of [^{11}C]PiB binding and post-mortem amyloid (Ikonomovic et al. 2008). Other PET studies in AD patients using a wide variety of ligands have been reported, including those that measure neuroinflammation and activated microglia (e.g., [^{11}C]PK11195 and [^{11}C]DAA1106) (Banati et al. 2000, Chaki et al. 1999) and neurotransmitter and enzyme activity [e.g., acetylcholine (ACh), GABA, dopamine, serotonin, and acetylcholinesterase (AChE)] (Pappata et al. 2008).

BIOMARKERS OF PATHOLOGY IN ALZHEIMER'S DISEASE

As stated above, structural MRI is the most widely used neuroimaging technique to investigate structural changes and neurodegeneration. MRI estimates of regional volumes, extracted using either manual or automated techniques, as well as global and regional tissue morphometry show significant brain atrophy in AD patients, following an anatomical distribution similar to the pattern reported in Braak et al. (1993) according to disease severity. A number of studies have investigated atrophy in AD participants in the ADNI sample and found a pattern of widespread atrophy, including in the MTL and lateral temporal lobe, medial and lateral parietal lobe, and the frontal lobe, with relative sparing of the occipital lobe and sensory-motor cortex (Jack et al. 2010a, Risacher et al. 2009, Weiner et al. 2011) (Figure 2*a*). Longitudinal studies have shown high rates of cortical atrophy in patients with AD, particularly in the temporal lobe. Patients with AD show an approximate annual hippocampal decline of 4.5% relative to only an approximate 1% annual decline in HC (for meta-analysis, see Barnes et al. 2009).

Advanced MRI techniques have also been used in studies of patients with AD. DWI/DTI studies indicate that AD patients show reduced FA and increased MD/ADC relative to HCs in many WM structures throughout the brain (Ibrahim et al. 2009). MRS techniques show that AD patients have decreased NAA levels and increased myo-inositol relative to HCs throughout the brain, with the most significant changes in the temporal lobe and hippocampus (Chantal et al. 2004, Schuff et al. 2002). Studies of brain perfusion with MRI have consistently demonstrated decreased perfusion or "hypoperfusion" in patients with AD, particularly in temporoparietal regions, as well as frontal, parietal, and temporal cortices (Harris et al. 1998).

Results from fMRI studies in AD patients have shown conflicting results. Most studies with AD patients have shown decreased or even absent activation relative to HCs in the hippocampus, MTL, posterior cingulate, parietal lobe, and frontal lobe during episodic memory encoding and recall tasks (Celone et al. 2006, Dickerson et al. 2005) (Figure 3*a*). Alternatively, a small number of studies have suggested increased activation during episodic memory encoding and recall in the posterior cingulate, precuneus, parietal lobe, and frontal lobe (Pariente et al. 2005). Functional connectivity studies have also shown alterations in patients with AD, including decreased connectivity in task-related and resting-state networks (Celone et al. 2006, Grady et al. 2003, Zhou et al. 2008). In particular, a network of brain regions that includes the medial parietal lobe, MTL, and medial frontal lobe is deactivated upon task initiation. This network, which is referred to as the default mode network (DMN) (Buckner et al. 2005, Greicius et al. 2003), shows decreased activity at rest, decreased connectivity, and reduced deactivation upon task initiation in AD patients (Buckner et al. 2005, Celone et al. 2006, Grady et al. 2003, Greicius et al. 2004) (Figure 3*b*). The observed decreased functional connectivity is consistent with DTI studies showing decreased structural connectivity (i.e., impaired integrity of WM tracts) within and between the memory and resting-state networks (Zhou et al. 2008).

NIH-PA Author Manuscript

FDG PET studies of patients with AD have shown significant reductions in cerebral metabolism relative to HC, specifically in the temporoparietal cortex, posterior cingulate, parietal lobe, temporal lobe, and in the MTL, including the hippocampus (Buckner et al. 2005, Langbaum et al. 2009, Mosconi 2005) (Figure 2*b*). In addition, more impaired AD patients also show hypometabolism in the frontal lobe and prefrontal cortex relative to less impaired patients and HCs (Herholz et al. 1999, Mosconi 2005). Longitudinal studies also show a faster rate of annual decline in metabolism in the temporal, parietal, and frontal lobes, as well as the posterior cingulate and precuneus in AD patients relative to HCs (Alexander et al. 2002, Small et al. 2000).

Amyloid imaging PET studies in patients with AD have shown significantly higher uptake and binding of amyloid tracers (most commonly $[^{11}C]$PiB) relative to HC in brain regions known to show amyloid deposition, including the frontal, temporal, and parietal lobes, posterior cingulate, and precuneus (Jack et al. 2008, Klunk et al. 2004) (Figure 2*c*). Across $[^{11}C]$PiB studies, 96% of AD patients show significant amyloid accumulation, measured as a "positive" $[^{11}C]$PiB signal (Johnson et al. 2012). Longitudinal assessments of amyloid using $[^{11}C]$PiB in AD patients have shown minimal increases in $[^{11}C]$PiB signal over 1–2 years in patients who showed significant $[^{11}C]$PiB signal at baseline (Jack et al. 2009, Klunk et al. 2006). Thus, researchers have tentatively concluded that amyloid deposition occurs earlier in the disease, and by the time cognitive decline occurs and a diagnosis of AD is made, brain amyloid burden is relatively stable and increased deposition is minimal. There is great interest in development of PET tracers for tau and other abnormal proteins beyond beta amyloid, but these developments are presently at an early stage.

In addition to evaluating cerebral metabolism and the presence of amyloid, researchers have investigated specific alterations in neurotransmitter systems and neuroinflammation using PET. Using PET techniques with tracers specific for AChE as a surrogate measure for ACh synaptic density, significant reductions in binding were found in AD patients in the MTL, including the hippocampus and amygdala, as well as in the frontal, parietal, and temporal lobes and cingulate cortex (Rinne et al. 2003). Neuroimaging of neurotransmitter systems other than ACh has been relatively limited. However, studies in AD patients have shown decreases in GABA, serotonin, and dopamine synaptic densities (Pappata et al. 2008). In addition to the accumulation of amyloid and hyperphosphorylated tau, AD patients show a large amount of activated microglia, particularly surrounding amyloid plaques (Haga et al. 1989). Tracers have been developed (i.e., $[^{11}C]$PK11195 and $[^{11}C]$ DAA1106) that show specific binding to activated microglia in vitro, as well as in the living brain (Chaki et al. 1999, Venneti et al. 2009). Studies of neuroinflammation have shown significantly elevated global and regional binding of both tracers in patients with AD relative to HCs, particularly in the MTL, including the amygdala and EC, as well as in the frontal lobe, parietal lobe, lateral temporal lobe, occipital lobe, anterior cingulate, and cerebellum (Edison et al. 2008, Okello et al. 2009). However, other studies have shown minimal binding of these tracers in patients with AD relative to HCs (Wiley et al. 2009). These differences likely reflect conflicting quantification methodologies, and future studies will likely elucidate the role of neuroinflammation in AD and MCI, as well as utility of this technique as a biomarker.

The major protein species assessed in CSF studies of AD patients are levels of $A\beta_{40}$, $A\beta_{42}$, total tau, and phosphorylated tau (p-tau). Patients with AD show a significantly decreased level of $A\beta_{40}$ and $A\beta_{42}$ and increased level of total tau and p-tau relative to HCs (Shaw et al. 2007, 2009; Trojanowski et al. 2010). Other CSF measures, including assessment of isoprostane and homocysteine, have also shown some elevations in patients with AD (Shaw et al. 2007, Trojanowski et al. 2010).

NIH-PA Author Manuscript

NIH-PA Author Manuscript

NIH-PA Author Manuscript

Overall, the findings from studies of patients with AD show widespread and significant neurodegeneration in both gray and white matter, altered brain perfusion and neurochemical properties, alterations in brain function during tasks and at rest, reduced brain glucose utilization, significant amyloid accumulation, notable neuroinflammation, a loss of neurotransmitter activity and receptor density, and an accumulation of Aβ and tau proteins in the CSF. All of these studies suggest that significant brain pathology has accumulated by the time of an AD diagnosis, including widespread cell death. Therefore, identification of patients at risk for the development of AD before the extensive amyloid and neurodegenerative pathology occurs may provide clues about disease mechanisms as well as provide a window in which to intervene and prevent disease progression.

BIOMARKERS OF PATHOLOGY IN MILD COGNITIVE IMPAIRMENT

As described above, MCI is considered to be a prodromal stage of AD, with an annualized conversion rate of 10% to 15% (MCI to AD) (Petersen et al. 1999). Evaluating the extent of AD pathology using biomarkers in patients with MCI may provide clues regarding the biological mechanisms underlying progression to AD as well as assist with early identification of patients at greatest risk to progress to an AD diagnosis, which will be important for treatment development and clinical trials.

Using structural MRI methods, MCI patients have been shown to have intermediate atrophy between AD patients and HCs, supporting this as an intermediate stage between healthy aging and AD (Du et al. 2001). MCI patients, particularly the more clinically mild patients, tend to have more focal reductions in volume and GM density than AD patients. Specifically, MCI patients show significant atrophy in the MTL, particularly in the EC and hippocampus, and focal cortical atrophy, particularly in the temporal, parietal, and frontal lobes (Chetelat et al. 2002, Hamalainen et al. 2007, Whitwell et al. 2008) (Figure 2*a*). Global morphometric techniques have also been shown to effectively monitor expansion of atrophy as MCI patients progress clinically over the course of three years, showing the expected pattern of atrophy expansion, starting in the MTL and progressing to encompass more extensive cortical regions (Whitwell et al. 2007). Longitudinal studies have shown greater atrophy rates in patients with MCI, including hippocampal annual atrophy rates of approximately 3% (for meta-analysis, see Barnes et al. 2009) and greater rates of cortical atrophy and ventricular expansion than HCs (Jack et al. 2004). Changes in global and local tissue morphometry have also been shown to correctly classify MCI patients and HCs with an overall accuracy of 87% (Trivedi et al. 2006), while measures of regions of interest have been shown to classify MCI patients and HCs with an overall accuracy of between 75% and 85% (Du et al. 2001, Killiany et al. 2002).

MRI measures of volume, morphometry, and rates of brain atrophy have also shown promise in predicting the course of AD progression. In fact, numerous studies have demonstrated significantly reduced hippocampal and EC volume, as well as reduced cortical thickness in the medial and lateral temporal cortex, parietal lobe, and frontal lobes, in patients destined to convert from MCI to probable AD (MCI-converters), up to two years prior to clinical conversion, relative to MCI patients that remain at a stable MCI diagnosis (MCI-stable) (deToledo-Morrell et al. 2004, Devanand et al. 2008, Jack et al. 1999, Risacher et al. 2009). Techniques assessing global and local tissue morphometry have also shown significantly reduced GM density and volume in MCI-converters relative to MCI-stables (Hamalainen et al. 2007, Whitwell et al. 2008). In a study by our group (Risacher et al. 2009) using data from ADNI, we found significantly reduced hippocampal and amygdalar volume, reduced EC thickness, and reduced lateral temporal and parietal lobe cortical thicknesses in patients who converted from MCI to AD within 12 months relative to those who remained stable. Rates of brain atrophy, including annualized whole-brain,

NIH-PA Author Manuscript

NIH-PA Author Manuscript

NIH-PA Author Manuscript

hippocampal, and EC volume decline, as well as rates of ventricular enlargement, are also accelerated in patients who are rapidly converting from MCI to AD (Jack et al. 2004, Risacher et al. 2010). In addition to detecting differences in MCI-converters, baseline and rate of hippocampal atrophy have been used to predict MCI to probable AD conversion. Using Cox proportional hazard models, reduced baseline hippocampal volume and increased annual hippocampal atrophy rates accurately predicted MCI to probable AD conversion (Devanand et al. 2008, Jack et al. 1999). Baseline hippocampal volume also correctly classified MCI-converters and MCI-stables with an overall accuracy of between 75% and 90% (deToledo-Morrell et al. 2004, Killiany et al. 2002). Thus, in MCI a set of established anatomic features measured using structural MRI methods serves as both an antecedent biomarker for clinical conversion/progression and a useful marker to monitor disease course.

In studies utilizing DTI techniques, MCI patients show intermediate reductions in FA relative to AD patients and HCs, with significantly smaller FA values than HCs in the corpus callo-sum, temporal WM, hippocampus, cingulum bundles, superior longitudinal fasiculus, thalamus, parahippocampal subgyral WM, posterior uncinate fasiculus, and posterior cingulate (Rose et al. 2006, Zhang et al. 2007). Increased ADC and MD values are also seen in patients with MCI relative to HCs (Fellgiebel et al. 2004, Ibrahim et al. 2009). Measures of FA and MD also have been shown to predict future conversion from MCI to probable AD (Kantarci et al. 2005).

MRS studies have shown reductions in NAA in patients with MCI relative to HC (Chantal et al. 2004, Kantarci et al. 2000), although NAA values tend to be intermediate between those seen in AD and HC participants. Additionally, NAA/Cr has been shown to correlate with cognitive performance (Chantal et al. 2002) and to predict future MCI to AD conversion (Metastasio et al. 2006).

Changes in cerebral blood flow, as measured using arterial spin-labeling perfusion, have also been reported in patients with MCI relative to HCs. Specifically, MCI patients show decreased brain perfusion, particularly in the medial and inferior parietal lobes (Alsop et al. 2010). The findings of studies utilizing DTI, MRS, and perfusion imaging suggest that these advanced MRI techniques may be useful biomarkers for detecting early pathophysiological changes in prodromal stages of AD, although additional studies are needed.

Task-related and resting-state fMRI techniques have been utilized in studies of patients with MCI and suggest alterations in brain function. Conflicting results in patients with MCI have been shown in studies measuring brain function during memory tasks. Some studies have shown decreased activation relative to HCs during episodic memory encoding (Celone et al. 2006, Johnson et al. 2006a, Trivedi et al. 2008) and recall tasks (Celone et al. 2006, Johnson et al. 2006a, Trivedi et al. 2008), whereas others show increased activation (Celone et al. 2006, Dickerson et al. 2005, Trivedi et al. 2008), particularly in regions of the hippocampus, parahippocampal gyrus, MTL, posterior cingulate, precuneus, parietal lobe, and frontal lobe (Figure 3*a*). Interestingly, the level of disease severity of the targeted patient populations may explain some of these conflicting findings. The studies to date have suggested that MCI patients who are minimally impaired tend to show increased activation, which may represent a compensatory mechanism engaged to assist with successful completion of the task. However, more impaired patients with MCI, especially those with advanced atrophy that can no longer support increased neuronal activity, show decreased activation during tasks. A few studies have provided evidence supporting this theory, showing that the extent of hippocampal activation during a memory task was related to clinical severity within MCI patients (Celone et al. 2006, Dickerson et al. 2005, O'Brien et al. 2010). Increased activation in the hippocampal region during memory tasks at baseline may also predict a faster rate of future clinical decline of MCI patients, coinciding with a greater longitudinal decline in

NIH-PA Author Manuscript

NIH-PA Author Manuscript

NIH-PA Author Manuscript

hippocampal activation during memory tasks (Miller et al. 2008, O'Brien et al. 2010). Studies evaluating brain connectivity during cognitive tasks and at rest have demonstrated altered connectivity in patients with MCI relative to HCs. Generally, patients with MCI show reduced whole-brain functional connectivity during memory tasks, as well as reduced connectivity of the hippocampus (Celone et al. 2006, Zhou et al. 2008). Similar to the activation fMRI studies, mildly impaired MCI patients actually show increased functional connectivity between the memory network and the resting-state network, suggesting compensatory changes (Bai et al. 2009, Celone et al. 2006, Grady et al. 2003), whereas more impaired MCI patients show decreased or absent connectivity between these regions (Celone et al. 2006). Studies using resting-state connectivity techniques, which are designed to evaluate intrinsic brain networks that are not necessarily task related, have shown significant alterations in patients with MCI. In particular, patients with MCI show alterations in DMN connectivity and the connectivity of MTL regions. Similar to the task-related activity and functional connectivity, mildly impaired MCI patients show greater deactivation upon task initiation and increased connectivity of the DMN, whereas more advanced MCI patients show impaired deactivation of the DMN upon task initiation and decreased DMN connectivity (Celone et al. 2006, Grady et al. 2003) (Figure 3*b*). Furthermore, more impaired MCI patients show impaired connectivity between the hippocampus (episodic memory network) and the DMN at rest (Buckner et al. 2005, Greicius et al. 2004).

FDG PET studies of patients with MCI have shown significant differences in resting cerebral glucose metabolism between patients and healthy elderly. Specifically, patients with MCI show reduced glucose metabolism in the temporoparietal cortex, posterior cingulate, parietal lobe, temporal lobe, and in the MTL, including the hippocampus (Buckner et al. 2005, Langbaum et al. 2009, Mosconi 2005) (Figure 2*b*). Longitudinal studies in MCI patients also show a significantly greater rate of annual decline in metabolism in the temporal, parietal, and frontal lobes, as well as the posterior cingulate and precuneus relative to HCs (Alexander et al. 2002). Temporoparietal and posterior cingulate hypometabolism has also been shown to predict cognitive decline and progression from MCI to probable AD, with a predictive accuracy for future MCI to AD conversion of between 75% and 95% (Herholz et al. 1999, Mosconi et al. 2004).

Using amyloid imaging tracers (most commonly [^{11}C]PiB), studies in patients with MCI have shown significantly increased tracer uptake and binding relative to HCs, particularly in regions of the medial and lateral parietal lobe, anterior and posterior cingulate cortex, medial and lateral frontal lobe, and the lateral temporal lobe (Jack et al. 2008, 2009) (Figure 2*c*). In fact, nearly two-thirds of patients with MCI show significant amyloid accumulation as measured using [^{11}C]PiB (Johnson et al. 2012). In addition, MCI patients with significant amyloid accumulation show a higher likelihood for future conversion to AD (Forsberg et al. 2008). Longitudinal studies in patients with MCI demonstrate that once a participant shows a positive [^{11}C]PiB signal, there is very little annual increase in amyloid deposition (Jack et al. 2009). However, in patients who do not show amyloid deposition at baseline, as measured by [^{11}C]PiB, some additional amyloid accumulation may be possible.

Very few studies evaluating neurotransmitter system activity have been completed in patients with MCI. Studies evaluating ACh neurotransmission (i.e., targeting AChE activity) and ACh receptor levels have demonstrated significant decreases in ACh levels and receptor density in MCI patients, particularly in the temporal lobe (Herholz et al. 2005, Sabri et al. 2008). In addition, these changes in ACh synaptic density have been shown to predict conversion from MCI to AD (Herholz et al. 2005). PET techniques targeting the ACh system have also shown promise in monitoring treatment response to AChE inhibitors in patients with MCI (Bohnen et al. 2005). Studies of serotonin neurotransmission have also showed reduced receptor density in patients with MCI (Hasselbalch et al. 2008). PET studies

NIH-PA Author Manuscript

NIH-PA Author Manuscript

NIH-PA Author Manuscript

of neuroinflammation have also been performed in MCI patients. Although findings have been mixed, one study showed that patients with MCI have elevated tracer binding in the frontal lobe and cingulate (Okello et al. 2009). Future studies elucidating the role of neurotransmission and neuroinflammation in MCI patients will help to establish the utility of these techniques as biomarkers.

Patients with MCI show altered CSF protein levels, particularly of $A\beta_{40}$, $A\beta_{42}$, total tau, and p-tau. Specifically, MCI patients show decreased levels of $A\beta_{40}$ and $A\beta_{42}$ and increased levels of total tau and p-tau relative to HCs (Shaw et al. 2007, 2009; Trojanowski et al. 2010). In addition, an increased level of homocysteine has been observed in patients with MCI (Trojanowski et al. 2010). Decreased $A\beta_{42}$ levels and increased tau levels have also been shown to be predictive of future conversion from MCI to AD (Shaw et al. 2007, 2009; Trojanowski et al. 2010).

In summary, patients with MCI show significant AD-related neuropathology, including notable brain atrophy, alterations in brain function, reduced glucose metabolism, amyloid accumulation, altered neurotransmission and neuroinflammation, and abnormal levels of Aβ and tau in the CSF. These biomarkers often indicate that more impaired patients have greater AD-like pathology and that MCI patients with more significant brain atrophy and functional changes, as well as altered glucose metabolism and amyloid deposition, are at greater risk for future progression to AD. Future studies of imaging and nonimaging biomarkers in patients with MCI will allow for additional understanding of the pathological processes occurring as the disease progresses toward AD. Biomarkers are now often employed in clinical trials of potential therapeutics for sample enrichment and/or measuring treatment efficacy. Use of biomarkers in clinical settings to detect and predict outcomes in patients with MCI may soon be warranted as key tools for personalization of diagnostics and therapeutics.

BIOMARKERS OF PATHOLOGY IN PRECLINICAL PATIENTS

Many investigators believe that the ideal treatment for AD will be preventative, potentially as an intervention prior to the emergence of clinical symptoms (i.e., before MCI). Therefore, a major goal is identification of early AD-like pathology that may indicate a higher likelihood for future development of AD symptoms in individuals with no significant clinical symptoms. There are a number of ways to potentially identify older adults at increased risk for the development of AD. We focus here on three groups of participants that have been identified as at greater risk for future AD: (*a*) cognitively healthy older adults who show abnormal amyloid accumulation using either PET or CSF measures; (*b*) participants at risk for AD due to genetic background and/or a family history of AD; and, (*c*) older adults with cognitive complaints, particularly in the memory domain.

Initial studies with amyloid imaging tracers showed that approximately 25% to 30% of older adults with normal cognition are amyloid positive (Johnson et al. 2012, Mintun et al. 2006, Villemagne et al. 2008). As might be expected, longitudinal follow-up studies of these patients suggest that they show subtle changes in cognition (Mormino et al. 2009, Pike et al. 2011) and are significantly more likely to progress to MCI or AD (Mintun et al. 2006, Villemagne et al. 2008). As such, these patients may represent a biomarker-determined preclinical AD sample, which may be ideal for testing anti-amyloid and related interventions.

In order to better characterize these patients, studies to evaluate brain atrophy, brain function, and glucose metabolism have been completed utilizing structural and functional MRI and FDG PET techniques, respectively. Amyloid-positive HCs show significant

NIH-PA Author Manuscript NIH-PA Author Manuscript NIH-PA Author Manuscript

baseline brain atrophy and a faster rate of atrophy relative to amyloid-negative HCs, particularly in regions of the MTL, lateral temporal lobe, anterior cingulate, and medial and lateral parietal and frontal lobes (Bourgeat et al. 2010, Chetelat et al. 2010, Jack et al. 2008). Studies with functional MRI have also demonstrated changes in task-related activation and deactivation, as well as altered resting-state connectivity in the DMN, in amyloid-positive HCs relative to amyloid-negative HCs (Rami et al. 2012, Sperling et al. 2009). However, in an initial study with FDG PET techniques, no significant alterations in glucose metabolism were seen in amyloid-positive HCs relative to amyloid-negative HCs (Cohen et al. 2009). In sum, these findings in amyloid-positive HCs suggest that these participants may be in a preclinical stage of AD, with a higher likelihood for future cognitive decline.

Although older adults with amyloid pathology are considered to be at higher risk for future cognitive decline, the reason for cognitively normal performance in participants with high amyloid levels is currently unknown. Researchers have theorized that these participants show cognitive resilience to the presence of notable pathology, which is referred to as cognitive reserve (Stern 2002). Cognitive reserve is often thought to be related to educational and occupational attainment and has been shown to independently modulate the relationship between amyloid accumulation and cognition in healthy older adults (Rentz et al. 2010) as well as in patients with MCI and AD (Kemppainen et al. 2008, Vemuri et al. 2011). Specifically, patients who had higher levels of cognitive reserve, as measured either by educational attainment or performance on a measure of baseline general verbal ability (e.g., the American National Adult Reading Test), had better cognition than those with lower cognitive reserve, even at equivalently high levels of amyloid accumulation. Furthermore, cognitive reserve was shown to be associated with cognition independent of known imaging and CSF biomarkers (Vemuri et al. 2011), suggesting that cognitive reserve is an important factor in determining cognition, even in the presence of significant AD neuropathology.

Individuals who are at risk for the development of AD can also be identified by genetic background and/or a family history of AD. The most commonly associated genetic variant with late-onset AD is the apolipoprotein E epsilon 4 (*APOE* ε4) allele, which confers a dose-dependent increased likelihood of developing AD (Corder et al. 1993). Therefore, healthy older adults who are positive for the *APOE* ε4 allele are at higher risk for progressing to MCI and AD. A number of studies utilizing imaging and nonimaging biomarkers in these patients have been conducted. Structural MRI studies have shown that HC participants who are positive for *APOE* ε4 show more baseline atrophy and faster brain atrophy rates, particularly in the MTL, than participants who are negative for *APOE* ε4 (Donix et al. 2010b, Honea et al. 2009, Risacher et al. 2010, Wishart et al. 2006a). Family history of AD has also been shown to be associated with MTL atrophy in HCs (Donix et al. 2010a). Additionally, hippocampal activation during episodic memory encoding and recall tasks, working memory, and connectivity in multiple brain networks, including the DMN, is altered in cognitively normal older controls who are *APOE* ε4 positive and/or have a positive family history for dementia (Bookheimer et al. 2000, Johnson et al. 2006b, Trachtenberg et al. 2012, Wishart et al. 2006b). Alterations in resting cerebral glucose metabolism in an AD-like pattern have also been shown in participants who are positive for *APOE* ε4 and/or those with a positive family history of dementia (de Leon et al. 2001, Small et al. 2000). Finally, HC participants who are positive for *APOE* ε4 show greater amyloid accumulation, measured using PET and CSF measures, as well as increased CSF tau and p-tau levels (Honea et al. 2012, Mosconi et al. 2010, Reiman et al. 2009, Rowe et al. 2010).

Finally, cognitively healthy older adults who have significant cognitive complaints, subjective cognitive impairment, or subjective memory impairment may also represent a population in a preclinical stage of AD, as they show a higher likelihood for clinical progression and cognitive decline (Duara et al. 2011, Jessen et al. 2010, Reisberg et al.

NIH-PA Author Manuscript

NIH-PA Author Manuscript

NIH-PA Author Manuscript

2010). Studies with imaging and nonimaging biomarkers have been completed in older adults with cognitive complaints. Specifically, older adults with cognitive complaints show significant atrophy, particularly in the MTL, relative to HCs without complaints (Saykin et al. 2006, Scheef et al. 2012, Striepens et al. 2010). Furthermore, DTI measures show significant WM changes in older adults with cognitive complaints relative to those without complaints (Wang et al. 2012). Studies of glucose metabolism with FDG have demonstrated reduced cerebral metabolism in older adults with cognitive complaints relative to those without complaints, particularly in the presence of an *APOE* ε4 allele (Mosconi et al. 2008). CSF measures of Aβ and tau are also altered in older adults with cognitive complaints, but only in the presence of an *APOE* ε4 allele (Mosconi et al. 2008). Therefore, the findings in older adults with cognitive complaints suggest that these participants may represent a preclinical stage of AD that could be targeted for interventional therapies. One limitation in this area is the lack of well-standardized definitions of cognitive complaints, subjective cognitive impairment, subjective memory impairment, and related classifications (Abdulrab & Heun 2008). Further progress in this area would be facilitated by standardizing criteria and assessment approaches for pre-MCI stages of AD.

BIOMARKERS OF PATHOLOGY IN FAMILIAL ALZHEIMER'S DISEASE

Although the majority of AD cases represent late-onset or sporadic AD, nearly 5% of AD cases are caused by dominantly inherited genetic mutations, usually in one of three genes: amyloid precursor protein (*APP*), presenilin 1 (*PS1*), or presenilin 2 (*PS2*). Often featuring symptom onset at an earlier age seen than in sporadic AD patients (i.e., before age 65), these cases are referred to as familial AD or early-onset AD. Although these diseases may show symptomology and pathology that are somewhat different from late-onset AD, the major AD hallmarks (i.e., amyloid plaques and NFTs) are present. Therefore, these patients may represent a useful sample for studying early AD-like pathologic changes with biomarkers, particularly because the age of symptom onset tends to be consistent across generations. Therefore, using an estimated age of symptom onset, changes in neuropathology and cognition can be assessed using biomarkers decades before onset of disease. Studies with familial AD patients have shown greater brain atrophy, faster longitudinal atrophy rates, WM changes measured using DTI, reduced CSF Aβ levels and increased CSF tau, p-tau, and isoprostane levels, increased plasma Aβ levels, and subtle declines in cognition in presymptomatic mutation carriers (MCs) relative to noncarriers (NCs) (Bateman et al. 2012; Fox et al. 1996; Gregory et al. 2006; Ringman et al. 2007, 2008). Bateman et al. (2012) compared multiple biomarkers in the same sample (the Dominantly Inherited Alzheimer Network consortium study) and evaluated changes in target biomarkers in five-year intervals from 25 years before the estimated age of symptom onset to 10 years after onset of symptoms to assess the relative sensitivity and ordering of biomarkers at various stages of disease (Bateman et al. 2012) (Figure 4). This study found that amyloid accumulation assessed by PET was the earliest difference that could be detected between presymptomatic MCs and NCs, with increased amyloid in MCs approximately 15 years before the estimated symptom onset (Figure 4). Pathologic changes in other imaging (MRI, FDG PET), CSF (Aβ, tau), and plasma (Aβ) biomarkers were also detectable in MCs relative to NCs in the presymptomatic phase, typically between 10 and 15 years before the estimated symptom onset. Cognition began to decline approximately five years before the estimated symptom onset, with memory function showing the earliest changes. Overall, the use of biomarkers in the study of familial AD has shown neuropathological changes similar to those seen in late-onset AD, in both presymptomatic and symptomatic familial AD patients. Studies in these patients may give further information about the role of biomarkers in studies of late-onset AD as well as provide sensitive measures for detecting disease-related changes and monitoring disease progression in patients with familial AD.

NIH-PA Author Manuscript

NIH-PA Author Manuscript

NIH-PA Author Manuscript

THE EMERGENT BIOMARKER-DRIVEN THEORETICAL FRAMEWORK OF ALZHEIMER'S DISEASE

In 2010, Jack and colleagues presented a theoretical framework for imaging and nonimaging biomarkers of AD (Jack et al. 2010b) (Figure 5). The proposed theory maintains that amyloid accumulation is the earliest marker of AD-like pathological changes, and thus amyloid assessment techniques (i.e., PET, CSF) will show the earliest changes in patients that ultimately progress to AD. After the amyloid accumulation, measures of neuronal functioning (i.e., FDG PET, CSF tau, and fMRI) would next show alterations, followed by measures of regional and cortical GM and WM atrophy. Finally, changes in cognition and clinical dementia status could be observed. These biomarkers are presented across the clinical disease spectrum, with changes in amyloid, neuronal function, and brain atrophy potentially detectable before any clinical symptoms. Furthermore, the change in each biomarker over time and as the disease progresses represents a sigmoidal function, which ultimately reaches a plateau at which no additional change in the measure can be detected. Although this theoretical framework is still preliminary and needs further testing, a few studies to date have confirmed the ordering of biomarkers and the sigmoidal-like shape of the measures over time (Caroli & Frisoni 2010, Jack et al. 2012, Weiner et al. 2011). Interestingly, these curves may be shifted or altered by other factors, such as lifestyle and health factors, genetic variations (e.g., *APOE*), and cognitive reserve (Jack et al. 2010b) (Figure 6). Future studies to further investigate this theory, as well as the integration of novel biomarkers (i.e., plasma protein biomarkers and advanced imaging techniques) and other factors known to affect AD prevalence (i.e., genetic variants, family history, and lifestyle factors), will help to clarify further the pathologic and biological processes associated with the progression toward AD.

IMAGING GENETICS

Genetic variation plays a significant role in the development of AD and MCI, as indicated by previous studies that have suggested a high heritability for AD (Gatz et al. 2006). Although *APOE* is the most significant genetic variant associated with AD (Bertram & Tanzi 2008, Corder et al. 1993), it does not account for all of the AD heritability. Genome-wide association studies (GWAS) using high-density genotyping technology have led to the identification of additional replicated loci (Hollingworth et al. 2011, Naj et al. 2011), although all have effect sizes that are much smaller than those of *APOE*. The primary outcome measure in these large studies was the presence or absence of an AD diagnosis. Many researchers believe that refinement of disease-associated phenotypes for GWAS will lead to the identification of additional important loci. A novel direction of AD research has been the use of imaging and nonimaging biomarkers as endophenotypes in studies designed to identify novel genetic variants associated with AD pathology. Studies have suggested that using continuous and biologically specific phenotypes may lead to increased power in genetic associations (Potkin et al. 2009b). To date, a few studies have been published in this area and have identified a number of genetic variants associated with MRI atrophy measures (Furney et al. 2011; Potkin et al. 2009a; Shen et al. 2010; Stein et al. 2010, 2012), CSF measures (Cruchaga et al. 2010, Han et al. 2010, Kim et al. 2011), and other biomarkers (Saykin et al. 2010, Swaminathan et al. 2011, Xu et al. 2010). Future imaging genetic studies will likely help identify important genetic factors underlying AD, which is a polygenic or complex disease. This should contribute to improved early identification of people at risk for developing AD and help to elucidate further the biological mechanisms associated with AD, which in turn may identify prominent targets for therapeutic development.

NIH-PA Author Manuscript NIH-PA Author Manuscript NIH-PA Author Manuscript

CONCLUSION

Imaging and nonimaging biomarkers of AD have demonstrated sensitivity to detecting AD pathology even in prodromal and preclinical stages of disease. Patients with clinical AD and those in prodromal stages show extensive amyloid accumulation (measured using PET and CSF methods), alterations in brain functioning both during tasks and at rest (measured using fMRI techniques), cortical and subcortical GM and WM atrophy (measured using structural MRI and DTI), and altered cerebral perfusion (measured using dynamic susceptibility contrast-enhanced MRI or arterial spin labeling) and glucose utilization (measured using FDG PET), as well as other changes in brain neurochemistry (measured using MRS and CSF protein levels), neuroinflammation (measured using PET), and neurotransmission (measured using PET). These tools provide researchers the ability to evaluate AD pathology in vivo and thus may assist with clinical diagnosis and optimization of trials of therapeutic interventions. Biomarkers for early detection of pathology, prediction of future clinical progression, and monitoring of treatment response will become increasingly important in the quest to ultimately prevent this devastating disease.

Acknowledgments

Data collection and sharing for the Alzheimer's Disease Neuroimaging Initiative (ADNI) was funded by the National Institutes of Health (NIH) Grant U01 AG024904 (PI: M.W. Weiner). ADNI is also funded by the National Institute on Aging (NIA), the National Institute of Biomedical Imaging and Bioengineering (NIBIB), and through generous contributions from Abbott, Alzheimer's Association, Alzheimer's Drug Discovery Foundation, Amorfix Life Sciences Ltd., AstraZeneca, Bayer HealthCare, BioClinica Inc., Biogen Idec Inc., Bristol-Myers Squibb Company, Eisai Inc., Elan Pharmaceuticals Inc., Eli Lilly and Company, F. Hoffmann-La Roche Ltd. and its affiliated company Genentech Inc., GE HealthCare, Innogenetics N.V., IXICO Ltd., Janssen Alzheimer Immunotherapy Research & Development LLC, Johnson & Johnson Pharmaceutical Research & Development LLC, Medpace Inc., Merck & Co. Inc., Meso Scale Diagnostics LLC, Novartis Pharmaceuticals Corporation, Pfizer Inc., Servier, Synarc Inc., and Takeda Pharmaceutical Company, with participation from the U.S. FDA. The Canadian Institutes of Health Research is providing funds to support ADNI clinical sites in Canada. Private sector contributions are facilitated by the Foundation for the National Institutes of Health (http://www.fnih.org). The grantee organization is the Northern California Institute for Research and Education, and the study is coordinated by the Alzheimer's Disease Cooperative Study at the University of California, San Diego. ADNI data are disseminated by the Laboratory for Neuro Imaging at the University of California, Los Angeles. ADNI is also supported by NIH grants P30 AG010129, K01 AG030514, and the Dana Foundation. Additional support for this manuscript was provided by the following sources: NIA R01 AG19771 (to A.J.S.), P30 AG10133-18S1 (to Dr. Bernardino Ghetti and A.J.S.), and an Indiana CTSI Predoctoral Fellowship (TL1 RR025759 to S.L.R.).

LITERATURE CITED

Abdulrab K, Heun R. Subjective memory impairment. A review of its definitions indicates the need for a comprehensive set of standardised and validated criteria. Eur. Psychiatry. 2008; 23:321–30. [PubMed: 18434102]

Aisen PS, Petersen RC, Donohue MC, Gamst A, Raman R, et al. Clinical core of the Alzheimer's Disease Neuroimaging Initiative: progress and plans. Alzheimers Dement. 2010; 6:239–46. [PubMed: 20451872]

Albert MS, DeKosky ST, Dickson D, Dubois B, Feldman HH, et al. The diagnosis of mild cognitive impairment due to Alzheimer's disease: recommendations from the National Institute on Aging—Alzheimer's Association workgroups on diagnostic guidelines for Alzheimer's disease. Alzheimers Dement. 2011; 7:270–79. [PubMed: 21514249]

Alexander GE, Chen K, Pietrini P, Rapoport SI, Reiman EM. Longitudinal PET evaluation of cerebral metabolic decline in dementia: a potential outcome measure in Alzheimer's disease treatment studies. Am. J. Psychiatry. 2002; 159:738–45. [PubMed: 11986126]

Alsop DC, Dai W, Grossman M, Detre JA. Arterial spin labeling blood flow MRI: its role in the early characterization of Alzheimer's disease. J. Alzheimers Dis. 2010; 20:871–80. [PubMed: 20413865]

Ashburner J, Friston KJ. Voxel-based morphometry—the methods. Neuroimage. 2000; 11:805–21. [PubMed: 10860804]

NIH-PA Author Manuscript NIH-PA Author Manuscript NIH-PA Author Manuscript

Alzheimer's Assoc.. 2012 Alzheimer's Disease Facts and Figures. Alzheimer's Assoc.; Chicago, IL: 2012. http://www.alz.org/documents_ custom/2012_facts_figures_fact_sheet.pdf

Bai F, Zhang Z, Watson DR, Yu H, Shi Y, et al. Abnormal functional connectivity of hippocampus during episodic memory retrieval processing network in amnestic mild cognitive impairment. Biol. Psychiatry. 2009; 65:951–58. [PubMed: 19028382]

Banati RB, Newcombe J, Gunn RN, Cagnin A, Turkheimer F, et al. The peripheral benzodiazepine binding site in the brain in multiple sclerosis: quantitative in vivo imaging of microglia as a measure of disease activity. Brain. 2000; 123(Pt. 11):2321–37. [PubMed: 11050032]

Barnes J, Bartlett JW, van de Pol LA, Loy CT, Scahill RI, et al. A meta-analysis of hippocampal atrophy rates in Alzheimer's disease. Neurobiol. Aging. 2009; 30:1711–23. [PubMed: 18346820]

Bateman RJ, Xiong C, Benzinger TL, Fagan AM, Goate A, et al. Clinical and biomarker changes in dominantly inherited Alzheimer's disease. N. Engl. J. Med. 2012; 367:795–804. [PubMed: 22784036]

Beckett LA, Harvey DJ, Gamst A, Donohue M, Kornak J, et al. The Alzheimer's Disease Neuroimaging Initiative: annual change in biomarkers and clinical outcomes. Alzheimers Dement. 2010; 6:257–64. [PubMed: 20451874]

Bennett DA, Schneider JA, Wilson RS, Bienias JL, Arnold SE. Neurofibrillary tangles mediate the association of amyloid load with clinical Alzheimer disease and level of cognitive function. Arch. Neurol. 2004; 61:378–84. [PubMed: 15023815]

Bertram L, Tanzi RE. Thirty years of Alzheimer's disease genetics: the implications of systematic meta-analyses. Nat. Rev. Neurosci. 2008; 9:768–78. [PubMed: 18802446]

Bohnen NI, Kaufer DI, Hendrickson R, Ivanco LS, Lopresti BJ, et al. Degree of inhibition of cortical acetylcholinesterase activity and cognitive effects by donepezil treatment in Alzheimer's disease. J. Neurol. Neurosurg. Psychiatry. 2005; 76:315–19. [PubMed: 15716518]

Bookheimer SY, Strojwas MH, Cohen MS, Saunders AM, Pericak-Vance MA, et al. Patterns of brain activation in people at risk for Alzheimer's disease. N. Engl. J. Med. 2000; 343:450–56. [PubMed: 10944562]

Bourgeat P, Chetelat G, Villemagne VL, Fripp J, Raniga P, et al. Beta-amyloid burden in the temporal neocortex is related to hippocampal atrophy in elderly subjects without dementia. Neurology. 2010; 74:121–27. [PubMed: 20065247]

Braak H, Braak E, Bohl J. Staging of Alzheimer-related cortical destruction. Eur. Neurol. 1993; 33:403–8. [PubMed: 8307060]

Buckner RL, Snyder AZ, Shannon BJ, LaRossa G, Sachs R, et al. Molecular, structural, and functional characterization of Alzheimer's disease: evidence for a relationship between default activity, amyloid, and memory. J. Neurosci. 2005; 25:7709–17. [PubMed: 16120771]

Caroli A, Frisoni GB. The dynamics of Alzheimer's disease biomarkers in the Alzheimer's Disease Neuroimaging Initiative cohort. Neurobiol. Aging. 2010; 31:1263–74. [PubMed: 20538373]

Celone KA, Calhoun VD, Dickerson BC, Atri A, Chua EF, et al. Alterations in memory networks in mild cognitive impairment and Alzheimer's disease: an independent component analysis. J. Neurosci. 2006; 26:10222–31. [PubMed: 17021177]

Chaki S, Funakoshi T, Yoshikawa R, Okuyama S, Okubo T, et al. Binding characteristics of [3H]DAA1106, a novel and selective ligand for peripheral benzodiazepine receptors. Eur. J. Pharmacol. 1999; 371:197–204. [PubMed: 10357257]

Chantal S, Braun CM, Bouchard RW, Labelle M, Boulanger Y. Similar 1H magnetic resonance spectroscopic metabolic pattern in the medial temporal lobes of patients with mild cognitive impairment and Alzheimer disease. Brain Res. 2004; 1003:26–35. [PubMed: 15019560]

Chantal S, Labelle M, Bouchard RW, Braun CM, Boulanger Y. Correlation of regional proton magnetic resonance spectroscopic metabolic changes with cognitive deficits in mild Alzheimer disease. Arch. Neurol. 2002; 59:955–62. [PubMed: 12056931]

Chetelat G, Desgranges B, De La Sayette V, Viader F, Eustache F, Baron JC. Mapping gray matter loss with voxel-based morphometry in mild cognitive impairment. Neuroreport. 2002; 13:1939–43. [PubMed: 12395096]

NIH-PA Author Manuscript
NIH-PA Author Manuscript
NIH-PA Author Manuscript

Chetelat G, Villemagne VL, Bourgeat P, Pike KE, Jones G, et al. Relationship between atrophy and beta-amyloid deposition in Alzheimer disease. Ann. Neurol. 2010; 67:317–24. [PubMed: 20373343]

Cohen AD, Price JC, Weissfeld LA, James J, Rosario BL, et al. Basal cerebral metabolism may modulate the cognitive effects of Abeta in mild cognitive impairment: an example of brain reserve. J. Neurosci. 2009; 29:14770–78. [PubMed: 19940172]

Corder EH, Saunders AM, Strittmatter WJ, Schmechel DE, Gaskell PC, et al. Gene dose of apolipoprotein E type 4 allele and the risk of Alzheimer's disease in late onset families. Science. 1993; 261:921–23. [PubMed: 8346443]

Cruchaga C, Kauwe JS, Mayo K, Spiegel N, Bertelsen S, et al. SNPs associated with cerebrospinal fluid phospho-tau levels influence rate of decline in Alzheimer's disease. PLoS Genet. 2010; 6(9):e1001101. [PubMed: 20862329]

Dale A, Fischl B, Sereno M. Cortical surface-based analysis. I. Segmentation and surface reconstruction. Neuroimage. 1999; 9:179–94. [PubMed: 9931268]

de Leon MJ, Convit A, Wolf OT, Tarshish CY, DeSanti S, et al. Prediction of cognitive decline in normal elderly subjects with 2-[(18)F]fluoro-2-deoxy-D-glucose/positron-emission tomography (FDG/PET). Proc. Natl. Acad. Sci. USA. 2001; 98:10966–71. [PubMed: 11526211]

de Leon MJ, Mosconi L, Blennow K, DeSanti S, Zinkowski R, et al. Imaging and CSF studies in the preclinical diagnosis of Alzheimer's disease. Ann. N. Y. Acad. Sci. 2007; 1097:114–45. [PubMed: 17413016]

deToledo-Morrell L, Stoub TR, Bulgakova M, Wilson RS, Bennett DA, et al. MRI-derived entorhinal volume is a good predictor of conversion from MCI to AD. Neurobiol. Aging. 2004; 25:1197–203. [PubMed: 15312965]

Devanand DP, Liu X, Tabert MH, Pradhaban G, Cuasay K, et al. Combining early markers strongly predicts conversion from mild cognitive impairment to Alzheimer's disease. Biol. Psychiatry. 2008; 64:871–79. [PubMed: 18723162]

Dickerson BC, Salat DH, Greve DN, Chua EF, Rand-Giovannetti E, et al. Increased hippocampal activation in mild cognitive impairment compared to normal aging and AD. Neurology. 2005; 65:404–11. [PubMed: 16087905]

Dik MG, Jonker C, Comijs HC, Bouter LM, Twisk JW, et al. Memory complaints and APOE-epsilon4 accelerate cognitive decline in cognitively normal elderly. Neurology. 2001; 57:2217–22. [PubMed: 11756600]

Donix M, Burggren AC, Suthana NA, Siddarth P, Ekstrom AD, et al. Family history of Alzheimer's disease and hippocampal structure in healthy people. Am. J. Psychiatry. 2010a; 167:1399–406. [PubMed: 20686185]

Donix M, Burggren AC, Suthana NA, Siddarth P, Ekstrom AD, et al. Longitudinal changes in medial temporal cortical thickness in normal subjects with the APOE-4 polymorphism. Neuroimage. 2010b; 53:37–43. [PubMed: 20541611]

Du AT, Schuff N, Amend D, Laakso MP, Hsu YY, et al. Magnetic resonance imaging of the entorhinal cortex and hippocampus in mild cognitive impairment and Alzheimer's disease. J. Neurol. Neurosurg. Psychiatry. 2001; 71:441–47. [PubMed: 11561025]

Duara R, Loewenstein DA, Greig MT, Potter E, Barker W, et al. Pre-MCI and MCI: neuropsychological, clinical, and imaging features and progression rates. Am. J. Geriatr. Psychiatry. 2011; 19:951–60. [PubMed: 21422909]

Edison P, Archer HA, Gerhard A, Hinz R, Pavese N, et al. Microglia, amyloid, and cognition in Alzheimer's disease: an [11C](R)PK11195-PET and [11C]PIB-PET study. Neurobiol. Dis. 2008; 32:412–19. [PubMed: 18786637]

Fan Y, Resnick SM, Wu X, Davatzikos C. Structural and functional biomarkers of prodromal Alzheimer's disease: a high-dimensional pattern classification study. Neuroimage. 2008; 41:277–85. [PubMed: 18400519]

Fellgiebel A, Wille P, Muller MJ, Winterer G, Scheurich A, et al. Ultrastructural hippocampal and white matter alterations in mild cognitive impairment: a diffusion tensor imaging study. Dement. Geriatr. Cogn. Disord. 2004; 18:101–8. [PubMed: 15087585]

NIH-PA Author Manuscript

NIH-PA Author Manuscript

NIH-PA Author Manuscript

Fischl B, Dale AM. Measuring the thickness of the human cerebral cortex from magnetic resonance images. Proc. Natl. Acad. Sci. USA. 2000; 97:11050–55. [PubMed: 10984517]

Forsberg A, Engler H, Almkvist O, Blomquist G, Hagman G, et al. PET imaging of amyloid deposition in patients with mild cognitive impairment. Neurobiol. Aging. 2008; 29:1456–65. [PubMed: 17499392]

Fox NC, Warrington EK, Freeborough PA, Hartikainen P, Kennedy AM, et al. Presymptomatic hippocampal atrophy in Alzheimer's disease. A longitudinal MRI study. Brain. 1996; 119(Pt. 6): 2001–7. [PubMed: 9010004]

Furney SJ, Simmons A, Breen G, Pedroso I, Lunnon K, et al. Genome-wide association with MRI atrophy measures as a quantitative trait locus for Alzheimer's disease. Mol. Psychiatry. 2011; 16:1130–38. [PubMed: 21116278]

Gatz M, Reynolds CA, Fratiglioni L, Johansson B, Mortimer JA, et al. Role of genes and environments for explaining Alzheimer disease. Arch. Gen. Psychiatry. 2006; 63:168–74. [PubMed: 16461860]

Grady CL, McIntosh AR, Beig S, Keightley ML, Burian H, Black SE. Evidence from functional neuroimaging of a compensatory prefrontal network in Alzheimer's disease. J. Neurosci. 2003; 23:986–93. [PubMed: 12574428]

Gregory GC, Macdonald V, Schofield PR, Kril JJ, Halliday GM. Differences in regional brain atrophy in genetic forms of Alzheimer's disease. Neurobiol. Aging. 2006; 27:387–93. [PubMed: 15894410]

Greicius MD, Krasnow B, Reiss AL, Menon V. Functional connectivity in the resting brain: a network analysis of the default mode hypothesis. Proc. Natl. Acad. Sci. USA. 2003; 100:253–58. [PubMed: 12506194]

Greicius MD, Srivastava G, Reiss AL, Menon V. Default-mode network activity distinguishes Alzheimer's disease from healthy aging: evidence from functional MRI. Proc. Natl. Acad. Sci. USA. 2004; 101:4637–42. [PubMed: 15070770]

Haga S, Akai K, Ishii T. Demonstration of microglial cells in and around senile (neuritic) plaques in the Alzheimer brain. An immunohistochemical study using a novel monoclonal antibody. Acta Neuropathol. 1989; 77:569–75. [PubMed: 2750476]

Hamalainen A, Tervo S, Grau-Olivares M, Niskanen E, Pennanen C, et al. Voxel-based morphometry to detect brain atrophy in progressive mild cognitive impairment. Neuroimage. 2007; 37:1122–31. [PubMed: 17683950]

Han MR, Schellenberg GD, Wang LS. Genome-wide association reveals genetic effects on human Abeta42 and tau protein levels in cerebrospinal fluids: a case control study. BMC Neurol. 2010; 10:90. [PubMed: 20932310]

Harris GJ, Lewis RF, Satlin A, English CD, Scott TM, et al. Dynamic susceptibility contrast MR imaging of regional cerebral blood volume in Alzheimer disease: a promising alternative to nuclear medicine. Am. J. Neuroradiol. 1998; 19:1727–32. [PubMed: 9802497]

Hasselbalch SG, Madsen K, Svarer C, Pinborg LH, Holm S, et al. Reduced 5-HT2A receptor binding in patients with mild cognitive impairment. Neurobiol. Aging. 2008; 29:1830–38. [PubMed: 17544547]

Herholz K, Nordberg A, Salmon E, Perani D, Kessler J, et al. Impairment of neocortical metabolism predicts progression in Alzheimer's disease. Dement. Geriatr. Cogn. Disord. 1999; 10:494–504. [PubMed: 10559566]

Herholz K, Weisenbach S, Kalbe E, Diederich NJ, Heiss WD. Cerebral acetylcholine esterase activity in mild cognitive impairment. Neuroreport. 2005; 16:1431–34. [PubMed: 16110265]

Hollingworth P, Harold D, Sims R, Gerrish A, Lambert JC, et al. Common variants at ABCA7, MS4A6A/MS4A4E, EPHA1, CD33 and CD2AP are associated with Alzheimer's disease. Nat. Genet. 2011; 43:429–35. [PubMed: 21460840]

Honea RA, Vidoni E, Harsha A, Burns JM. Impact of APOE on the healthy aging brain: a voxel-based MRI and DTI study. J. Alzheimers Dis. 2009; 18:553–64. [PubMed: 19584447]

Honea RA, Vidoni ED, Swerdlow RH, Burns JM. Maternal family history is associated with Alzheimer's disease biomarkers. J. Alzheimers Dis. 2012; 31:659–68. [PubMed: 22669011]

NIH-PA Author Manuscript NIH-PA Author Manuscript NIH-PA Author Manuscript

Hua X, Leow AD, Parikshak N, Lee S, Chiang M-C, et al. Tensor-based morphometry as a neuroimaging biomarker for Alzheimer's disease: an MRI study of 676 AD, MCI, and normal subjects. Neuroimage. 2008; 43:458–69. [PubMed: 18691658]

Ibrahim I, Horacek J, Bartos A, Hajek M, Ripova D, et al. Combination of voxel based morphometry and diffusion tensor imaging in patients with Alzheimer's disease. Neuro Endocrinol. Lett. 2009; 30:39–45. [PubMed: 19300399]

Ikonomovic MD, Klunk WE, Abrahamson EE, Mathis CA, Price JC, et al. Post-mortem correlates of in vivo PiB-PET amyloid imaging in a typical case of Alzheimer's disease. Brain. 2008; 131:1630–45. [PubMed: 18339640]

Iqbal K, Grundke-Iqbal I. Alzheimer neurofibrillary degeneration: significance, etiopathogenesis, therapeutics and prevention. J. Cell. Mol. Med. 2008; 12:38–55. [PubMed: 18194444]

Jack CR Jr, Bernstein MA, Borowski BJ, Gunter JL, Fox NC, et al. Update on the magnetic resonance imaging core of the Alzheimer's Disease Neuroimaging Initiative. Alzheimers Dement. 2010a; 6:212–20. [PubMed: 20451869]

Jack CR Jr, Knopman DS, Jagust WJ, Shaw LM, Aisen PS, et al. Hypothetical model of dynamic biomarkers of the Alzheimer's pathological cascade. Lancet Neurol. 2010b; 9:119–28. [PubMed: 20083042]

Jack CR Jr, Lowe VJ, Senjem ML, Weigand SD, Kemp BJ, et al. 11C PiB and structural MRI provide complementary information in imaging of Alzheimer's disease and amnestic mild cognitive impairment. Brain. 2008; 131:665–80. [PubMed: 18263627]

Jack CR Jr, Lowe VJ, Weigand SD, Wiste HJ, Senjem ML, et al. Serial PIB and MRI in normal, mild cognitive impairment and Alzheimer's disease: implications for sequence of pathological events in Alzheimer's disease. Brain. 2009; 132:1355–65. [PubMed: 19339253]

Jack CR Jr, Petersen RC, Xu YC, O'Brien PC, Smith GE, et al. Prediction of AD with MRI-based hippocampal volume in mild cognitive impairment. Neurology. 1999; 52:1397–403. [PubMed: 10227624]

Jack CR Jr, Petersen RC, Xu YC, Waring SC, O'Brien PC, et al. Medial temporal atrophy on MRI in normal aging and very mild Alzheimer's disease. Neurology. 1997; 49:786–94. [PubMed: 9305341]

Jack CR Jr, Shiung MM, Gunter JL, O'Brien PC, Weigand SD, et al. Comparison of different MRI brain atrophy rate measures with clinical disease progression in AD. Neurology. 2004; 62:591–600. [PubMed: 14981176]

Jack CR Jr, Vemuri P, Wiste HJ, Weigand SD, Lesnick TG, et al. Shapes of the trajectories of 5 major biomarkers of Alzheimer disease. Arch. Neurol. 2012; 69:856–67. [PubMed: 22409939]

Jagust WJ, Bandy D, Chen K, Foster NL, Landau SM, et al. The Alzheimer's Disease Neuroimaging Initiative positron emission tomography core. Alzheimers Dement. 2010; 6:221–29. [PubMed: 20451870]

Jessen F, Wiese B, Bachmann C, Eifflaender-Gorfer S, Haller F, et al. Prediction of dementia by subjective memory impairment: effects of severity and temporal association with cognitive impairment. Arch. Gen. Psychiatry. 2010; 67:414–22. [PubMed: 20368517]

Johnson KA, Fox NC, Sperling RA, Klunk WE. Brain imaging in Alzheimer disease. Cold Spring Harb. Perspect. Med. 2012; 2:a006213. [PubMed: 22474610]

Johnson KA, Gregas M, Becker JA, Kinnecom C, Salat DH, et al. Imaging of amyloid burden and distribution in cerebral amyloid angiopathy. Ann. Neurol. 2007; 62:229–34. [PubMed: 17683091]

Johnson SC, Schmitz TW, Moritz CH, Meyerand ME, Rowley HA, et al. Activation of brain regions vulnerable to Alzheimer's disease: the effect of mild cognitive impairment. Neurobiol. Aging. 2006a; 27:1604–12. [PubMed: 16226349]

Johnson SC, Schmitz TW, Trivedi MA, Ries ML, Torgerson BM, et al. The influence of Alzheimer disease family history and apolipoprotein E epsilon4 on mesial temporal lobe activation. J. Neurosci. 2006b; 26:6069–76. [PubMed: 16738250]

Kantarci K, Jack CR Jr, Xu YC, Campeau NG, O'Brien PC, et al. Regional metabolic patterns in mild cognitive impairment and Alzheimer's disease: a 1H MRS study. Neurology. 2000; 55:210–17. [PubMed: 10908893]

NIH-PA Author Manuscript NIH-PA Author Manuscript NIH-PA Author Manuscript

Kantarci K, Petersen RC, Boeve BF, Knopman DS, Weigand SD, et al. DWI predicts future progression to Alzheimer disease in amnestic mild cognitive impairment. Neurology. 2005; 64:902–4. [PubMed: 15753434]

Kemppainen NM, Aalto S, Karrasch M, Nagren K, Savisto N, et al. Cognitive reserve hypothesis: Pittsburgh compound B and fluorodeoxyglucose positron emission tomography in relation to education in mild Alzheimer's disease. Ann. Neurol. 2008; 63:112–18. [PubMed: 18023012]

Killiany RJ, Hyman BT, Gomez-Isla T, Moss MB, Kikinis R, et al. MRI measures of entorhinal cortex versus hippocampus in preclinical AD. Neurology. 2002; 58:1188–96. [PubMed: 11971085]

Kim S, Swaminathan S, Shen L, Risacher SL, Nho K, et al. Genome-wide association study of CSF biomarkers Abeta1-42, t-tau, and p-tau181p in the ADNI cohort. Neurology. 2011; 76:69–79. [PubMed: 21123754]

Klunk WE, Engler H, Nordberg A, Wang Y, Blomqvist G, et al. Imaging brain amyloid in Alzheimer's disease with Pittsburgh compound-B. Ann. Neurol. 2004; 55:306–19. [PubMed: 14991808]

Klunk WE, Lopresti BJ, Ikonomovic MD, Lefterov IM, Koldamova RP, et al. Binding of the positron emission tomography tracer Pittsburgh compound-B reflects the amount of amyloid-beta in Alzheimer's disease brain but not in transgenic mouse brain. J. Neurosci. 2005; 25:10598–606. [PubMed: 16291932]

Klunk WE, Mathis CA, Price JC, Lopresti BJ, DeKosky ST. Two-year follow-up of amyloid deposition in patients with Alzheimer's disease. Brain. 2006; 129:2805–7. [PubMed: 17071918]

Korf ES, Wahlund LO, Visser PJ, Scheltens P. Medial temporal lobe atrophy on MRI predicts dementia in patients with mild cognitive impairment. Neurology. 2004; 63:94–100. [PubMed: 15249617]

Langbaum JB, Chen K, Lee W, Reschke C, Bandy D, et al. Categorical and correlational analyses of baseline fluorodeoxyglucose positron emission tomography images from the Alzheimer's Disease Neuroimaging Initiative (ADNI). Neuroimage. 2009; 45:1107–16. [PubMed: 19349228]

Logothetis NK, Pauls J, Augath M, Trinath T, Oeltermann A. Neurophysiological investigation of the basis of the fMRI signal. Nature. 2001; 412:150–57. [PubMed: 11449264]

Mawuenyega KG, Sigurdson W, Ovod V, Munsell L, Kasten T, et al. Decreased clearance of CNS beta-amyloid in Alzheimer's disease. Science. 2010; 330:1774. [PubMed: 21148344]

McKhann GM, Knopman DS, Chertkow H, Hyman BT, Jack CR Jr, et al. The diagnosis of dementia due to Alzheimer's disease: recommendations from the National Institute on Aging-Alzheimer's Association workgroups on diagnostic guidelines for Alzheimer's disease. Alzheimers Dement. 2011; 7:263–69. [PubMed: 21514250]

Metastasio A, Rinaldi P, Tarducci R, Mariani E, Feliziani FT, et al. Conversion of MCI to dementia: role of proton magnetic resonance spectroscopy. Neurobiol. Aging. 2006; 27:926–32. [PubMed: 15936850]

Miller SL, Fenstermacher E, Bates J, Blacker D, Sperling RA, Dickerson BC. Hippocampal activation in adults with mild cognitive impairment predicts subsequent cognitive decline. J. Neurol. Neurosurg. Psychiatry. 2008; 79:630–35. [PubMed: 17846109]

Mintun MA, Larossa GN, Sheline YI, Dence CS, Lee SY, et al. [11C]PIB in a nondemented population: potential antecedent marker of Alzheimer disease. Neurology. 2006; 67:446–52. [PubMed: 16894106]

Mormino EC, Kluth JT, Madison CM, Rabinovici GD, Baker SL, et al. Episodic memory loss is related to hippocampal-mediated beta-amyloid deposition in elderly subjects. Brain. 2009; 132:1310–23. [PubMed: 19042931]

Mosconi L. Brain glucose metabolism in the early and specific diagnosis of Alzheimer's disease. FDG-PET studies in MCI and AD. Eur. J. Nucl. Med. Mol. Imaging. 2005; 32:486–510. [PubMed: 15747152]

Mosconi L, De Santi S, Brys M, Tsui WH, Pirraglia E, et al. Hypometabolism and altered cerebrospinal fluid markers in normal apolipoprotein E E4 carriers with subjective memory complaints. Biol. Psychiatry. 2008; 63:609–18. [PubMed: 17720148]

Mosconi L, Perani D, Sorbi S, Herholz K, Nacmias B, et al. MCI conversion to dementia and the APOE genotype: a prediction study with FDG-PET. Neurology. 2004; 63:2332–40. [PubMed: 15623696]

NIH-PA Author Manuscript NIH-PA Author Manuscript NIH-PA Author Manuscript

Mosconi L, Rinne JO, Tsui WH, Berti V, Li Y, et al. Increased fibrillar amyloid-β burden in normal individuals with a family history of late-onset Alzheimer's. Proc. Natl. Acad. Sci. USA. 2010; 107:5949–54. [PubMed: 20231448]

Naj AC, Jun G, Beecham GW, Wang LS, Vardarajan BN, et al. Common variants at MS4A4/ MS4A6E, CD2AP, CD33 and EPHA1 are associated with late-onset Alzheimer's disease. Nat. Genet. 2011; 43:436–41. [PubMed: 21460841]

O'Brien JL, O'Keefe KM, LaViolette PS, DeLuca AN, Blacker D, et al. Longitudinal fMRI in elderly reveals loss of hippocampal activation with clinical decline. Neurology. 2010; 74:1969–76. [PubMed: 20463288]

Ogawa S, Tank DW, Menon R, Ellermann JM, Kim SG, et al. Intrinsic signal changes accompanying sensory stimulation: functional brain mapping with magnetic resonance imaging. Proc. Natl. Acad. Sci. USA. 1992; 89:5951–55. [PubMed: 1631079]

Okello A, Edison P, Archer HA, Turkheimer FE, Kennedy J, et al. Microglial activation and amyloid deposition in mild cognitive impairment: a PET study. Neurology. 2009; 72:56–62. [PubMed: 19122031]

Pappata S, Salvatore E, Postiglione A. In vivo imaging of neurotransmission and brain receptors in dementia. J. Neuroimaging. 2008; 18:111–24. [PubMed: 18380693]

Pariente J, Cole S, Henson R, Clare L, Kennedy A, et al. Alzheimer's patients engage an alternative network during a memory task. Ann. Neurol. 2005; 58:870–79. [PubMed: 16315273]

Petersen RC, Smith GE, Waring SC, Ivnik RJ, Tangalos EG, Kokmen E. Mild cognitive impairment: clinical characterization and outcome. Arch. Neurol. 1999; 56:303–8. [PubMed: 10190820]

Pike KE, Ellis KA, Villemagne VL, Good N, Chetelat G, et al. Cognition and beta-amyloid in preclinical Alzheimer's disease: data from the AIBL study. Neuropsychologia. 2011; 49:2384–90. [PubMed: 21529702]

Potkin SG, Guffanti G, Lakatos A, Turner JA, Kruggel F, et al. Hippocampal atrophy as a quantitative trait in a genome-wide association study identifying novel susceptibility genes for Alzheimer's disease. PLoS ONE. 2009a; 4:e6501. [PubMed: 19668339]

Potkin SG, Turner JA, Guffanti G, Lakatos A, Torri F, et al. Genome-wide strategies for discovering genetic influences on cognition and cognitive disorders: methodological considerations. Cogn. Neuropsychiatry. 2009b; 14:391–418. [PubMed: 19634037]

Rami L, Sala-Llonch R, Sole-Padulles C, Fortea J, Olives J, et al. Distinct functional activity of the precuneus and posterior cingulate cortex during encoding in the preclinical stage of Alzheimer's disease. J. Alzheimers Dis. 2012; 31:517–26. [PubMed: 22596271]

Reiman EM, Chen K, Liu X, Bandy D, Yu M, et al. Fibrillar amyloid-beta burden in cognitively normal people at 3 levels of genetic risk for Alzheimer's disease. Proc. Natl. Acad. Sci. USA. 2009; 106:6820–25. [PubMed: 19346482]

Reisberg B, Shulman MB, Torossian C, Leng L, Zhu W. Outcome over seven years of healthy adults with and without subjective cognitive impairment. Alzheimers Dement. 2010; 6:11–24. [PubMed: 20129317]

Rentz DM, Locascio JJ, Becker JA, Moran EK, Eng E, et al. Cognition, reserve, and amyloid deposition in normal aging. Ann. Neurol. 2010; 67:353–64. [PubMed: 20373347]

Ringman JM, O'Neill J, Geschwind D, Medina L, Apostolova LG, et al. Diffusion tensor imaging in preclinical and presymptomatic carriers of familial Alzheimer's disease mutations. Brain. 2007; 130:1767–76. [PubMed: 17522104]

Ringman JM, Younkin SG, Pratico D, Seltzer W, Cole GM, et al. Biochemical markers in persons with preclinical familial Alzheimer disease. Neurology. 2008; 71:85–92. [PubMed: 18509095]

Rinne JO, Kaasinen V, Jarvenpaa T, Nagren K, Roivainen A, et al. Brain acetylcholinesterase activity in mild cognitive impairment and early Alzheimer's disease. J. Neurol. Neurosurg. Psychiatry. 2003; 74:113–15. [PubMed: 12486280]

Risacher SL, Saykin AJ, West JD, Shen L, Firpi HA, McDonald BC. Baseline MRI predictors of conversion from MCI to probable AD in the ADNI cohort. Curr. Alzheimer Res. 2009; 6:347–61. [PubMed: 19689234]

NIH-PA Author Manuscript NIH-PA Author Manuscript NIH-PA Author Manuscript

Risacher SL, Shen L, West JD, Kim S, McDonald BC, et al. Longitudinal MRI atrophy biomarkers: relationship to conversion in the ADNI cohort. Neurobiol. Aging. 2010; 31:1401–18. [PubMed: 20620664]

Rose SE, McMahon KL, Janke AL, O'Dowd B, de Zubicaray G, et al. Diffusion indices on magnetic resonance imaging and neuropsychological performance in amnestic mild cognitive impairment. J. Neurol. Neurosurg. Psychiatry. 2006; 77:1122–28. [PubMed: 16754694]

Rowe CC, Ellis KA, Rimajova M, Bourgeat P, Pike KE, et al. Amyloid imaging results from the Australian Imaging, Biomarkers and Lifestyle (AIBL) study of aging. Neurobiol. Aging. 2010; 31:1275–83. [PubMed: 20472326]

Sabri O, Kendziorra K, Wolf H, Gertz HJ, Brust P. Acetylcholine receptors in dementia and mild cognitive impairment. Eur. J. Nucl. Med. Mol. Imaging. 2008; 35(Suppl. 1):S30–45. [PubMed: 18228017]

Saykin AJ, Shen L, Foroud TM, Potkin SG, Swaminathan S, et al. Alzheimer's Disease Neuroimaging Initiative biomarkers as quantitative phenotypes: genetics core aims, progress, and plans. Alzheimers Dement. 2010; 6:265–73. [PubMed: 20451875]

Saykin AJ, Wishart HA, Rabin LA, Santulli RB, Flashman LA, et al. Older adults with cognitive complaints show brain atrophy similar to that of amnestic MCI. Neurology. 2006; 67:834–42. [PubMed: 16966547]

Scheef L, Spottke A, Daerr M, Joe A, Striepens N, et al. Glucose metabolism, gray matter structure, and memory decline in subjective memory impairment. Neurology. 2012; 79:1332–39. [PubMed: 22914828]

Schuff N, Capizzano AA, Du AT, Amend DL, O'Neill J, et al. Selective reduction of N-acetylaspartate in medial temporal and parietal lobes in AD. Neurology. 2002; 58:928–35. [PubMed: 11914410]

Shaw LM, Korecka M, Clark CM, Lee VM, Trojanowski JQ. Biomarkers of neurodegeneration for diagnosis and monitoring therapeutics. Nat. Rev. Drug Discov. 2007; 6:295–303. [PubMed: 17347655]

Shaw LM, Vanderstichele H, Knapik-Czajka M, Clark CM, Aisen PS, et al. Cerebrospinal fluid biomarker signature in Alzheimer's Disease Neuroimaging Initiative subjects. Ann. Neurol. 2009; 65:403–13. [PubMed: 19296504]

Shen L, Kim S, Risacher SL, Nho K, Swaminathan S, et al. Whole genome association study of brain-wide imaging phenotypes for identifying quantitative trait loci in MCI and AD: a study of the ADNI cohort. Neuroimage. 2010; 53:1051–63. [PubMed: 20100581]

Small GW, Ercoli LM, Silverman DH, Huang SC, Komo S, et al. Cerebral metabolic and cognitive decline in persons at genetic risk for Alzheimer's disease. Proc. Natl. Acad. Sci. USA. 2000; 97:6037–42. [PubMed: 10811879]

Sperling RA, Aisen PS, Beckett LA, Bennett DA, Craft S, et al. Toward defining the preclinical stages of Alzheimer's disease: recommendations from the National Institute on Aging-Alzheimer's Association workgroups on diagnostic guidelines for Alzheimer's disease. Alzheimers Dement. 2011; 7:280–92. [PubMed: 21514248]

Sperling RA, Laviolette PS, O'Keefe K, O'Brien J, Rentz DM, et al. Amyloid deposition is associated with impaired default network function in older persons without dementia. Neuron. 2009; 63:178–88. [PubMed: 19640477]

Stein JL, Hua X, Lee S, Ho AJ, Leow AD, et al. Voxelwise genome-wide association study (vGWAS). Neuroimage. 2010; 53:1160–74. [PubMed: 20171287]

Stein JL, Medland SE, Vasquez AA, Hibar DP, Senstad RE, et al. Identification of common variants associated with human hippocampal and intracranial volumes. Nat. Genet. 2012; 44:552–61. [PubMed: 22504417]

Stern Y. What is cognitive reserve? Theory and research application of the reserve concept. J. Int. Neuropsychol. Soc. 2002; 8:448–60. [PubMed: 11939702]

Striepens N, Scheef L, Wind A, Popp J, Spottke A, et al. Volume loss of the medial temporal lobe structures in subjective memory impairment. Dement. Geriatr. Cogn. Disord. 2010; 29:75–81. [PubMed: 20110703]

NIH-PA Author Manuscript
NIH-PA Author Manuscript
NIH-PA Author Manuscript

Swaminathan S, Shen L, Risacher SL, Yoder KK, West JD, et al. Amyloid pathway-based candidate gene analysis of [(11)C]PiB-PET in the Alzheimer's Disease Neuroimaging Initiative (ADNI) cohort. Brain Imaging Behav. 2011; 6:1–15. [PubMed: 21901424]

Trachtenberg AJ, Filippini N, Ebmeier KP, Smith SM, Karpe F, Mackay CE. The effects of APOE on the functional architecture of the resting brain. Neuroimage. 2012; 59:565–72. [PubMed: 21851856]

Trivedi MA, Murphy CM, Goetz C, Shah RC, Gabrieli JD, et al. fMRI activation changes during successful episodic memory encoding and recognition in amnestic mild cognitive impairment relative to cognitively healthy older adults. Dement. Geriatr. Cogn. Disord. 2008; 26:123–37. [PubMed: 18663302]

Trivedi MA, Wichmann AK, Torgerson BM, Ward MA, Schmitz TW, et al. Structural MRI discriminates individuals with mild cognitive impairment from age-matched controls: a combined neuropsychological and voxel based morphometry study. Alzheimers Dement. 2006; 2:296–302. [PubMed: 19020671]

Trojanowski JQ, Vandeerstichele H, Korecka M, Clark CM, Aisen PS, et al. Update on the biomarker core of the Alzheimer's Disease Neuroimaging Initiative subjects. Alzheimers Dement. 2010; 6:230–38. [PubMed: 20451871]

Vandenberghe R, Van Laere K, Ivanoiu A, Salmon E, Bastin C, et al. 18F-flutemetamol amyloid imaging in Alzheimer disease and mild cognitive impairment: a phase 2 trial. Ann. Neurol. 2010; 68:319–29. [PubMed: 20687209]

Vemuri P, Weigand SD, Przybelski SA, Knopman DS, Smith GE, et al. Cognitive reserve and Alzheimer's disease biomarkers are independent determinants of cognition. Brain. 2011; 134:1479–92. [PubMed: 21478184]

Venneti S, Wiley CA, Kofler J. Imaging microglial activation during neuroinflammation and Alzheimer's disease. J. Neuroimmune Pharmacol. 2009; 4:227–43. [PubMed: 19052878]

Villemagne VL, Pike KE, Darby D, Maruff P, Savage G, et al. Abeta deposits in older non-demented individuals with cognitive decline are indicative of preclinical Alzheimer's disease. Neuropsychologia. 2008; 46:1688–97. [PubMed: 18343463]

Walsh DM, Selkoe DJ. A beta oligomers—a decade of discovery. J. Neurochem. 2007; 101:1172–84. [PubMed: 17286590]

Wang Y, Saykin AJ, Pfeuffer J, Lin C, Mosier KM, et al. Regional reproducibility of pulsed arterial spin labeling perfusion imaging at 3T. Neuroimage. 2011; 54:1188–95. [PubMed: 20800097]

Wang Y, West JD, Flashman LA, Wishart HA, Santulli RB, et al. Selective changes in white matter integrity in MCI and older adults with cognitive complaints. Biochim. Biophys. Acta. 2012; 1822:423–30. [PubMed: 21867750]

Weiner MW, Aisen PS, Jack CR Jr, Jagust WJ, Trojanowski JQ, et al. The Alzheimer's Disease Neuroimaging Initiative: progress report and future plans. Alzheimers Dement. 2010; 6:202–11.e7. [PubMed: 20451868]

Weiner MW, Veitch DP, Aisen PS, Beckett LA, Cairns NJ, et al. The Alzheimer's Disease Neuroimaging Initiative: a review of papers published since its inception. Alzheimers Dement. 2011; 8(1 Suppl):S1–6. [PubMed: 22047634]

Whitwell JL, Przybelski SA, Weigand SD, Knopman DS, Boeve BF, et al. 3D maps from multiple MRI illustrate changing atrophy patterns as subjects progress from mild cognitive impairment to Alzheimer's disease. Brain. 2007; 130:1777–86. [PubMed: 17533169]

Whitwell JL, Shiung MM, Przybelski SA, Weigand SD, Knopman DS, et al. MRI patterns of atrophy associated with progression to AD in amnestic mild cognitive impairment. Neurology. 2008; 70:512–20. [PubMed: 17898323]

Wiley CA, Lopresti BJ, Venneti S, Price J, Klunk WE, et al. Carbon 11-labeled Pittsburgh compound B and carbon 11-labeled (R)-PK11195 positron emission tomographic imaging in Alzheimer disease. Arch. Neurol. 2009; 66:60–67. [PubMed: 19139300]

Wishart HA, Saykin AJ, McAllister TW, Rabin LA, McDonald BC, et al. Regional brain atrophy in cognitively intact adults with a single APOE epsilon4 allele. Neurology. 2006a; 67:1221–24. [PubMed: 17030756]

NIH-PA Author Manuscript
NIH-PA Author Manuscript
NIH-PA Author Manuscript

Wishart HA, Saykin AJ, Rabin LA, Santulli RB, Flashman LA, et al. Increased brain activation during working memory in cognitively intact adults with the APOE epsilon4 allele. Am. J. Psychiatry. 2006b; 163:1603–10. [PubMed: 16946187]

Wong DF, Rosenberg PB, Zhou Y, Kumar A, Raymont V, et al. In vivo imaging of amyloid deposition in Alzheimer disease using the radioligand 18F-AV-45 (florbetapir [corrected] F 18). J. Nucl. Med. 2010; 51:913–20. [PubMed: 20501908]

Xu C, Wang Z, Fan M, Liu B, Song M, et al. Effects of BDNF Val66Met polymorphism on brain metabolism in Alzheimer's disease. Neuroreport. 2010; 21:802–7. [PubMed: 20613678]

Zhang Y, Schuff N, Jahng GH, Bayne W, Mori S, et al. Diffusion tensor imaging of cingulum fibers in mild cognitive impairment and Alzheimer disease. Neurology. 2007; 68:13–19. [PubMed: 17200485]

Zhou Y, Dougherty JH Jr, Hubner KF, Bai B, Cannon RL, Hutson RK. Abnormal connectivity in the posterior cingulate and hippocampus in early Alzheimer's disease and mild cognitive impairment. Alzheimers Dement. 2008; 4:265–70. [PubMed: 18631977]

NIH-PA Author Manuscript
NIH-PA Author Manuscript
NIH-PA Author Manuscript

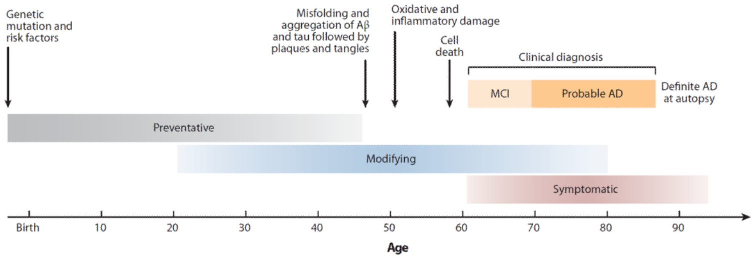

Figure 1.
Hypothetical timeline for the progression of neuropathology and clinical symptoms associated with Alzheimer's disease (AD). This figure shows a theoretical timeline for the progression of AD-related neuropathology and clinical changes, with changes in amyloid and tau pathology occurring years before the onset of AD. The gray-, blue-, and red-shaded bars reflect time points at which different types of potential interventions may be beneficial (gray, preventative; blue, disease modifying; red, symptomatic). Reprinted by permission from Macmillan Publishers Ltd: Nature Reviews Drug Discovery, [Shaw LM et al., Biomarkers of neurodegeneration for diagnosis and monitoring therapeutics. *Nat. Rev. Drug Discov*. 6:295–303], copyright 2007. MCI, mild cognitive impairment.

NIH-PA Author Manuscript
NIH-PA Author Manuscript
NIH-PA Author Manuscript

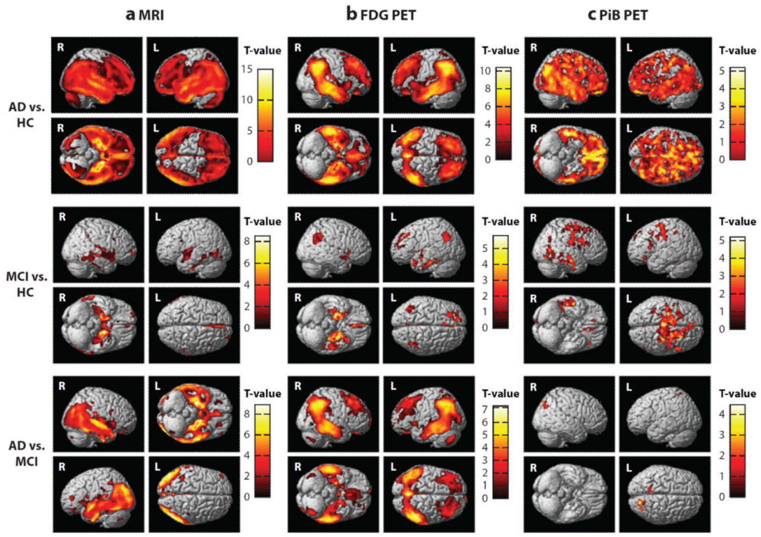

Figure 2.
Differences in brain atrophy, glucose metabolism, and amyloid deposition between patients with Alzheimer's disease (AD) and mild cognitive impairment (MCI) and healthy older adults controls (HCs). The pattern of differences between AD, MCI, and HC is demonstrated in (*a*) brain atrophy [measured using structural magnetic resonance imaging (MRI)], (*b*) glucose metabolism [measured using [^{18}F]fluorodeoxyglucose positron emission tomography (FDG PET)], and (*c*) amyloid accumulation [measured using [^{11}C]Pittsburgh compound B positron emission tomography (PiB PET)]. Relative to HC, patients with AD show significantly reduced brain gray matter (GM) density throughout cortical and subcortical regions (*a*; AD versus HC), reduced glucose metabolism in regions of the medial and lateral parietal lobe, medial and lateral temporal lobes, and medial and lateral frontal lobes (*b*; AD versus HC), as well as increased amyloid accumulation throughout the cerebral cortex (*c*; AD versus HC). MCI patients also show similar, although more focal, changes relative to HC, including reduced GM density in the medial and lateral temporal lobes (*a*; MCI versus HC), reduced glucose metabolism in the medial and lateral temporal lobes, medial and lateral parietal lobes, and frontal lobe (*b*; MCI versus HC), and greater amyloid deposition in the frontal, parietal, and temporal cortices (*c*; MCI versus HC). The comparisons of these measures between patients with AD to patients with MCI show interesting patterns of association with disease severity. Specifically, patients with AD show significantly more GM atrophy in regions of the medial and lateral temporal lobes and parietal lobes (*a*; AD versus MCI) and reduced glucose metabolism in the medial and lateral temporal lobes, medial and lateral parietal lobes, and frontal lobe (*b*; AD versus MCI) relative to MCI patients. However, only minor differences in amyloid accumulation between AD and MCI patients are observed (*c*; AD versus MCI), suggesting the majority of amyloid accumulation occurs before a participant has reached a clinical diagnosis of MCI, as has been previously reported (Jack et al. 2009, 2010b). This figure was generated using data from the Alzheimer's Disease Neuroimaging Initiative cohort and utilizing traditional methods that have been previously described (Jagust et al. 2010, Risacher et al. 2009, Swaminathan et al. 2011). Panel *a* is displayed at a voxel-wise threshold of $p < 0.01$ (family-wise error correction for multiple comparisons) and minimum cluster size (k) = 50 voxels and includes 189 AD, 396 MCI, and 225 HC participants; panel *b* is displayed at a voxel-wise threshold of $p < 0.001$ (uncorrected for multiple comparisons) and k = 50 voxels and includes 97 AD, 203 MCI, and 102 HC participants; panel *c* is displayed at a voxel-wise

threshold of $p < 0.01$ (uncorrected for multiple comparisons) and $k = 50$ voxels and includes 25 AD, 56 MCI, and 22 HC participants.

NIH-PA Author Manuscript
NIH-PA Author Manuscript
NIH-PA Author Manuscript

NIH-PA Author Manuscript

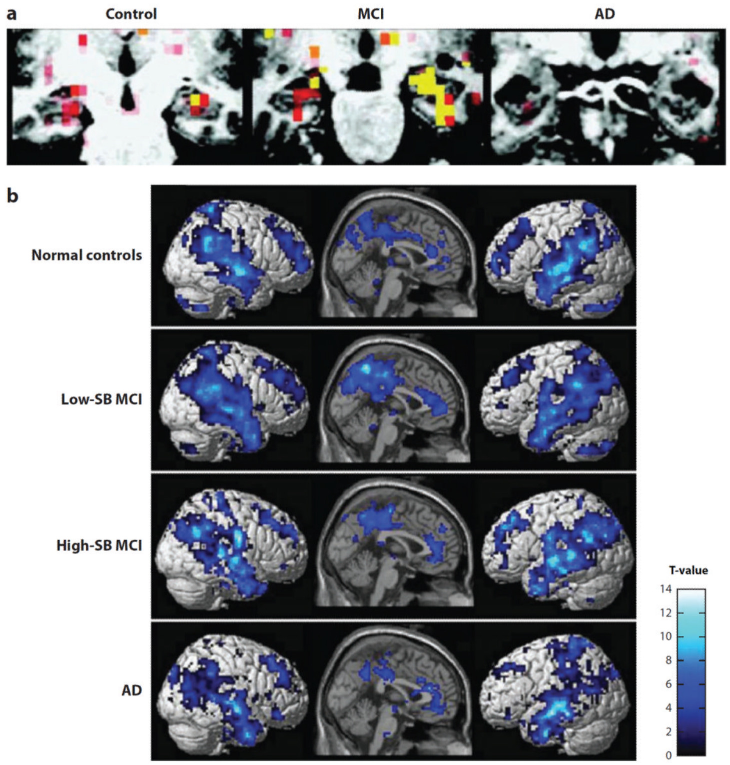

Figure 3.
Functional MRI activation and deactivation patterns in Alzheimer's disease (AD), mild cognitive impairment (MCI), and healthy control (HC) participants. This figure demonstrates (*a*) patterns of activation in the medial temporal lobe and patterns of (*b*) deactivation in the default mode network (DMN) during a memory task in patients with AD and MCI, as well as HCs. (*a*) During a face-name encoding task, patients with AD showed significantly reduced hippocampal activation relative to HCs and patients with MCI. However, patients with MCI showed increased hippocampal activation relative to HCs. (*b*) Upon initiation of an episodic encoding task, MCI and AD patients demonstrated altered deactivation of the DMN, which includes the medial and lateral parietal lobes, medial and lateral temporal lobes, and prefrontal cortex. However, these changes were different by disease severity. Specifically, less impaired MCI patients [low CDR-Sum of Boxes score (low SB)] demonstrated increased connectivity in the DMN relative to HC, whereas more impaired MCI (high-SB) and AD patients showed decreased DMN connectivity relative to HCs. Panel *a* is adapted with permission from Dickerson BC et al. 2005. Increased hippocampal activation in mild cognitive impairment compared to normal aging and AD. *Neurology* 65:404–11. Panel *b* is adapted with permission from Celone KA et al. 2006. Alterations in memory networks in mild cognitive impairment and Alzheimer's disease: an independent component analysis. *J. Neurosci.* 26:10222–31.

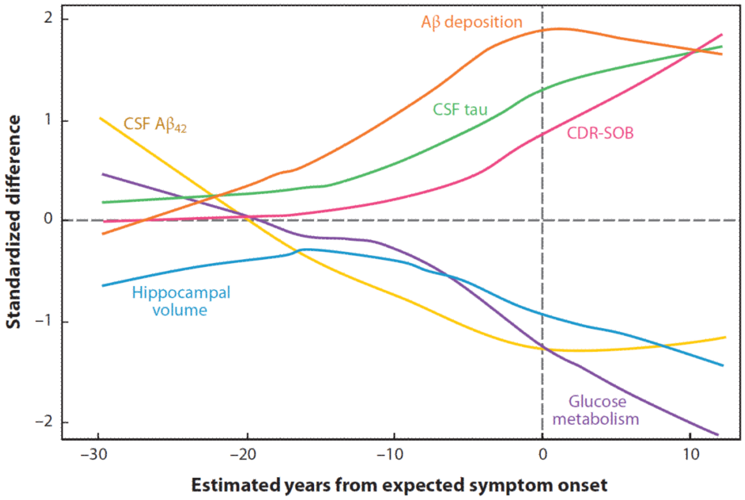

Figure 4.

Comparison of neuroimaging, cerebrospinal fluid (CSF), and cognitive measures relative to the estimated age of symptom onset in patients with familial Alzheimer's disease (AD). The relative difference between carriers of autosomal dominant AD mutations [mutation carriers (MCs)] and noncarriers (NCs) in neuroimaging, CSF, and cognitive measures at multiple time points prior to and after the estimated age of symptom onset is shown. Measures of amyloid-beta (Aβ) appear to change earliest in MCs relative to NCs, followed by measures of CSF tau, hippocampal atrophy, and glucose hypometabolism. All of the neuroimaging and CSF biomarkers show alterations in MCs relative to NCs before the estimated age of symptom onset. Reproduced from *The New England Journal of Medicine*, Bateman RJ et al., Clinical and biomarker changes in dominantly inherited Alzheimer's disease, 367, 795–804, © 2012 Massachusetts Medical Society. Reprinted with permission from Massachusetts Medical Society. CDR-SOB, Clinical Dementia Rating Scale, Sum of Boxes score.

NIH-PA Author Manuscript
NIH-PA Author Manuscript
NIH-PA Author Manuscript

NIH-PA Author Manuscript
NIH-PA Author Manuscript
NIH-PA Author Manuscript

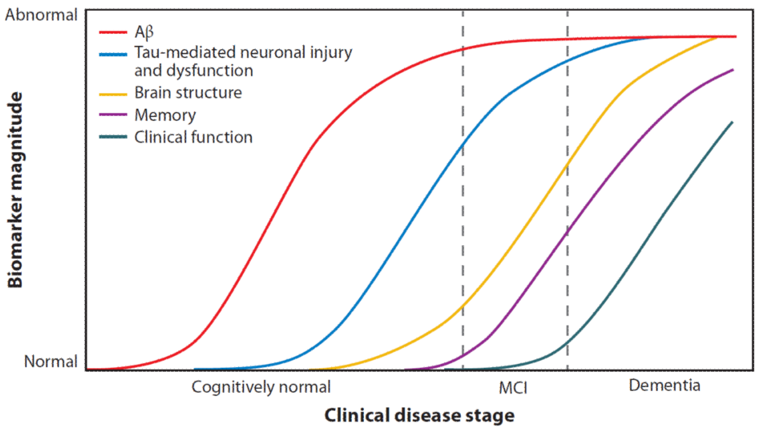

Figure 5.

Hypothetical model of biomarker change across the cascade of Alzheimer's disease (AD) pathologic progression and clinical decline. This figure represents a theoretical framework for the ordering and dynamic sensitivity of various AD biomarkers across the pathologic and clinical AD spectrum. Specifically, this model suggests that the earliest neuropathological changes occur with the accumulation of amyloid-beta (Aβ), followed by neuronal injury and dysfunction, then neurodegeneration, and finally cognitive and clinical decline. These various stages can be monitored using known AD biomarkers, including monitoring of Aβ accumulation with cerebrospinal fluid (CSF) and positron emission tomography (PET) measures, neuronal injury and dysfunction with CSF tau measures, [^{18}F]fluorodeoxyglucose PET, and potentially functional magnetic resonance imaging (MRI) measures, neurodegeneration with structural MRI, and cognition and clinical decline with psychometric and clinical testing. Reprinted from *The Lancet Neurology*, Volume 9(1), Jack CR Jr et al., Hypothetical model of dynamic biomarkers of the Alzheimer's pathological cascade, 119–28, copyright 2010, with permission from Elsevier. MCI, mild cognitive impairment.

NIH-PA Author Manuscript

NIH-PA Author Manuscript

NIH-PA Author Manuscript

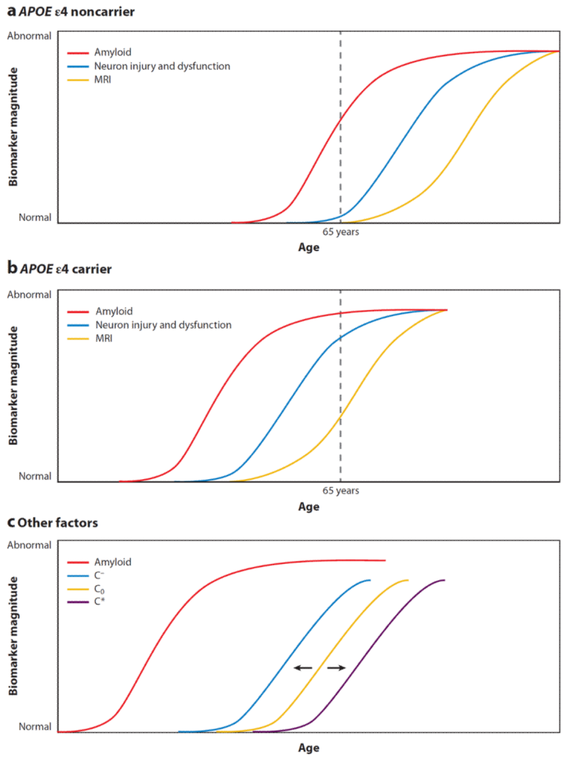

Figure 6.

Modulation of biomarker curves by genetics [apolipoprotein E (*APOE*) genotype] and cognitive reserve. Factors such as genetic variations and cognitive reserve may shift the hypothetical biomarker curves relative to age and disease stage. Genetic variation in the *APOE* gene may shift the amyloid-beta (Aβ) and neurodegeneration curves, with (*a*) individuals positive for *APOE* ε4 showing a leftward shift (pathology occurring at an earlier age) and/or (*b*) individuals negative for *APOE* ε4 showing a rightward shift (pathology occurring at a later age). (*c*) Other cognitive and/or health and lifestyle factors may also shift these hypothetical curves, particularly the curve representing changes in cognition. Those with low cognitive reserve and/or with a high number of medical comorbidities (C^-) may show a leftward shift of the cognition curve, whereas those with high cognitive reserve and/or few medical comorbidities (C*) may show a rightward shift, relative to the initially hypothesized cognition curve (C_o). Reprinted from *The Lancet Neurology*, Volume 9(1), Jack CR Jr et al., Hypothetical model of dynamic biomarkers of the Alzheimer's pathological cascade, 119–28, copyright 2010, with permission from Elsevier. MRI, magnetic resonance imaging.

Statistics of highlighted entities of class BIM (page 1)

Entity	Frequency
Manuscript	91
manuscript	31
tomography	11
performance	6
standard	3
manual	2
article	2
collection	1
organization	1
Journal	1
report	1
Access	1
group	1
groups	1

Statistics of highlighted entities of class NIFT (page 1)

Entity	Frequency
CSF	35
MRI	33
tau	25
PET	25
atrophy	22
brain atrophy	20
hippocampal	17
Alzheimer disease	16
hippocampus	15
[11C]PiB	13
parietal lobe	12
amyloid deposition	12
fMRI	12
frontal lobe	11
DTI	10
positron emission tomography	10
FDG PET	8
structural MRI	7
parietal lobes	7
cerebrospinal fluid	7
frontal lobes	7
perfusion	6
MRS	6
precuneus	6
hippocampal atrophy	6
p-tau	5
FA	5
NAA	5
Pittsburgh compound B	4
amyloid imaging	4
brain regions	4
gray matter	4
diffusion tensor imaging	4
DWI	4
magnetic resonance imaging	4
cerebral metabolism	4
functional MRI	4
neocortex	3
hypoperfusion	3
perfusion imaging	3

Statistics of highlighted entities of class NIFT (page 2)

Entity	Frequency
hippocampal volume	3
cortical thickness	3
WM atrophy	3
functional magnetic resonance imaging	3
entorhinal cortex	3
cerebral cortex	3
brain metabolism	3
[18F]fluorodeoxyglucose	3
amygdala	3
serotonin	3
white matter	3
magnetic resonance spectroscopy	3
arterial spin labeling	3
cerebral perfusion	2
voxel-based morphometry	2
dopamine	2
BOLD	2
Amyloid imaging	2
cortical atrophy	2
Voxel-based morphometry	2
cerebral glucose metabolism	2
Structural MRI	2
N-acetylaspartate	2
Functional MRI	2
rate of atrophy	2
hypometabolism	2
neocortical	2
voxel based morphometry	2
myo-inositol	2
acetylcholine	2
Pittsburgh compound-B	2
cerebral blood volume	2
PiB	2
CBF	2
Diffusion tensor imaging	2
occipital lobe	2
structural connectivity	1
brain volume	1
Hypometabolism	1
American National Adult Reading Test	1

Statistics of highlighted entities of class NIFT (page 3)

Entity	Frequency
positron-emission tomography	1
diffusion weighted imaging	1
Hippocampal atrophy	1
neurotransmitter activity	1
Cerebrospinal fluid	1
pattern of atrophy	1
Arterial spin labeling	1
mean diffusivity	1
(FA)	1
blood oxygenation	1
parahippocampal gyrus	1
arterial spin-labeling	1
ventricular expansion	1
fractional anisotropy	1
Acetylcholine	1
Medial temporal atrophy	1
thalamus	1
Clinical Dementia Rating	1
white matter integrity	1
PIB	1
Magnetic resonance imaging	1
1H MRS	1
glucose hypometabolism	1
Tensor-based morphometry	1
[11C]PIB	1
Abeta42	1
p- tau	1
basal nucleus of Meynert	1
structural imaging	1
cerebellum	1
PET ligands	1
t-tau	1
blood oxygen level	1
entorhinal volume	1
scores	1
blood- oxygenation-level-dependent (BOLD)	1
resting-state fMRI	1
FDG- PET	1
axonal integrity	1
blood oxygen levels	1

Statistics of highlighted entities of class NIFT (page 4)

Entity	Frequency
Hippocampal	1
structural magnetic resonance imaging	1
2-[(18)F]fluoro-2-deoxy-D-glucose	1
PET tracers	1
neuronal function	1
Amyloid deposition	1
18F-AV-45	1
focal cortical atrophy	1
Changes in cerebral blood flow	1
Medial temporal lobe atrophy	1
FDG-PET	1
amygdalar volume	1
18F-flutemetamol	1
11C PiB	1
Dynamic susceptibility contrast MR imaging	1

Statistics of highlighted entities of class QIBO (page 1)

Entity	Frequency
disease	120
brain	77
activation	29
hippocampal	26
preclinical	23
diagnosis	21
Disease	18
cortex	17
cerebral	16
Brain	15
hippocampus	15
volume	14
cortical	13
blood	11
density	10
positron emission tomography	10
thickness	5
neurotransmitter	5
model	4
cortices	4
area	3
DISEASE	3
Volume	3
cell	3
magnetic resonance spectroscopy	3
Hippocampal	2
Cerebral	2
amount	2
Activation	1
Functional MRI (fMRI)	1
cells	1
Staging	1
mouse	1
Cell	1
apoptosis	1
Correlation	1
positron-emission tomography	1
PRECLINICAL	1
enzyme	1
Diagnosis	1

Statistics of highlighted entities of class QIBO (page 2)

Entity	Frequency
Raman	1
enlargement	1
functional MRI (fMRI)	1
correlation	1
Cortical	1
syndrome	1
biological processes	1
cerebral blood flow	1
cerebellum	1

Key for highlighting of entities

Class	Color	Overall Frequency
BIM		153
NIFT		552
QIBO		495
