# Supplementary material for: Neuroimaging Feature Terminology: A Controlled Terminology for the Annotation of Brain Imaging Features
Source: J Alzheimers Dis. 2017 Aug 14;59(4):1153–69. doi: 10.3233/JAD-161148 (PMC5611802; doi:10.3233/JAD-161148)
Supplement: Supplementary file 1 [file jad-59-jad161148-s001.zip › Supplementary_File1/Neuroimaging Biomarkers of Neurodegenerative Diseases.pdf]

NIH-PA Author Manuscript
NIH-PA Author Manuscript
NIH-PA Author Manuscript

NIH Public Access
Author Manuscript
Semin Neurol. Author manuscript; available in PMC 2014 November 14.

Published in final edited form as:
Semin Neurol. 2013 September ; 33(4): 386–416. doi:10.1055/s-0033-1359312.

Neuroimaging Biomarkers of Neurodegenerative Diseases and Dementia

Shannon L. Risacher, PhD[1] and **Andrew J. Saykin, PsyD**[1]
[1]Center for Neuroimaging, Department of Radiology and Imaging Sciences, and Indiana Alzheimer Disease Center Indiana University School of Medicine, Indianapolis, Indiana

Abstract

Neurodegenerative disorders leading to dementia are common diseases that affect many older and some young adults. Neuroimaging methods are important tools for assessing and monitoring pathological brain changes associated with progressive neurodegenerative conditions. In this review, the authors describe key findings from neuroimaging studies (magnetic resonance imaging and radionucleotide imaging) in neurodegenerative disorders, including Alzheimer's disease (AD) and prodromal stages, familial and atypical AD syndromes, frontotemporal dementia, amyotrophic lateral sclerosis with and without dementia, Parkinson's disease with and without dementia, dementia with Lewy bodies, Huntington's disease, multiple sclerosis, HIV-associated neurocognitive disorder, and prion protein associated diseases (i.e., Creutzfeldt-Jakob disease). The authors focus on neuroimaging findings of *in vivo* pathology in these disorders, as well as the potential for neuroimaging to provide useful information for differential diagnosis of neurodegenerative disorders.

Keywords

neuroimaging; dementia; Alzheimer's disease (AD); mild cognitive impairment (MCI); frontotemporal dementia (FTD); amyotrophic lateral sclerosis (ALS); dementia with Lewy bodies (DLB); Parkinson's disease (PD); Huntington's disease (HD); multiple sclerosis (MS); HIV-associated neurocognitive disorder (HAND); Cruetzfeldt-Jakob disease (CJD); Gerstmann-Straussler-Scheinker disease (GSS)

Neurodegenerative diseases and dementias feature progressive and often irreversible degeneration of cells within the central nervous system (CNS). Although primarily affecting older adults, some forms of neurodegenerative disease (such as variant Creutzfeldt-Jakob disease [CJD], multiple sclerosis [MS], and HIV-associated neurocognitive disorder [HAND]) can affect younger individuals.[1,2] With disparate, but sometimes overlapping clinical presentations and etiologies, neurodegenerative disorders and dementias can be difficult to correctly diagnose. Neuroimaging techniques have the potential to assist with clinical diagnosis and monitoring of disease progression in most, if not all, of the neurodegenerative disorders. Our goal here is to provide an overview of neuroimaging findings in the most common neurodegenerative conditions, as well as recent developments in each area (Table 1).

Copyright © 2013 by Thieme Medical Publishers, Inc.

Address for correspondence: Andrew J. Saykin, PsyD, ABPP-CN, Department of Radiology and Imaging Sciences, Center for Neuroimaging, Indiana University School of Medicine, IU Health Neuroscience Center, Suite 4100, 355 West 16th Street, Indianapolis, IN 46202 (asaykin@iupui.edu).

NIH-PA Author Manuscript NIH-PA Author Manuscript NIH-PA Author Manuscript

Degenerative Diseases and Dementias

Alzheimer's disease (AD) is the most common age-related neurodegenerative disease, affecting more than 5 million individuals in the United States, mostly age 65 or older, and that number is expected to more than triple by 2050.[3] The earliest clinical symptoms are memory impairments, particularly in episodic and semantic domains, as well as deficits in language and executive functioning.[4] Patients with AD also show a significant impairment in daily functioning with disruption or cessation of the ability to perform complex activities and later more simple tasks. Clinicians and researchers have recently updated AD diagnostic criteria for use in clinical practice and research.[4] Currently, the diagnosis of AD is made clinically, based on cognition and the relative impact of impairments on daily activities. Attempts to diagnose AD at an earlier stage have led to the development of a clinical syndrome termed amnestic mild cognitive impairment (MCI).[5] Recently, new criteria for diagnosis of MCI in clinical and research settings have been published.[6] Patients with MCI typically show deficits in episodic memory that fall more than 1 standard deviation below age and education adjusted and culturally appropriate normative levels.[6] More recently, researchers have proposed dividing MCI into an earlier stage (early MCI [E-MCI]) and a later stage (late MCI [L-MCI]), with E-MCI patients showing a 1 to 1.5 standard deviation memory deficit and L-MCI showing a greater than 1.5 standard deviation deficit. This classification has only recently been introduced and future studies will help to elucidate differences between these MCI subgroups. The most common presentation of MCI features memory impairment (amnestic MCI), but can co-occur with other cognitive deficits such as executive function or language deficits (multidomain MCI).[6] Amnestic MCI is widely considered to be a prodromal form of AD, as nearly 10 to 15% of amnestic L-MCI patients convert to probable AD each year, relative to only 1 to 2% of the general older adult population.[5] Recently, researchers and clinicians have been attempting to detect AD-related changes and predict progression even earlier than MCI (e.g., pre-MCI or preclinical AD). A conceptual framework for identifying preclinical AD patients has been presented in a recent article.[7]

Alzheimer's disease is characterized by two neuropathological hallmarks: amyloid plaques and neurofibrillary tangles. Amyloid plaques are extracellular aggregations of the amyloid-β (Aβ) peptide that are found throughout the brain of AD patients. Neurofibrillary tangles result from the hyperphosphorylation of the microtubule-associated protein tau, which forms insoluble filamentous structures that combine to create paired helical filaments, a key component of the neurofibrillary tangles seen in the brains of patients with AD. The temporal relationship and direct link between amyloid plaques and neurofibrillary tangles is not completely elucidated at this time. Current theories suggest that amyloid plaque formation precedes neurofibrillary tangles, with amyloid accumulation occurring during a long preclinical period lasting years to decades.[8] The biochemical processes involved in Alzheimer's disease development ultimately converge upon widespread cell death and neuronal loss, likely through apoptosis. The first regions of the brain to show neuronal loss associated with AD are in the medial temporal lobe (MTL), including the entorhinal cortex, hippocampus, amygdala, and parahippocampal cortex, as well as cholinergic innervations to the neocortex from the nucleus basalis of Meynert.[9] By the time a patient has reached a diagnosis of AD, neurodegeneration is usually found throughout the neocortex and subcortical regions, with significant atrophy of the temporal, parietal, and frontal cortices, but relative sparing of the primary occipital cortex and primary sensory–motor regions.[9]

Although the majority of AD cases represent late-onset or sporadic AD, nearly 5% of AD cases are caused by dominantly inherited genetic mutations, usually in one of three genes: amyloid precursor protein (*APP*), presenilin 1 (*PS1*), or presenilin 2 (*PS2*). Often featuring an onset of symptoms that is at an earlier age than sporadic AD patients (i.e., before age 65),

NIH-PA Author Manuscript NIH-PA Author Manuscript NIH-PA Author Manuscript

these cases are referred to as familial AD or early-onset AD. Although these diseases can show somewhat different symptomology and pathology than late-onset AD, the major AD hallmarks (i.e., amyloid plaques, neurofibrillary tangles) are present. Therefore, these patients may represent a useful sample for studying early changes in biomarkers, particularly because the age of symptom onset tends to be consistent across generations. Therefore, using an estimated age of symptom onset (EAO), changes in neuropathology and cognition can be assessed using biomarkers decades before onset of disease.[10] Other diseases associated with AD neuropathology show atypical presentation, including posterior cortical atrophy (PCA) and logopenic aphasia. Posterior cortical atrophy is a disorder of higher visual function that causes significant visual dysfunction in the absence of ocular disease, as well as constructional apraxia, visual field deficits, and environmental disorientation.[11,12] This disorder is primarily thought to be associated with changes in posterior brain regions, including the parietal and occipital lobes. Logopenic aphasia is a type of primary progressive aphasia (PPA) associated with AD (i.e., amyloid) rather than frontotemporal dementia-(FTD-) like pathology and features impaired word retrieval and sentence repetition in the absence of motor speech or grammatical abnormalities.[13] Cerebral amyloid angiopathy (CAA) is also associated with AD-like amyloid pathology. However, amyloid deposits are largely observed in the walls of small cerebral arteries and capillaries in CAA.[14] Patients with CAA often show cognitive decline, seizures, headaches, and stroke-like symptoms.[15]

Vascular dementia and vascular-associated cognitive impairment (VCI), a form of cognitive impairment with notable cerebrovascular pathology and/or risk factors, can be identified using self-reports of stroke and/or other vascular events or diseases (myocardial infarction, atherosclerosis, hypertension, etc.), neurologic and psychometric evaluation, and/or structural and functional imaging techniques. The major requirements for a diagnosis of vascular dementia or vascular-associated MCI include the presence of clinically significant cognitive impairments, which can be in any cognitive domain, but are commonly observed in executive function and/or memory, and the presence of significant cerebrovascular pathology and/or risk factors, assessed using clinical or neuroimaging techniques. Beyond these requirements, patients are diagnosed by clinical severity and the impact on activities of daily living (ADLs), similar to the diagnosis of AD. Specifically, patients diagnosed with vascular-associated MCI must show a cognitive deficit, but no significant impairment in ADLs, whereas a diagnosis of vascular dementia requires significant impairment in both clinically assessed cognitive status and ADLs.

Frontotemporal dementia (FTD) is an overarching diagnosis that encompasses multiple disorders with varying symptoms. Behavioral variant FTD (bvFTD) is characterized by a change in personality and behavior, disinhibition, apathy, loss of empathy, obsessive–compulsive behaviors, and changes in appetite.[13,16,17] Behavioral variant FTD is most commonly associated with pathological tau accumulation, such as seen in Pick's disease, but can also feature accumulation of a TAR-DNA-binding protein called TDP-43.[13,17] Primary progressive aphasia (PPA) is another form of FTD, which is divided into two forms: semantic dementia (SD) and progressive non-fluent aphasia (PNFA). Semantic dementia features fluent aphasia, anomia, and single-word comprehension deficits and later in the disease course behavioral symptoms similar to those seen bvFTD. Pathologically, TDP-43 accumulation usually underlies SD, but rare cases featuring tau pathology associated with Pick's disease have been observed.[13,17] Progressive nonfluent aphasia features speech production difficulties with agrammatism and apraxia of speech, as well as phonemic errors, anomia, and impairments in sentence comprehension.[13] Progressive nonfluent aphasia typically features changes due to tau pathology, although mutations in the progranulin gene (*GRN*) resulting in TDP-43 pathology, can cause PNFA symptoms, but without apraxia of speech.[13,17] Frontotemporal dementia can also feature motor dysfunction and motor neuron

NIH-PA Author Manuscript NIH-PA Author Manuscript NIH-PA Author Manuscript

disease (MND).[13,17,18] These disorders have been linked to Parkinson's-like symptoms, such as those seen in corticobasal degeneration (CBD) and progressive supranuclear palsy (PSP), which feature tau pathology, or changes due to TDP-43 pathology, which presents as FTD-MND with Lewy body-like pathology or FTD associated with amyotrophic lateral sclerosis (FTD-ALS).[13,17,18] Clinically, the Parkinson's-like FTD dementias (CBD and PSP) can show either behavioral-type symptoms (i.e., those seen in bvFTD) or language-type symptoms (most commonly PFNA-like symptoms), along with executive dysfunction, in the presence of cortical and extrapyramidal motor dysfunction.[13] Patients with FTD associated with TDP-43 (FTD-ALS, others) most commonly present with behavioral symptoms (bvFTD-like) in the presence of motor dysfunction.[18] Amyotrophic lateral sclerosis can also occur without behavioral symptoms, although non-FTD ALS patients commonly still have subthreshold cognitive changes.[19]

Parkinson's disease (PD) is caused by deposition of inclusions of α-synuclein called Lewy bodies and feature spontaneous motor parkinsonism, visual hallucinations, and potentially changes in cognition[20]; 70 to 80% of patients with PD develop cognitive impairment and/or dementia over the course of the disease.[20,21] Two types of Parkinson's dementias have been defined, including Parkinson's disease dementia (PDD), in which patients develop cognitive symptoms more than 1 year after motor symptoms, and dementia with Lewy bodies (DLB), in which patients develop cognitive symptoms concurrent with or within a year of motor symptoms. [20] Cognitive symptoms in PDD and DLB are variable, but often feature impairments in visual spatial functioning, executive function, language, and/or memory.[20,21] However, whether PDD and DLB actually represent separate disorders is under debate.[20] Thus, in the present article, PDD and DLB will be discussed together.

Huntington's disease (HD) is an autosomal dominant inherited neurodegenerative condition caused by trinucleotide repeates (CAG) in the gene coding for the protein huntingtin (*HTT*). Pathological features include progressive degeneration of striatal GABAergic interneurons.[2,22] Clinical symptoms of HD include motor symptoms, such as chorea, bradykinesia, dystonia, and incoordination, and cognitive symptoms, including changes in visuomotor function, executive function, and memory.[22] Because HD is an autosomal dominant disorder, prodromal phases of this disease can be studied (i.e., prior to clinical onset in mutation carriers) to assess disease development and progression.

Multiple sclerosis (MS) is a neurodegenerative condition featuring degeneration of the myelin sheaths that surround neuronal axons, which results in significant impairment in neuronal transmission.[23,24] Although the exact cause of MS is unknown, it is thought to be the result of either an autoimmune syndrome in which inflammatory cells attack the myelin or a dysfunction of the myelin-producing cells.[25] Multiple sclerosis typically presents either as discrete attacks (relapsing-remitting) or progressive over time (progressive MS).[26] Symptoms of MS can vary dramatically, as MS lesions can occur throughout the cortical white matter, but the most common are autonomic, visual, motor, and sensory problems. [24] Cognitive symptoms usually include behavioral and emotional changes (i.e., depression), as well as impairments in executive functioning, attention, and memory.[24]

HIV-associated neurocognitive disorders (HAND) can be classified into three types based on severity: (1) asymptomatic neurocognitive impairment (ANI), which features cognitive impairment 1 SD below age and education adjusted norms in two cognitive domains but no functional impairment; (2) HIV-associated mild neurocognitive disorder (HMD; also referred to as mild cognitive motor dysfunction [MCMD]), which features cognitive impairment 1 SD below adjusted norms in two cognitive domains and mild impairment in daily functioning; (3) HIV-associated dementia (HAD; also known as AIDS dementia complex [ADC]), which is characterized by cognitive impairment 2 SD or more below age-

NIH-PA Author Manuscript
NIH-PA Author Manuscript
NIH-PA Author Manuscript

and education-adjusted norms in at least two cognitive domains and significant impairment in daily functioning.[27] In the present review, we will combine these three severity categories into one group (HAND). Although these classifications may represent stages of disease, further study is needed for this determination. Approximately 22 to 55% of patients with acquired immunodeficiency syndrome (AIDS) show cognitive dysfunction. Symptoms include disorientation, mood disturbances, and impairment in executive function, speed of information processing, attention and working memory, motor speed, and new learning and retrieval.[28–31] However, long-term and semantic memory, language, and visuospatial function remain relatively intact.[31] Some patients also show motor symptoms.[29] However, symptoms can vary significantly across individuals.[31]

Prion-associated diseases are rare neurodegenerative disorders caused by abnormal processing of the prion protein, which leads to lethal transmissible spongiform encephalopathies (TSEs).[1] Prion-associated diseases can either be sporadic (sporadic Creutzfeldt-Jakob disease [sCJD]; sporadic fatal insomnia [SFI]), genetic (genetic CJD; Gerstmann-Straussler-Scheinker diseases [GSS]; fatal familial insomnia [FFI]), or acquired through infectious transmission of tissue carrying the misfolded prion protein (Kuru; iatrogenic CJD [iCJD]; variant CJD [vCJD]).[1] The different variants of prion-associated dementia show somewhat different symptoms, including varying rates of progression and ages of onset, but the majority feature significant motor and sensory dysfunction, cognitive impairment, and personality changes or psychiatric disorders.[1]

Neuroimaging Biomarkers

The two types of neuroimaging most commonly used as biomarkers of neurodegeneration and dementia include magnetic resonance imaging (MRI) and radionucleotide imaging (i.e., single-photon emission computerized tomography [SPECT], positron emission tomography [PET]). The most widely used neuroimaging technique to investigate anatomical changes and neurodegeneration *in vivo* is structural MRI, which can assess global and local atrophic brain changes. More advanced structural MRI techniques, including diffusion weighted and diffusion tensor imaging [DWI/DTI], magnetic resonance spectroscopy [MRS], and perfusion imaging are also used for investigation of dementia often in a research context. DWI/DTI techniques measure the integrity of tissue using primarily two types of measures, fractional anisotropy (FA) and mean diffusivity (MD) or apparent diffusion coefficient (ADC). Reduced FA and increased MD/ADC are considered to be markers of neuronal fiber loss and reduced gray matter and white matter integrity. MRS is a noninvasive neurochemical technique allowing the measurement of biological metabolites in target tissue that has been used in studies of brain aging, neurodegeneration, and dementia. Two major metabolites that often show alterations in patients with dementia include: (1) N-acetylaspartate (NAA), a marker of neuronal integrity; and (2) myo-inositol (mIns), a measure of glial cell proliferation and neuronal damage. However, other MRS analyte signals can also provide information related to membrane integrity and metabolism. Cerebral perfusion is also commonly measured in studies of neurodegeneration and dementia, including with MRI using either dynamic susceptibility contrast enhanced MRI or arterial spin labeling (ASL),[32,33] or using SPECT or PET techniques (discussed below). MRI can also be used to measure brain function. Functional MRI (fMRI) measures brain activity during a cognitive, sensory, or motor task or at rest by measuring blood flow and blood oxygen levels. The primary outcome measured inmost fMRI studies is blood oxygenation level dependent (BOLD) contrast signal in which regional brain activity is measured via changes in local blood flow and oxygenation.[34] Under normal conditions activity-related brain metabolism is tightly coupled to regional blood oxygenation and flow (i.e., blood flow increases to keep the regional blood oxygen level high during brain activation and associated increases metabolic demand). Therefore, the BOLD signal is a

NIH-PA Author Manuscript NIH-PA Author Manuscript NIH-PA Author Manuscript

useful measure for brain activation.[35] However, altered coupling of neuronal metabolism and blood flow due to brain atrophy and/or hypoperfusion may cause alterations in the BOLD signal. Therefore, fMRI studies in older and demented patient populations with brain atrophy should be carefully evaluated and interpreted with these considerations in mind. fMRI studies often evaluate brain activity during cognitive or functional motor tests. In addition to estimates of regional task-related brain activity, quantification of brain networks can provide a unique measure of brain activity. Techniques for quantifying brain connectivity from fMRI data have recently been developed and applied in studies of brain aging during functional activation (i.e., during performance of tasks), as well as during a "resting" or "task free" state.[36]

SPECT and PET use radiolabeled ligands to measure perfusion, metabolic, and neurochemical processes *in vivo*. SPECT is primarily used to evaluate brain perfusion in studies of neurodegeneration and dementia. Multiple types of PET ligands have been utilized in studies of dementia, including: (1) [18F]fluorodeoxyglucose (FDG), which measures brain glucose metabolism; (2) tracers that assess brain protein deposits, most commonly to measure amyloid deposition (e.g., [11C]Pittsburgh Compound B (PiB), [18F]florbetapir, others); (3) tracers that assess neurotransmitter systems (i.e., dopamine, serotonin, acetylcholine [ACh], etc.) by binding to neurotransmitter receptors, neurotransmitter transporters, or other associated proteins (e.g., catabolic or metabolic enzymes); and (4) tracers that measure the level of activated microglia (e.g., [11C]PK11195, [11C]DAA1106, [11C]PBR28, others). PET studies allow for an assessment of functional changes in brain metabolism and neurotransmitter and other protein levels, which can provide important information about degenerative changes occurring in the brains of patients.

Neuroimaging Biomarkers of Degenerative Diseases and Dementias

Alzheimer's Disease and Prodromal Stages

The most widely used neuroimaging technique to investigate structural changes and neurodegeneration in AD is structural MRI. MRI estimates of regional volumes, extracted using either manual or automated techniques, as well as global and regional tissue morphometry, show the presence of significant brain atrophy in AD patients, following an anatomical distribution similar to the stage-specific neuropathological pattern reported by Braak and Braak.[9] Several structural MRI studies have investigated atrophy in AD and found a pattern of widespread atrophy, including in the MTL and lateral temporal lobe (LTL), medial and lateral parietal lobe, and the frontal lobe, with relative sparing of the occipital lobe and sensory-motor cortex (Fig. 1A, Fig. 2A).[37–39] MCI patients have been shown to have intermediate atrophy between AD patients and healthy older controls (HC), supporting this as an intermediate clinical stage between healthy aging and AD.[40] MCI patients tend to have more focal reductions in volume and gray matter density than AD patients, particularly in the more clinically mild patients, in the entorhinal cortex and hippocampus, as well as focal cortical atrophy particularly in the temporal, parietal, and frontal lobes (Fig. 1A).[41–43] MRI measures of volume, morphometry, and rates of brain atrophy have also shown promise in predicting MCI to AD progression, with significantly reduced hippocampal and entorhinal cortex volumes, as well as reduced cortical thickness in the medial and lateral temporal cortex, parietal lobes, and frontal lobes, in patients destined to convert from MCI to probable AD (MCI-converters), up to 2 years prior to clinical conversion, relative to MCI patients that remain at a diagnosis of MCI (MCI-stable).[39,44–46] Longitudinal studies have shown higher rates of cortical atrophy in patients with AD and MCI, particularly in the temporal lobe. Patients with AD have an approximate annual hippocampal decline of −4.5%, while MCI patients have an annual rate of hippocampal decline of −3%, relative to only an approximate −1% annual change in HC (for a meta-

NIH-PA Author Manuscript NIH-PA Author Manuscript NIH-PA Author Manuscript

analysis, see Barnes et al[47]). Cognitively normal older adults at risk for progression to dementia, due to the presence of cerebral amyloid, genetic background, or the presence of subjective cognitive decline, also show notable brain atrophy and increased atrophy rates, particularly in regions of the MTL.[48–57]

Advanced MRI techniques have also been used in studies of patients with AD, MCI, and older adults at risk for AD. DWI/ DTI studies have indicated that AD patients have reduced FA and increased diffusion relative to HCs in many white matter structures throughout the brain, with MCI patients showing intermediate changes[58–61]. Furthermore, DTI measures showed significant white matter changes in older adults at risk for dementia due to subjective cognitive decline relative to those without significant complaints.[62] MRS techniques demonstrated that AD patients have decreased NAA levels and increased mIns relative to HCs throughout the brain, with the most significant changes in the temporal lobe and hippocampus. [63,64] MCI patients have also been shown to have reductions in NAA relative to HC,[63,65] although NAA values tend to be intermediate between those seen in AD and HC participants. Studies of brain perfusion with MRI have consistently demonstrated decreased perfusion or "hypoperfusion" in patients with AD, particularly in temporoparietal regions, as well as frontal, parietal, and temporal cortices,[66] whereas MCI patients showed decreased brain perfusion in the medial and inferior parietal lobes.[32]

Results from fMRI studies in AD and MCI patients have shown conflicting results. Most studies with AD patients have shown decreased or even absent activation relative to HCs in the MTL, posterior cingulate, parietal lobe, and frontal lobe during episodic memory encoding and recall tasks.[67,68] Furthermore, some studies in MCI patients have shown decreased activation relative to HC during episodic memory encoding[67,69,70] and recall tasks.[67,69,70] However, other studies in both AD and MCI showed increased activation during cognitive tasks.[67,68,70–72] Interestingly, the level of disease severity of patient populations may explain some of these conflicting findings. Increased activation may represent a compensatory mechanism engaged to assist with successful completion of the task in less impaired patients (particularly those with MCI), while more impaired patients, especially those with advanced atrophy, show decreased activation during tasks.[67,68,73] Patients at risk for progression to AD due to genetic background also show altered hippocampal activation during episodic encoding and recall, as well as altered activation during working memory tasks.[74–77]

Functional connectivity studies have also demonstrated alterations in patients with AD and MCI, including decreased connectivity in task-related and resting-state networks. [67,78,79] In particular, a network of brain regions that are deactivated upon task initiation that includes the medial parietal lobe, MTL, and medial frontal lobe, which is referred to as the default mode network (DMN),[36,80] shows decreased activity at rest, decreased connectivity, and reduced deactivation upon task initiation in AD and MCI patients.[67,78,80,81] However, similar to the task-related fMRI studies, mildly impaired MCI patients actually show increased functional connectivity between the memory network and the DMN, suggesting compensatory changes,[67,78,82] while more impaired MCI patients have decreased or absent connectivity between these networks.[67] In addition, older adults at risk for AD show changes in task-related connectivity, as well as altered resting-state connectivity in the DMN.[83–86]

FDG PET studies of patients with AD have shown significant reductions in cerebral glucose metabolism relative to HC, with MCI patients showing intermediate changes, in the temporoparietal cortex, posterior cingulate, parietal lobe, temporal lobe, and in the MTL, including the hippocampus (Fig. 1B).[80,87,88] More impaired AD patients also have more hypometabolism in the frontal lobe and prefrontal cortex relative to less impaired patients

NIH-PA Author Manuscript NIH-PA Author Manuscript NIH-PA Author Manuscript

and HCs.[88,89] Longitudinal studies demonstrated a significantly greater rate of annual decline in metabolism in the temporal, parietal. and frontal lobes, as well as the posterior cingulate and precuneus in AD and MCI relative toHC.[90,91] Cognitively healthy older adults at risk for progression to AD due to genetic background and the presence of subjective cognitive decline also show alterations in glucose metabolism.[91–93]

PET imaging studies with tracers that bind to cerebral amyloid (most commonly [11C]PiB) have shown increased uptake in patients with AD and MCI in brain regions known to show amyloid deposition in neuropathological studies, including the frontal, temporal, and parietal lobes, posterior cingulate, and precuneus (Fig. 1C).[54,94,95] Across [11C]PiB studies, 96% of AD patients showed significant amyloid accumulation, measured as a "positive" [11C]PiB signal,[96] while nearly two-thirds of patients with MCI showed significant amyloid accumulation.[96] In addition, MCI patients with significant amyloid accumulation have a higher likelihood of future conversion to AD.[97] Longitudinal assessments of amyloid using [11C]PiB in AD and MCI patients have shown minimal increases in [11C]PiB signal over 1 to 2 years in patients who showed significant [11C]PiB signal at baseline.[95,98] However, in patients who do not show significant amyloid deposition at baseline, additional amyloid accumulation may be possible. Thus, researchers have tentatively concluded that amyloid deposition occurs early in the disease and by the time sufficient cognitive decline for a diagnosis of AD occurs, brain amyloid burden is relatively stable and increased deposition is minimal. Finally, patients at risk for progression to AD due to genetic background also show higher amyloid accumulation. [99–102] Given the results of amyloid PET studies to date, it is noteworthy that in 2011 the United States Food and Drug Administration approved [18F]Florbetapir (Amyvid, Eli Lilly & Co., Indianapolis, IN) for clinical assessment of cerebral amyloid in the context of cognitive decline.[103]

In addition to evaluating cerebral metabolism and the presence of amyloid, researchers have investigated specific alterations in neurotransmitter systems and neuroinflammation in AD and MCI patients using PET. Using PET techniques with tracers specific for acetylcholinesterase (AChE) as a surrogate measure for ACh synaptic density, significant reductions in binding were found in AD and MCI, particularly in the temporal lobe.[104–106] Studies in AD patients have also shown decreases in GABA, serotonin, and dopamine synaptic densities,[107] whereas MCI patients have been shown to have deficits in serotonergic neurotransmission only.[108] Studies of activated microglia have shown mixed results in patients with MCI and AD. Some studies demonstrated significantly elevated global and regional activated microglia in patients with AD relative to HCs,[109,110] while other studies have shown minimal signal in AD and MCI relative to HCs.[111] These differences likely reflect small samples and conflicting quantification methodologies. Future studies are needed to elucidate the role of activated microglia in AD and MCI, as well as utility of this class of PET tracers as a biomarker of immune status in neurodegenerative disorders.

Overall, neuroimaging studies have been useful for quantifying ongoing neuropathological changes in patients with AD, as well as in the prodromal stages of disease. Measures of brain atrophy, brain function and connectivity, brain perfusion and metabolism, and levels of amyloid have shown progressive changes associated with the development and progression of AD. Future studies utilizing newer techniques and in less-affected patient populations will be important for further understanding AD pathology, early disease detection, and the development of targeted therapies.

Familial and Atypical Alzheimer's Disease

Neuroimaging studies in familial AD patients (i.e., those with mutations in *APP*, *PS1*, or *PS2*) have shown greater brain atrophy, faster longitudinal atrophy rates, white matter

NIH-PA Author Manuscript
NIH-PA Author Manuscript
NIH-PA Author Manuscript

changes measured using DTI, reduced brain metabolism, and increased brain amyloid, in affected patients and in presymptomatic mutation carriers relative to noncarriers.[10,112–116] Overall, the use of biomarkers in the study of familial AD has shown similar neuropathological changes as seen in late-onset AD, in both presymptomatic and symptomatic familial AD patients. Studies in these patients may provide information relevant to the role of biomarkers for late-onset AD, as well as to provide sensitive measures for detecting disease related changes and monitoring disease progression in patients with familial AD. However, it is also noteworthy that in some cases the profile of biomarkers in familial AD patients can differ from that observed in late-onset AD. For example, some familial AD patients may show amyloid deposition in the striatum, a finding which is not often observed in late-onset AD.[117] Future studies to explore the similarities and differences in familial and sporadic AD pathology will provide important information, such those associated with the Dominantly Inherited Alzheimer Network (DIAN).[10]

Sporadic AD usually presents with changes in memory. However, a few related disorders have been identified with atypical presentations (atypical AD), including PCA and log-openic aphasia. Both diseases show widespread amyloid deposition and neurofibrillary tangles, which supports the theory that these disorders are AD dementias despite their atypical clinical presentation. Neuroimaging studies of PCA have demonstrated notable atrophy in posterior brain regions, including in the posterior temporal, parietal, and occipital lobes (Fig. 2B).[11,118,119] A DTI study of white matter integrity also showed notable atrophy of the ventral visual processing stream, with reduced FA in the bilateral inferior longitudinal fasciculus and inferior fronto-occipital fasciculus.[118] Patients with PCA have also been shown to have severe hypoperfusion in occipitoparietal regions, but increased perfusion in frontal, anterior cingulate, and mesiotemporal regions.[120,121] Finally, PCA patients show positive binding of [11C]PiB with a traditional AD-like pattern, except for more signal than AD patients in the occipital lobe.[122]

Structural MRI studies in logopenic aphasia have shown significant degeneration of the left posterior superior temporal lobe, temporoparietal junction, inferior parietal lobe, posterior cingulate, precuneus, and MTL (Fig. 2C). In more severe patients, atrophy was also observed in left anterior temporal lobe regions, along the sylvian fissure and into the frontal lobe, as well as in regions of the right temporal and parietal lobes.[13,16,123] DTI studies in logopenic aphasia have also shown atrophic changes, including reduced white matter integrity in the left temporoparietal junction and bilateral (but left > right) inferior longitudinal fasciculus, uncinate fasciculus, superior longitudinal fasciculus, and other subcortical projections.[124,125] SPECT and FDG PET studies have shown reduced perfusion and brain metabolism in the left temporoparietal lobe, respectively.[120,123,126] In addition, a recent study demonstrated increased [11C]PiB uptake in patients with logopenic aphasia, suggesting the presence of significant cerebral amyloid.[126]

Cerebral amyloid angiopathy is primarily characterized by vascular pathology on structural imaging. Patients with CAA typically show cerebral microhemorrhages, often at the cortical gray matter/white matter interface and/or in cortico-subcortical junctions of the frontomesial, fronto-orbital, and parietal lobes, microbleeds found predominately in posterior cortical regions, and other ischemic related changes (i.e., white matter lesions and infarcts).[14,120,127,128] A functional MRI study of CAA patients also demonstrated altered vascular function, including reduced vascular reactivity to visual stimulation in the presence of normal blood flow.[129] Studies with SPECT imaging showed hypoperfusion in parietal, temporal, and frontal lobes in patients with CAA.[130] Finally, a PET study with [11C]PiB in patients with CAA demonstrated significant tracer uptake, supporting the presence of extensive cerebral amyloid deposition.[131]

NIH-PA Author Manuscript NIH-PA Author Manuscript NIH-PA Author Manuscript

Vascular Cognitive Impairment and Dementia

A few studies have evaluated the extent of brain structural and functional changes in vascular dementia and VCI using *in vivo* neuroimaging techniques. Although the definitions of vascular dementia and VCI vary significantly across studies, samples of patients with subcortical ischemic vascular dementia (SIVD) and leukoaraiosis, which is extensive white matter pathology identified using MRI, are most commonly evaluated. Several studies have investigated patients with SIVD and other patients with vascular dementia using structural MRI techniques and shown that SIVD patients and patients with leukoaraiosis show greater number of white matter lesion than cognitively healthy older adults without subcortical infarcts and patients with AD.[132–137] The presence of more white matter lesions is also significantly associated with impaired cognition, particularly in executive function and processing speed domains, as well as a greater dementia severity and the presence of cognitive complaints. [136,138–140] Patients with SIVD and leukoaraiosis also show significant gray matter, white matter, and hippocampal atrophy relative to HCs,[132,134,135,137,141–144] which has also been linked to the extent of white matter lesion pathology. [133–136,145,146] Only a limited number of studies have investigated structural MRI changes in patients in earlier stages of vascular dementia, such as vascular-related MCI.[133,134,136,143] Seo and colleagues reported cortical thinning in patients with MCI linked to subcortical ischemia, particularly in frontal, temporal, and occipital regions.[143] Patients with vascular-associated MCI also show a significantly greater extent of white matter lesions than HC, the presence of which is associated with progression to dementia.[133] Studies utilizing DTI have demonstrated significant changes in SIVD and leukoaraiosis patients, even in normal-appearing white matter.[147–154] In fact, DTI measures of decreased white matter integrity have shown significant association with dementia severity, cognition, motor function, and cerebral atrophy.[147,148,150–154] A few studies have also evaluated fMRI measures in patients with vascular dementia, in particular SIVD. Two studies evaluated task-related fMRI in SIVD patients and demonstrated reduced activation and altered brain blood flow-metabolic coupling during an executive function and motor task, respectively.[155,156] Finally, a study by Sun and colleagues showed altered posterior cingulate cortex functional connectivity in SIVD patients using resting-state fMRI.[157] Schuff and colleagues assessed brain perfusion in SIVD using ASL and demonstrated reduced cerebral blood flow, particularly in frontal and parietal lobes.[158] These results support previous studies utilizing PET and SPECT techniques, which showed reduced cerebral perfusion and metabolism in patients with vascular dementia.[159,160] In fact, FDG PET studies have shown hypometabolism in a scattered pattern in cortical and subcortical regions in vascular dementia. [161] Finally, amyloid PET tracers show minimal binding in the majority of patients with vascular dementia in the absence of CAA.[162]

Neuroimaging studies in vascular dementia have demonstrated notable changes in brain atrophy, function, perfusion and metabolism secondary to vascular pathology. Prospective studies evaluating patients in earlier stages of disease would be useful to identify the progressive changes associated with the development of vascular dementia, as well as the effect of any interventional treatments. In addition, studies of patients with vascular pathology and other types of comorbid pathology (AD, FTD, etc.) will provide the opportunity to assess the overlap of multiple diseases and the relative contribution of various pathologies to cognitive decline.

Frontotemporal Dementia

Behavioral variant FTD is characterized primarily by changes in personality and behavior and is caused by accumulation of pathological tau protein or TDP-43 or in rare cases by changes in the fused in sarcoma (FUS) protein.[13,16,17] Genetic forms of bvFTD can be linked to mutations in the tau gene (*MAPT*), which results in tau pathology, the progranulin

NIH-PA Author Manuscript
NIH-PA Author Manuscript
NIH-PA Author Manuscript

gene (*GRN*), which results in TDP-43 pathology, as well as several other genes.[13,16,17] Generally, bvFTD patients show widespread atrophy in the frontal lobes, anterior cingulate, anterior insula, and thalamus (Fig. 3B).[13,120,163,164] Longitudinally, faster atrophy rates are observed in the frontal lobes.[120,165] Some differences in atrophy are observed in bvFTD based on underlying pathology. bvFTD due to Pick's disease shows atrophy in the prefrontal cortex, temporal lobes, anterior cingulate and insula, which is typically bilateral but with slightly greater atrophy on the left than right.[17,166] The frontal atrophy in bvFTD patients with Pick's disease is usually greater than that seen in other bvFTD forms, such as CBD, patients with *MAPT* mutations, and those with underlying TDP-43 pathology.[17,167] Patients with *MAPT* mutations tend to be a heterogeneous group with atrophy observed in the frontal and temporal lobes, insula, anterior cingulate, parietal lobe, basal ganglia, and brainstem.[17,168] Furthermore, patients with *MAPT* mutations may show more temporal lobe atrophy than other bvFTD forms.[13,169] Patients with bvFTD with TDP-43 pathology show widespread frontal, temporal, and parietal atrophy, which tends to be asymmetric but either side can show predominance.[17,166,170,171] The parietal atrophy tends to be more severe in patients with TDP-43 bvFTD variants than those caused by tau pathology.[17] Patients with mutations in the *GRN* gene show a similar pattern of frontal, temporal, and parietal atrophy, but may show a greater asymmetry than bvFTD patients with TDP-43 who do not have a *GRN* mutation.[172] Finally, bvFTD patients with underlying FUS pathology show a unique pattern of severe caudate atrophy, along with similar frontal atrophy to that seen in the other bvFTD forms.[17,173] DTI studies of white matter integrity in bvFTD have demonstrated reduced FA in frontal and temporal white matter, including in the uncinate fasciculus, anterior cingulum, superior longitudinal fasciculus, and inferior longitudinal fasciculus relative to HC.[128,174,175] Patients with bvFTD show greater frontal lobe white matter changes than AD patients, including in the anterior cingulum, anterior corpus callosum, and uncinate fasciculus.[175,176] DTI studies in bvFTD patients with *MAPT* and *GRN* mutations have also shown reduced white matter integrity throughout the frontotemporal white matter.[177]

Studies of task-related fMRI activation in bvFTD have shown altered activation patterns during working memory and emotional processing tasks, including reduced frontal and parietal activation during working memory[178] and emotion-specific abnormalities in frontal and limbic regions, as well as altered activation in posterior regions (i.e., fusiform gyrus, inferior parietal cortex) during an implicit face-expression task.[179] Resting-state fMRI has also demonstrated altered functional connectivity in patients with bvFTD, particularly in the salience network, which is a network of regions involved in filtering sensory and emotional stimuli and directed attention that includes the anterior cingulate cortex, bilateral insula, dorsolateral prefrontal cortex, supplementary motor area, and other temporal, frontal, and parietal cortical regions.[180] Patients with bvFTD show decreased connectivity in the dorsal and ventral salience network, including in the basal ganglia and frontal lobe, but increased connectivity in the precuneus relative to HC.[181–183] Relative to AD patients, bvFTD patients show an opposite pattern of functional connectivity, with decreased connectivity in the salience network and increased connectivity in the DMN.[181,184] Alterations in connectivity of other regions has also been reported, including in an attention/working memory network, which showed reduced connectivity with the DMN, and an executive network, as well as in cingulate and frontal white matter regions.[177,182] Patients with *MAPT* mutations also show alterations in connectivity of the DMN, with increased connectivity in the medial parietal lobe and reduced connectivity in the lateral temporal and medial prefrontal cortices.[185] Patients with bvFTD showed reduced cerebral perfusion, primarily in frontal and temporal lobes, in studies utilizing both SPECT and ASL techniques. [120,128,163,164,174,186] FDG PET studies of brain metabolism in bvFTD have also demonstrated notable hypometabolism in frontal and temporal regions. [120,128,163,164] Studies with amyloid tracers (i.e., [11C] PiB) showed minimal binding in patients with bvFTD.[131]

NIH-PA Author Manuscript NIH-PA Author Manuscript NIH-PA Author Manuscript

The semantic variant of primary progressive aphasia (PPA), semantic dementia (SD), features language difficulties with fluency, anomia, and single-word comprehension and is most commonly associated with TDP-43 pathology. Patients with SD show asymmetrical atrophy of the temporal lobes, most commonly left > right, particularly in anterior and inferior temporal lobe regions, including the temporal pole, perirhinal cortex, anterior fusiform, hippocampus, and amygdala (Fig. 3A).[13,16,187–189] More severe patients may also show atrophy in parts of the superior and posterior left temporal lobe, regions of the left frontal lobe, left insula, and left anterior cingulate, as well as increasing atrophy in the right temporal lobe.[13,190] Longitudinally, SD patients show progressive atrophy of the left temporal lobe, followed by the right temporal lobe.[13,191] DTI techniques have shown reduced white matter integrity in bilateral temporal lobes (left > right), including in the inferior longitudinal fasciculus, left parahippocampal white matter, and in the uncinate fasciculus, with the lowest FA values seen in the left anterior temporal lobe.[125,174,175] fMRI studies of SD patients have shown altered activation patterns during a variety of tasks, including during sound processing, autobiographical memory, and surface dyslexia.[192–194] Resting-state functional connectivity studies have also shown decreased connectivity of frontotemporal and frontolimbic circuitry, but increased connectivity in local networks of the prefrontal cortex in SD patients relative to HC.[195] SPECT and PET studies of SD patients demonstrated reduced perfusion and metabolism primarily in the left anterior temporal lobe,[13,120,126] while a study with [11C]PiB showed minimal binding.[126]

The nonfluent variant of PPA, progressive nonfluent aphasia (PNFA), is more heterogeneous than SD featuring speech production impairment with agrammatism, phonemic errors, anomia, sentence comprehension impairment, and potentially apraxia of speech. Progressive nonfluent aphasia can be caused by either tau or TDP-43 pathology, the latter of which does not show apraxia of speech.[13] Patients with PNFA show atrophy primarily in anterior perisylvian regions, including in the left inferior frontal lobe, insula, and premotor cortex, with further involvement of other frontal lobe regions, the temporal and parietal lobes, as well as the caudate and thalamus in later disease stages (Fig. 3C).[13,16,190,196,197] Interestingly, PNFA patients with underlying Pick's disease (tau) pathology have more severe temporal lobe atrophy than other forms, while those with a *GRN* mutation (TDP-43 pathology) show notable atrophy in the left lateral temporal lobe.[17,196] DTI studies in PNFA patients demonstrated moderate decreases in white matter integrity relative to HC in the left arcuate fasciculus, most especially in the frontoparietal component, in the superior motor pathway, and in left perisylvian, inferior frontal, insular, and supplemental motor area regions.[125,174,175] A study utilizing fMRI in PNFA patients demonstrated reduced activation in the left inferior frontal lobe during sentence reading and comprehension relative to HC.[198] FDG PET studies have demonstrated hypometabolism in left inferior frontal gyrus, frontal operculum, insula, premotor cortex, and supplementary motor area in PNFA patients. [11,199,200] Studies with [11C]PiB showed minimal binding in patients with PNFA, however, some signal was observed in those with underlying Pick's disease pathology. [126,199] Finally, PNFA patients show reduced striatal dopaminergic signal with a tracer targeting pre-synaptic dopaminergic transporters.[201]

Frontotemporal dementia with motor symptoms has multiple forms, including CBD, PSP, FTD with motor neuron disease (FTD-MND), and FTD with ALS (FTD-ALS). These diseases can present with behavioral or language symptoms (typically PNFA), but usually they present with behavioral symptoms. However, all of these disorders also feature motor dysfunction. Corticobasal degeneration and PSP are caused by tau pathology, while FTD-MND and FTD-ALS are associated with TDP-43 pathology. Structural imaging studies in CBD and PSP have shown significant atrophy in the posterior frontal cortex in both disorders, with more atrophy in the basal ganglia and faster longitudinal decline in whole brain volume in CBD than PSP.[17,166,202,203] On the other hand, PSP may show more

NIH-PA Author Manuscript NIH-PA Author Manuscript NIH-PA Author Manuscript

atrophy in the posterior frontal lobe white matter, brainstem, cerebellum, and midbrain than CBD.[17,202] Atrophy in CBD is also typically asymmetrical, while atrophy in PSP is usually symmetrical.[17,204] DTI studies in CBD demonstrated a loss of white matter integrity in the motor thalamus, precentral and postcentral gyri, and bilateral supplementary motor area, while PSP patients showed decreased white matter integrity in the anterior part of the thalamus, cingulum, primary and supplementary motor areas, and frontoorbital white matter.[205] ASL studies in CBD have also shown reduced cerebral perfusion in the right hemisphere.[206] SPECT studies have demonstrated reductions in neurotransmitters in both CBD and PSP, with reduced dopaminergic transporter binding in the striatum and reduced acetylcholine transporter binding in the anterior cingulate and thalamus relative to HC.[207,208] FDG PET studies in CBD and PSP also showed cerebral hypometabolism, with reduced metabolism in cortical regions contralateral to the physically affected side in CBD and hypometabolism in the prefrontal cortex, caudate, thalamus, and mesencephalon in PSP.[209]

FTD-MND and FTD-ALS are both primarily linked to TDP-43 pathology (although a few FTD-MND patients may show FUS pathology) and feature behavioral or language deficits along with motor dysfunction. Patients with FTD-MND or FTD-ALS show frontal and temporal lobe atrophy, in addition to atrophy in the anterior cingulate, occipital lobe, and precentral gyrus in FTD-ALS only.[13,17,19,170,171,210] DTI studies have shown decreased white matter integrity relative to HC in frontal and temporal regions, including the corpus callosum, corticospinal tract, cingulum, inferior longitudinal fasciculus, inferior fronto-occipital fasciculus, and uncinate fasciculus, which was associated with poorer performance on cognitive tasks.[211–214] Task-related and resting-state fMRI and functional connectivity studies have also shown alterations in brain function and connectivity in patients with FTD-ALS. Reduced activation in FTD-ALS patients measured using PET and fMRI was observed in the frontal lobe, insula, and thalamus during an executive task and in the frontal lobe, anterior cingulate, supramarginal gyrus, temporal lobe, and occipitotemporal regions during a verbal fluency task.[19,215–217] Reduced frontal activation during an emotional task was also observed in nondemented FTD-ALS patients.[211,218] Reorganization of motor networks and decreased functional connectivity of a sensorimotor network, the DMN, and a frontoparietal network were also seen in resting-state studies of FTD-ALS patients.[19,219] Patients with FTD-MND demonstrated reduced perfusion in SPECT studies in the frontal lobe, including the premotor cortex and precentral gyrus, as well as the temporal lobe, cingulate, insula, thalamus, and striatum. [220] Patients with FTD-ALS also show hypoperfusion in similar areas of the frontal and temporal lobes, which correlates with impaired cognition.[19,211,221,222] FDG PET studies in FTD-MND patients demonstrated reduced metabolism in the frontal, anterior and medial temporal lobe, basal ganglia, and thalamus (Fig. 4),[223,224] whereas patients with FTD-ALS show hypometabolism in the frontal lobe, superior occipital lobe, and thalamus.[19,211,225] Patients with FTD-ALS also show reduced serotonin binding in the frontal lobe, as well as a reduced number of GABA-A receptors in the frontal lobe, superior temporal lobe, parietal lobe, occipital lobe, and insula.[19,211,226] Some forms of FTD-MND and FTD-ALS are caused by genetic mutations in chromosome 9 (*C9ORF72*) or *GRN*.[17,18] Patients with FTD-MND carrying a mutation in chromosome 9 have more thalamic atrophy than those with FTD-MND without the chromosome 9 mutation, as well as greater frontal lobe, temporal lobe, insular, and posterior cortical atrophy than seen in FTD patients with other mutations.[227,228]

In sum, neuroimaging studies in FTD have been useful for identifying and quantifying structural and functional changes in the brain during disease, including frontal and temporal atrophy, altered brain function and connectivity, reduced cerebral perfusion and metabolism, and changes in neurotransmission. However, additional studies in larger cohorts to better

NIH-PA Author Manuscript

NIH-PA Author Manuscript

NIH-PA Author Manuscript

characterize and differentiate the various FTD subtypes, as well as the overlap between FTD and ALS, are needed.

Amyotrophic Lateral Sclerosis

Amyotrophic lateral sclerosis (ALS) is a progressive degenerative motor disease that includes cognitive changes in up to 63% of patients (FTD-ALS, see above section).[19] However, patients with ALS without cognitive symptoms also show structural and functional changes in the brain, although usually these changes are less severe than those in ALS patients with cognitive decline.[19] Patients with ALS show progressive atrophy in motor and extramotor regions, most especially in the precentral gyrus.[211,229–231] DTI studies demonstrated widespread loss of white matter integrity, including in the corticospinal tract, the posterior limb of the internal capsule, cingulum, midposterior corpus callosum, and in frontal and temporal white matter tracts, such as the uncinate fasciculus, inferior longitudinal fasciculus, and inferior fronto-occipital white matter.[211–213,232–234] Functional MRI studies have shown altered brain activation in ALS patients during motor tasks, including increased activation in motor and premotor areas, the supplementary motor area, inferior parietal lobes, superior temporal lobes, and cerebellum during movement and increased activation in basal ganglia, cerebellum, and brainstem during motor learning. [211,235–238] During a sensory task, patients with ALS had reduced activity in primary and secondary sensory areas but increased activation in associative sensory areas.[211,239] Altered activation during emotional processing in nondemented ALS patients was also seen, with increased activation in the left hemisphere but reduced activation in the right frontal lobe.[218] Changes in functional connectivity in patients with ALS have also been observed. Studies have found mixed findings, with decreased connectivity of a sensorimotor network, the DMN, and an interhemispheric motor network seen in some studies but increased connectivity in sensorimotor, premotor, prefrontal cortex, and thalamic networks seen in other studies.[211,219,240,241] MRS studies have shown alterations in patients with ALS, including decreased NAA and increased choline, glutamate, glutamine, and mIns in the corticospinal tract, posterior limb of the internal capsule, and periventricular white matter, as well as a decreased NAA/choline ratio in the thalamus, basal ganglia, middle cingulate, and frontal and parietal lobes.[211,242–245] SPECT and PET studies in ALS have observed reduced cortical perfusion and metabolism, which was associated with reduced cognition even in nondemented ALS patients. [211,246–248] Dopaminergic and GABAergic cell loss in the basal ganglia and substantia nigra has also been reported. [211,226,249] Finally, an increase in binding of a PET tracer that labels activated microglia, [11C]PK-11195, was observed in ALS patients in the corticospinal tract and extramotor regions with the greatest binding observed contralateral to the physically affected side.[211,250,251]

Neuroimaging studies in ALS, with and without concurrent cognitive symptoms, have shown notable changes in brain structure and function likely due to ongoing neurodegeneration. Future studies designed to investigate additional changes in ALS and FTD-ALS patients will help to expand the understanding of these diseases.

Parkinson’s Disease/Dementia with Lewy Bodies

Parkinson’s disease (PD) is a degenerative motor disease that may or may not feature cognitive impairments. However, up to 80% of PD patients will eventually develop cognitive symptoms. [21] Pathological and clinical differences between Parkinson’s disease with dementia (PDD) and dementia with Lewy bodies (DLB) are minimal and subject to debate. Thus, imaging findings in these disorders (PDD/DLB)will be discussed together, followed by a discussion of imaging in PD without dementia. Patients with PDD/DLB show fluctuations in attention, executive function, and higher order visual function, in addition to motor symptoms which are the result of widespread deposition of α-synuclein. Structural

NIH-PA Author Manuscript

NIH-PA Author Manuscript

NIH-PA Author Manuscript

imaging studies have shown widespread atrophy in cortical and subcortical regions in patients with PDD/DLB, including in the temporal, parietal, and frontal lobes, in the MTL (hippocampus, amygdala, entorhinal cortex), basal ganglia, thalamus, hypothalamus, substantia nigra, insula, and occipital lobe.[11,20,21,181,252–257] Although atrophy patterns are similar in PDD and DLB, some studies have suggested increased fronto-temporal atrophy but less caudate atrophy in DLB patients relative to PDD.[20,258,259] In addition, amyloid positive PDD/DLB patients show more cerebral atrophy than PDD/DLB patients who are amyloid negative.[20,258,260] Longitudinally, patients with PDD/DLB show faster rates of cerebral atrophy than PD patients without dementia and HC, particularly in regions of the medial and lateral temporal lobe, as well as occipitotemporal areas.[20,21,203,258,261,262] DTI studies in PDD/DLB demonstrated reduced white matter integrity in the frontal, temporal, and parietal lobes, pons, thalamus, precuneus, caudate, corpus callosum, and inferior longitudinal fasciculus.[20,128,181,263–267] Some studies again showed greater pathology in DLB than PDD, with more reduced FA in the bilateral posterior temporal lobe, posterior cingulate, and bilateral visual association areas in DLB.[176,263] MRS studies in PDD patients have shown reduced NAA/creatinine and glutamine/glutamate ratios in the posterior cingulate and bilateral hippocampus.[20,268–270] Studies of patients with PDD/DLB utilizing fMRI techniques demonstrated reduced activation in the lateral occipitotemporal lobe during visual motion and in the ventral occipitotemporal lobe during face matching, but increased activation in the superior temporal sulcus during the latter task.[20,271] Reduced activation in visual areas was also seen during presentation of a simple visual motion stimuli.[272] Alterations in brain activation during executive function paradigms in patients with PDD/DLB have also been observed, although mixed findings have been reported including increased activation and decreased activation in the prefrontal cortex during various tasks.[252,273] Resting-state functional connectivity studies have also shown changes in brain connectivity in patients with PDD/DLB, including reduced global and local cortico-cortical connectivity.[181] Other studies have shown altered connectivity of the precuneus, with increased connectivity of the precuneus with regions of the dorsal attention network and putamen, but decreased connectivity of the precuneus with the DMN and visual cortices.[274] ASL and SPECT studies have shown reduced cortical perfusion in posterior cortical areas in PDD/DLB patients, including in occipital and temporoparietal regions.[11,129,272,275–277] Hypometabolism has also been reported in FDG PET studies of PDD/DLB patients, particularly in the basal ganglia, cerebellum, and frontal, temporal, parietal, and occipital lobes with relative sparing of metabolism in the MTL.[11,21,128,275,278–280] Furthermore, occipital lobe hypometabolism was associated with visual hallucinations in DLB patients. [253] PET studies with amyloid tracers (i.e., [11C]PiB) have shown positive amyloid binding in ~40% of PDD/DLB patients (50% of DLB, 30% of PDD), with a similar anatomical distribution to the pattern seen in AD patients.[131,260] Reduced dopaminergic transporter binding in the basal ganglia has also been observed in PDD/DLB patients, with decreased binding in the caudate, which is associated with cognitive symptoms, and decreased binding in the putamen, which is associated with motor symptoms (Fig. 5).[21,131,253,258,281,282] Decreased cholinergic neurotransmission has also been seen in patients with PDD/DLB throughout the cortex, particularly in medial occipital and posterior cortical regions, which is more severe than changes seen in PD patients without dementia and AD patients.[21,128,131,258,283–287]

Patients with PD without dementia also show atrophic and functional brain changes, although they tend to be milder than those seen in PDD/DLB patients. Some studies have shown gray matter atrophy in the left anterior cingulate, left gyrus rectus, left parahippocampal gyrus, and right frontal lobe in PD patients, while other studies show minimal or no atrophy.[252] Mild hippocampal atrophy has also been observed, although significantly less atrophy than seen in PDD/DLB and AD.[21,288–290] Further, patients with PD show a slightly faster cortical atrophy rate than HC, particularly in regions of the

NIH-PA Author Manuscript
NIH-PA Author Manuscript
NIH-PA Author Manuscript

cingulate, occipitotemporal lobe, insula, hypothalamus, nucleus accumbens, and hippocampus.[21,291] Studies utilizing PET techniques have most commonly been reported in PD. Using FDG PET, patients with PD showed reduced metabolism in frontal, temporal, parietal, and occipital lobes, as well as in the basal ganglia and thalamus.[21,278,292] Parietal and frontal metabolism also shows longitudinal decreases over time.[21,293] However, PET studies with [11C]PiB have shown no significant binding in PD patients without dementia.[131] PET studies evaluating different neurotransmitter systems have also been widely used in PD patients, including assessments of dopaminergic, serotonergic, cholinergic, GABAergic, and opioid neurotransmission. Reduced dopaminergic neurotransmission in the striatum has been observed in patients with PD, with the most significant changes in the putamen contralateral to the physically affected side (Fig. 5).[2,283,294,295] Early in the disease, increased dopaminergic receptor binding has been observed in the putamen, frontal lobe, anterior cingulate, and globus pallidus.[283,296,297] However, later in the disease course reduced dopaminergic receptor binding is also seen in the thalamus, anterior cingulate, and frontal and temporal lobes.[283,298,299] Reduced serotonergic neurotransmission in the orbitofrontal cortex, caudate, putamen, and midbrain has also been reported in patients with PD.[283,300] Furthermore, ACh neurotransmission is reduced in cortical regions in PD, even early in disease, while increased ACh receptors have been reported in the frontal and temporal lobes.[283,284,287,301] Decreased GABAergic neurotransmission has also been reported, primarily in the pons and putamen,[302] while striatal, thalamic, cingulate, and frontal areas show reduced opioid neurotransmission. [283,303] Finally, increased microglial activation has been observed in patients with PD in both striatal and extrastriatal regions.[283,304,305]

Overall, studies in patients with PD with or without dementia, as well as DLB patients, have shown significant atrophic, functional, and molecular brain changes. Additional studies in early stage PD-related disorders before cognitive changes will help further the understanding of disease development in relation to phenomenology, as well as the potential for neuroimaging biomarkers to be used in clinical assessment and monitoring of treatments.

Huntington's Disease

Huntington's disease (HD) is an autosomal dominantly inherited progressive degenerative disease causing motor and cognitive abnormalities. Progressive reductions in striatal volume can be seen in both presymptomatic (pre-HD) and symptomatic ("manifest") HD patients, even up to 15 to 20 years before the clinical symptoms appear (Fig. 6).[306–310] Atrophy of the putamen is greater than that in the caudate early in the disease and later atrophy expands to the globus pallidus and nucleus accumbens. [306,308,309,311] This striatal atrophy is associated with impaired motor and cognitive function.[308,311] Atrophy is also seen in other gray matter and white matter regions in both pre-HD and manifest HD, including cerebral thinning throughout the cortex, atrophy in the cingulate and thalamus, and atrophy of the white matter tracts near the striatum, as well as the corpus callosum, posterior white matter tract, and frontal lobe white matter.[306,312–314] Subcortical and cortical atrophy, specifically in the left superior frontal gyrus, left inferior parietal lobule, and bilateral caudate, has also been shown to be associated with impaired saccade eye movement.[315] Longitudinally, faster rates of atrophy in the striatum are observed in both pre-HD and manifest HD patients, whereas greater whole brain atrophy rates are observed in manifest HD patients only.[306,310,312] DTI studies have shown reduced white matter integrity in the frontal lobe, precentral gyrus, postcentral gyrus, corpus callosum, anterior and posterior limbs of the internal capsule, putamen, and globus pallidus in patients with pre-HD and manifest HD.[316–318] fMRI studies have demonstrated task-induced activation differences in pre-HD and manifest HD patients in the striatum, cingulate, and premotor regions during several cognitive tasks.[319] Furthermore, decreased activation of the primary motor cortex, medial

NIH-PA Author Manuscript

NIH-PA Author Manuscript

NIH-PA Author Manuscript

premotor regions, prefrontal cortex, and parietal lobe, along with increased insula activation were observed during a motor task.[22] fMRI studies of antisaccade eye movement have also shown altered brain activation in task-related regions.[320] Studies of resting-state connectivity have shown reduced DMN connectivity in the anterior prefrontal cortex, inferior parietal lobe, and posterior cingulate, as well as reduced connectivity between cortical motor regions and the striatum in pre-HD patients.[321,322] Functional connectivity during a working memory task was also shown to be altered in patients with pre-HD in regions of the prefrontal cortex, striatum, and frontoparietal regions.[319] PET studies have also shown alterations in brain metabolism, dopaminergic neurotransmission, and activated microglia in pre-HD and manifest HD patients. Reduced brain metabolism is observed in pre-HD patients, which may be useful for detecting and monitoring disease progression.[2,323,324] Reduced dopaminergic receptor binding was observed in both cross-sectional and longitudinal studies in pre-HD and manifest HD patients.[2,325,326] The longitudinal decline in dopaminergic receptors was also associated with decline in both cognition and motor function.[2,327–329] Studies utilizing [11C]PK-11195 have shown increased activated microglia in the striatum, extrastriatal regions, and the hypothalamus in both pre-HD and manifest HD patients, which correlated with reduced dopaminergic receptor binding, increased motor dysfunction, and predicted time of clinical onset.[2,330–333]

Overall, neuroimaging studies in patients with HD have shown marked changes in brain structure and function, particularly in striatal regions. Alterations observed in pre-HD patients are particularly interesting, as studies in this population allow for exploration of the progression of disease before the onset of clinical symptoms. Future studies exploring neuroimaging measures in both pre-HD and HD patients will assist with better clinical diagnosis, even prior to disease onset, and monitoring of potential therapeutics in the context of early intervention.

Multiple Sclerosis

Multiple sclerosis (MS) features motor, sensory, visual, and autonomic system dysfunction due to progressive lesions in cerebral gray matter and whitematter.[24] Patients can present with various forms including relapsing-remitting MS (RRMS), many of whom later develop secondary progressive MS, primary progressive MS, and clinically isolated MS syndrome. The most commonly reported neuroimaging feature in MS is focal hyperintense white matter lesions on T2-weighted, FLAIR, and contrast-enhanced MRI scans.[334,335] T1-weighted hypointense lesions ("black holes") have also been reported. [336] Patients with MS also show atrophy of the gray matter and white matter, particularly reduced gray matter in the cerebellum, thalamus, subgenual gyrus, middle cingulate cortex, superior frontal lobe, and bilateral temporal and occipital lobes.[334] Gray matter atrophy is predictive of cognitive symptoms and long-term disability.[334,335,337] DTI techniques have also shown damage in normal appearing gray matter and white matter, as well as lesion tissues.[335] Loss of whitematter integrity in the corpus callosum, corona radiata, superior and inferior longitudinal fasciculi, internal and external capsule, posterior thalamic radiations, cerebral peduncles, and superior cerebellar peduncles was observed and correlated with both motor and cognitive symptoms.[334] In normal appearing white matter, widespread abnormalities are observed, even in the earliest stages, which progress as the disease worsens.[338–340] Changes in normal-appearing gray matter appear later in the disease course, with increased diffusivity and increased or decreased FA depending on phase of gray matter inflammation observed in later-stage patients. [338,341,342] In white matter tissue with lesions, significant alterations in white matter integrity are observed in all MS forms except for in primary progressive MS.[338,343,344] However, gray matter lesions actually show an increase in FA, which may reflect more inflammation.[338,345]

NIH-PA Author Manuscript NIH-PA Author Manuscript NIH-PA Author Manuscript

fMRI studies in MS patients have shown significant alterations in brain activation during cognitive tasks, including during tests of working memory, episodic memory, processing speed, and attention. MS patients show increased activation in the right hemisphere, most especially in the prefrontal cortex, during working memory tasks, as well as widespread increased activation during episodic memory tasks, which was positively associated with increased lesion load.[346–350] A similar finding of increased activation in the right prefrontal cortex was seen during a test of processing speed.[351] These increases in activation may represent compensatory changes to maintain clinical performance, as patients with performance deficits actually show decreased brain activation during episodic memory.[348] In tests of attention, patients showed variable changes in brain activation dependent on clinical stage, with mildly impaired patients showing increased activation and decreased activation observed in those with more severe cognitive impairment.[352,353]

Resting-state studies have also shown alterations in connectivity in patients with MS, although the findings have been mixed. In patients with primary and secondary progressive MS, decreased connectivity of the DMN is observed, particularly in the anterior cingulate. This decreased connectivity in the anterior cingulate is associated with functional impairments and DTI abnormalities in the corpus callosum and cingulum on DTI measures.[354,355] A study in RRMS patients showed decreased connectivity of the salience, working memory, sensorimotor, and visual networks, as well as the DMN, increased connectivity of the auditory network, and both increases and decreases in the connectivity of the executive control network.[354,356] Decreased connectivity was correlated to the extent of disability and lesion load.[354,356] Altered connectivity between networks was also observed with decreased connectivity between the executive control network and the DMN, but increased connectivity between the executive control network and the salience network.[354,356] However, other studies have primarily shown increased connectivity in various brain networks. For example, increased connectivity in several motor, sensory, and cognitive networks was observed in RRMS patients, with increased connectivity in frontoparietal networks associated with poorer clinical status.[354,357] Increased connectivity in the DMN, an attention network, and cognitive control network have also been shown to be associated with impaired cognition.[354,358] However, a different study demonstrated that increased connectivity in the cerebellum, middle temporal gyrus, occipital pole, and angular gyrus were associated with better cognition.[354,359] Reorganization of the DMN has also been reported in RRMS patients, with decreased connectivity in midline regions (i.e., anterior and posterior cingulate) and increased connectivity in peripheral posterior brain regions.[354,360] The conflicting findings in these studies may be related to the type of MS and disease severity of the patient population studied, as well as the brain locations evaluated.

PET studies in patients with MS have shown changes in cerebral perfusion and metabolism, as well as increased microglial activation. Reduced cerebral blood flow in gray matter and white matter is observed in MS patients, which correlates with impaired cognition and a higher level of disability.[2,361] Hypometabolism is also observed in the thalamus, deep cortical gray matter structures, and the frontal lobe.[2,362] This hypometabolism is associated with impaired cognition, as well as corpus callosum atrophy (left hemisphere more associated than the right).[2,362,363] In longitudinal studies, decreasing cortical metabolism was observed in MS patients, which was associated with disease progression. [2,364] Studies with [11C]PK-11195 have shown a widespread pattern of activated microglia in both lesion tissue and normal appearing gray matter and white matter.[2,365–367] The increased microglial activation observed in cortical gray matter was associated with increased clinical disability[2,368]

Studies of neuroimaging biomarkers in MS patients have routinely shown the presence of cerebral lesions in gray matter and white matter regions, along with brain atrophy and

NIH-PA Author Manuscript NIH-PA Author Manuscript NIH-PA Author Manuscript

alterations in brain function and molecular systems. Future studies in the earliest phases of MS will allow further exploration of the development and progression of the disease, as well as the efficacy of targeted treatments.

HIV-Associated Neurocognitive Disorder

HIV-associated neurocognitive disorder (HAND) primarily involves impairments in attention, executive function, motor speed, and memory. Structural MRI studies of patients with HAND showed gray matter atrophy throughout the cerebral cortex, particularly in anterior cingulate, lateral temporal cortex, primary motor and sensory cortices, and frontal and parietal lobes.[29,30,369–372] White matter atrophy and abnormalities are also common.[30,369,370] Some patients present with progressive multifocal leukoencephalopathy characterized by focal white matter lesions typically in subcortical regions.[373,374] Motor and cognitive symptoms are also associated with decreased basal ganglia volume.[370,375] DTI studies have shown that reductions in white matter integrity in the cortical white matter, corpus callosum, and corona radiata are associated with cognitive impairment.[376–378] MRS studies of patients with HAND have shown alterations in brain metabolites, including decreased NAA/Creatinine (Cr) and increased choline, mIns, choline/Cr, and mIns/Cr in the frontal white matter and basal ganglia.[369,375] In addition, HAND patients with concurrent hepatitis C infection show greater increases in mIns/Cr in the basal ganglia than those with only HAND.[379] fMRI studies have shown both decreased and increased activation during various cognitive and motor tasks. Specifically, decreased activation during a motor task was observed in HAND patients, while increased activation was observed during attention and working memory tasks in the frontal and parietal lobes.[380–382] Increased activation was also observed in HAND patients during episodic memory recognition, while decreased activation was observed during episodic encoding in the MTL.[383] Finally, decreased activation was observed in the left caudate, left dorsolateral prefrontal cortex, and bilateral ventral prefrontal cortex, while increased activation was observed in the right postcentral/supramarginal gyri during an executive function task.[384] Functional connectivity has also been evaluated in patients with HAND both during a cognitive task and at rest. A study of task-related connectivity during an executive function task showed reduced connectivity in the caudate, prefrontal cortex, and basal ganglia in HAND patients, while increased connectivity was observed in the caudate and anterior parietal lobe.[384] Resting-state connectivity in the DMN, salience network, and control network is also reduced in patientswith HAND.[385] Furthermore, decreased internet work connectivity has been observed, particularly between the DMN and a dorsal attention network.[385] Studies of perfusion with SPECT and MRI techniques have shown hypoperfusion in patients with HAND, particularly in the inferior lateral frontal lobe, inferior medial parietal lobe, and in other frontoparietal regions.[386,387] This hypoperfusion was associated with dementia severity.[387] Alternatively, hyperperfusion was observed in the posterior inferior parietal white matter and in deep gray matter structures.[386,388] Hypometabolism in the cerebral cortex and hypermetabolismin the basal ganglia was also observed in HAND patients using [18F]FDG PET.[389–391] Another study observed asymmetrical glucose metabolism in the prefrontal cortex and premotor regions in HAND patients. [392] Finally, a PET study utilizing tracers that bind to dopamine transporters (DAT) or D2 receptors observed decreased DAT binding in the putamen and ventral striatum but no difference in D2 receptor binding in HAND patients relative to HC.[393] The observed reductions in DAT binding were associated with disease severity.[393]

HIV-associated neurocognitive disorder is relatively common in HIV-positive individuals. Imaging studies of this disorder have found significant abnormalities in brain structure, function, perfusion, glucose metabolism, and neurotransmission which likely underlie the observed clinical dysfunction. The advent of retroviral therapies has been shown to alter the

NIH-PA Author Manuscript NIH-PA Author Manuscript NIH-PA Author Manuscript

observed brain changes associated with HAND; however, additional studies are needed. Additional studies designed to evaluate the clinical significance of imaging techniques in various HIV-positive populations, particularly in those who are treated with retroviral therapies, are ongoing and will provide important information about the nature of cognitive dysfunction in these individuals.

Prion Protein Diseases

Degenerative disorders and dementias caused by the accumulation of abnormal prion proteins can occur sporadically (sporadic CJD), due to exposure to food (variant CJD) or tissues (iatrogenic CJD) containing the abnormal prion protein, or due to a genetic variation in the prion protein gene (*PRNP*) (genetic CJD, Gerstmann-Straussler-Scheinker disease [GSS], fatal familial insomnia [FFI]). These diseases feature cognitive and motor dysfunction, although other presentations with various symptoms are possible. Imaging studies in sporadic CJD have primarily utilized DWI techniques to evaluate diffusion in gray matter and white matter structures. Sporadic CJD patients show increased diffusivity in the caudate, putamen, cerebellum, globus pallidus, and regions of the cerebral gray matter and white matter (Figs. 7A, –7B).[1,394–400] The thalamus may also show abnormalities in some forms of sporadic CJD. Changes in the basal ganglia are associated with a faster disease progression.[1,401] However, these alterations may disappear as the disease progresses in the presence of more severe atrophy.[396,399,402] DWI and T2-weighted imaging studies in variant CJD show abnormalities in the pulvinar thalamus and sometimes in the dorsomedial thalamic nuclei (Figs. 7E-H).[1,394,395,403–405] Other alterations are seen in the periaqueductal gray, caudate, and parieto-occipital white matter.[1,395,403] Similar to sporadic CJD, these alterations may disappear as the disease progresses and atrophic changes expand.[403] Studies utilizing MRS techniques have shown reduced NAA and increased mIns in patients with variant CJD, likely reflecting ongoing neurodegeneration. [1,394,395,406,407] SPECT studies have also shown cortical hypoperfusion in patients with variant CJD.[395,408] Iatrogenic CJD patients show increased diffusion on DWI and hyperintensities on T2-weighted scans in regions of the caudate head, putamen, cortical gray matter, and sometimes in the cerebellum and thalamus.[1,394,395,409] Longitudinally, iatrogenic CJD patients show progressive atrophy associated with disease progression.[395,410] MRS studies in iatrogenic CJD also demonstrated reduced NAA in the cerebellum.[1,407,411] Genetic prion diseases also show changes in MRI and PET studies. Altered diffusion in the striatum, thalamus, and frontal and occipital cortices was observed in most genetic CJD patients.[1,395] MRS studies have also shown increased levels of mIns, but no change in NAA level in the cerebral cortex and basal ganglia in genetic CJD.[1] Patients with GSS and some genetic CJD showed mixed results using structural MRI measures, with either no atrophy or generalized cerebral and cerebellar atrophy observed (Figs. 7C, –7D).[1,395,412–414] However, hyperintensities on T2-weighted scans were commonly observed in the basal ganglia and posterior limb of the internal capsule in GSS.[1,395,415] MRS studies also showed increased mIns in the cortex and basal ganglia of GSS patients.[416] SPECT and FDG PET studies in GSS patients demonstrate hypoperfusion in the cerebral cortex, most especially in the occipital lobe, and hypometabolism in frontal, temporal, and parietal lobes, respectively.[1,414,417,418] Patients with FFI may or may not show mild cerebral atrophy, but often increased diffusion in the thalamus is observed on DWI scans.[1,395,407,419–421] MRS studies in FFI patients have also shown decreased NAA and increased mIns in the thalamus. [1,421,422] Hypometabolism has also been observed using FDG PET in the thalamus and cingulate of patients with FFI with relative sparing of the occipital lobes.[1,423]

Despite being quite rare, prion diseases can result in pronounced and sometimes rapid cognitive, motor, and clinical decline. Imaging studies in prion diseases have shown atrophy and changes in gray matter and white matter diffusion, as well as altered metabolite levels

NIH-PA Author Manuscript NIH-PA Author Manuscript NIH-PA Author Manuscript

and reduced cerebral perfusion and metabolism. Future studies to further explore these rare diseases may provide additional insight into the pathology underlying prion diseases, as well as monitoring of potential treatments.

Differential Diagnosis of Dementias

Differential diagnosis of degenerative conditions not associated with a known genetic variant or other disease state (i.e., HIV) can sometimes be difficult due to overlapping clinical symptoms. In diseases presenting without motor symptoms, such as late-onset AD, atypical AD, and some forms of FTD, structural MRI and PET studies can often be helpful in differentiating between diseases (Table 1). Specifically, patients with AD show significant degeneration in the MTL, as well as in posterior brain regions (i.e., parietal lobe), while patients with FTD show primarily frontal lobe and lateral temporal lobe degeneration, with relative sparing of most parietal lobe regions. Furthermore, PET studies with amyloid tracers will provide good delineation of AD/atypical AD and FTD syndromes, as AD patients will typically show significant amyloid deposition and FTD patients usually will not. Distinguishing between traditional late-onset AD and atypical forms of AD is most commonly based on clinical symptoms, as domains other than memory tend to be more affected in the atypical forms. However, structural MRI may also provide additional support for specific diagnoses, with PCA patients often showing greater parietal and occipital atrophy and logopenic aphasia showing more asymmetrical left posterior temporal and temporoparietal atrophy than seen in traditional AD. Patients with CAA and VaD will also present with more vascular abnormalities, including microbleeds/microhemorrhages, and white matter lesions than seen in more typical late-onset AD patients. Furthermore, the pattern of FDG PET hypometabolism in vascular dementia is less diffuse with patchy areas corresponding to hypoperfusion compared with the pattern observed in AD patients, and widespread amyloid deposition in pure vascular dementia without CAA is not commonly observed.

Distinguishing between diseases associated with motor symptoms in the absence or presence of cognitive symptoms can also be quite difficult. Frontotemporal dementia with motor neuron disease and FTD-ALS both show cognitive and motor symptoms and are associated atrophy and reduced perfusion/metabolism in frontal and temporal lobes. Differentiation of these two diseases is probably not well assisted by neuroimaging techniques currently. Parkinson’s disease dementia and DLB show greater basal ganglia and less MTL atrophy than seen in typical AD patients, as well as decreased dopaminergic neurotransmission in the striatum on PET or SPECT. Distinguishing PDD/DLB from FTD-MND/ALS using neuroimaging can potentially be difficult, although PDD/ DLB patients tend to show more posterior cortical atrophy and hypoperfusion/metabolism, particularly in the parietal and occipital lobes, than seen in FTD-MND/ALS. Multiple sclerosis is characterized by the notable white matter lesions on T2-weighted and enhanced MRI scans, which are not as commonly seen in other degenerative disorders. Although MS is typically diagnosed at an earlier age, differentiation of MS and other demyelinating disorders and microvascular changes associated with aging or early VCI can be challenging in some cases. Finally, sporadic and variant prion diseases can be distinguished from most other dementias by the significant abnormalities seen in the thalamus relative to other areas of the brain, as well as history and other clinical features.

Conclusion

Imaging studies of neurodegenerative diseases and dementias are highly informative regarding structural, functional, and molecular brain changes underlying the observed clinical symptoms. Often neuroimaging techniques can be helpful if not essential for

NIH-PA Author Manuscript
NIH-PA Author Manuscript
NIH-PA Author Manuscript

differential diagnosis of various syndromes. Further studies with advanced MRI techniques and future PET tracers for proteinopathies beyond amyloid (i.e., tau, α-synuclein, and TDP-43)will likely provide even more information about pathology associated with the various degenerative and dementing syndromes. In addition, neuroimaging techniques may be useful in clinical trials of new therapeutics designed to treat these disabling and often refractory disorders for both monitoring disease-related changes or as end-points to complement current clinical outcome measures.

Acknowledgments

This manuscript was supported by grants from the National Institute on Aging (NIA R01 AG19771, P30 AG10133).

References

1. Letourneau-Guillon L, Wada R, Kucharczyk W. Imaging of prion diseases. J Magn Reson Imaging. 2012; 35(5):998–1012. [PubMed: 22499277]
2. Politis M, Piccini P. Positron emission tomography imaging in neurological disorders. J Neurol. 2012; 259(9):1769–1780. [PubMed: 22297461]
3. Alzheimer's Association. Facts and Figures. Chicago, IL: Alzheimer's Association; 2012.
4. McKhann, gmatter; Knopman, DS.; Chertkow, H., et al. The diagnosis of dementia due to Alzheimer's disease: recommendations from the National Institute on Aging-Alzheimer's Association workgroups on diagnostic guidelines for Alzheimer's disease. Alzheimers Dement. 2011; 7(3):263–269. [PubMed: 21514250]
5. Petersen RC, Smith GE, Waring SC, Ivnik RJ, Tangalos EG, Kokmen E. Mild cognitive impairment: clinical characterization and outcome. Arch Neurol. 1999; 56(3):303–308. [PubMed: 10190820]
6. Albert MS, DeKosky ST, Dickson D, et al. The diagnosis of mild cognitive impairment due to Alzheimer's disease: recommendations from the National Institute on Aging-Alzheimer's Association workgroups on diagnostic guidelines for Alzheimer's disease. Alzheimers Dement. 2011; 7(3):270–279. [PubMed: 21514249]
7. Sperling RA, Aisen PS, Beckett LA, et al. Toward defining the preclinical stages of Alzheimer's disease: recommendations from the National Institute on Aging-Alzheimer's Association workgroups on diagnostic guidelines for Alzheimer's disease. Alzheimers Dement. 2011; 7(3):280–292. [PubMed: 21514248]
8. Jack CR Jr, Knopman DS, Jagust WJ, et al. Hypothetical model of dynamic biomarkers of the Alzheimer's pathological cascade. Lancet Neurol. 2010; 9(1):119–128. [PubMed: 20083042]
9. Braak H, Braak E, Bohl J. Staging of Alzheimer-related cortical destruction. Eur Neurol. 1993; 33(6):403–408. [PubMed: 8307060]
10. Bateman RJ, Xiong C, Benzinger TL, et al. Dominantly Inherited Alzheimer Network. Clinical and biomarker changes in dominantly inherited Alzheimer's disease. N Engl J Med. 2012; 367(9):795–804. [PubMed: 22784036]
11. McGinnis SM. Neuroimaging in neurodegenerative dementias. Semin Neurol. 2012; 32(4):347–360. [PubMed: 23361481]
12. Andrade K, Kas A, Valabrègue R, et al. Visuospatial deficits in posterior cortical atrophy: structural and functional correlates. J Neurol Neurosurg Psychiatry. 2012; 83(9):860–863. [PubMed: 22645257]
13. Rohrer JD. Structural brain imaging in frontotemporal dementia. Biochim Biophys Acta. 2012; 1822(3):325–332. [PubMed: 21839829]
14. Viswanathan A, Greenberg SM. Cerebral amyloid angiopathy in the elderly. Ann Neurol. 2011; 70(6):871–880. [PubMed: 22190361]
15. Kinnecom C, Lev MH, Wendell L, et al. Course of cerebral amyloid angiopathy-related inflammation. Neurology. 2007; 68(17):1411–1416. [PubMed: 17452586]

NIH-PA Author Manuscript NIH-PA Author Manuscript NIH-PA Author Manuscript

16. Grossman M. Biomarkers to identify the pathological basis for frontotemporal lobar degeneration. J Mol Neurosci. 2011; 45(3):366–371. [PubMed: 21833654]
17. Whitwell JL, Josephs KA. Neuroimaging in frontotemporal lobar degeneration–predicting molecular pathology. Nat Rev Neurology. 2011; 8(3):131–142.
18. Lomen-Hoerth C. Clinical phenomenology and neuroimaging correlates in ALS-FTD. J Mol Neurosci. 2011; 45(3):656–662. [PubMed: 21971978]
19. Trojsi F, Monsurrò MR, Esposito F, Tedeschi G. Widespread structural and functional connectivity changes in amyotrophic lateral sclerosis: insights from advanced neuroimaging research. Neural Plast. 2012; 2012:473538. [PubMed: 22720174]
20. Watson R, Blamire AM, O'Brien JT. Magnetic resonance imaging in Lewy body dementias. Dement Geriatr Cogn Disord. 2009; 28(6):493–506. [PubMed: 19996594]
21. Silbert LC, Kaye J. Neuroimaging and cognition in Parkinson's disease dementia. Brain Pathol. 2010; 20(3):646–653. [PubMed: 20522090]
22. Papp KV, Kaplan RF, Snyder PJ. Biological markers of cognition in prodromal Huntington's disease: a review. Brain Cogn. 2011; 77(2):280–291. [PubMed: 21889251]
23. Sá MJ. Physiopathology of symptoms and signs in multiple sclerosis. Arq Neuropsiquiatr. 2012; 70(9):733–740. [PubMed: 22990733]
24. Compston A, Coles A. Multiple sclerosis. Lancet. 2008; 372(9648):1502–1517. [PubMed: 18970977]
25. Nakahara J, Maeda M, Aiso S, Suzuki N. Current concepts in multiple sclerosis: autoimmunity versus oligodendrogliopathy. Clin Rev Allergy Immunol. 2012; 42(1):26–34. [PubMed: 22189514]
26. Lublin FD, Reingold SC. National Multiple Sclerosis Society (USA) Advisory Committee on Clinical Trials of New Agents in Multiple Sclerosis. Defining the clinical course of multiple sclerosis: results of an international survey. Neurology. 1996; 46(4):907–911. [PubMed: 8780061]
27. Antinori A, Arendt G, Becker JT, et al. Updated research nosology for HIV-associated neurocognitive disorders. Neurology. 2007; 69(18):1789–1799. [PubMed: 17914061]
28. Tozzi V, Balestra P, Lorenzini P, et al. Prevalence and risk factors for human immunodeficiency virus-associated neurocognitive impairment, 1996 to 2002: results from an urban observational cohort. J Neurovirol. 2005; 11(3):265–273. [PubMed: 16036806]
29. Bonnet F, Amieva H, Marquant F, et al. S CO3 Aquitaine Cohort. Cognitive disorders in HIV-infected patients: are they HIV-related? AIDS. 2013; 27(3):391–400. [PubMed: 23079813]
30. Steinbrink F, Evers S, Buerke B, et al. German Competence Network HIV/AIDS. Cognitive impairment in HIV infection is associated with MRI and CSF pattern of neurodegeneration. Eur J Neurol. 2013; 20(3):420–428. [PubMed: 23095123]
31. Robertson K, Liner J, Heaton R. Neuropsychological assessment of HIV-infected populations in international settings. Neuropsychol Rev. 2009; 19(2):232–249. [PubMed: 19455425]
32. Alsop DC, Dai W, Grossman M, Detre JA. Arterial spin labeling blood flow MRI: its role in the early characterization of Alzheimer's disease. J Alzheimers Dis. 2010; 20(3):871–880. [PubMed: 20413865]
33. Wang Y, Saykin AJ, Pfeuffer J, et al. Regional reproducibility of pulsed arterial spin labeling perfusion imaging at 3T. Neuroimage. 2011; 54(2):1188–1195. [PubMed: 20800097]
34. Ogawa S, Tank DW, Menon R, et al. Intrinsic signal changes accompanying sensory stimulation: functional brain mapping with magnetic resonance imaging. Proc Natl Acad Sci U S A. 1992; 89(13):5951–5955. [PubMed: 1631079]
35. Logothetis NK, Pauls J, Augath M, Trinath T, Oeltermann A. Neurophysiological investigation of the basis of the fMRI signal. Nature. 2001; 412(6843):150–157. [PubMed: 11449264]
36. Greicius MD, Krasnow B, Reiss AL, Menon V. Functional connectivity in the resting brain: a network analysis of the default mode hypothesis. Proc Natl Acad Sci U S A. 2003; 100(1):253–258. [PubMed: 12506194]
37. Jack CR Jr, Bernstein MA, Borowski BJ, et al. Alzheimer's Disease Neuroimaging Initiative. Update on the magnetic resonance imaging core of the Alzheimer's disease neuroimaging initiative. Alzheimers Dement. 2010; 6(3):212–220. [PubMed: 20451869]

NIH-PA Author Manuscript
NIH-PA Author Manuscript
NIH-PA Author Manuscript

38. Weiner MW, Veitch DP, Aisen PS, et al. Alzheimer's Disease Neuroimaging Initiative. The Alzheimer's Disease Neuroimaging Initiative: a review of papers published since its inception. Alzheimers Dement. 2013 Sep; 9(5):e111–e194. [PubMed: 23932184]
39. Risacher SL, Saykin AJ, West JD, Shen L, Firpi HA, McDonald BC. Alzheimer's Disease Neuroimaging Initiative (ADNI). Baseline MRI predictors of conversion from MCI to probable AD in the ADNI cohort. Curr Alzheimer Res. 2009; 6(4):347–361. [PubMed: 19689234]
40. Du AT, Schuff N, Amend D, et al. Magnetic resonance imaging of the entorhinal cortex and hippocampus in mild cognitive impairment and Alzheimer's disease. J Neurol Neurosurg Psychiatry. 2001; 71(4):441–447. [PubMed: 11561025]
41. Chételat G, Desgranges B, De La Sayette V, Viader F, Eustache F, Baron JC. Mapping gray matter loss with voxel-based morphometry in mild cognitive impairment. Neuroreport. 2002; 13(15): 1939–1943. [PubMed: 12395096]
42. Hämäläinen A, Tervo S, Grau-Olivares M, et al. Voxel-based morphometry to detect brain atrophy in progressive mild cognitive impairment. Neuroimage. 2007; 37(4):1122–1131. [PubMed: 17683950]
43. Whitwell JL, Shiung MM, Przybelski SA, et al. MRI patterns of atrophy associated with progression to AD in amnestic mild cognitive impairment. Neurology. 2008; 70(7):512–520. [PubMed: 17898323]
44. deToledo-Morrell L, Stoub TR, Bulgakova M, et al. MRI-derived entorhinal volume is a good predictor of conversion from MCI to AD. Neurobiol Aging. 2004; 25(9):1197–1203. [PubMed: 15312965]
45. Devanand DP, Liu X, Tabert MH, et al. Combining early markers strongly predicts conversion from mild cognitive impairment to Alzheimer's disease. Biol Psychiatry. 2008; 64(10):871–879. [PubMed: 18723162]
46. Jack CR Jr, Petersen RC, Xu YC, et al. Prediction of AD with MRI-based hippocampal volume in mild cognitive impairment. Neurology. 1999; 52(7):1397–1403. [PubMed: 10227624]
47. Barnes J, Bartlett JW, van de Pol LA, et al. A meta-analysis of hippocampal atrophy rates in Alzheimer's disease. Neurobiol Aging. 2009; 30(11):1711–1723. [PubMed: 18346820]
48. Honea RA, Vidoni E, Harsha A, Burns JM. Impact of APOE on the healthy aging brain: a voxel-based MRI and DTI study. J Alzheimers Dis. 2009; 18(3):553–564. [PubMed: 19584447]
49. Risacher SL, Shen L, West JD, et al. Alzheimer's Disease Neuroimaging Initiative (ADNI). Longitudinal MRI atrophy biomarkers: relationship to conversion in the ADNI cohort. Neurobiol Aging. 2010; 31(8):1401–1418. [PubMed: 20620664]
50. Donix M, Burggren AC, Suthana NA, et al. Longitudinal changes in medial temporal cortical thickness in normal subjects with the APOE-4 polymorphism. Neuroimage. 2010; 53(1):37–43. [PubMed: 20541611]
51. Wishart HA, Saykin AJ, McAllister TW, et al. Regional brain atrophy in cognitively intact adults with a single APOE epsilon4 allele. Neurology. 2006; 67(7):1221–1224. [PubMed: 17030756]
52. Chételat G, Villemagne VL, Bourgeat P, et al. Australian Imaging Biomarkers and Lifestyle Research Group. Relationship between atrophy and beta-amyloid deposition in Alzheimer disease. Ann Neurol. 2010; 67(3):317–324. [PubMed: 20373343]
53. Bourgeat P, Chételat G, Villemagne VL, et al. AIBL Research Group. Beta-amyloid burden in the temporal neocortex is related to hippocampal atrophy in elderly subjects without dementia. Neurology. 2010; 74(2):121–127. [PubMed: 20065247]
54. Jack CR Jr, Lowe VJ, Senjem ML, et al. 11C PiB and structural MRI provide complementary information in imaging of Alzheimer's disease and amnestic mild cognitive impairment. Brain. 2008; 131(Pt 3):665–680. [PubMed: 18263627]
55. Saykin AJ, Wishart HA, Rabin LA, et al. Older adults with cognitive complaints show brain atrophy similar to that of amnestic MCI. Neurology. 2006; 67(5):834–842. [PubMed: 16966547]
56. Scheef L, Spottke A, Daerr M, et al. Glucose metabolism, gray matter structure, and memory decline in subjective memory impairment. Neurology. 2012; 79(13):1332–1339. [PubMed: 22914828]

NIH-PA Author Manuscript NIH-PA Author Manuscript NIH-PA Author Manuscript

57. Striepens N, Scheef L, Wind A, et al. Volume loss of the medial temporal lobe structures in subjective memory impairment. Dement Geriatr Cogn Disord. 2010; 29(1):75–81. [PubMed: 20110703]
58. Ibrahim I, Horacek J, Bartos A, et al. Combination of voxel based morphometry and diffusion tensor imaging in patients with Alzheimer's disease. Neuroendocrinol Lett. 2009; 30(1):39–45. [PubMed: 19300399]
59. Rose SE, McMahon KL, Janke AL, et al. Diffusion indices on magnetic resonance imaging and neuropsychological performance in amnestic mild cognitive impairment. J Neurol Neurosurg Psychiatry. 2006; 77(10):1122–1128. [PubMed: 16754694]
60. Zhang Y, Schuff N, Jahng GH, et al. Diffusion tensor imaging of cingulum fibers in mild cognitive impairment and Alzheimer disease. Neurology. 2007; 68(1):13–19. [PubMed: 17200485]
61. Fellgiebel A, Wille P, Müller MJ, et al. Ultrastructural hippocampal and white matter alterations in mild cognitive impairment: a diffusion tensor imaging study. Dement Geriatr Cogn Disord. 2004; 18(1):101–108. [PubMed: 15087585]
62. Wang Y, West JD, Flashman LA, et al. Selective changes in white matter integrity in MCI and older adults with cognitive complaints. Biochim Biophys Acta. 2012; 1822(3):423–430. [PubMed: 21867750]
63. Chantal S, Braun CM, Bouchard RW, Labelle M, Boulanger Y. Similar 1H magnetic resonance spectroscopic metabolic pattern in the medial temporal lobes of patients with mild cognitive impairment and Alzheimer disease. Brain Res. 2004; 1003(1–2):26–35. [PubMed: 15019560]
64. Schuff N, Capizzano AA, Du AT, et al. Selective reduction of N-acetylaspartate in medial temporal and parietal lobes in AD. Neurology. 2002; 58(6):928–935. [PubMed: 11914410]
65. Kantarci K, Jack CR Jr, Xu YC, et al. Regional metabolic patterns in mild cognitive impairment and Alzheimer's disease: A 1H MRS study. Neurology. 2000; 55(2):210–217. [PubMed: 10908893]
66. Harris GJ, Lewis RF, Satlin A, et al. Dynamic susceptibility contrast MR imaging of regional cerebral blood volume in Alzheimer disease: a promising alternative to nuclear medicine. AJNR Am J Neuroradiol. 1998; 19(9):1727–1732. [PubMed: 9802497]
67. Celone KA, Calhoun VD, Dickerson BC, et al. Alterations in memory networks in mild cognitive impairment and Alzheimer's disease: an independent component analysis. J Neurosci. 2006; 26(40):10222–10231. [PubMed: 17021177]
68. Dickerson BC, Salat DH, Greve DN, et al. Increased hippocampal activation in mild cognitive impairment compared to normal aging and AD. Neurology. 2005; 65(3):404–411. [PubMed: 16087905]
69. Johnson SC, Schmitz TW, Moritz CH, et al. Activation of brain regions vulnerable to Alzheimer's disease: the effect of mild cognitive impairment. Neurobiol Aging. 2006; 27(11):1604–1612. [PubMed: 16226349]
70. Trivedi MA, Murphy CM, Goetz C, et al. fMRI activation changes during successful episodic memory encoding and recognition in amnestic mild cognitive impairment relative to cognitively healthy older adults. Dement Geriatr Cogn Disord. 2008; 26(2):123–137. [PubMed: 18663302]
71. Saykin AJ, Flashman LA, Frutiger SA, et al. Neuroanatomic substrates of semantic memory impairment in Alzheimer's disease: patterns of functional MRI activation. J Int Neuropsychol Soc. 1999; 5(5):377–392. [PubMed: 10439584]
72. Pariente J, Cole S, Henson R, et al. Alzheimer's patients engage an alternative network during a memory task. Ann Neurol. 2005; 58(6):870–879. [PubMed: 16315273]
73. O'Brien JL, O'Keefe KM, LaViolette PS, et al. Longitudinal fMRI in elderly reveals loss of hippocampal activation with clinical decline. Neurology. 2010; 74(24):1969–1976. [PubMed: 20463288]
74. Bookheimer SY, Strojwas MH, Cohen MS, et al. Patterns of brain activation in people at risk for Alzheimer's disease. N Engl J Med. 2000; 343(7):450–456. [PubMed: 10944562]
75. Johnson SC, Schmitz TW, Trivedi MA, et al. The influence of Alzheimer disease family history and apolipoprotein E epsilon4 on mesial temporal lobe activation. J Neurosci. 2006; 26(22):6069–6076. [PubMed: 16738250]

NIH-PA Author Manuscript NIH-PA Author Manuscript NIH-PA Author Manuscript

76. Wishart HA, Saykin AJ, Rabin LA, et al. Increased brain activation during working memory in cognitively intact adults with the APOE epsilon4 allele. Am J Psychiatry. 2006; 163(9):1603–1610. [PubMed: 16946187]
77. Johnson SC, Saykin AJ, Baxter LC, et al. The relationship between fMRI activation and cerebral atrophy: comparison of normal aging and Alzheimer disease. Neuroimage. 2000; 11(3):179–187. [PubMed: 10694460]
78. Grady CL, McIntosh AR, Beig S, Keightley ML, Burian H, Black SE. Evidence from functional neuroimaging of a compensatory prefrontal network in Alzheimer's disease. J Neurosci. 2003; 23(3):986–993. [PubMed: 12574428]
79. Zhou Y, Dougherty JH Jr, Hubner KF, Bai B, Cannon RL, Hutson RK. Abnormal connectivity in the posterior cingulate and hippocampus in early Alzheimer's disease and mild cognitive impairment. Alzheimers Dement. 2008; 4(4):265–270. [PubMed: 18631977]
80. Buckner RL, Snyder AZ, Shannon BJ, et al. Molecular, structural, and functional characterization of Alzheimer's disease: evidence for a relationship between default activity, amyloid, and memory. J Neurosci. 2005; 25(34):7709–7717. [PubMed: 16120771]
81. Greicius MD, Srivastava G, Reiss AL, Menon V. Default-mode network activity distinguishes Alzheimer's disease from healthy aging: evidence from functional MRI. Proc Natl Acad Sci U S A. 2004; 101(13):4637–4642. [PubMed: 15070770]
82. Bai F, Zhang Z, Watson DR, et al. Abnormal functional connectivity of hippocampus during episodic memory retrieval processing network in amnestic mild cognitive impairment. Biol Psychiatry. 2009; 65(11):951–958. [PubMed: 19028382]
83. Sperling RA, Laviolette PS, O'Keefe K, et al. Amyloid deposition is associated with impaired default network function in older persons without dementia. Neuron. 2009; 63(2):178–188. [PubMed: 19640477]
84. Rami L, Sala-Llonch R, Solé-Padullés C, et al. Distinct functional activity of the precuneus and posterior cingulate cortex during encoding in the preclinical stage of Alzheimer's disease. J Alzheimers Dis. 2012; 31(3):517–526. [PubMed: 22596271]
85. Trachtenberg AJ, Filippini N, Ebmeier KP, Smith SM, Karpe F, Mackay CE. The effects of APOE on the functional architecture of the resting brain. Neuroimage. 2012; 59(1):565–572. [PubMed: 21851856]
86. Wang Y, Risacher SL, West JD, et al. Altered default mode network connectivity in older adults with cognitive complaints and amnestic mild cognitive impairment. J Alzheimers Dis. 2013; 35(4): 751–760. [PubMed: 23481685]
87. Langbaum JB, Chen K, Lee W, et al. Alzheimer's Disease Neuroimaging Initiative. Categorical and correlational analyses of baseline fluorodeoxyglucose positron emission tomography images from the Alzheimer's Disease Neuroimaging Initiative (ADNI). Neuroimage. 2009; 45(4):1107–1116. [PubMed: 19349228]
88. Mosconi L. Brain glucose metabolism in the early and specific diagnosis of Alzheimer's disease. FDG-PET studies in MCI and AD. Eur J Nucl Med Mol Imaging. 2005; 32(4):486–510. [PubMed: 15747152]
89. Herholz K, Nordberg A, Salmon E, et al. Impairment of neocortical metabolism predicts progression in Alzheimer's disease. Dement Geriatr Cogn Disord. 1999; 10(6):494–504. [PubMed: 10559566]
90. Alexander GE, Chen K, Pietrini P, Rapoport SI, Reiman EM. Longitudinal PET evaluation of cerebral metabolic decline in dementia: a potential outcome measure in Alzheimer's disease treatment studies. Am J Psychiatry. 2002; 159(5):738–745. [PubMed: 11986126]
91. Small GW, Ercoli LM, Silverman DH, et al. Cerebral metabolic and cognitive decline in persons at genetic risk for Alzheimer's disease. Proc Natl Acad Sci U S A. 2000; 97(11):6037–6042. [PubMed: 10811879]
92. de Leon MJ, Convit A, Wolf OT, et al. Prediction of cognitive decline in normal elderly subjects with 2-[(18)F]fluoro-2-deoxy-D-glucose/positron-emission tomography (FDG/PET). Proc Natl Acad Sci U S A. 2001; 98(19):10966–10971. [PubMed: 11526211]

NIH-PA Author Manuscript NIH-PA Author Manuscript NIH-PA Author Manuscript

93. Mosconi L, De Santi S, Brys M, et al. Hypometabolism and altered cerebrospinal fluid markers in normal apolipoprotein E E4 carriers with subjective memory complaints. Biol Psychiatry. 2008; 63(6):609–618. [PubMed: 17720148]

94. Klunk WE, Engler H, Nordberg A, et al. Imaging brain amyloid in Alzheimer's disease with Pittsburgh compound-B. Ann Neurol. 2004; 55(3):306–319. [PubMed: 14991808]

95. Jack CR Jr, Lowe VJ, Weigand SD, et al. Alzheimer's Disease Neuroimaging Initiative. Serial PIB and MRI in normal, mild cognitive impairment and Alzheimer's disease: implications for sequence of pathological events in Alzheimer's disease. Brain. 2009; 132(Pt 5):1355–1365. [PubMed: 19339253]

96. Johnson KA, Fox NC, Sperling RA, Klunk WE. Brain imaging in Alzheimer disease. Cold Spring Harb Perspect Med. 2012; 2(4):a006213. [PubMed: 22474610]

97. Forsberg A, Engler H, Almkvist O, et al. PET imaging of amyloid deposition in patientswith mild cognitive impairment. Neurobiol Aging. 2008; 29(10):1456–1465. [PubMed: 17499392]

98. Klunk WE, Mathis CA, Price JC, Lopresti BJ, DeKosky ST. Two-year follow-up of amyloid deposition in patients with Alzheimer's disease. Brain. 2006; 129(Pt 11):2805–2807. [PubMed: 17071918]

99. Reiman EM, Chen K, Liu X, et al. Fibrillar amyloid-beta burden in cognitively normal people at 3 levels of genetic risk for Alzheimer's disease. Proc Natl Acad Sci U S A. 2009; 106(16):6820–6825. [PubMed: 19346482]

100. Honea RA, Vidoni ED, Swerdlow RH, Burns JM. Alzheimer's Disease Neuroimaging Initiative. Maternal family history is associated with Alzheimer's disease biomarkers. J Alzheimers Dis. 2012; 31(3):659–668. [PubMed: 22669011]

101. Mosconi L, Rinne JO, Tsui WH, et al. Increased fibrillar amyloid-beta burden in normal individuals with a family history of late-onset Alzheimer's. Proc Natl Acad Sci U S A. 2010; 107(13):5949–5954. [PubMed: 20231448]

102. Rowe CC, Ellis KA, Rimajova M, et al. Amyloid imaging results from the Australian Imaging, Biomarkers and Lifestyle (AIBL) study of aging. Neurobiol Aging. 2010; 31(8):1275–1283. [PubMed: 20472326]

103. Clark CM, Schneider JA, Bedell BJ, et al. AV45-A07 Study Group. Use of florbetapir-PET for imaging beta-amyloid pathology. JAMA. 2011; 305(3):275–283. [PubMed: 21245183]

104. Rinne JO, Kaasinen V, Järvenpää T, et al. Brain acetylcholinesterase activity in mild cognitive impairment and early Alzheimer's disease. J Neurol Neurosurg Psychiatry. 2003; 74(1):113–115. [PubMed: 12486280]

105. Herholz K, Weisenbach S, Kalbe E, Diederich NJ, Heiss WD. Cerebral acetylcholine esterase activity in mild cognitive impairment. Neuroreport. 2005; 16(13):1431–1434. [PubMed: 16110265]

106. Sabri O, Kendziorra K, Wolf H, Gertz HJ, Brust P. Acetylcholine receptors in dementia and mild cognitive impairment. Eur J Nucl Med Mol Imaging. 2008; 35(Suppl 1):S30–S45. [PubMed: 18228017]

107. Pappatà S, Salvatore E, Postiglione A. In vivo imaging of neurotransmission and brain receptors in dementia. J Neuroimaging. 2008; 18(2):111–124. [PubMed: 18380693]

108. Hasselbalch SG, Madsen K, Svarer C, et al. Reduced 5-HT2A receptor binding in patients with mild cognitive impairment. Neurobiol Aging. 2008; 29(12):1830–1838. [PubMed: 17544547]

109. Edison P, Archer HA, Gerhard A, et al. Microglia, amyloid, and cognition in Alzheimer's disease: An [11C](R)PK11195-PET and [11C]PIB-PET study. Neurobiol Dis. 2008; 32(3):412–419. [PubMed: 18786637]

110. Okello A, Edison P, Archer HA, et al. Microglial activation and amyloid deposition in mild cognitive impairment: a PET study. Neurology. 2009; 72(1):56–62. [PubMed: 19122031]

111. Wiley CA, Lopresti BJ, Venneti S, et al. Carbon 11-labeled Pittsburgh compound B and carbon 11-labeled (R)-PK11195 positron emission tomographic imaging in Alzheimer disease. Arch Neurol. 2009; 66(1):60–67. [PubMed: 19139300]

112. Ringman JM, O'Neill J, Geschwind D, et al. Diffusion tensor imaging in preclinical and presymptomatic carriers of familial Alzheimer's disease mutations. Brain. 2007; 130(Pt 7):1767–1776. [PubMed: 17522104]

NIH-PA Author Manuscript

NIH-PA Author Manuscript

NIH-PA Author Manuscript

113. Ringman JM, Younkin SG, Pratico D, et al. Biochemical markers in persons with preclinical familial Alzheimer disease. Neurology. 2008; 71(2):85–92. [PubMed: 18509095]

114. Fox NC, Warrington EK, Freeborough PA, et al. Presymptomatic hippocampal atrophy in Alzheimer's disease. A longitudinal MRI study. Brain. 1996; 119(Pt 6):2001–2007. [PubMed: 9010004]

115. Gregory GC, Macdonald V, Schofield PR, Kril JJ, Halliday gmatter. Differences in regional brain atrophy in genetic forms of Alzheimer's disease. Neurobiol Aging. 2006; 27(3):387–393. [PubMed: 15894410]

116. Bateman RJ, Aisen PS, De Strooper B, et al. Autosomal-dominant Alzheimer's disease: a review and proposal for the prevention of Alzheimer's disease. Alzheimers Res Ther. 2011; 3(1):1. [PubMed: 21211070]

117. Villemagne VL, Ataka S, Mizuno T, et al. High striatal amyloid beta-peptide deposition across different autosomal Alzheimer disease mutation types. Arch Neurol. 2009; 66(12):1537–1544. [PubMed: 20008660]

118. Migliaccio R, Agosta F, Scola E, et al. Ventral and dorsal visual streams in posterior cortical atrophy: a DT MRI study. Neurobiol Aging. 2012; 33(11):2572–2584. [PubMed: 22277261]

119. Lehmann M, Crutch SJ, Ridgway GR, et al. Cortical thickness and voxel-based morphometry in posterior cortical atrophy and typical Alzheimer's disease. Neurobiol Aging. 2011; 32(8):1466–1476. [PubMed: 19781814]

120. de Souza LC, Lehéricy S, Dubois B, Stella F, Sarazin M. Neuroimaging in dementias. Curr Opin Psychiatry. 2012; 25(6):473–479. [PubMed: 23037962]

121. Kas A, de Souza LC, Samri D, et al. Neural correlates of cognitive impairment in posterior cortical atrophy. Brain. 2011; 134(Pt 5):1464–1478. [PubMed: 21478188]

122. Ng SY, Villemagne VL, Masters CL, Rowe CC. Evaluating atypical dementia syndromes using positron emission tomography with carbon 11 labeled Pittsburgh compound B. Arch Neurol. 2007; 64(8):1140–1144. [PubMed: 17698704]

123. Madhavan A, Whitwell JL, Weigand SD, et al. FDG PET and MRI in logopenic primary progressive aphasia versus dementia of the Alzheimer's type. PLoS ONE. 2013; 8(4):e62471. [PubMed: 23626825]

124. Mahoney CJ, Malone IB, Ridgway GR, et al. White matter tract signatures of the progressive aphasias. Neurobiol Aging. 2013; 34(6):1687–1699. [PubMed: 23312804]

125. Magnin E, Cattin F, Vandel P, Galmiche J, Moulin T, Rumbach L. Fractional anisotropy in three variants of primary progressive aphasia. Eur Neurol. 2012; 68(4):229–233. [PubMed: 22964895]

126. Rabinovici GD, Jagust WJ, Furst AJ, et al. Abeta amyloid and glucose metabolism in three variants of primary progressive aphasia. Ann Neurol. 2008; 64(4):388–401. [PubMed: 18991338]

127. Wattjes MP. Structural MRI. Int Psychogeriatr. 2011 Sep; 23(Suppl 2):S13–S24. [PubMed: 21729419]

128. Tartaglia MC, Rosen HJ, Miller BL. Neuroimaging in dementia. Neurotherapeutics. 2011; 8(1): 82–92. [PubMed: 21274688]

129. Dumas A, Dierksen GA, Gurol ME, et al. Functional magnetic resonance imaging detection of vascular reactivity in cerebral amyloid angiopathy. Ann Neurol. 2012; 72(1):76–81. [PubMed: 22829269]

130. Chung YA, OJH, Kim JY, et al. Hypoperfusion and ischemia in cerebral amyloid angiopathy documented by 99mTc-ECD brain perfusion SPECT. J Nucl Med. 2009; 50(12):1969–1974. [PubMed: 19910418]

131. Villemagne VL, Rowe CC. Amyloid imaging. Int Psychogeriatr. 2011 Sep; 23(Suppl 2):S41–S49. [PubMed: 21729418]

132. Du AT, Schuff N, Laakso MP, et al. Effects of subcortical ischemic vascular dementia and AD on entorhinal cortex and hippocampus. Neurology. 2002; 58(11):1635–1641. [PubMed: 12058091]

133. Eckerström C, Olsson E, Klasson N, et al. High white matter lesion load is associated with hippocampal atrophy in mild cognitive impairment. Dement Geriatr Cogn Disord. 2011; 31(2): 132–138. [PubMed: 21293123]

NIH-PA Author Manuscript
NIH-PA Author Manuscript
NIH-PA Author Manuscript

134. Fein G, Di Sclafani V, Tanabe J, et al. Hippocampal and cortical atrophy predict dementia in subcortical ischemic vascular disease. Neurology. 2000; 55(11):1626–1635. [PubMed: 11113215]

135. Jagust WJ, Zheng L, Harvey DJ, et al. Neuropathological basis of magnetic resonance images in aging and dementia. Ann Neurol. 2008; 63(1):72–80. [PubMed: 18157909]

136. Mungas D, Jagust WJ, Reed BR, et al. MRI predictors of cognition in subcortical ischemic vascular disease and Alzheimer's disease. Neurology. 2001; 57(12):2229–2235. [PubMed: 11756602]

137. Scher AI, Xu Y, Korf ES, et al. Hippocampal morphometry in population-based incident Alzheimer's disease and vascular dementia: the HAAS. J Neurol Neurosurg Psychiatry. 2011; 82(4):373–376. [PubMed: 20826877]

138. Mori E. Impact of subcortical ischemic lesions on behavior and cognition. Ann N Y Acad Sci. 2002; 977:141–148. [PubMed: 12480744]

139. van de Pol LA, Korf ES, van der Flier wmatter, et al. Magnetic resonance imaging predictors of cognition in mild cognitive impairment. Arch Neurol. 2007; 64(7):1023–1028. [PubMed: 17620494]

140. Verdelho A, Madureira S, Moleiro C, et al. LADIS Study. White matter changes and diabetes predict cognitive decline in the elderly: the LADIS study. Neurology. 2010; 75(2):160–167. [PubMed: 20625169]

141. Cho H, Kwon JH, Seo HJ. Medial temporal lobe atrophy in vascular dementia: visual temporal lobe rating scale. Arch Gerontol Geriatr. 2009; 48(3):415–418. [PubMed: 18468705]

142. Li C, Du H, Zheng J, Wang J. A voxel-based morphometric analysis of cerebral gray matter in subcortical ischemic vascular dementia patients and normal aged controls. Int J Med Sci. 2011; 8(6):482–486. [PubMed: 21850200]

143. Seo SW, Ahn J, Yoon U, et al. Cortical thinning in vascular mild cognitive impairment and vascular dementia of subcortical type. J Neuroimaging. 2010; 20(1):37–45. [PubMed: 19220710]

144. van de Pol L, Gertz HJ, Scheltens P, Wolf H. Hippocampal atrophy in subcortical vascular dementia. Neurodegener Dis. 2011; 8(6):465–469. [PubMed: 21613775]

145. Chen Y, Chen X, Xiao W, Mok VC, Wong KS, Tang WK. Frontal lobe atrophy is associated with small vessel disease in ischemic stroke patients. Clin Neurol Neurosurg. 2009; 111(10):852–857. [PubMed: 19744770]

146. Looi JC, Tatham V, Kumar R, et al. Caudate nucleus volumes in stroke and vascular dementia. Psychiatry Res. 2009; 174(1):67–75. [PubMed: 19796921]

147. Chen TF, Lin CC, Chen YF, et al. Diffusion tensor changes in patients with amnesic mild cognitive impairment and various dementias. Psychiatry Res. 2009; 173(1):15–21. [PubMed: 19442496]

148. Della Nave R, Foresti S, Pratesi A, et al. Whole-brain histogram and voxel-based analyses of diffusion tensor imaging in patients with leukoaraiosis: correlation with motor and cognitive impairment. AJNR Am J Neuroradiol. 2007; 28(7):1313–1319. [PubMed: 17698534]

149. Fu JL, Zhang T, Chang C, Zhang YZ, Li WB. The value of diffusion tensor imaging in the differential diagnosis of subcortical ischemic vascular dementia and Alzheimer's disease in patients with only mild white matter alterations on T2-weighted images. Acta Radiol. 2012; 53(3):312–317. [PubMed: 22416261]

150. Kim SH, Park JS, Ahn HJ, et al. Voxel-based analysis of diffusion tensor imaging in patients with subcortical vascular cognitive impairment: correlates with cognitive and motor deficits. J Neuroimaging. 2011; 21(4):317–324. [PubMed: 20977535]

151. Nitkunan A, Barrick TR, Charlton RA, Clark CA, Markus HS. Multimodal MRI in cerebral small vessel disease: its relationship with cognition and sensitivity to change over time. Stroke. 2008; 39(7):1999–2005. [PubMed: 18436880]

152. O'Sullivan M, Morris RG, Huckstep B, Jones DK, Williams SC, Markus HS. Diffusion tensor MRI correlates with executive dysfunction in patients with ischaemic leukoaraiosis. J Neurol Neurosurg Psychiatry. 2004; 75(3):441–447. [PubMed: 14966162]

NIH-PA Author Manuscript NIH-PA Author Manuscript NIH-PA Author Manuscript

153. O'Sullivan M, Summers PE, Jones DK, Jarosz JM, Williams SC, Markus HS. Normal-appearing white matter in ischemic leukoaraiosis: a diffusion tensor MRI study. Neurology. 2001; 57(12): 2307–2310. [PubMed: 11756617]

154. Xu Q, Zhou Y, Li YS, et al. Diffusion tensor imaging changes correlate with cognition better than conventional MRI findings in patients with subcortical ischemic vascular disease. Dement Geriatr Cogn Disord. 2010; 30(4):317–326. [PubMed: 20881397]

155. Li C, Zheng J, Wang J, Gui L. Comparison between Alzheimer's disease and subcortical vascular dementia: attentional cortex study in functional magnetic resonance imaging. J Int Med Res. 2011; 39(4):1413–1419. [PubMed: 21986142]

156. Tak S, Yoon SJ, Jang J, Yoo K, Jeong Y, Ye JC. Quantitative analysis of hemodynamic and metabolic changes in subcortical vascular dementia using simultaneous near-infrared spectroscopy and fMRI measurements. Neuroimage. 2011; 55(1):176–184. [PubMed: 21094685]

157. Sun YW, Qin LD, Zhou Y, et al. Abnormal functional connectivity in patients with vascular cognitive impairment, no dementia: a resting-state functional magnetic resonance imaging study. Behav Brain Res. 2011; 223(2):388–394. [PubMed: 21605598]

158. Schuff N, Matsumoto S, Kmiecik J, et al. Cerebral blood flow in ischemic vascular dementia and Alzheimer's disease, measured by arterial spin-labeling magnetic resonance imaging. Alzheimers Dement. 2009; 5(6):454–462. [PubMed: 19896584]

159. Pakrasi S, O'Brien JT. Emission tomography in dementia. Nucl Med Commun. 2005; 26(3):189–196. [PubMed: 15722899]

160. Powers WJ, Zazulia AR. PET in Cerebrovascular Disease. PET Clin. 2010; 5(1):83106. [PubMed: 20543975]

161. Heiss WD, Zimmermann-Meinzingen S. PET imaging in the differential diagnosis of vascular dementia. J Neurol Sci. 2012; 322(1–2):268–273. [PubMed: 23043907]

162. Yoon CW, Shin JS, Kim HJ, et al. Cognitive deficits of pure subcortical vascular dementia vs. Alzheimer disease: PiB-PET-based study. Neurology. 2013; 80(6):569–573. [PubMed: 23325910]

163. Piguet O, Hornberger M, Mioshi E, Hodges JR. Behavioural-variant frontotemporal dementia: diagnosis, clinical staging, and management. Lancet Neurol. 2011; 10(2):162–172. [PubMed: 21147039]

164. Seelaar H, Rohrer JD, Pijnenburg YA, Fox NC, van Swieten JC. Clinical, genetic and pathological heterogeneity of frontotemporal dementia: a review. J Neurol Neurosurg Psychiatry. 2011; 82(5):476–486. [PubMed: 20971753]

165. Krueger CE, Dean DL, Rosen HJ, et al. Longitudinal rates of lobar atrophy in frontotemporal dementia, semantic dementia, and Alzheimer's disease. Alzheimer Dis Assoc Disord. 2010; 24(1):43–48. [PubMed: 19571735]

166. Whitwell JL, Jack CR Jr, Parisi JE, et al. Imaging signatures of molecular pathology in behavioral variant frontotemporal dementia. J Mol Neurosci. 2011; 45(3):372–378. [PubMed: 21556732]

167. Whitwell JL, Josephs KA, Rossor MN, et al. Magnetic resonance imaging signatures of tissue pathology in frontotemporal dementia. Arch Neurol. 2005; 62(9):1402–1408. [PubMed: 16157747]

168. Ghetti B, Spina S, Murrell JR, et al. In vivo and postmortem clinicoanatomical correlations in frontotemporal dementia and parkinsonism linked to chromosome 17. Neurodegener Dis. 2008; 5(3–4):215–217. [PubMed: 18322394]

169. Whitwell JL, Przybelski SA, Weigand SD, et al. Distinct anatomical subtypes of the behavioural variant of frontotemporal dementia: a cluster analysis study. Brain. 2009; 132(Pt 11):2932–2946. [PubMed: 19762452]

170. Whitwell JL, Jack CR Jr, Parisi JE, et al. Does TDP-43 type confer a distinct pattern of atrophy in frontotemporal lobar degeneration? Neurology. 2010; 75(24):2212–2220. [PubMed: 21172844]

171. Rohrer JD, Geser F, Zhou J, et al. TDP-43 subtypes are associated with distinct atrophy patterns in frontotemporal dementia. Neurology. 2010; 75(24):2204–2211. [PubMed: 21172843]

172. Rohrer JD, Lashley T, Schott JM, et al. Clinical and neuroanatomical signatures of tissue pathology in frontotemporal lobar degeneration. Brain. 2011; 134(Pt 9):2565–2581. [PubMed: 21908872]

NIH-PA Author Manuscript NIH-PA Author Manuscript NIH-PA Author Manuscript

173. Josephs KA, Whitwell JL, Parisi JE, et al. Caudate atrophy on MRI is a characteristic feature of FTLD-FUS. Eur J Neurol. 2010; 17(7):969–975. [PubMed: 20236174]
174. Zhang Y, Schuff N, Ching C, et al. Joint assessment of structural, perfusion, and diffusion MRI in Alzheimer's disease and frontotemporal dementia. Int J Alzheimers Dis. 2011; 2011:546871. [PubMed: 21760989]
175. Whitwell JL, Avula R, Senjem ML, et al. Gray and white matter water diffusion in the syndromic variants of frontotemporal dementia. Neurology. 2010; 74(16):1279–1287. [PubMed: 20404309]
176. Ebmeier KP, Filippini N, Heise V, Sexton CE. Other magnetic resonance imaging techniques. Int Psychogeriatr. 2011; 23 (Suppl 2):S50–S57. [PubMed: 21843379]
177. Dopper EG, Rombouts SA, Jiskoot LC, et al. Structural and functional brain connectivity in presymptomatic familial frontotemporal dementia. Neurology. 2013; 80(9):814–823. [PubMed: 23390180]
178. Rombouts SA, van Swieten JC, Pijnenburg YA, Goekoop R, Barkhof F, Scheltens P. Loss of frontal fMRI activation in early frontotemporal dementia compared to early AD. Neurology. 2003; 60(12):1904–1908. [PubMed: 12821731]
179. Virani K, Jesso S, Kertesz A, Mitchell D, Finger E. Functional neural correlates of emotional expression processing deficits in behavioural variant frontotemporal dementia. J Psychiatry Neurosci. 2013; 38(3):174–182. [PubMed: 23031250]
180. Seeley WW, Menon V, Schatzberg AF, et al. Dissociable intrinsic connectivity networks for salience processing and executive control. J Neurosci. 2007; 27(9):2349–2356. [PubMed: 17329432]
181. Pievani M, de Haan W, Wu T, Seeley WW, Frisoni GB. Functional network disruption in the degenerative dementias. Lancet Neurol. 2011; 10(9):829–843. [PubMed: 21778116]
182. Filippi M, Agosta F, Scola E, et al. Functional network connectivity in the behavioral variant of frontotemporal dementia. Cortex. 2012
183. Seeley WW, Crawford R, Rascovsky K, et al. Frontal paralimbic network atrophy in very mild behavioral variant frontotemporal dementia. Arch Neurol. 2008; 65(2):249–255. [PubMed: 18268196]
184. Zhou J, Greicius MD, Gennatas ED, et al. Divergent network connectivity changes in behavioural variant frontotemporal dementia and Alzheimer's disease. Brain. 2010; 133(Pt 5):1352–1367. [PubMed: 20410145]
185. Whitwell JL, Josephs KA, Avula R, et al. Altered functional connectivity in asymptomatic MAPT subjects: a comparison to bvFTD. Neurology. 2011; 77(9):866–874. [PubMed: 21849646]
186. Du AT, Jahng GH, Hayasaka S, et al. Hypoperfusion in frontotemporal dementia and Alzheimer disease by arterial spin labeling MRI. Neurology. 2006; 67(7):1215–1220. [PubMed: 17030755]
187. Mummery CJ, Patterson K, Price CJ, Ashburner J, Frackowiak RS, Hodges JR. A voxel-based morphometry study of semantic dementia: relationship between temporal lobe atrophy and semantic memory. Ann Neurol. 2000; 47(1):36–45. [PubMed: 10632099]
188. Bonner MF, Vesely L, Price C, et al. Reversal of the concreteness effect in semantic dementia. Cogn Neuropsychol. 2009; 26(6):568–579. [PubMed: 20183015]
189. Hodges JR, Patterson K. Semantic dementia: a unique clinicopathological syndrome. Lancet Neurol. 2007; 6(11):1004–1014. [PubMed: 17945154]
190. Rohrer JD, Warren JD, Modat M, et al. Patterns of cortical thinning in the language variants of frontotemporal lobar degeneration. Neurology. 2009; 72(18):1562–1569. [PubMed: 19414722]
191. Czarnecki K, Duffy JR, Nehl CR, et al. Very early semantic dementia with progressive temporal lobe atrophy: an 8-year longitudinal study. Arch Neurol. 2008; 65(12):1659–1663. [PubMed: 19064755]
192. Maguire EA, Kumaran D, Hassabis D, Kopelman MD. Autobiographical memory in semantic dementia: a longitudinal fMRI study. Neuropsychologia. 2010; 48(1):123–136. [PubMed: 19720072]
193. Goll JC, Ridgway GR, Crutch SJ, Theunissen FE, Warren JD. Nonverbal sound processing in semantic dementia: a functional MRI study. Neuroimage. 2012; 61(1):170–180. [PubMed: 22405732]

NIH-PA Author Manuscript NIH-PA Author Manuscript NIH-PA Author Manuscript

194. Wilson SM, Brambati SM, Henry RG, et al. The neural basis of surface dyslexia in semantic dementia. Brain. 2009; 132(Pt 1):71–86. [PubMed: 19022856]

195. Farb NA, Grady CL, Strother S, et al. Abnormal network connectivity in frontotemporal dementia: evidence for prefrontal isolation. Cortex. 2013; 49(7):1856–1873. [PubMed: 23092697]

196. Josephs KA, Duffy JR, Strand EA, et al. Clinicopathological and imaging correlates of progressive aphasia and apraxia of speech. Brain. 2006; 129(Pt 6):1385–1398. [PubMed: 16613895]

197. Peelle JE, Troiani V, Gee J, et al. Sentence comprehension and voxel-based morphometry in progressive nonfluent aphasia, semantic dementia, and nonaphasic frontotemporal dementia. J Neurolinguist. 2008; 21(5):418–432.

198. Cooke A, DeVita C, Gee J, et al. Neural basis for sentence comprehension deficits in frontotemporal dementia. Brain Lang. 2003; 85(2):211–221. [PubMed: 12735939]

199. Caso F, Gesierich B, Henry M, et al. Nonfluent/agrammatic PPA with in-vivo cortical amyloidosis and Pick's disease pathology. Behav Neurol. 2013; 26(1–2):95–106. [PubMed: 22713404]

200. Nestor PJ, Graham NL, Fryer TD, Williams GB, Patterson K, Hodges JR. Progressive non-fluent aphasia is associated with hypometabolism centred on the left anterior insula. Brain. 2003; 126(Pt 11):2406–2418. [PubMed: 12902311]

201. Gil-Navarro S, Lomeña F, Cot A, et al. Decreased striatal dopamine transporter uptake in the non-fluent/agrammatic variant of primary progressive aphasia. Eur J Neurol. 2013

202. Josephs KA, Whitwell JL, Dickson DW, et al. Voxel-based morphometry in autopsy proven PSP and CBD. Neurobiol Aging. 2008; 29(2):280–289. [PubMed: 17097770]

203. Whitwell JL, Jack CR Jr, Parisi JE, et al. Rates of cerebral atrophy differ in different degenerative pathologies. Brain. 2007; 130(Pt 4):1148–1158. [PubMed: 17347250]

204. Tokumaru AM, Saito Y, Murayama S, et al. Imaging-pathologic correlation in corticobasal degeneration. AJNR Am J Neuroradiol. 2009; 30(10):1884–1892. [PubMed: 19833793]

205. Erbetta A, Mandelli ML, Savoiardo M, et al. Diffusion tensor imaging shows different topographic involvement of the thalamus in progressive supranuclear palsy and corticobasal degeneration. AJNR Am J Neuroradiol. 2009; 30(8):1482–1487. [PubMed: 19589886]

206. Dashjamts T, Yoshiura T, Hiwatashi A, et al. Asymmetrical cerebral perfusion demonstrated by noninvasive arterial spin-labeling perfusion imaging in a patient with corticobasal degeneration. Jpn J Radiol. 2010; 28(1):75–78. [PubMed: 20112098]

207. Cilia R, Rossi C, Frosini D, et al. Dopamine Transporter SPECT Imaging in Corticobasal Syndrome. PLoS ONE. 2011; 6(5):e18301. [PubMed: 21559307]

208. Mazère J, Meissner WG, Mayo W, et al. Progressive supranuclear palsy: in vivo SPECT imaging of presynaptic vesicular acetylcholine transporter with [123I]-iodobenzovesamicol. Radiology. 2012; 265(2):537–543. [PubMed: 23012462]

209. Teune LK, Bartels AL, de Jong BM, et al. Typical cerebral metabolic patterns in neurodegenerative brain diseases. Mov Disord. 2010; 25(14):2395–2404. [PubMed: 20669302]

210. Kuwahara H, Tsuchiya K, Saito Y, et al. Frontotemporal lobar degeneration with motor neuron disease showing severe and circumscribed atrophy of anterior temporal lobes. J Neurol Sci. 2010; 297(1–2):92–96. [PubMed: 20674934]

211. Turner MR, Agosta F, Bede P, Govind V, Lulé D, Verstraete E. Neuroimaging in amyotrophic lateral sclerosis. Biomarkers Med. 2012; 6(3):319–337.

212. Sage CA, Peeters RR, Görner A, Robberecht W, Sunaert S. Quantitative diffusion tensor imaging in amyotrophic lateral sclerosis. Neuroimage. 2007; 34(2):486–499. [PubMed: 17097892]

213. Sage CA, Van Hecke W, Peeters R, et al. Quantitative diffusion tensor imaging in amyotrophic lateral sclerosis: revisited. Hum Brain Mapp. 2009; 30(11):3657–3675. [PubMed: 19404990]

214. Sarro L, Agosta F, Canu E, et al. Cognitive functions and white matter tract damage in amyotrophic lateral sclerosis: a diffusion tensor tractography study. AJNR Am J Neuroradiol. 2011; 32(10):1866–1872. [PubMed: 22016410]

215. Abrahams S, Goldstein LH, Kew JJ, et al. Frontal lobe dysfunction in amyotrophic lateral sclerosis. A PET study. Brain. 1996; 119(Pt 6):2105–2120. [PubMed: 9010014]

NIH-PA Author Manuscript NIH-PA Author Manuscript NIH-PA Author Manuscript

216. Abrahams S, Leigh PN, Kew JJ, Goldstein LH, Lloyd CM, Brooks DJ. A positron emission tomography study of frontal lobe function (verbal fluency) in amyotrophic lateral sclerosis. J Neurol Sci. 1995; 129(Suppl):44–46. [PubMed: 7595618]

217. Abrahams S, Goldstein LH, Simmons A, et al. Word retrieval in amyotrophic lateral sclerosis: a functional magnetic resonance imaging study. Brain. 2004; 127(Pt 7):1507–1517. [PubMed: 15163610]

218. Palmieri A, Naccarato M, Abrahams S, et al. Right hemisphere dysfunction and emotional processing in ALS: an fMRI study. J Neurol. 2010; 257(12):1970–1978. [PubMed: 20593194]

219. Mohammadi B, Kollewe K, Samii A, Krampfl K, Dengler R, Münte TF. Changes of resting state brain networks in amyotrophic lateral sclerosis. Exp Neurol. 2009; 217(1):147–153. [PubMed: 19416664]

220. Guedj E, Le Ber I, Lacomblez L, et al. French Research Network on FTD/FTD-MND. Brain spect perfusion of frontotemporal dementia associated with motor neuron disease. Neurology. 2007; 69(5):488–490. [PubMed: 17664410]

221. Talbot PR, Goulding PJ, Lloyd JJ, Snowden JS, Neary D, Testa HJ. Inter-relation between “classic” motor neuron disease and frontotemporal dementia: neuropsychological and single photon emission computed tomography study. J Neurol Neurosurg Psychiatry. 1995; 58(5):541–547. [PubMed: 7745399]

222. Ishikawa T, Morita M, Nakano I. Constant blood flow reduction in premotor frontal lobe regions in ALS with dementia - a SPECT study with 3D-SSP. Acta Neurol Scand. 2007; 116(5):340–344. [PubMed: 17922728]

223. Garraux G, Salmon E, Degueldre C, Lemaire C, Franck G. Medial temporal lobe metabolic impairment in dementia associated with motor neuron disease. J Neurol Sci. 1999; 168(2):145–150. [PubMed: 10526199]

224. Jeong Y, Park KC, Cho SS, et al. Pattern of glucose hypometabolism in frontotemporal dementia with motor neuron disease. Neurology. 2005; 64(4):734–736. [PubMed: 15728305]

225. Ludolph AC, Langen KJ, Regard M, et al. Frontal lobe function in amyotrophic lateral sclerosis: a neuropsychologic and positron emission tomography study. Acta Neurol Scand. 1992; 85(2):81–89. [PubMed: 1574993]

226. Lloyd CM, Richardson MP, Brooks DJ, Al-Chalabi A, Leigh PN. Extramotor involvement in ALS: PET studies with the GABA(A) ligand [(11)C]flumazenil. Brain. 2000; 123(Pt 11):2289–2296. [PubMed: 11050028]

227. Sha SJ, Takada LT, Rankin KP, et al. Frontotemporal dementia due to C9ORF72 mutations: clinical and imaging features. Neurology. 2012; 79(10):1002–1011. [PubMed: 22875087]

228. Yokoyama JS, Rosen HJ. Neuroimaging features of C9ORF72 expansion. Alzheimers Res Ther. 2012; 4(6):45. [PubMed: 23153366]

229. Kassubek J, Unrath A, Huppertz HJ, et al. Global brain atrophy and corticospinal tract alterations in ALS, as investigated by voxel-based morphometry of 3-D MRI. Amyotroph Lateral Scler Other Motor Neuron Disord. 2005; 6(4):213–220. [PubMed: 16319024]

230. Chen Z, Ma L. Grey matter volume changes over the whole brain in amyotrophic lateral sclerosis: A voxel-wise meta-analysis of voxel based morphometry studies. Amyotroph Lateral Scler. 2010; 11(6):549–554. [PubMed: 20929296]

231. Roccatagliata L, Bonzano L, Mancardi G, Canepa C, Caponnetto C. Detection of motor cortex thinning and corticospinal tract involvement by quantitative MRI in amyotrophic lateral sclerosis. Amyotroph Lateral Scler. 2009; 10(1):47–52. [PubMed: 18622772]

232. Filippini N, Douaud G, Mackay CE, Knight S, Talbot K, Turner MR. Corpus callosum involvement is a consistent feature of amyotrophic lateral sclerosis. Neurology. 2010; 75(18): 1645–1652. [PubMed: 21041787]

233. Menke RA, Abraham I, Thiel CS, et al. Fractional anisotropy in the posterior limb of the internal capsule and prognosis in amyotrophic lateral sclerosis. Arch Neurol. 2012; 69(11):1493–1499. [PubMed: 22910997]

234. Li J, Pan P, Song W, Huang R, Chen K, Shang H. A meta-analysis of diffusion tensor imaging studies in amyotrophic lateral sclerosis. Neurobiol Aging. 2012; 33(8):1833–1838. [PubMed: 21621298]

NIH-PA Author Manuscript
NIH-PA Author Manuscript
NIH-PA Author Manuscript

235. Konrad C, Henningsen H, Bremer J, et al. Pattern of cortical reorganization in amyotrophic lateral sclerosis: a functional magnetic resonance imaging study. Exp Brain Res. 2002; 143(1):51–56. [PubMed: 11907690]

236. Han J, Ma L. Functional magnetic resonance imaging study of the brain in patients with amyotrophic lateral sclerosis. Chin Med Sci J. 2006; 21(4):228–233. [PubMed: 17249197]

237. Stanton BR, Williams VC, Leigh PN, et al. Altered cortical activation during a motor task in ALS. Evidence for involvement of central pathways. J Neurol. 2007; 254(9):1260–1267. [PubMed: 17385077]

238. Konrad C, Jansen A, Henningsen H, et al. Subcortical reorganization in amyotrophic lateral sclerosis. Exp Brain Res. 2006; 172(3):361–369. [PubMed: 16463149]

239. Lulé D, Diekmann V, Müller HP, Kassubek J, Ludolph AC, Birbaumer N. Neuroimaging of multimodal sensory stimulation in amyotrophic lateral sclerosis. J Neurol Neurosurg Psychiatry. 2010; 81(8):899–906. [PubMed: 20543183]

240. Jelsone-Swain LM, Fling BW, Seidler RD, Hovatter R, Gruis K, Welsh RC. Reduced Interhemispheric Functional Connectivity in the Motor Cortex during Rest in Limb-Onset Amyotrophic Lateral Sclerosis. Front Syst Neurosci. 2010; 4:158. [PubMed: 21228916]

241. Agosta F, Valsasina P, Absinta M, et al. Sensorimotor functional connectivity changes in amyotrophic lateral sclerosis. Cereb Cortex. 2011; 21(10):2291–2298. [PubMed: 21368084]

242. Han J, Ma L. Study of the features of proton MR spectroscopy ((1) H-MRS) on amyotrophic lateral sclerosis. J Magn Reson Imaging. 2010; 31(2):305–308. [PubMed: 20099342]

243. Schuff N, Rooney WD, Miller R, et al. Reanalysis of multislice (1)H MRSI in amyotrophic lateral sclerosis. Magn Reson Med. 2001; 45(3):513–516. [PubMed: 11241711]

244. Yin H, Lim CC, Ma L, et al. Combined MR spectroscopic imaging and diffusion tensor MRI visualizes corticospinal tract degeneration in amyotrophic lateral sclerosis. J Neurol. 2004; 251(10):1249–1254. [PubMed: 15503106]

245. Lombardo F, Frijia F, Bongioanni P, et al. Diffusion tensor MRI and MR spectroscopy in long lasting upper motor neuron involvement in amyotrophic lateral sclerosis. Arch Ital Biol. 2009; 147(3):69–82. [PubMed: 20014653]

246. Ludolph AC, Elger CE, Böttger IW, Kuttig AG, Lottes G, Brune GG. N-isopropyl-p-123I-amphetamine single photon emission computer tomography in motor neuron disease. Eur Neurol. 1989; 29(5):255–260. [PubMed: 2792143]

247. Tanaka M, Kondo S, Hirai S, Sun X, Yamagishi T, Okamoto K. Cerebral blood flow and oxygen metabolism in progressive dementia associated with amyotrophic lateral sclerosis. J Neurol Sci. 1993; 120(1):22–28. [PubMed: 8289076]

248. Kew JJ, Goldstein LH, Leigh PN, et al. The relationship between abnormalities of cognitive function and cerebral activation in amyotrophic lateral sclerosis. A neuropsychological and positron emission tomography study. Brain. 1993; 116(Pt 6):1399–1423. [PubMed: 8293278]

249. Takahashi H, Snow BJ, Bhatt MH, Peppard R, Eisen A, Calne DB. Evidence for a dopaminergic deficit in sporadic amyotrophic lateral sclerosis on positron emission scanning. Lancet. 1993; 342(8878):1016–1018. [PubMed: 8105264]

250. Turner MR, Gerhard A, Al-Chalabi A, et al. Mills' and other isolated upper motor neurone syndromes: in vivo study with 11C-(R)-PK11195 PET. J Neurol Neurosurg Psychiatry. 2005; 76(6):871–874. [PubMed: 15897516]

251. Turner MR, Cagnin A, Turkheimer FE, et al. Evidence of widespread cerebral microglial activation in amyotrophic lateral sclerosis: an [11C](R)-PK11195 positron emission tomography study. Neurobiol Dis. 2004; 15(3):601–609. [PubMed: 15056468]

252. Monchi O, Degroot C, Mejia-Constain B, Bruneau MA. Neuroimaging studies of different cognitive profiles in Parkinson's disease. Parkinsonism Relat Disord. 2012; 18(Suppl 1):S77–S79. [PubMed: 22166462]

253. Tateno M, Kobayashi S, Saito T. Imaging improves diagnosis of dementia with lewy bodies. Psychiatry Investig. 2009; 6(4):233–240.

254. Hashimoto M, Kitagaki H, Imamura T, et al. Medial temporal and whole-brain atrophy in dementia with Lewy bodies: a volumetric MRI study. Neurology. 1998; 51(2):357–362. [PubMed: 9710003]

NIH-PA Author Manuscript
NIH-PA Author Manuscript
NIH-PA Author Manuscript

255. Burton EJ, Karas G, Paling SM, et al. Patterns of cerebral atrophy in dementia with Lewy bodies using voxel-based morphometry. Neuroimage. 2002; 17(2):618–630. [PubMed: 12377138]
256. Ballmaier M, O'Brien JT, Burton EJ, et al. Comparing gray matter loss profiles between dementia with Lewy bodies and Alzheimer's disease using cortical pattern matching: diagnosis and gender effects. Neuroimage. 2004; 23(1):325–335. [PubMed: 15325380]
257. Burton EJ, McKeith IG, Burn DJ, Williams ED, O'Brien JT. Cerebral atrophy in Parkinson's disease with and without dementia: a comparison with Alzheimer's disease, dementia with Lewy bodies and controls. Brain. 2004; 127(Pt 4):791–800. [PubMed: 14749292]
258. Johansen KK, White LR, Sando SB, Aasly JO. Biomarkers: Parkinson disease with dementia and dementia with Lewy bodies. Parkinsonism Relat Disord. 2010; 16(5):307–315. [PubMed: 20338799]
259. Beyer MK, Larsen JP, Aarsland D. Gray matter atrophy in Parkinson disease with dementia and dementia with Lewy bodies. Neurology. 2007; 69(8):747–754. [PubMed: 17709706]
260. Shimada H, Shinotoh H, Hirano S, et al. β-Amyloid in Lewy body disease is related to Alzheimer's disease-like atrophy. Mov Disord. 2013; 28(2):169–175. [PubMed: 23225334]
261. Burton EJ, McKeith IG, Burn DJ, O'Brien JT. Brain atrophy rates in Parkinson's disease with and without dementia using serial magnetic resonance imaging. Mov Disord. 2005; 20(12):1571–1576. [PubMed: 16116613]
262. O'Brien JT, Paling S, Barber R, et al. Progressive brain atrophy on serial MRI in dementia with Lewy bodies, AD, and vascular dementia. Neurology. 2001; 56(10):1386–1388. [PubMed: 11376193]
263. Lee JE, Park HJ, Park B, et al. A comparative analysis of cognitive profiles and white-matter alterations using voxel-based diffusion tensor imaging between patients with Parkinson's disease dementia and dementia with Lewy bodies. J Neurol Neurosurg Psychiatry. 2010; 81(3):320–326. [PubMed: 19828477]
264. Bozzali M, Cherubini A. Diffusion tensor MRI to investigate dementias: a brief review. Magn Reson Imaging. 2007; 25(6):969–977. [PubMed: 17451903]
265. Bozzali M, Falini A, Cercignani M, et al. Brain tissue damage in dementia with Lewy bodies: an in vivo diffusion tensor MRI study. Brain. 2005; 128(Pt 7):1595–1604. [PubMed: 15817515]
266. Firbank MJ, Blamire AM, Krishnan MS, et al. Atrophy is associated with posterior cingulate white matter disruption in dementia with Lewy bodies and Alzheimer's disease. Neuroimage. 2007; 36(1):1–7. [PubMed: 17412610]
267. Matsui H, Nishinaka K, Oda M, Niikawa H, Kubori T, Udaka F. Dementia in Parkinson's disease: diffusion tensor imaging. Acta Neurol Scand. 2007; 116(3):177–181. [PubMed: 17714331]
268. Griffith HR, den Hollander JA, Okonkwo OC, O'Brien T, Watts RL, Marson DC. Brain metabolism differs in Alzheimer's disease and Parkinson's disease dementia. Alzheimers Dement. 2008; 4(6):421–427. [PubMed: 19012867]
269. Griffith HR, den Hollander JA, Okonkwo OC, O'Brien T, Watts RL, Marson DC. Brain N-acetylaspartate is reduced in Parkinson disease with dementia. Alzheimer Dis Assoc Disord. 2008; 22(1):54–60. [PubMed: 18317247]
270. Griffith HR, Okonkwo OC, O'Brien T, Hollander JA. Reduced brain glutamate in patients with Parkinson's disease. NMR Biomed. 2008; 21(4):381–387. [PubMed: 17907264]
271. Sauer J, ffytche DH, Ballard C, Brown RG, Howard R. Differences between Alzheimer's disease and dementia with Lewy bodies: an fMRI study of task-related brain activity. Brain. 2006; 129(Pt 7):1780–1788. [PubMed: 16670180]
272. Taylor JP, Firbank MJ, He J, et al. Visual cortex in dementia with Lewy bodies: magnetic resonance imaging study. Br J Psychiatry. 2012; 200(6):491–498. [PubMed: 22500014]
273. Monchi O, Petrides M, Doyon J, Postuma RB, Worsley K, Dagher A. Neural bases of set-shifting deficits in Parkinson's disease. J Neurosci. 2004; 24(3):702–710. [PubMed: 14736856]
274. Galvin JE, Price JL, Yan Z, Morris JC, Sheline YI. Resting bold fMRI differentiates dementia with Lewy bodies vs Alzheimer disease. Neurology. 2011; 76(21):1797–1803. [PubMed: 21525427]

NIH-PA Author Manuscript NIH-PA Author Manuscript NIH-PA Author Manuscript

275. Torosyan N, Silverman DH. Neuronuclear imaging in the evaluation of dementia and mild decline in cognition. Semin Nucl Med. 2012; 42(6):415–422. [PubMed: 23026363]

276. Kantarci K, Lowe VJ, Boeve BF, et al. Multimodality imaging characteristics of dementia with Lewy bodies. Neurobiol Aging. 2012; 33(9):2091–2105. [PubMed: 22018896]

277. Gilman S, Koeppe RA, Little R, et al. Differentiation of Alzheimer's disease from dementia with Lewy bodies utilizing positron emission tomography with [18F]fluorodeoxyglucose and neuropsychological testing. Exp Neurol. 2005; 191(Suppl 1):S95–S103. [PubMed: 15629765]

278. Peppard RF, Martin WR, Carr GD, et al. Cerebral glucose metabolism in Parkinson's disease with and without dementia. Arch Neurol. 1992; 49(12):1262–1268. [PubMed: 1449406]

279. Schapiro MB, Pietrini P, Grady CL, et al. Reductions in parietal and temporal cerebral metabolic rates for glucose are not specific for Alzheimer's disease. J Neurol Neurosurg Psychiatry. 1993; 56(8):859–864. [PubMed: 8350100]

280. Vander Borght T, Minoshima S, Giordani B, et al. Cerebral metabolic differences in Parkinson's and Alzheimer's diseases matched for dementia severity. J Nucl Med. 1997; 38(5):797–802. [PubMed: 9170449]

281. O'Brien JT, Colloby S, Fenwick J, et al. Dopamine transporter loss visualized with FP-CIT SPECT in the differential diagnosis of dementia with Lewy bodies. Arch Neurol. 2004; 61(6): 919–925. [PubMed: 15210531]

282. Rinne JO, Portin R, Ruottinen H, et al. Cognitive impairment and the brain dopaminergic system in Parkinson disease: [18F]fluorodopa positron emission tomographic study. Arch Neurol. 2000; 57(4):470–475. [PubMed: 10768619]

283. Brooks DJ, Pavese N. Imaging biomarkers in Parkinson's disease. Prog Neurobiol. 2011; 95(4): 614–628. [PubMed: 21896306]

284. Mori T, Maeda J, Shimada H, et al. Molecular imaging of dementia. Psychogeriatrics. 2012; 12(2):106–114. [PubMed: 22712644]

285. Bohnen NI, Kaufer DI, Hendrickson R, et al. Cognitive correlates of cortical cholinergic denervation in Parkinson's disease and parkinsonian dementia. J Neurol. 2006; 253(2):242–247. [PubMed: 16133720]

286. Bohnen NI, Kaufer DI, Ivanco LS, et al. Cortical cholinergic function is more severely affected in parkinsonian dementia than in Alzheimer disease: an in vivo positron emission tomographic study. Arch Neurol. 2003; 60(12):1745–1748. [PubMed: 14676050]

287. Hilker R, Thomas AV, Klein JC, et al. Dementia in Parkinson disease: functional imaging of cholinergic and dopaminergic pathways. Neurology. 2005; 65(11):1716–1722. [PubMed: 16344512]

288. Bouchard TP, Malykhin N, Martin WR, et al. Age and dementia-associated atrophy predominates in the hippocampal head and amygdala in Parkinson's disease. Neurobiol Aging. 2008; 29(7): 1027–1039. [PubMed: 17368653]

289. Brück A, Kurki T, Kaasinen V, Vahlberg T, Rinne JO. Hippocampal and prefrontal atrophy in patients with early non-demented Parkinson's disease is related to cognitive impairment. J Neurol Neurosurg Psychiatry. 2004; 75(10):1467–1469. [PubMed: 15377698]

290. Camicioli R, Moore MM, Kinney A, Corbridge E, Glassberg K, Kaye JA. Parkinson's disease is associated with hippocampal atrophy. Mov Disord. 2003; 18(7):784–790. [PubMed: 12815657]

291. Hu MT, White SJ, Chaudhuri KR, Morris RG, Bydder gmatter, Brooks DJ. Correlating rates of cerebral atrophy in Parkinson's disease with measures of cognitive decline. J Neural Transm. 2001; 108(5):571–580. [PubMed: 11459077]

292. Hu MT, Taylor-Robinson SD, Chaudhuri KR, et al. Cortical dysfunction in non-demented Parkinson's disease patients: a combined (31)P-MRS and (18)FDG-PET study. Brain. 2000; 123(Pt 2):340–352. [PubMed: 10648441]

293. Huang C, Tang C, Feigin A, et al. Changes in network activity with the progression of Parkinson's disease. Brain. 2007; 130(Pt 7):1834–1846. [PubMed: 17470495]

294. Fearnley JM, Lees AJ. Ageing and Parkinson's disease: substantia nigra regional selectivity. Brain. 1991; 114(Pt 5):2283–2301. [PubMed: 1933245]

NIH-PA Author Manuscript NIH-PA Author Manuscript NIH-PA Author Manuscript

295. Morrish PK, Rakshi JS, Bailey DL, Sawle GV, Brooks DJ. Measuring the rate of progression and estimating the preclinical period of Parkinson's disease with [18F]dopa PET. J Neurol Neurosurg Psychiatry. 1998; 64(3):314–319. [PubMed: 9527140]
296. Rinne JO, Laihinen A, Rinne UK, Någren K, Bergman J, Ruotsalainen U. PET study on striatal dopamine D2 receptor changes during the progression of early Parkinson's disease. Mov Disord. 1993; 8(2):134–138. [PubMed: 8474478]
297. Sawle GV. The detection of preclinical Parkinson's disease: what is the role of positron emission tomography? Mov Disord. 1993; 8(3):271–277. [PubMed: 8341290]
298. Kaasinen V, Någren K, Hietala J, et al. Extrastriatal dopamine D2 and D3 receptors in early and advanced Parkinson's disease. Neurology. 2000; 54(7):1482–1487. [PubMed: 10751262]
299. Kaasinen V, Aalto S, Nagren K, Hietala J, Sonninen P, Rinne JO. Extrastriatal dopamine D(2) receptors in Parkinson's disease: a longitudinal study. J Neural Transm. 2003; 110(6):591–601. [PubMed: 12768355]
300. Guttman M, Boileau I, Warsh J, et al. Brain serotonin transporter binding in non-depressed patientswith Parkinson's disease. Eur J Neurol. 2007; 14(5):523–528. [PubMed: 17437611]
301. Asahina M, Suhara T, Shinotoh H, Inoue O, Suzuki K, Hattori T. Brain muscarinic receptors in progressive supranuclear palsy and Parkinson's disease: a positron emission tomographic study. J Neurol Neurosurg Psychiatry. 1998; 65(2):155–163. [PubMed: 9703164]
302. Shtilbans A, Henchcliffe C. Biomarkers in Parkinson's disease: an update. Curr Opin Neurol. 2012; 25(4):460–465. [PubMed: 22772875]
303. Piccini P, Weeks RA, Brooks DJ. Alterations in opioid receptor binding in Parkinson's disease patients with levodopa-induced dyskinesias. Ann Neurol. 1997; 42(5):720–726. [PubMed: 9392571]
304. Gerhard A, Pavese N, Hotton G, et al. In vivo imaging of microglial activation with [11C](R)-PK11195 PET in idiopathic Parkinson's disease. Neurobiol Dis. 2006; 21(2):404–412. [PubMed: 16182554]
305. Ouchi Y, Yoshikawa E, Sekine Y, et al. Microglial activation and dopamine terminal loss in early Parkinson's disease. Ann Neurol. 2005; 57(2):168–175. [PubMed: 15668962]
306. Georgiou-Karistianis N, Scahill R, Tabrizi SJ, Squitieri F, Aylward E. Structural MRI in Huntington's disease and recommendations for its potential use in clinical trials. Neurosci Biobehav Rev. 2013; 37(3):480–490. [PubMed: 23376047]
307. Paulsen JS, Langbehn DR, Stout JC, et al. Predict-HD Investigators and Coordinators of the Huntington Study Group. Detection of Huntington's disease decades before diagnosis: the Predict-HD study. J Neurol Neurosurg Psychiatry. 2008; 79(8):874–880. [PubMed: 18096682]
308. Paulsen JS, Nopoulos PC, Aylward E, et al. PREDICT-HD Investigators and Coordinators of the Huntington's Study Group (HSG). Striatal and white matter predictors of estimated diagnosis for Huntington disease. Brain Res Bull. 2010; 82(3–4):201–207. [PubMed: 20385209]
309. Tabrizi SJ, Langbehn DR, Leavitt BR, et al. TRACK-HD investigators. Biological and clinical manifestations of Huntington's disease in the longitudinal TRACK-HD study: cross-sectional analysis of baseline data. Lancet Neurol. 2009; 8(9):791–801. [PubMed: 19646924]
310. Tabrizi SJ, Reilmann R, Roos RA, et al. TRACK-HD investigators. Potential endpoints for clinical trials in premanifest and early Huntington's disease in the TRACK-HD study: analysis of 24. month observational data. Lancet Neurol. 2012; 11(1):42–53. [PubMed: 22137354]
311. Majid DS, Aron AR, Thompson W, et al. Basal ganglia atrophy in prodromal Huntington's disease is detectable over one year using automated segmentation. Mov Disord. 2011; 26(14): 2544–2551. [PubMed: 21932302]
312. Tabrizi SJ, Scahill RI, Durr A, et al. TRACK-HD Investigators. Biological and clinical changes in premanifest and early stage Huntington's disease in the TRACK-HD study: the 12-month longitudinal analysis. Lancet Neurol. 2011; 10(1):31–42. [PubMed: 21130037]
313. Henley SM, Wild EJ, Hobbs NZ, et al. Whole-brain atrophy as a measure of progression in premanifest and early Huntington's disease. Mov Disord. 2009; 24(6):932–936. [PubMed: 19243073]

NIH-PA Author Manuscript
NIH-PA Author Manuscript
NIH-PA Author Manuscript

314. Hobbs NZ, Pedrick AV, Say MJ, et al. The structural involvement of the cingulate cortex in premanifest and early Huntington's disease. Mov Disord. 2011; 26(9):1684–1690. [PubMed: 21557312]

315. Rupp J, Dzemidzic M, Blekher T, et al. Comparison of vertical and horizontal saccade measures and their relation to gray matter changes in premanifest and manifest Huntington disease. J Neurol. 2012; 259(2):267–276. [PubMed: 21850389]

316. Magnotta VA, Kim J, Koscik T, et al. Diffusion Tensor Imaging in Preclinical Huntington's Disease. Brain Imaging Behav. 2009; 3(1):77–84. [PubMed: 21415933]

317. Reading SA, Yassa MA, Bakker A, et al. Regional white matter change in pre-symptomatic Huntington's disease: a diffusion tensor imaging study. Psychiatry Res. 2005; 140(1):55–62. [PubMed: 16199141]

318. Rosas HD, Tuch DS, Hevelone ND, et al. Diffusion tensor imaging in presymptomatic and early Huntington's disease: Selective white matter pathology and its relationship to clinical measures. Mov Disord. 2006 Sep; 21(9):1317–1325. [PubMed: 16755582]

319. Klöppel S, Henley SM, Hobbs NZ, et al. Magnetic resonance imaging of Huntington's disease: preparing for clinical trials. Neuroscience. 2009; 164(1):205–219. [PubMed: 19409230]

320. Rupp J, Dzemidzic M, Blekher T, et al. Abnormal error-related antisaccade activation in premanifest and early manifest Huntington disease. Neuropsychology. 2011; 25(3):306–318. [PubMed: 21401260]

321. Unschuld PG, Joel SE, Liu X, et al. Impaired cortico-striatal functional connectivity in prodromal Huntington's Disease. Neurosci Lett. 2012; 514(2):204–209. [PubMed: 22425717]

322. Wolf RC, Sambataro F, Vasic N, et al. Default-mode network changes in preclinical Huntington's disease. Exp Neurol. 2012; 237(1):191–198. [PubMed: 22742947]

323. Weeks RA, Ceballos-Baumann A, Piccini P, Boecker H, Harding AE, Brooks DJ. Cortical control of movement in Huntington's disease. A PET activation study. Brain. 1997 Nov; 120(Pt 9):1569–1578. [PubMed: 9313640]

324. Feigin A, Leenders KL, Moeller JR, et al. Metabolic network abnormalities in early Huntington's disease: an [(18)F]FDG PET study. J Nucl Med. 2001 Nov; 42(11):1591–1595. [PubMed: 11696626]

325. Ginovart N, Lundin A, Farde L, et al. PET study of the pre- and post-synaptic dopaminergic markers for the neurodegenerative process in Huntington's disease. Brain. 1997; 120(Pt 3):503–514. [PubMed: 9126061]

326. Antonini A, Leenders KL, Eidelberg D. [11C]raclopride-PET studies of the Huntington's disease rate of progression: relevance of the trinucleotide repeat length. Ann Neurol. 1998; 43(2):253–255. [PubMed: 9485067]

327. Andrews TC, Weeks RA, Turjanski N, et al. Huntington's disease progression. PET and clinical observations. Brain. 1999; 122(Pt 12):2353–2363. [PubMed: 10581228]

328. Pavese N, Andrews TC, Brooks DJ, et al. Progressive striatal and cortical dopamine receptor dysfunction in Huntington's disease: a PET study. Brain. 2003; 126(Pt 5):1127–1135. [PubMed: 12690052]

329. Pavese N, Politis M, Tai YF, et al. Cortical dopamine dysfunction in symptomatic and premanifest Huntington's disease gene carriers. Neurobiol Dis. 2010; 37(2):356–361. [PubMed: 19853661]

330. Tai YF, Pavese N, Gerhard A, et al. Imaging microglial activation in Huntington's disease. Brain Res Bull. 2007; 72(2–3):148–151. [PubMed: 17352938]

331. Tai YF, Pavese N, Gerhard A, et al. Microglial activation in presymptomatic Huntington's disease gene carriers. Brain. 2007; 130(Pt 7):1759–1766. [PubMed: 17400599]

332. Politis M, Pavese N, Tai YF, et al. Microglial activation in regions related to cognitive function predicts disease onset in Huntington's disease: a multimodal imaging study. Hum Brain Mapp. 2011; 32(2):258–270. [PubMed: 21229614]

333. Pavese N, Gerhard A, Tai YF, et al. Microglial activation correlates with severity in Huntington disease: a clinical and PET study. Neurology. 2006; 66(11):1638–1643. [PubMed: 16769933]

NIH-PA Author Manuscript NIH-PA Author Manuscript NIH-PA Author Manuscript

334. Sbardella E, Petsas N, Tona F, et al. Assessing the correlation between grey and white matter damage with motor and cognitive impairment in multiple sclerosis patients. PLoS ONE. 2013; 8(5):e63250. [PubMed: 23696802]

335. Ceccarelli A, Bakshi R, Neema M. MRI in multiple sclerosis: a review of the current literature. Curr Opin Neurol. 2012; 25(4):402–409. [PubMed: 22691759]

336. Filippi M, Rocca MA, De Stefano N, et al. Magnetic resonance techniques in multiple sclerosis: the present and the future. Arch Neurol. 2011; 68(12):1514–1520. [PubMed: 22159052]

337. Rocca MA, Mesaros S, Pagani E, Sormani MP, Comi G, Filippi M. Thalamic damage and long-term progression of disability in multiple sclerosis. Radiology. 2010; 257(2):463–469. [PubMed: 20724544]

338. Sbardella E, Tona F, Petsas N, Pantano P. DTI Measurements in Multiple Sclerosis: Evaluation of Brain Damage and Clinical Implications. Mult Scler Int. 2013; 2013:671730. Epub 2013 Mar 31. [PubMed: 23606965]

339. Filippi M, Tortorella C, Rovaris M, et al. Changes in the normal appearing brain tissue and cognitive impairment in multiple sclerosis. J Neurol Neurosurg Psychiatry. 2000; 68(2):157–161. [PubMed: 10644780]

340. Cercignani M, Inglese M, Pagani E, Comi G, Filippi M. Mean diffusivity and fractional anisotropy histograms of patients with multiple sclerosis. AJNR Am J Neuroradiol. 2001; 22(5): 952–958. [PubMed: 11337342]

341. Bozzali M, Cercignani M, Sormani MP, Comi G, Filippi M. Quantification of brain gray matter damage in different MS phenotypes by use of diffusion tensor MR imaging. AJNR Am J Neuroradiol. 2002; 23(6):985–988. [PubMed: 12063230]

342. Vrenken H, Pouwels PJ, Geurts JJ, et al. Altered diffusion tensor in multiple sclerosis normal-appearing brain tissue: cortical diffusion changes seem related to clinical deterioration. J Magn Reson Imaging. 2006; 23(5):628–636. [PubMed: 16565955]

343. Ceccarelli A, Rocca MA, Valsasina P, et al. A multiparametric evaluation of regional brain damage in patients with primary progressive multiple sclerosis. Hum Brain Mapp. 2009; 30(9): 3009–3019. [PubMed: 19172642]

344. Preziosa P, Rocca MA, Mesaros S, et al. Intrinsic damage to the major white matter tracts in patients with different clinical phenotypes of multiple sclerosis: a voxel wise diffusion-tensor MR study. Radiology. 2011; 260(2):541–550. [PubMed: 21673227]

345. Kidd D, Barkhof F, McConnell R, Algra PR, Allen IV, Revesz T. Cortical lesions in multiple sclerosis. Brain. 1999; 122(Pt 1):17–26. [PubMed: 10050891]

346. Bobholz JA, Rao SM, Lobeck L, et al. fMRI study of episodic memory in relapsing-remitting MS: correlation with T2 lesion volume. Neurology. 2006; 67(9):1640–1645. [PubMed: 17101897]

347. Chiaravalloti N, Hillary F, Ricker J, et al. Cerebral activation patterns during working memory performance in multiple sclerosis using FMRI. J Clin Exp Neuropsychol. 2005; 27(1):33–54. [PubMed: 15814441]

348. Mainero C, Caramia F, Pozzilli C, et al. fMRI evidence of brain reorganization during attention and memory tasks in multiple sclerosis. Neuroimage. 2004; 21(3):858–867. [PubMed: 15006652]

349. Staffen W, Mair A, Zauner H, et al. Cognitive function and fMRI in patients with multiple sclerosis: evidence for compensatory cortical activation during an attention task. Brain. 2002; 125(Pt 6):1275–1282. [PubMed: 12023316]

350. Wishart HA, Saykin AJ, McDonald BC, et al. Brain activation patterns associated with working memory in relapsing-remitting MS. Neurology. 2004; 62(2):234–238. [PubMed: 14745059]

351. Genova HM, Hillary FG, Wylie G, Rypma B, Deluca J. Examination of processing speed deficits in multiple sclerosis using functional magnetic resonance imaging. J Int Neuropsychol Soc. 2009; 15(3):383–393. [PubMed: 19402924]

352. Nebel K, Wiese H, Seyfarth J, et al. Activity of attention related structures in multiple sclerosis patients. Brain Res. 2007; 1151:150–160. [PubMed: 17397807]

353. Penner IK, Rausch M, Kappos L, Opwis K, Radü EW. Analysis of impairment related functional architecture in MS patients during performance of different attention tasks. J Neurol. 2003 Jul; 250(4):461–472. [PubMed: 12700913]

NIH-PA Author Manuscript NIH-PA Author Manuscript NIH-PA Author Manuscript

354. Filippi M, Agosta F, Spinelli EG, Rocca MA. Imaging resting state brain function in multiple sclerosis. J Neurol. 2013 Jul; 260(7):1709–1713. [PubMed: 23052604]
355. Rocca MA, Absinta M, Filippi M. The role of advanced magnetic resonance imaging techniques in primary progressive MS. J Neurol. 2012; 259(4):611–621. [PubMed: 21814822]
356. Rocca MA, Valsasina P, Martinelli V, et al. Large-scale neuronal network dysfunction in relapsing-remitting multiple sclerosis. Neurology. 2012; 79(14):1449–1457. [PubMed: 22955126]
357. Faivre A, Rico A, Zaaraoui W, et al. Assessing brain connectivity at rest is clinically relevant in early multiple sclerosis. Mult Scler. 2012; 18(9):1251–1258. [PubMed: 22307385]
358. Hawellek DJ, Hipp JF, Lewis CM, Corbetta M, Engel AK. Increased functional connectivity indicates the severity of cognitive impairment in multiple sclerosis. Proc Natl Acad Sci U S A. 2011; 108(47):19066–19071. [PubMed: 22065778]
359. Loitfelder M, Filippi M, Rocca M, et al. Abnormalities of resting state functional connectivity are related to sustained attention deficits in MS. PLoS ONE. 2012; 7(8):e42862. [PubMed: 22912754]
360. Bonavita S, Gallo A, Sacco R, et al. Distributed changes in default-mode resting-state connectivity in multiple sclerosis. Mult Scler. 2011; 17(4):411–422. [PubMed: 21239414]
361. Sun X, Tanaka M, Kondo S, Okamoto K, Hirai S. Clinical significance of reduced cerebral metabolism in multiple sclerosis: a combined PET and MRI study. Ann Nucl Med. 1998; 12(2): 89–94. [PubMed: 9637279]
362. Paulesu E, Perani D, Fazio F, et al. Functional basis of memory impairment in multiple sclerosis: a[18F]FDG PET study. Neuroimage. 1996; 4(2):87–96. [PubMed: 9345500]
363. Pozzilli C, Fieschi C, Perani D, et al. Relationship between corpus callosum atrophy and cerebral metabolic asymmetries in multiple sclerosis. J Neurol Sci. 1992; 112(1–2):51–57. [PubMed: 1469440]
364. Blinkenberg M, Jensen CV, Holm S, Paulson OB, Sørensen PS. A longitudinal study of cerebral glucose metabolism, MRI, and disability in patients with MS. Neurology. 1999; 53(1):149–153. [PubMed: 10408551]
365. Banati RB, Newcombe J, Gunn RN, et al. The peripheral benzodiazepine binding site in the brain in multiple sclerosis: quantitative in vivo imaging of microglia as a measure of disease activity. Brain. 2000 May; 123(Pt 11):2321–2337. [PubMed: 11050032]
366. Debruyne JC, Versijpt J, Van Laere KJ, et al. PET visualization of microglia in multiple sclerosis patients using [11C]PK11195. Eur J Neurol. 2003 May; 10(3):257–264. [PubMed: 12752399]
367. Versijpt J, Debruyne JC, Van Laere KJ, et al. Microglial imaging with positron emission tomography and atrophy measurements with magnetic resonance imaging in multiple sclerosis: a correlative study. Mult Scler. 2005; 11(2):127–134. [PubMed: 15794383]
368. Politis M, Giannetti P, Su P, et al. Increased PK11195 PET binding in the cortex of patients with MS correlates with disability. Neurology. 2012; 79(6):523–530. [PubMed: 22764258]
369. Chang L, Ernst T, Leonido-Yee M, Walot I, Singer E. Cerebral metabolite abnormalities correlate with clinical severity of HIV-1 cognitive motor complex. Neurology. 1999; 52(1):100–108. [PubMed: 9921855]
370. Küper M, Rabe K, Esser S, et al. Structural gray and white matter changes in patients with HIV. J Neurol. 2011; 258(6):1066–1075. [PubMed: 21207051]
371. Ragin AB, Du H, Ochs R, et al. Structural brain alterations can be detected early in HIV infection. Neurology. 2012; 79(24):2328–2334. [PubMed: 23197750]
372. Thompson PM, Dutton RA, Hayashi KM, et al. Thinning of the cerebral cortex visualized in HIV/AIDS reflects CD4+ T lymphocyte decline. Proc Natl Acad Sci U S A. 2005; 102(43):15647–15652. [PubMed: 16227428]
373. Chang L, Ernst T, Tornatore C, et al. Metabolite abnormalities in progressive multifocal leukoencephalopathy by proton magnetic resonance spectroscopy. Neurology. 1997; 48(4):836–845. [PubMed: 9109865]
374. Mark AS, Atlas SW. Progressive multifocal leukoencephalopathy in patients with AIDS: appearance on MR images. Radiology. 1989; 173(2):517–520. [PubMed: 2798883]

NIH-PA Author Manuscript NIH-PA Author Manuscript NIH-PA Author Manuscript

375. Paul RH, Ernst T, Brickman AM, et al. ACTG 301 team ; ACTG 700 team ; HIV MRS Consortium. Relative sensitivity of magnetic resonance spectroscopy and quantitative magnetic resonance imaging to cognitive function among nondemented individuals infected with HIV. J Int Neuropsychol Soc. 2008; 14(5):725–733. [PubMed: 18764968]
376. Ragin AB, Storey P, Cohen BA, Epstein LG, Edelman RR. Whole brain diffusion tensor imaging in HIV-associated cognitive impairment. AJNR Am J Neuroradiol. 2004; 25(2):195–200. [PubMed: 14970017]
377. Tate DF, Conley J, Paul RH, et al. Quantitative diffusion tensor imaging tractography metrics are associated with cognitive performance among HIV-infected patients. Brain Imaging Behav. 2010; 4(1):68–79. [PubMed: 20503115]
378. Leite SC, Corrêa DG, Doring TM, et al. Diffusion tensor MRI evaluation of the corona radiata, cingulate gyri, and corpus callosum in HIV patients. J Magn Reson Imaging. 2013
379. Garvey LJ, Pavese N, Ramlackhansingh A, et al. Acute HCV/HIV coinfection is associated with cognitive dysfunction and cerebral metabolite disturbance, but not increased microglial cell activation. PLoS ONE. 2012; 7(7):e38980. [PubMed: 22808022]
380. Tucker KA, Robertson KR, Lin W, et al. Neuroimaging in human immunodeficiency virus infection. J Neuroimmunol. 2004; 157(1–2):153–162. [PubMed: 15579293]
381. Chang L, Speck O, Miller EN, et al. Neural correlates of attention and working memory deficits in HIV patients. Neurology. 2001; 57(6):1001–1007. [PubMed: 11571324]
382. Ernst T, Chang L, Jovicich J, Ames N, Arnold S. Abnormal brain activation on functional MRI in cognitively asymptomatic HIV patients. Neurology. 2002; 59(9):1343–1349. [PubMed: 12427881]
383. Maki PM, Cohen MH, Weber K, et al. Impairments in memory and hippocampal function in HIV-positive vs HIV-negative women: a preliminary study. Neurology. 2009; 72(19):1661–1668. [PubMed: 19433739]
384. Melrose RJ, Tinaz S, Castelo JM, Courtney MG, Stern CE. Compromised fronto-striatal functioning in HIV: an fMRI investigation of semantic event sequencing. Behav Brain Res. 2008; 188(2):337–347. [PubMed: 18242723]
385. Thomas JB, Brier MR, Snyder AZ, Vaida FF, Ances BM. Pathways to neurodegeneration: effects of HIV and aging on resting-state functional connectivity. Neurology. 2013; 80(13):1186–1193. [PubMed: 23446675]
386. Chang L, Ernst T, Leonido-Yee M, Speck O. Perfusion MRI detects rCBF abnormalities in early stages of HIV-cognitive motor complex. Neurology. 2000; 54(2):389–396. [PubMed: 10668700]
387. Maini CL, Pigorini F, Pau FM, et al. Cortical cerebral blood flow in HIV-1-related dementia complex. Nucl Med Commun. 1990; 11(9):639–648. [PubMed: 2234697]
388. Tracey I, Hamberg LM, Guimaraes AR, et al. Increased cerebral blood volume in HIV-positive patients detected by functional MRI. Neurology. 1998; 50(6):1821–1826. [PubMed: 9633734]
389. Rottenberg DA, Sidtis JJ, Strother SC, et al. Abnormal cerebral glucose metabolism in HIV-1 seropositive subjects with and without dementia. J Nucl Med. 1996 Jul; 37(7):1133–1141. [PubMed: 8965184]
390. Rottenberg DA, Moeller JR, Strother SC, et al. The metabolic pathology of the AIDS dementia complex. Ann Neurol. 1987; 22(6):700–706. [PubMed: 3501695]
391. Davison JM, Subramaniam RM, Surasi DS, Cooley T, Mercier G, Peller PJ. FDG PET/CT in patients with HIV. AJR Am J Roentgenol. 2011; 197(2):284–294. [PubMed: 21785073]
392. Pascal S, Resnick L, Barker WW, et al. Metabolic asymmetries in asymptomatic HIV-1 seropositive subjects: relationship to disease onset and MRI findings. J Nucl Med. 1991 Sep; 32(9):1725–1729. [PubMed: 1880574]
393. Wang GJ, Chang L, Volkow ND, et al. Decreased brain dopaminergic transporters in HIV-associated dementia patients. Brain. 2004; 127(Pt 11):2452–2458. [PubMed: 15319273]
394. Ioannides P, Karacostas D. Neuroimaging in human prion disease: Searching in the mist. World J Radiol. 2009 Dec 31; 1(1):45–49. [PubMed: 21160720]
395. Macfarlane RG, Wroe SJ, Collinge J, Yousry TA, Jäger HR. Neuroimaging findings in human prion disease. J Neurol Neurosurg Psychiatry. 2007; 78(7):664–670. [PubMed: 17135459]

NIH-PA Author Manuscript NIH-PA Author Manuscript NIH-PA Author Manuscript

396. Puoti G, Bizzi A, Forloni G, Safar JG, Tagliavini F, Gambetti P. Sporadic human prion diseases: molecular insights and diagnosis. Lancet Neurol. 2012; 11(7):618–628. [PubMed: 22710755]
397. Tschampa HJ, Kallenberg K, Urbach H, et al. MRI in the diagnosis of sporadic Creutzfeldt-Jakob disease: a study on inter-observer agreement. Brain. 2005; 128(Pt 9):2026–2033. [PubMed: 15958503]
398. Schröter A, Zerr I, Henkel K, Tschampa HJ, Finkenstaedt M, Poser S. Magnetic resonance imaging in the clinical diagnosis of Creutzfeldt-Jakob disease. Arch Neurol. 2000; 57(12):1751–1757. [PubMed: 11115241]
399. Ukisu R, Kushihashi T, Kitanosono T, et al. Serial diffusion-weighted MRI of Creutzfeldt-Jakob disease. AJR Am J Roentgenol. 2005; 184(2):560–566. [PubMed: 15671380]
400. Shiga Y, Miyazawa K, Sato S, et al. Diffusion-weighted MRI abnormalities as an early diagnostic marker for Creutzfeldt-Jakob disease. Neurology. 2004; 63(3):443–449. [PubMed: 15304574]
401. Meissner B, Körtner K, Bartl M, et al. Sporadic Creutzfeldt-Jakob disease: magnetic resonance imaging and clinical findings. Neurology. 2004; 63(3):450–456. [PubMed: 15314808]
402. Kim JH, Choi BS, Jung C, Chang Y, Kim S. Diffusion-weighted imaging and magnetic resonance spectroscopy of sporadic Creutzfeldt-Jakob disease: correlation with clinical course. Neuroradiology. 2011; 53(12):939–945. [PubMed: 21221559]
403. Collie DA, Summers DM, Sellar RJ, et al. Diagnosing variant Creutzfeldt-Jakob disease with the pulvinar sign: MR imaging findings in 86 neuropathologically confirmed cases. AJNR Am J Neuroradiol. 2003; 24(8):1560–1569. [PubMed: 13679271]
404. Zeidler M, Sellar RJ, Collie DA, et al. The pulvinar sign on magnetic resonance imaging in variant Creutzfeldt-Jakob disease. Lancet. 2000; 355(9213):1412–1418. [PubMed: 10791525]
405. Will RG, Zeidler M, Stewart GE, et al. Diagnosis of new variant Creutzfeldt-Jakob disease. Ann Neurol. 2000; 47(5):575–582. [PubMed: 10805327]
406. Pandya HG, Coley SC, Wilkinson ID, Griffiths PD. Magnetic resonance spectroscopic abnormalities in sporadic and variant Creutzfeldt-Jakob disease. Clin Radiol. 2003; 58(2):148–153. [PubMed: 12623045]
407. Galanaud D, Haik S, Linguraru MG, et al. Combined diffusion imaging and MR spectroscopy in the diagnosis of human prion diseases. AJNR Am J Neuroradiol. 2010; 31(7):1311–1318. [PubMed: 20430851]
408. de Silva R, Patterson J, Hadley D, Russell A, Turner M, Zeidler M. Single photon emission computed tomography in the identification of new variant Creutzfeldt-Jakob disease: case reports. BMJ. 1998; 316(7131):593–594. [PubMed: 9518911]
409. Meissner B, Kallenberg K, Sanchez-Juan P, et al. MRI and clinical syndrome in dura mater-related Creutzfeldt-Jakob disease. J Neurol. 2009; 256(3):355–363. [PubMed: 19159063]
410. García Santos JM, López Corbalán JA, Martínez-Lage JF, Sicilia Guillén JCT. CT and MRI in iatrogenic and sporadic Creutzfeldt-Jakob disease: as far as imaging perceives. Neuroradiology. 1996; 38(3):226–231. [PubMed: 8741192]
411. du Plessis DG. Prion protein disease and neuropathology of prion disease. Neuroimaging Clin N Am. 2008; 18(1):163–182. ix, ix. [PubMed: 18319161]
412. Fulbright RK, Hoffmann C, Lee H, Pozamantir A, Chapman J, Prohovnik I. MR imaging of familial Creutzfeldt-Jakob disease: a blinded and controlled study. AJNR Am J Neuroradiol. 2008; 29(9):1638–1643. [PubMed: 18635614]
413. Wimberger D, Uranitsch K, Schindler E, Kramer J. Gerstmann-Sträussler-Scheinker syndrome: MR findings. J Comput Assist Tomogr. 1993; 17(2):326–327. [PubMed: 8454765]
414. Arata H, Takashima H, Hirano R, et al. Early clinical signs and imaging findings in Gerstmann-Sträussler-Scheinker syndrome (Pro102Leu). Neurology. 2006; 66(11):1672–1678. [PubMed: 16769939]
415. Aralasmak A, Crain BJ, Zou WQ, Yousem DM. A prion disease—possible Gerstmann-Straussler-Scheinker disease: a case report. J Comput Assist Tomogr. 2006; 30(1):135–139. [PubMed: 16365589]
416. Waldman AD, Cordery RJ, MacManus DG, Godbolt A, Collinge J, Rossor MN. Regional brain metabolite abnormalities in inherited prion disease and asymptomatic gene carriers demonstrated

NIH-PA Author Manuscript NIH-PA Author Manuscript NIH-PA Author Manuscript

in vivo by quantitative proton magnetic resonance spectroscopy. Neuroradiology. 2006; 48(6): 428–433. [PubMed: 16598479]

417. Konaka K, Kaido M, Okuda Y, et al. Proton magnetic resonance spectroscopy of a patient with Gerstmann-Straussler-Scheinker disease. Neuroradiology. 2000; 42(9):662–665. [PubMed: 11071439]

418. Park MJ, Jo HY, Cheon SM, Choi SS, Kim YS, Kim JW. A case of gerstmann-sträussler-scheinker disease. J Clin Neurol. 2010; 6(1):46–50. [PubMed: 20386644]

419. Zerr I, Giese A, Windl O, et al. Phenotypic variability in fatal familial insomnia (D178N-129M) genotype. Neurology. 1998; 51(5):1398–1405. [PubMed: 9818868]

420. Almer G, Hainfellner JA, Brücke T, et al. Fatal familial insomnia: a new Austrian family. Brain. 1999; 122(Pt 1):5–16. [PubMed: 10050890]

421. Lodi R, Parchi P, Tonon C, et al. Magnetic resonance diagnostic markers in clinically sporadic prion disease: a combined brain magnetic resonance imaging and spectroscopy study. Brain. 2009; 132(Pt 10):2669–2679. [PubMed: 19755520]

422. Matoba M, Tonami H, Miyaji H, Yokota H, Yamamoto I. Creutzfeldt-Jakob disease: serial changes on diffusion-weighted MRI. J Comput Assist Tomogr. 2001; 25(2):274–277. [PubMed: 11242228]

423. Montagna P, Gambetti P, Cortelli P, Lugaresi E. Familial and sporadic fatal insomnia. Lancet Neurol. 2003; 2(3):167–176. [PubMed: 12849238]

424. Jagust WJ, Bandy D, Chen K, et al. Alzheimer's Disease Neuroimaging Initiative. The Alzheimer's Disease Neuroimaging Initiative positron emission tomography core. Alzheimers Dement. 2010; 6(3):221–229. [PubMed: 20451870]

425. Swaminathan S, Shen L, Risacher SL, et al. Alzheimer's Disease Neuroimaging Initiative. Amyloid pathway-based candidate gene analysis of [(11)C]PiB-PET in the Alzheimer's Disease Neuroimaging Initiative (ADNI) cohort. Brain Imaging Behav. 2012 Mar; 6(1):1–15. [PubMed: 21901424]

426. Risacher SL, Saykin AJ. Neuroimaging and other biomarkers for Alzheimer's disease: the changing landscape of early detection. Annu Rev Clin Psychol. 2013; 9:621–648. [PubMed: 23297785]

427. Kassubek J, Juengling FD, Kioschies T, et al. Topography of cerebral atrophy in early Huntington's disease: a voxel based morphometric MRI study. J Neurol Neurosurg Psychiatry. 2004; 75(2):213–220. [PubMed: 14742591]

NIH-PA Author Manuscript

NIH-PA Author Manuscript

NIH-PA Author Manuscript

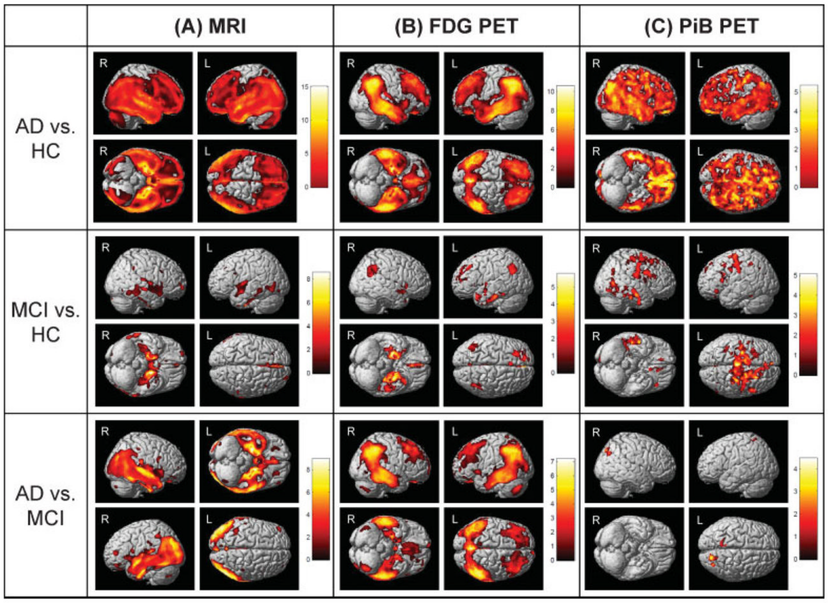

Fig. 1.
Differences in atrophy, glucose metabolism, and amyloid deposition between patients with Alzheimer's disease (AD), patients with mild cognitive impairment (MCI), and healthy older adults (HC). The pattern of differences between AD, MCI, and HC is demonstrated in (**A**) brain atrophy (measured using T1-weighted structural magnetic resonance imaging [(MRI]), (**B**) glucose metabolism (measured using [18F] fluorodeoxyglucose positron emission tomography [FDG PET]), and (**C**) amyloid accumulation (measured using [11C]Pittsburgh compound B positron emission tomography [PiB PET]). Relative to HC, patients with AD show significantly reduced brain gray matter density throughout cortical and subcortical regions (A; AD versus HC), reduced glucose metabolism in regions of the medial and lateral parietal lobe, medial and lateral temporal lobes, and medial and lateral frontal lobes (B; AD vs. HC), and greater amyloid accumulation throughout the cerebral cortex (C; AD vs. HC). Patients with MCI also show focal changes relative to HC, including reduced gray matter density in the medial and lateral temporal lobes (A; MCI vs. HC), reduced glucose metabolism in the medial and lateral temporal lobes, medial and lateral parietal lobes, and frontal lobe (B; MCI vs. HC), and greater amyloid deposition in the frontal, parietal, and temporal cortices (C;MCI vs. HC). The comparisons of these measures between patients with AD to patients with MCI also show interesting patterns of relating to disease severity. Patients with AD show significantly more gray matter atrophy in regions of the medial and lateral temporal lobes and parietal lobes (A; AD vs. MCI) and reduced glucose metabolism in the medial and lateral temporal lobes, medial and lateral parietal lobes, and frontal lobe (B; AD vs. MCI) relative to MCI patients. However, only minor differences in amyloid are observed between AD and MCI patients (C; AD vs. MCI), suggesting the majority of amyloid accumulation occurs before a participant has reached a clinical diagnosis of MCI. This figure was generated using data from the Alzheimer's Disease Neuroimaging Initiative cohort and utilizing traditional methods that have been previously described.[39,424,425] Panel (A) is displayed at a voxel-wise threshold of $p < 0.01$ (family-wise error correction for multiple comparisons) and minimum cluster size (k) = 50 voxels and includes 189 AD, 396 MCI, and 225 HC participants; panel (B) is displayed at a voxel-wise threshold of $p < 0.001$ (uncorrected for multiple comparisons) and k = 50 voxels and includes 97 AD, 203 MCI, and 102 HC participants; panel (C) is displayed at a voxel-wise threshold of $p < 0.01$ (uncorrected for multiple comparisons) and k = 50 voxels and includes 25 AD, 56 MCI, and 22 HC participants. (Reproduced from Risacher et al[426])

NIH-PA Author Manuscript
NIH-PA Author Manuscript
NIH-PA Author Manuscript

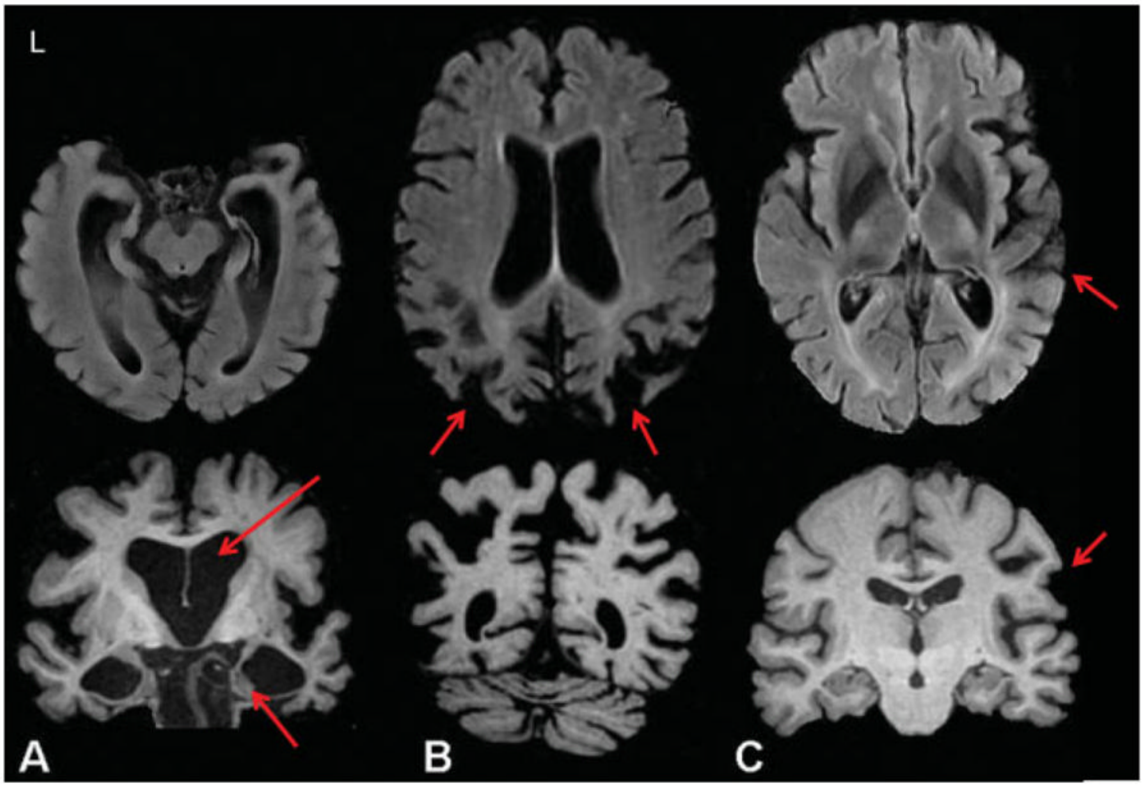

Fig. 2.

Differences in atrophy between traditional Alzheimer's disease and atypical Alzheimer's disease. (**A**) Significant but generalized cortical atrophy, as well as dramatic volumetric reductions in the medial temporal lobe (MTL) are observed in traditional late-onset Alzheimer's disease (AD) (*arrows*). However, different patterns of atrophy are observed in (**B**) posterior cortical atrophy (PCA) and (**C**) logopenic aphasia. (**B**) Patients with PCA show significantly more atrophy in posterior cortical regions (parietal lobe, occipital lobe) than seen in other forms of AD (*arrows*). (**C**) Patients with logopenic aphasia show relatively localized atrophy in the posterior temporal lobe and temporoparietal regions with greater atrophy observed in the left hemisphere than in the right (*arrows*). (Adapted from McGinnis et al[11])

NIH-PA Author Manuscript
NIH-PA Author Manuscript
NIH-PA Author Manuscript

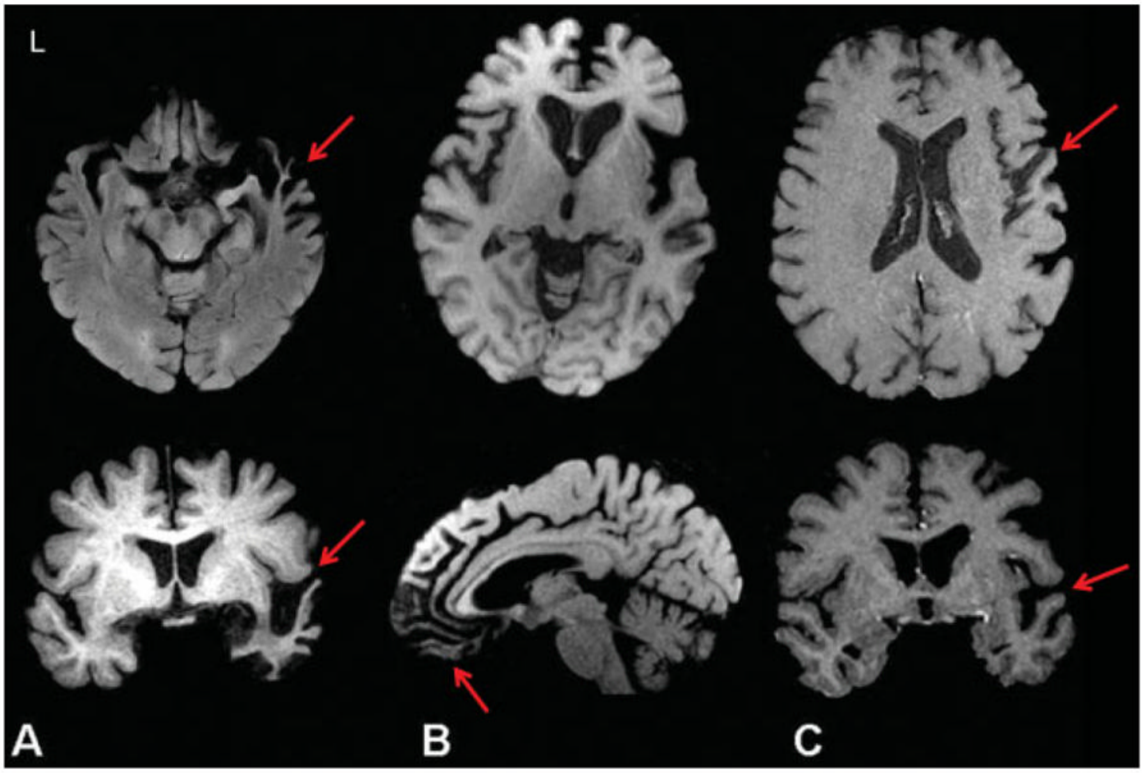

Fig. 3.
Atrophy in frontotemporal dementia (FTD) subtypes. (**A**) Significant left anterior temporal lobe atrophy is observed in the semantic dementia variant of FTD (*arrows*), while bilateral frontal and temporal lobe atrophy is seen in the (**B**) behavioral variant of FTD (*arrows*). (**C**) Patients with progressive nonfluent aphasia show atrophy in the left inferior frontal, insula, and anterior temporal lobe regions (*left* > *right; arrows*). (Adapted from McGinnis et al[11])

NIH-PA Author Manuscript
NIH-PA Author Manuscript
NIH-PA Author Manuscript

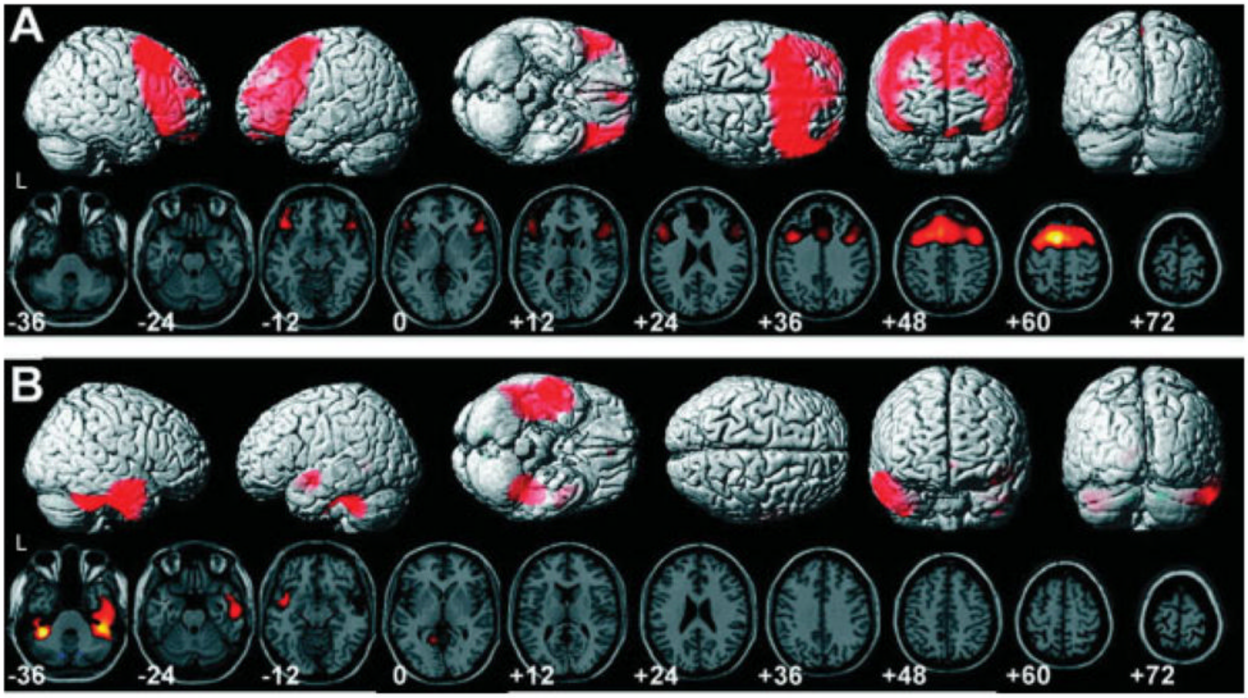

Fig. 4.
Hypometabolism in patients with frontotemporal dementia with motor neuron disease (FTD-MND) relative to FTD without motor neuron disease and healthy older adults (HC). (**A**) Significant bilateral frontal lobe hypometabolism, with relative sparing of the temporal lobe, was observed in patients with FTD with motor neuron disease (FTD-MND) relative to HC. (**B**) However, relative to FTD patients without motor neuron disease, FTD-MND patients show reduced bilateral temporal lobe metabolism. (Adapted from Jeong et al[224])

NIH-PA Author Manuscript
NIH-PA Author Manuscript
NIH-PA Author Manuscript

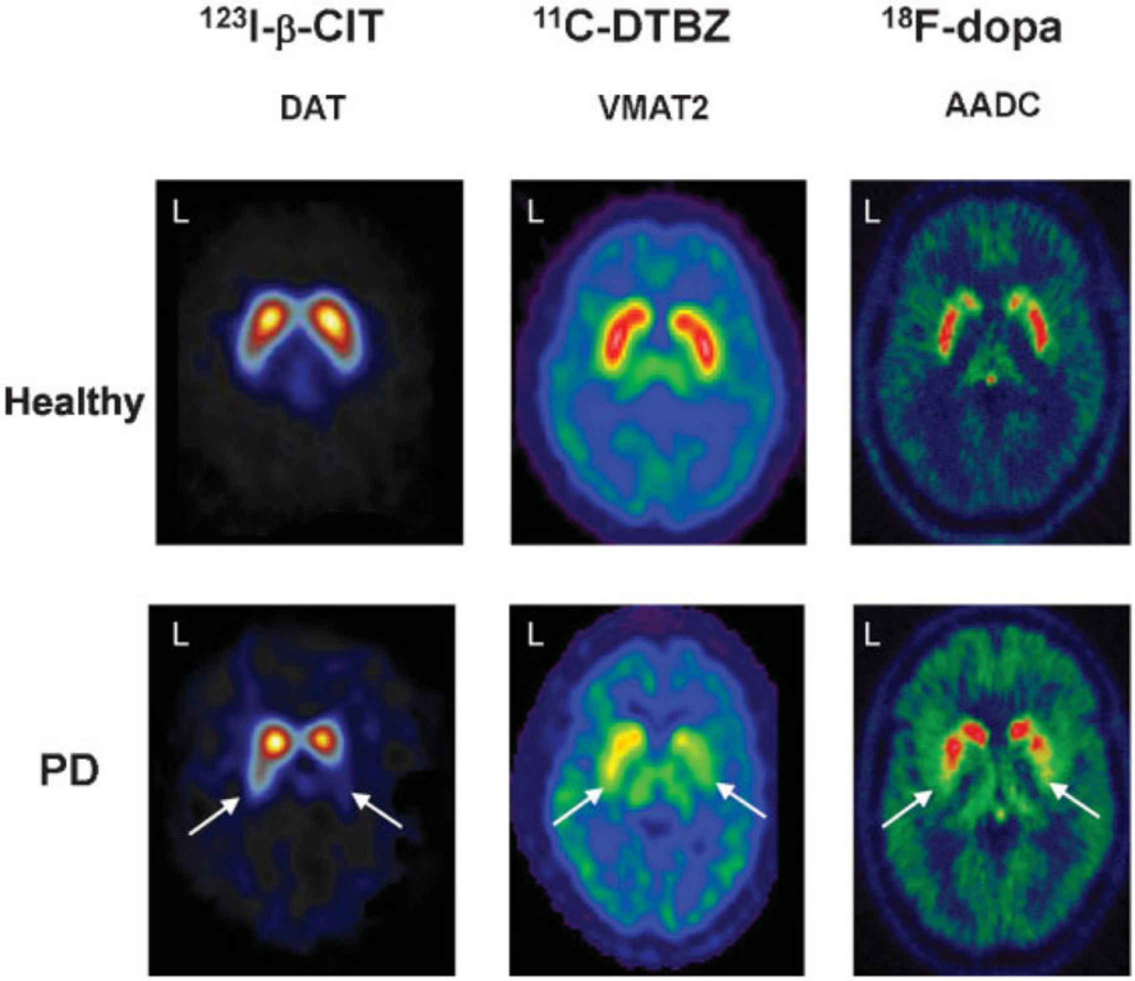

Fig. 5.

Dopaminergic deficits in Parkinson's disease (PD) relative to healthy adults. Reduced dopaminergic neurotransmission is observed in patients with PD relative to healthy adults ("healthy"), particularly in the posterior putamen (*arrows*). ^{123}I-β-CIT labels the dopamine transporter (DAT), which is located presynaptically on dopamine-releasing terminals. ^{11}C-DTBZ labels the vesicular monoamine transporter (VMAT) and ^{18}F-dopa labels amino acid decarboxylase (AADC). Both of these molecules are found in neuron terminals releasing dopamine. Overall, these three positron emission tomography (PET) tracers provide sensitive measures of the density of neuron terminals releasing dopamine in the striatum. (Adapted from Brooks et al[283])

NIH-PA Author Manuscript
NIH-PA Author Manuscript
NIH-PA Author Manuscript

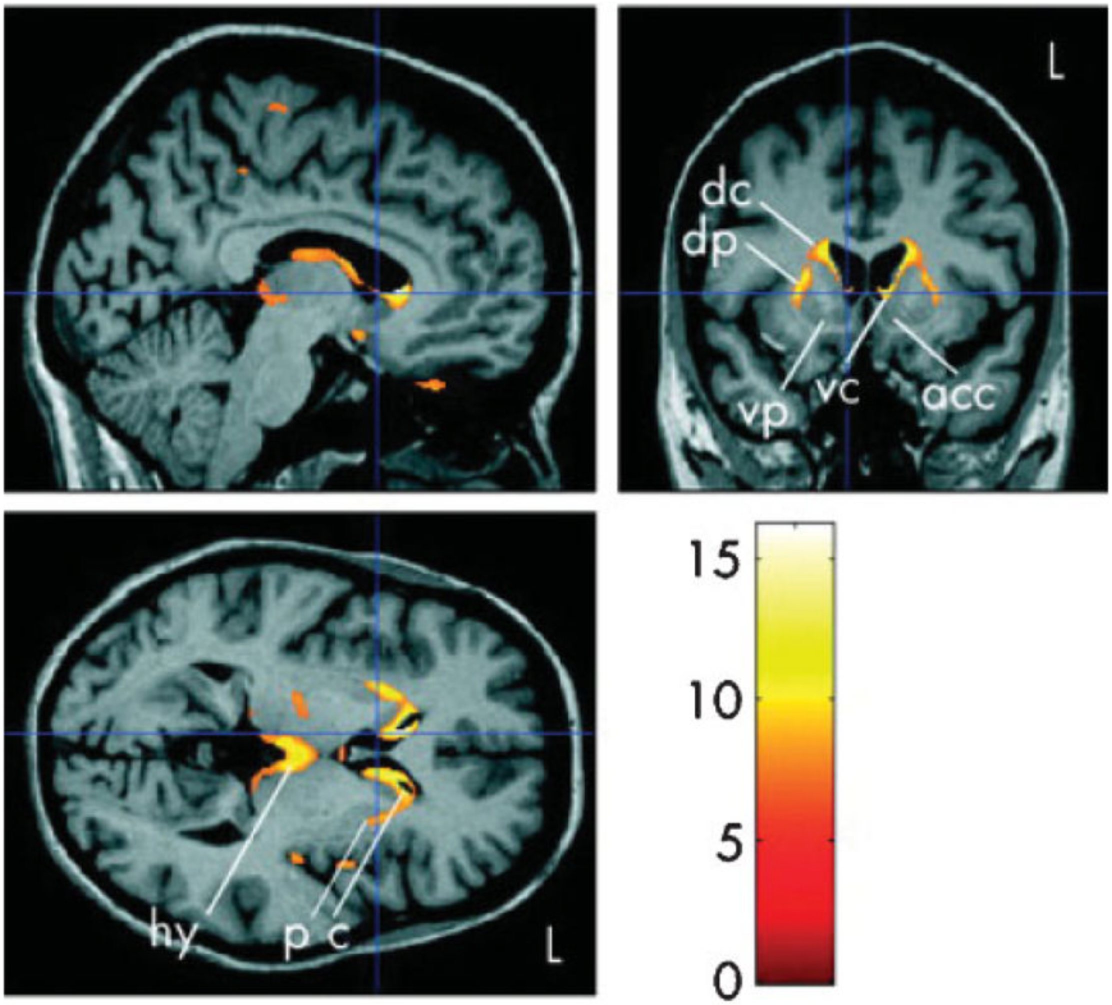

Fig. 6.
Atrophic changes in early Huntington's disease (HD) relative to healthy adults. Significant atrophy is seen in the dorsal caudate (dc), ventral caudate (vc), dorsal putamen (dp), and hypothalamus (hy) of patients with HD relative to controls. Less atrophy is observed in the ventral putamen (vp) and nucleus accumbens (acc). (Adapted from Kassubek et al[427])

NIH-PA Author Manuscript
NIH-PA Author Manuscript
NIH-PA Author Manuscript

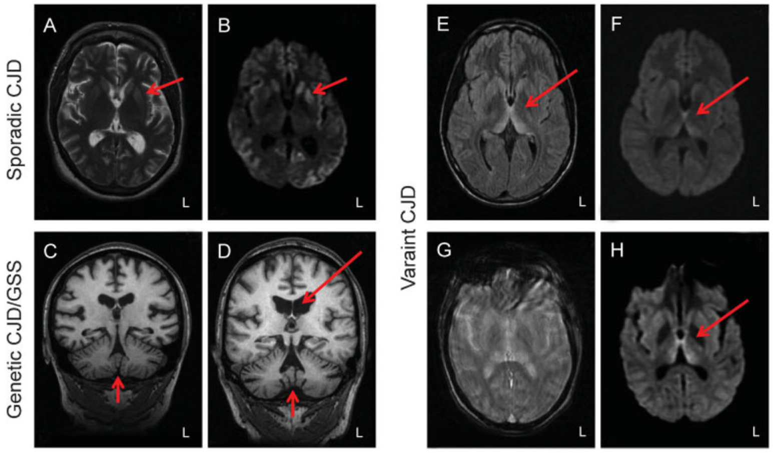

Fig. 7.

Structural imaging changes in prion protein related diseases. (**A**) An axial T2-weighted magnetic resonance image (MRI) of a patient with sporadic Creutzfeldt-Jakob disease (CJD) shows a subtle increase in signal intensity in the left anterior putamen (arrow). (**B**) A diffusion weighted image (DWI) also shows a more apparent hyperintense signal in the bilateral caudate and left putamen. Patients with (**C**) genetic CJD and (**D**) Gerstmann-Straussler-Scheinker disease show notable cortical and cerebellar atrophy. (**E**) An axial fluid attenuated inversion recovery (FLAIR) image in a patient with variant CJD shows hyperintense signal in the dorsomedial thalamus and pulvinar bilaterally, creating a hockey stick pattern. (**F**) A similar pattern, although slightly less apparent, is seen on the DWI scan. (**G**) An axial T2-weighted MRI of a patient with variant CJD is of limited diagnostic value due to patient movement, while (H) the DWI scan shows prominent hyperintensity in the bilateral dorsomedial thalamus and pulvinar. (Adapted from Macfarlane et al[395])

NIH-PA Author Manuscript
NIH-PA Author Manuscript
NIH-PA Author Manuscript

Table 1

Brief Summary of Neuroimaging Findings in Selected Neurodegenerative Diseases and Dementias

**Disease**	**Clinical symptoms**	**Atrophy pattern**	**Functional activation/connectivity changes**	**Molecular changes**	**Other imaging**
Sporadic Alzheimer's disease (AD)	Memory decline; Impairment in other cognitive domains; Dementia/Functional decline	MCI/mild AD: Atrophy of MTL (Hipp, EC)<br>More advanced AD: Atrophy of frontal, temporal, & parietal lobes	↑ or ↓ activation of task-related regions during cognitive tasks<br>↓ connectivity of brain networks (e.g., DMN)	[18F]FDG: ↓ metabolism in temporoparietal regions<br>Amyloid PET positive ↓ ACh, GABA, 5-HT, & DA neurotransmission ↑ activated microglia?	MRS: ↓ NAA, ↑ mIns<br>DTI: Widespread atrophy of frontal, temporal, & parietal white matter tracts<br>ASL/SPECT: ↓ perfusion of temporoparietal regions
Familial AD	Decline in memory and other cognitive domains; Dementia/ Functional decline	Atrophy in the MTL (Hipp, EC), lateral temporal lobe, and parietal cortex	↑ or ↓ activation of task-related regions during cognitive tasks<br>↑ and ↓ connectivity of DMN network	[18F]FDG: ↓ metabolism in temporoparietal regions<br>Amyloid PET positive – sporadic AD-like pattern plus striatal binding	DTI: Reduced integrity of fornix, CC, cingulum, and subcortical white matter tracts<br>SPECT: ↓ perfusion of temporoparietal regions
Posterior cortical atrophy	Visual and visuospatial deficits	Atrophy of posterior brain regions (posterior temporal, parietal, occipital lobes)	n/a	Amyloid PET positive	DTI: ↓ white matter integrity in ventral visual processing stream<br>ASL/SPECT: ↓ perfusion in occipitoparietal lobe, ↑ in frontal lobe, anterior cingulate, mesiotemporal lobe
Logopenic aphasia	Impaired language (word retrieval, sentence repetition)	Atrophy of posterior temporal lobe, temporoparietal lobe, medial parietal lobe, MTL (left > right)	n/a	[18F]FDG: ↓ metabolism left temporoparietal lobe<br>Amyloid PET positive	DTI: ↓ white matter integrity in left temporoparietal region<br>ASL/SPECT: ↓ perfusion in left temporoparietal lobe
Cerebral amyloid angiopathy	Cognitive/functional decline in any domain	Generalized cerebral atrophy with cerebral microhemorrhages, microbleeds, and other ischemic-related changes	Altered vascular reactivity during visual stimulation	Amyloid PET positive	SPECT: ↓ perfusion in parietal, temporal, and frontal lobes
Vascular dementia	Significant cognitive decline in any domain coupled with vascular event	Atrophy of the cerebral cortex and MTL; ischemic-related changes (i.e., white matter lesions)	↓ Activation and altered blood flow-metabolic coupling during cognitive and motor tasks<br>↓ Connectivity in the posterior cingulate	[18F]FDG: ↓ metabolism in frontal and parietal lobes<br>Amyloid PET negative	DTI: ↓ white matter integrity in widespread cortical regions (even in

NIH-PA Author Manuscript NIH-PA Author Manuscript NIH-PA Author Manuscript

Disease	Clinical symptoms	Atrophy pattern	Functional activation/connectivity changes	Molecular changes	Other imaging
					normal appearing white matter)<br>ASL/SPECT: ↓ perfusion in frontal and parietal lobes
Behavioral variant FTD	Personality and behavior changes (disinhibition, apathy, loss of empathy, etc.)	Atrophy in the frontal lobe, anterior cingulate, anterior insula, thalamus	↓ Activation in frontal and parietal lobe during working memory; altered activation during emotional tasks<br>↓ Connectivity in basal ganglia, frontal lobe (i.e., salience network)	[18F]FDG: ↓ Metabolism frontal lobe<br>Amyloid PET negative	DTI: ↓ white matter integrity in frontal and temporal lobes<br>ASL/SPECT: ↓ perfusion in frontal and parietal lobes
Semantic dementia	Impaired language/ fluency (fluent aphasia, anomia, etc.)	Asymmetrical atrophy of the anterior, medial, & inferior temporal lobes (left > right)	Altered activation during sound, memory, & language tasks<br>↓ connectivity in frontotemporal and frontolimbic networks; ↑ connectivity in PFC	[18F]FDG: ↓ Metabolism in the left anterior temporal lobe<br>Amyloid PET negative	DTI: ↓ white matter integrity in bilateral (left > right) temporal lobes<br>ASL/SPECT: ↓ perfusion in the left anterior temporal lobe
Progressive nonfluent aphasia (PNFA)	Impaired language (difficulty in speech production, agrammatism, apraxia of speech)	Mild PNFA: Atrophy in left inferior frontal, insula, premotor cortex, temporal lobe<br>Advanced PNFA: Atrophy spreads to include temporal and parietal lobes, caudate, and thalamus	↓ Activation during sentence reading and comprehension	[18F]FDG: ↓ metabolism in the frontal lobe, insula, motor areas<br>Amyloid PET negative (except those with Pick's disease)<br>Reduced striatal D A	DTI: ↓ white matter integrity in frontal lobe, insula, & superior motor pathway<br>ASL/SPECT: ↓perfusion in the left anterior temporal lobe
FTD with motor neuron disease (FTD-MND/FTD-ALS)	Behavioral/language impairments with motor dysfunction	Atrophy of frontal and temporal lobes, anterior cingulate, occipital lobe, precentral gyrus	↓ Activation in frontal lobe, anterior cingulate, temporal lobe, occipitotemporal lobe during verbal fluency and emotional task<br>Altered connectivity in sensorimotor, motor, & frontoparietal networks	[18F]FDG: ↓ metabolism in the frontal and temporal lobes, basal ganglia, thalamus<br>Reduced frontal lobe 5-HT binding<br>Reduced GABA-A receptors in cerebral cortex and insula	DTI: ↓ white matter integrity in CC, CST, cingulum, frontal lobe white matter tracts<br>ASL/SPECT: ↓ perfusion frontal and temporal lobes
Amyotrophic lateral sclerosis	Motor dysfunction	Atrophy of motor and extramotor regions (i.e., precentral gyrus)	↑ or ↓ activation of task-related regions during motor & emotional tasks<br>Altered connectivity in sensorimotor & motor networks, DMN	[18F]FDG: ↓ cortical metabolism<br>Reduced D A and GABA cells in the basal ganglia & substantia nigra<br>↑ activated microglia in CST and extra-motor regions	MRS: ↓ NAA, ↑ mIns, choline, Glmn, Glmt<br>DTI: ↓ white matter integrity in CC, CST, PLIC, cingulum, frontal & temporal white matter tracts<br>ASL/SPECT: ↓ cortical perfusion

NIH-PA Author Manuscript NIH-PA Author Manuscript NIH-PA Author Manuscript

Disease	Clinical symptoms	Atrophy pattern	Functional activation/connectivity changes	Molecular changes	Other imaging
Parkinson's disease dementia (PDD)/ dementia with Lewy bodies (DLB)	Motor dysfunction with cognitive impairment (spontaneous motor parkinsonism, visual hallucinations, etc.)	Widespread cortical and subcortical atrophy	↑ or ↓ activation during visual tasks<br>Altered global and local cortico-connectivity	[18F]FDG: ↓ metabolism in the basal ganglia, cerebellum, and cerebral cortex<br>Some amyloid PET positive<br>↓ striatal DA & cortical ACh neurotransmission	MRS: ↓ NAA/Cr ↓ Glmn/Glmt<br>DTI: ↓ white matter integrity in temporal lobe, medial parietal lobe, visual association areas<br>ASL/SPECT: ↓ perfusion in posterior cortex
Parkinson's disease (no dementia)	Motor dysfunction (spontaneous motor parkinsonism, visual hallucinations, etc.)	Less atrophy than in PDD/DLB but with a similar anatomic distribution	Similar but less severe functional and connectivity changes to those seen in PDD/DLB	[18F]FDG: ↓ metabolism in the basal ganglia, thalamus, and cerebral cortex<br>↓ DA, 5-HT, ACh, GABA, opoid neurotransmission<br>↑ activated microglia in striatal and extrastriatal regions	Similar but less severe changes on MRS, DTI, and ASL/SPECT to those seen in PDD/ DLB
Huntington's disease	Motor dysfunction with cognitive impairment (bradykinesia, incoordination, etc.); linked to mutation in HTT gene	Atrophy of striatum, cerebral cortex, cingulate, thalamus, and white matter regions	↑ or ↓ activation during motor & cognitive tasks<br>Altered connectivity in cortical-striatal network & DMN	[18F]FDG: ↓ cortical metabolism<br>↓ DA receptors<br>↑ activated microglia in striatum, extra-striatal regions, hypothalamus	DTI: ↓ integrity in frontal lobe, sensorimotor cortex, CC, internal capsule, and basal ganglia
Multiple sclerosis (MS)	Heterogeneous symptoms (autonomic, visual, motor, &/or sensory dysfunction)	Focal hyperintense lesions in white matter on T2-weighted scans; cerebral and cerebellar atrophy	↑ or ↓ activation during memory, attention, and executive tasks<br>Altered connectivity in salience, working memory, sensorimotor, visual networks, & DMN	[18F]FDG: ↓ metabolism in thalamus, deep gray matter structures, & frontal lobe<br>↑ activated microglia in normal and lesioned gray matter and white matter	DTI: ↓ integrity of normal and lesioned white matter and gray matter<br>ASL/SPECT: ↓ cerebral perfusion
HIV-Associated neurocognitive disorder	Impairment in executive function, motor speed, attention/working memory and episodic	Gray matter atrophy in anterior cingulate, lateral temporal lobe, and cerebral cortex; Cortical thinning in primary motor and sensory cortices; white matter atrophy in	↑ or ↓ activation during memory, attention, executive function, and motor tasks<br>Altered connectivity in frontostriatal network, DMN, salience network, and control network;	[18F]FDG: ↓ cortical metabolism but ↑ metabolism in the basal ganglia	MRS: ↓ NAA, ↑ mIns, choline, choline/Cr, mIns/Cr in frontal gray matter, white matter, and basal ganglia<br>DTI: ↓ integrity in cortical white matter, CC, and corona radiata<br>ASL/SPECT: ↓ perfusion in lateral frontal and inferior medial parietal regions; ↑ perfusion in parietal white

NIH-PA Author Manuscript NIH-PA Author Manuscript NIH-PA Author Manuscript

Disease	Clinical symptoms	Atrophy pattern	Functional activation/connectivity changes	Molecular changes	Other imaging
	memory; some motor dysfunction	midbrain and frontal lobe	loss of internetwork connectivity between DMN and a dorsal attention network	↓ DA transporter in basal ganglia	matter and deep gray matter structures
Sporadic/variant CJD; genetic CJD/GSS/FFI	Motor dysfunction, cognitive impairment, and psychiatric symptoms; FFI also features insomnia	Abnormalities of the basal ganglia, thalamus cerebellum, cortical gray matter & white matter	n/a	[18F]FDG: ↓ cortical metabolism	MRS: ↓ NAA, ↑ mIns

Abbreviations: 5-HT, serotonin; ACh, acetylcholine; ASL, arterial spin labeling; CC, corpus callosum; CJD, Creutzfeldt-Jakob disease; Cr, creatinine; CST, corticospinal tract; DA, dopamine; DMN, default-mode network; DTI, diffusion tensor imaging; EC, entorhinal cortex; FDG, [18F] fluorodeoxyglucose; FFI, fatal familial insomnia; FTD, frontotemporal dementia; Glmn, glutamine; Glmt, glutamate; GSS, Gerstmann-Straussler-Scheinker disease; Hipp, Hippocampus; MCI, mild cognitive impairment; mIns, myo-inositol; MRS, magnetic resonance spectroscopy; MTL, medial temporal lobe; NAA, N-acetylaspartate; PET, positron emission tomography; PLIC, posterior limb of the internal capsule; SPECT, single-photon emission computerized tomography.

Statistics of highlighted entities of class BIM (page 1)

Entity	Frequency
Manuscript	163
manuscript	54
SPECT	37
tomography	21
performance	8
Group	5
standard	3
image	3
article	2
event	2
group	2
Brief	1
interface	1
manual	1
report	1
contribution	1
Access	1
brief	1
spect	1

Statistics of highlighted entities of class NIFT (page 1)

Entity	Frequency
atrophy	77
white matter	68
PET	46
MRI	45
frontal lobe	44
SPECT	37
gray matter	36
thalamus	36
perfusion	34
fMRI	31
white matter integrity	26
parietal lobes	22
Atrophy	21
insula	20
parietal lobe	19
ASL	19
MRS	19
FDG PET	18
NAA	17
brain atrophy	16
striatum	16
putamen	15
magnetic resonance imaging	15
dopamine	14
Alzheimer disease	14
amyloid deposition	14
diffusion tensor imaging	14
positron emission tomography	14
[11C]PiB	13
tau	13
cerebral cortex	12
hippocampus	12
hypoperfusion	11
hypometabolism	11
corpus callosum	11
cerebellum	11
structural MRI	10
cerebral atrophy	10
DWI	10
Amyloid PET	9

Statistics of highlighted entities of class NIFT (page 2)

Entity	Frequency
hippocampal	9
precuneus	9
occipital lobe	9
frontal lobes	9
FA	8
brain regions	8
posterior cortical atrophy	8
occipital lobes	8
cerebral perfusion	8
PCA	8
brain metabolism	7
entorhinal cortex	7
hippocampal atrophy	7
temporal lobe atrophy	6
Magnetic resonance imaging	6
precentral gyrus	6
functional MRI	6
Hypometabolism	6
voxel-based morphometry	6
magnetic resonance spectroscopy	6
acetylcholine	5
thalamic	5
cortical atrophy	5
Parkinson disease	5
serotonin	5
Diffusion tensor imaging	5
globus pallidus	4
substantia nigra	4
amygdala	4
arterial spin labeling	4
Diffusion tensor MRI	4
functional magnetic resonance imaging	4
Structural MRI	4
Pittsburgh compound B	3
cerebellar atrophy	3
perfusion imaging	3
cerebral blood flow	3
Gray matter atrophy	3
microbleeds	3
Hippocampal	3

Statistics of highlighted entities of class NIFT (page 3)

Entity	Frequency
diffusion tensor MRI	3
neocortex	3
cerebral glucose metabolism	3
microhemorrhages	3
white matter lesion	3
midbrain	3
amyloid PET	3
PET tracers	3
gray matter atrophy	3
BOLD	2
cortical thickness	2
fractional anisotropy	2
FLAIR	2
Posterior cortical atrophy	2
functional imaging	2
White matter	2
Functional MRI	2
Functional magnetic resonance imaging	2
Cortical thinning	2
pulvinar sign	2
corpus callosum atrophy	2
resting-state fMRI	2
cortical thinning	2
PiB	2
Structural imaging	2
Cerebral blood flow	2
Frontal lobe	2
Amyloid imaging	2
arterial spin-labeling	2
Voxel-based morphometry	2
[18F]fluorodeoxyglucose	2
insular	2
voxel based morphometry	2
[18F] fluorodeoxyglucose	2
Dopamine	2
cerebral blood volume	2
diffusion-weighted MRI	2
pons	2
cerebral metabolism	2
FDG-PET	2

Statistics of highlighted entities of class NIFT (page 4)

Entity	Frequency
Fractional anisotropy	2
Hypoperfusion	2
N- acetylaspartate	2
atrophy of the left temporal lobe	1
temporal atrophy	1
positron-emission tomography	1
supramarginal gyrus	1
amyloid deposits	1
blood oxygenation level dependent (BOLD)	1
pattern of atrophy	1
(1) H-MRS	1
mean diffusivity	1
Alzheimer Disease	1
Arterial spin labeling	1
whole-brain atrophy	1
Diffusion-weighted imaging	1
hippocampal volume	1
temporal pole	1
Acetylcholine	1
bold fMRI	1
PIB	1
1H MRS	1
superior temporal sulcus	1
glucose hypometabolism	1
Cortical thickness	1
inferior frontal gyrus	1
N-acetylaspartate	1
middle temporal gyrus	1
rates of atrophy	1
Pittsburgh Compound B	1
entorhinal volume	1
default mode network connectivity	1
myo-inositol	1
Grey matter volume	1
cingulate gyri	1
Resting-state fMRI	1
left anterior temporal lobe atrophy	1
occipital lobe hypometabolism	1
Brain atrophy	1
whole brain atrophy	1

Statistics of highlighted entities of class NIFT (page 5)

Entity	Frequency
spect	1
blood oxygen levels	1
T2-weighted imaging	1
left caudate	1
structural magnetic resonance imaging	1
postcentral gyri	1
cerebrospinal fluid	1
postcentral gyrus	1
2-[(18)F]fluoro-2-deoxy-D-glucose	1
Amyloid deposition	1
Diffusion Tensor Imaging	1
left putamen	1
Dynamic susceptibility contrast MR imaging	1
cerebellar	1
Mean diffusivity	1
Cerebral perfusion	1
supramarginal gyri	1
Frontal lobe atrophy	1
single photon emission computed tomography	1
Hippocampal atrophy	1
fluid attenuated inversion recovery	1
hyperintensity	1
(PET) tracers	1
gyrus rectus	1
99mTc-ECD	1
atrophy of anterior temporal lobes	1
(FA)	1
white matter atrophy	1
superior frontal gyrus	1
resting-state functional magnetic resonance imaging	1
nucleus basalis of Meynert	1
parahippocampal gyrus	1
blood oxygenation	1
depression	1
Thalamic	1
Positron emission tomography	1
FMRI	1
near-infrared spectroscopy	1
oxygen metabolism	1
Cerebral glucose metabolism	1

Statistics of highlighted entities of class NIFT (page 6)

Entity	Frequency
White matter atrophy	1
Brain metabolism	1
proton MR spectroscopy	1
Diffusion-weighted MRI	1
occipital pole	1
Perfusion	1
18F-dopa	1
structural imaging	1
[11C] PiB	1
neuronal integrity	1
PET ligands	1
cortex thinning	1
neocortical	1
blood oxygen level	1
CSF	1
Whole-brain atrophy	1
inferior parietal lobule	1
frontal lobe hypometabolism	1
Single photon emission computed tomography	1
whole brain volume	1
rCBF	1
Pittsburgh compound-B	1
Corpus callosum	1
angular gyrus	1
fusiform gyrus	1
focal cortical atrophy	1
Cerebral atrophy	1
Medial temporal lobe atrophy	1
volumetric MRI	1
white-matter	1
11C PiB	1
diffusion tensor imaging tractography	1
[123I]-iodobenzovesamicol	1
diffusion tensor MR imaging	1
computerized tomography	1
subcortical atrophy	1

Statistics of highlighted entities of class QIBO (page 1)

Entity	Frequency
disease	265
brain	120
activation	94
cortex	65
cerebral	65
Brain	64
cortical	62
diagnosis	30
Disease	26
hippocampal	17
striatum	16
blood	15
positron emission tomography	14
areas	14
Cerebral	12
hippocampus	12
volume	11
cerebellum	11
preclinical	10
Cortical	9
syndrome	8
cortices	8
area	6
neurotransmitter	6
magnetic resonance spectroscopy	6
Activation	5
correlation	5
density	5
hypothalamus	5
Cortex	4
Hippocampal	4
cells	4
cerebellar	4
tomography	3
thickness	3
inflammation	3
cell	3
vascular disease	3
cerebral blood flow	3
Diagnosis	2

Statistics of highlighted entities of class QIBO (page 2)

Entity	Frequency
ischemia	2
diffusion-weighted MRI	2
Cerebral blood flow	2
Functional MRI (fMRI)	1
Staging	1
amino acid	1
cell proliferation	1
positron-emission tomography	1
prognosis	1
single photon emission computed tomography	1
Preclinical	1
myocardial infarction	1
Single photon emission computed tomography	1
staging	1
class	1
Perfusion MRI	1
Positron emission tomography	1
hypertension	1
sarcoma	1
model	1
apoptosis	1
atherosclerosis	1
T2-weighted MRI	1
Syndrome	1
Diffusion-weighted MRI	1
length	1
carbon 11	1
Volume	1
central nervous system	1
PET/CT	1
arterial spin labeling MRI	1

Key for highlighting of entities

Class	Color	Overall Frequency
BIM		308
NIFT		1334
QIBO		1056
