# Supplementary material for: Neuroimaging Feature Terminology: A Controlled Terminology for the Annotation of Brain Imaging Features
Source: J Alzheimers Dis. 2017 Aug 14;59(4):1153–69. doi: 10.3233/JAD-161148 (PMC5611802; doi:10.3233/JAD-161148)
Supplement: Supplementary file 1 [file jad-59-jad161148-s001.zip › Supplementary_File1/Update on the Biomarker Core of the Alzheimer's Disease.pdf]

NIH Public Access
Author Manuscript
Alzheimers Dement. Author manuscript; available in PMC 2011 May 1.

Published in final edited form as:
Alzheimers Dement. 2010 May ; 6(3): 230–238. doi:10.1016/j.jalz.2010.03.008.

Update on the Biomarker Core of the Alzheimer's Disease Neuroimaging Initiative Subjects

John Q Trojanowski, M.D., Ph.D., **John Q Trojanowski, MD, PhD**, **Hugo Vandeerstichele, PhD**, **Magdalena Korecka, PhD**, **Christopher M Clark, MD**, **Paul S Aisen, MD**, **Ronald C Petersen, MD**, **Kaj Blennow, MD, PhD**, **Holly Soares, PhD**, **Adam Simon, PhD**, **Piotr Lewczuk, MD**, **Robert Dean, MD**, **Eric Siemers, MD**, **William Z Potter, MD**, **Michael W Weiner, MD**, **Clifford R Jack Jr, MD**, **William Jagust, MD**, **Arthur W Toga, PhD**, **Virginia M.-Y. Lee, PhD**, and **Leslie M Shaw, PhD**

Abstract

Here we review progress by the Penn Biomarker Core in the Alzheimer's disease Neuroimaging Initiative (ADNI) towards developing a pathological cerebrospinal fluid (CSF) and plasma biomarker signature for mild Alzheimer's disease (AD) as well as a biomarker profile that predicts conversion of mild cognitive impairment (MCI) and/or normal control (NC) subjects to AD. The Penn Biomarker Core also collaborated with other ADNI Cores to integrate data across ADNI to temporally order changes in clinical measures, imaging data and chemical biomarkers that serve as mileposts and predictors of the conversion of NC to MCI as well as MCI to AD, and the progression of AD.

Initial CSF studies by the ADNI Biomarker Core revealed a pathological CSF biomarker signature of AD defined by the combination of Aβ1-42 and total tau (T-tau) that effectively delineates mild AD in the large multisite prospective clinical investigation conducted in ADNI. This signature appears to predict conversion from MCI to AD. Data fusion efforts across ADNI Cores generated a model for the temporal ordering of AD biomarkers which suggests that Aβ amyloid biomarkers become abnormal first, followed by changes in neurodegenerative biomarkers (CSF tau, FDG-PET, MRI) and the onset of clinical symptoms. The timing of these changes varies in individual patients due to genetic and environmental factors that increase or decrease an individual's resilience in response to progressive accumulations of AD pathologies. Further studies in ADNI will refine this model and render the biomarkers studied in ADNI more applicable to routine diagnosis and to clinical trials of disease modifying therapies.

Keywords

Alzheimer's disease; cerebrospinal fluid; plasma; biomarkers; mild cognitive impairment

Corresponding Author: Dr. John Q Trojanowski, M.D., Ph.D., University of Pennsylvania School of Medicine.

Publisher's Disclaimer: This is a PDF file of an unedited manuscript that has been accepted for publication. As a service to our customers we are providing this early version of the manuscript. The manuscript will undergo copyediting, typesetting, and review of the resulting proof before it is published in its final citable form. Please note that during the production process errors may be discovered which could affect the content, and all legal disclaimers that apply to the journal pertain.

NIH-PA Author Manuscript
NIH-PA Author Manuscript
NIH-PA Author Manuscript

NIH-PA Author Manuscript
NIH-PA Author Manuscript
NIH-PA Author Manuscript

Introduction[1]

Alzheimer's Disease and the Quest for Informative Biomarkers

Alzheimer's disease (AD) is the most common dementia [1,2], and the hallmark lesions of AD are Aβ plaques and neurofibrillary tangles (NFTs) formed by abnormal tau. Clinical symptoms closely relate to NFTs, neurodegeneration and synapse loss [3-5]. AD can be divided into a pre-symptomatic phase in which subjects are cognitively normal but have AD pathology, a prodromal phase known as mild cognitive impairment (MCI) and a third phase when patients show dementia with impairments in multiple domains and loss of function in activities of daily living [4,6-8]. Although it has been suggested that diagnostic criteria for early AD should be redefined by the presence of memory impairments plus biomarker evidence of AD [9], this is still controversial despite numerous studies showing that AD biomarkers predict conversion from MCI to AD [10-19], and the diagnosis of AD still requires the presence of dementia [20]. However, it is timely to consider developing strategies to identify AD onset before symptom onset to optimize potential efficacy of disease modifying therapies, and to enable drug development aimed at AD prevention. Initial data emerging from ADNI offer encouragement that success in accomplishing this may be close at hand. Thus, here we summarize progress by the Penn Biomarker Core of ADNI towards developing a pathological cerebrospinal fluid (CSF) and plasma biomarker signature for mild AD subjects as well as a biomarker profile that predicts conversion of MCI and normal control (NC) subjects to AD. Further, the Penn Biomarker Core has collaborated with other ADNI Cores in data fusion efforts across ADNI to temporally order changes in clinical measures, imaging data and chemical biomarkers that serve as mileposts and predictors of the conversion of NC to MCI as well as MCI to AD, and the progression of AD. Thus, we also briefly summarize this work and the hypothetical model of temporal changes in AD biomarkers which emerges now from the initial funding period of ADNI (ADNI-1) and informs future AD Biomarker Core studies in the ADNI renewal period (ADNI-2) and the ADNI Grand Opportunity (ADNI-GO) grant.

ADNI Biomarker Core Progress: 2004-2009

The Penn Biomarker Core made considerable progress to implement the Aims of ADNI-1 including: 1) the establishment of biofluid collection, 2) the shipping and storage standard operating procedures (SOPs), 3) establishing an archive of ADNI biofluids, and 4) the launch of our studies of these fluids (see also the ADNI website http://www.adni-info.org/index where all biomarker data are posted). Other progress includes establishing the Resource Allocation Review Committee (RARC), conducting meetings with ADNI Industrial Scientific Advisory Board (ISAB) members, meetings with international biomarker scientists, establishing an international CSF quality control program for continuing quality assessment of biomarker assays in World Wide ADNI (WW-ADNI), and support for ADNI “add-on” studies. In keeping with the ADNI mission, all ADNI biomarker data are posted on the ADNI website after they are obtained following data analyses and quality assessment.

Development of SOPs for Biofluid Collection, Shipping, Aliquoting, Storage and Curation—The Penn Biomarker Core established ADNI biomarker SOPs at the outset of ADNI-1. This was done in consultation with ISAB and other biomarker scientists to establish consensus for collection, handling, shipment, labeling, aliquoting, storage and tracking 24/7×365 days/year of DNA, CSF, plasma, serum and urine samples. We also worked closely with the ADNI Clinical Core to develop biofluid tracking forms that provide a detailed history

[1]Since this is an update on the progress of the Penn Biomarker Core of ADNI-1, most citations listed here are from progress by this Core since 2004 and they are identified by *. Due to the explosion in publications on AD biomarkers, research from other centers are mainly cited in the reviews listed here.

NIH-PA Author Manuscript NIH-PA Author Manuscript NIH-PA Author Manuscript

for each sample. These SOPs are essential to assure: a) sample integrity; b) accurate identification of samples received and aliquots prepared from them; c) sample stability.

An example of the value of detailed characterization of each collected biofluid is documenting the time to freezing on dry ice at study sites. Thus, for the RARC approved proteomic studies of ADNI plasma and CSF samples, data on the time at room temp before freezing each sample could be informative for interpreting results on time/temp sensitive AD biomarkers, and detailed sample history permits selection of samples using a specific time stipulation, e.g. 1-2 hrs at room temp, but not longer for plasma samples. Another aspect of sample timing involves CSF collection where knowing the length of time from collection to time of transfer is important. For example, some early CSF samples were mistakenly collected into polystyrene collection tubes at the sites. This was rapidly corrected, but this information enabled the Biomarker Core to understand the effects of this collection error, and simulations of the effects of brief exposure to polystyrene alleviated concerns about this potential confound. Finally, to illustrate the fastidiousness with which ADNI sites obtained and processed biofluid samples, the average time CSF was in contact with any transfer tube was 25.7 min for ADNI Baseline CSF samples thereby limiting significant exposure time to any inappropriate CSF collection tube.

Current Status of the ADNI-1 Biofluid Archive—Shortly after SOPs for the collection, processing, bar code labeling, packaging and shipment of biofluids for ApoE genotyping, cell immortalization and biomarker studies were finalized, these SOPs were incorporated into the ADNI procedure manual, distributed to all ADNI sites and the first ADNI biofluids started to arrive at the Penn Biomarker Core in August 2005. Through April 30, 2007 a total of 1108 blood samples were collected at the Screening visit and all were received and rapidly processed for ApoE genotyping at Penn so results could be entered into the ADNI database within a week of receipt to balance the ADNI cohort for ApoE status. Residual blood samples were stored at -80 °C for DNA preparation and genetic studies. As of June, 2009 a total of 12,053 ADNI biofluid samples have been received and processed. To respond to the request of the ADNI Clinical Core that clinic visits take place 5 days/week, we arranged for receipt and freezer storage of biofluids 6 days/week including Saturdays. Thus far, a total of 119,106 aliquots of serum, plasma, CSF and urine have been prepared, bar code labeled and stored in dedicated ADNI freezers at -80 °C. Temperature monitoring of each freezer is done 24/7/365 with a telephone alarm system and one Penn Biomarker Core staff person is always "on-call" to respond to an alarm. For each primary biofluid sample collected, the following information is maintained in the ADNI Biomarker Core database at Penn: biofluid type (CSF, plasma, serum, urine), coded subject and visit ID, 6 digit license plate number, visit date and time, date and time of receipt, condition of samples as received, biofluid sample volume and number of aliquots. The database is backed up daily on an external "brick" hard drive and on a DVD disk. The latter are stored outside the Biomarker Core laboratory in a secure location in a different building to assure data security in the event of a catastrophic failure of the server on which the database resides.

Round Robin Study to Validate and Standardize Methods to Measure CSF Tau and Aβ—To reliably measure Aβ and tau in ADNI CSF samples, the Penn ADNI Biomarker Core identified several sources of variation in quantifying T-tau, tau phosphorylated a threonine 181 (P-tau$_{181p}$) and Aβ$_{1-42}$ that were shown by Luminex or ELISA methods to have at least 85% sensitivity and 80% specificity for diagnosing AD, predicting MCI progression to AD, and identifying elderly Clinical Dementia Rating (CDR) scale 0 individuals likely to progress to CDR>0 [*16,*21]. We also validated the INNO-BIA AlzBio3 reagents (Innogenetics, Belgium[2]) in a 7 site study that included academic and ISAB sites.

NIH-PA Author Manuscript NIH-PA Author Manuscript NIH-PA Author Manuscript

A pre-qualification, as well as a qualification study was done using a standardized protocol for testing. The pre-qualification round provided the required experience for each participating site. Each run of the qualification study included the use of seven ready-to-use calibrator mixtures for assay calibration, two control samples which are included in the kit for run acceptance, three run validation samples (prepared by the addition of reference materials in diluent (a pre-defined combination of synthetic Aβ_{1-42} and P-tau$_{181p}$ and recombinant tau protein), and 5 pools of CSF, including 2 CSF pools prepared from AD patient CSF samples (0.5 mL aliquots into polypropylene tubes, frozen at -80 °C). Shipping of the samples to participants included temperature monitoring. Three analytical runs were completed using the standardized test protocol. Briefly, the precision study demonstrated the following:

1. Repeatability and Reproducibility

Within each of the 7 centers, the within-center %CV [combined within-run and between run precision] for measurement of the three CSF biomarkers was ~10% over the 3 runs for each biomarker. For the CSF pools, the between-center variability was greater than that for the aqueous-based controls, and studies are underway to explain differences between laboratories. Further, we collaborate with the WW-ADNI CSF biomarker quality control program to improve laboratory performance of these tests. Finally, results using the pre-made calibrators are highly reproducible between the 7 centers (for more details see the report "ADNI Interlaboratory Study" posted on the ADNI website).

2. Sample stability

Stability of the 3 aqueous-based and 3 CSF pools was demonstrated for the 8 month interval between the pre-qualification and qualification studies. Ongoing studies in the Biomarker Core are evaluating longer term stability. Notably, the effect of brief exposure of 20 freshly obtained CSF samples to polystyrene for 1 hr at room temp, as compared to polypropylene, decreased the concentration of Aβ_{1-42}, by 14.5%, and tau by 11%, but there was no change in P-Tau$_{181P}$ concentration (a collaborative study with Kaj Blennow).

3. ADNI CSF sample analyses

Tolerance intervals were generated from the CSF pool data for the Biomarker Core as a guide for the acceptability of analytical runs of ADNI CSF samples.

4. Publication of results

These data together with the pre-qualification study data now are being prepared for publication [Shaw et al, In preparation]. This paper reports on data from individual participating laboratories regarding other aspects of CSF biomarker measurements related to pre-analytical sample handling (freeze-thaw, long-term/short-term stability; diurnal variation), as well as instrumentation aspects (comparison of instruments and algorithms). All these data were posted on the ADNI website following completion and brief summaries for the ADNI sites were prepared and distributed at ADNI meetings as well as placed on the ADNI website. Moreover, we published a detailed review [*21] of the steps and procedures required to bring potential AD biomarkers from concept to their implementation as useful biomarkers with particular emphasis on how this is done for the multiplexed analysis of CSF Aβ and tau.

Baseline Studies of CSF Tau and Aβ in >400 ADNI Subjects—This study of ADNI baseline CSF samples sought to develop a pathological CSF biomarker signature for AD [*16]. To do this, Aβ_{1-42}, T-tau) and P-tau$_{181}$ were measured in: a) CSF samples obtained at baseline for 100 mild AD, 196 MCI and 114 elderly NC subjects in ADNI; b) an independent set of 56

[2]These reagents are for research only and not for use in any diagnostic procedures listed here.

NIH-PA Author Manuscript
NIH-PA Author Manuscript
NIH-PA Author Manuscript

autopsy-confirmed AD subjects and 52 age-matched elderly NC followed in the NIA funded Penn AD Core Center (ADCC) with ADNI SOPs using the Luminex platform based multiplex immunoassay [*16,*21]. Detection of an AD CSF profile for T-tau, P-tau_{181} and $A\beta_{1\text{-}42}$ in ADNI subjects was achieved using receiver operating characteristic (ROC) cutpoints and logistic regression models derived from the autopsy-confirmed CSF biomarker data. Our data showed that CSF $A\beta_{1\text{-}42}$ was the most sensitive biomarker for AD detection in CSF from non-ADNI autopsy-confirmed subjects with an ROC area under the curve of 0.913 and sensitivity for AD detection of 96.4%. A unique bimodal characteristic of the distribution of CSF $A\beta_{1\text{-}42}$ was detected in each ADNI subgroup, and a logistic regression model for $A\beta_{1\text{-}42}$, T-tau and ApoEε4 allele count provided the best delineation of mild AD. An AD-like pathological baseline CSF profile for T-tau/$A\beta_{1\text{-}42}$ was detected in 33/37 ADNI MCI subjects who converted to probable AD during the first year of the study. Based on these data, we conclude that the pathological CSF biomarker signature of AD defined by the combination of $A\beta_{1\text{-}42}$ and T-tau in the Penn autopsy-confirmed AD cohort and tested in the cohort followed in ADNI for 12 months effectively detects mild AD in a large multisite prospective clinical investigation, and this signature appears to predict conversion from MCI to AD.

The cutoff values established by Shaw et al [*16] were validated in a follow up study with EU-ADNI and ISAB collaborators [*22] wherein we sought to identify AD biomarker patterns in an independent, unsupervised manner, without clinical diagnoses using a mixture modeling approach to analyze the ADNI CSF Aβ42, T-tau and P-tau_{181} data in Shaw et al [*16]. This analysis was validated on two additional data sets, one of which was an autopsy-confirmed EU cohort. Using the ADNI data set, a CSF Aβ1-42/P-tau_{181} biomarker mixture model identified one feature linked to AD, while the other matched the NC status. The AD signature was found in 90%, 72%, and 36% of patients in the AD, MCI, and NC groups, respectively. The NC group with the AD signature was enriched in ApoEε4 allele carriers.

Further, we collaborated with Hopkins investigators outside ADNI to investigate the effect of CSF abnormalities on rate of decline in everyday function in NC, MCI and AD [*23]. Briefly, CSF T-tau, P-tau_{181}, and $A\beta_{42}$ data from 114 NC, 195 MCI patients, and 100 mild AD ADNI subjects and their Functional Activities Questionnaire (FAQ) and ADAS-Cog data were analyzed by random effects regressions. All CSF analytes were associated with functional decline in MCI, and all but T-tau/$A\beta_{42}$ were associated with functional decline in controls. Among controls, P-tau_{181} was the most sensitive to functional decline whereas in MCI it was $A\beta_{42}$. CSF biomarkers were uniformly more sensitive to functional decline than the ADAS-Cog among controls and variably so in MCI, whereas the ADAS-Cog was more sensitive than CSF biomarkers in AD. The impact of CSF biomarkers on functional decline in MCI was partially mediated by their impact on cognitive status. Across all diagnostic groups, persons with a combination of tau and $A\beta_{42}$ abnormalities exhibited the steepest rate of functional decline. These data indicate that CSF tau and Aβ42 abnormalities are associated with functional decline, and thus with future development of AD in controls and MCI patients. However, they are not predictive of further functional degradation in AD. Hence, persons with AD-like CSF tau and $A\beta_{42}$ abnormalities are at greatest risk of functional impairment.

Assessment of Sulphatides, Homocysteine and Isoprostanes as AD Biomarkers in ADNI-1

Sulphatides: Plans to validate CSF sulphatides as AD biomarkers in ADNI-1 were based on promising reports in 2004, but subsequent studies did not confirm these findings so we did not pursue sulphatides further. Therefore, we re-budgeted the savings from this change to support the 2.5-fold increase in the number of CSF samples we obtained, i.e. an increase from 20% of all ADNI subjects described in the original application to >50% after ADNI-1 was launched.

NIH-PA Author Manuscript NIH-PA Author Manuscript NIH-PA Author Manuscript

Homocysteine: ADNI-1 included plans to study homocysteine in plasma and CSF. Using a validated enzyme immunoassay methodology, we measured homocysteine in 813 baseline ADNI plasma samples. These revealed that there was no significant difference in mean plasma homocysteine concentration in AD vs MCI, but there was between NC and AD ($p<0.01$) and NC and MCI ($p<0.01$). These data are consistent with previous studies showing an association between elevated baseline plasma homocysteine concentration and risk for development of AD [*24]. Inclusion of homocysteine in the logistic regression model described above for $A\beta_{1-42}$, T-tau and ApoEε4 allele number showed it was non-significant as a variable in this model so the value of measuring plasma homocysteine levels is uncertain. However, additional analyses are underway for plasma homocysteine including correlations with other biomarkers and longitudinal changes. Measurement of homocysteine concentrations in CSF was achieved in 410 CSF samples collected at baseline using a validated enzyme immunoassay (developed and performed by Merck) designed specifically to measure the much lower concentrations present in this biofluid as compared to plasma. However, these studies showed no significant difference between NC and either AD or MCI in mean homocysteine values.

Isoprostanes: Since earlier studies suggested that quantification of F(2)-Isoprostanes is a reliable index of oxidative stress in vivo and is valuable in the diagnosis and monitoring of AD [reviewed in *24], the most relevant isoprostanes were studied in CSF, plasma and urine as described in the ADNI-1 application. To do this, we developed and validated a semiautomated high-throughput HPLC tandem mass spectrometry assay for the quantification of 8-iso-PGF_{2a} in human urine and plasma [*25]. Briefly, after protein precipitation, samples were injected into the HPLC system and extracted online. The extracts were then back-flushed onto the analytical column and detected with an atmospheric pressure chemical ionization-triple quadrupole mass spectrometer monitoring the deprotonated molecular ions [M-H](-) of 8-iso-PGF_{2a} (m/z = 353-->193) and the internal standard 8-iso-PGF_{2a},8-iso-PGF_{2a} -d(4) (m/z = 357-->197). We then applied these methods to studies of human urine and plasma and showed that the assay was linear from 0.025 to 80 microg/L and in human plasma from 0.0025 to 80 microg/L (r(2)>0.99). Inter-day accuracy and precision for concentrations above the lower limit of quantification were <10%. Concentrations of 8-iso-PGF_{2a} in urine of 16 NC individuals ranged from 55-348 ng/g creatinine. In 16 plasma samples from NC individuals, free 8-iso-PGF_{2a} was detectable in all samples and concentrations were 3-25 ng/L. These data show that our assay meets all method performance criteria, allows for analysis of >80 samples/day, and has the sensitivity to quantify 8-iso-PGF_{2a} concentrations in plasma and urine from NC individuals.

Next, we undertook validation studies of a liquid chromatography method with tandem mass spectrometry detection for simultaneous analysis of 8-iso-PGF_{2a} and 8,12-iso-iPF_{2a}-VI (*11). Notably, these are the most frequently studied isoprostanes in human CSF and brain tissue [*24,*25]. An API 5000 triple quadrupole instrument with an APCI ion source was used in this study. We used this method to quantify both isoprostanes in CSF from non-ADNI Penn AD patients and age matched NC as well as in postmortem brains from AD and non-AD controls. Our results do not confirm some previous reports that the CSF isoprostanes studied here are useful AD biomarkers. However, we currently conduct similar isoprostane assays of ADNI plasma samples to determine if plasma isoprostanes are informative for risk for conversion from MCI to AD since the elderly population studied here will have increased risk for cardiovascular and cerebrovascular disease which could affect risk for AD.

Cross-sectional and Longitudinal Measures in NC, MCI and AD Subjects in ADNI—In collaboration with Petersen et al. [*26], we characterized NC, MCI and mild AD subjects to enable the assessment of the utility of neuroimaging and chemical biomarker measures in 819 subjects (229 NC, 398 with MCI and 192 AD) enrolled at baseline in ADNI and followed for 12 months. The MCI subjects were more memory impaired than the NC subjects, but not as impaired as the AD subjects. Non-memory cognitive measures were minimally impaired in

NIH-PA Author Manuscript

MCI subjects. The MCI subjects progressed to dementia in 12 months at a rate of 16.5% per year and ~50% of the MCI subjects were on anti-dementia therapies. There was minimal movement on the ADAS-Cog for the NC subjects, slight movement of the MCI subjects of 1.1 and a modest change for the AD subjects of 4.3. Baseline CSF measures of Aβ42 separated the three groups as expected [*16] and successfully predicted the 12-month change in cognitive measures. Thus, the 12-month progression rate of MCI was as predicted, and the CSF measures heralded progression of clinical measures over 12 months.

Ventricular Expansion and CSF Biomarkers in NC, MCI and AD Subjects in ADNI—A collaborative study with the Thompson lab [*27] sought to improve on the single-atlas ventricular segmentation method using multi-atlas segmentation. We also calculated minimal numbers of subjects needed to detect correlations between clinical scores and ventricular maps. Correlations were analyzed between AD tau and Aβ42 CSF biomarkers [*16] and localizable deficits in the brain, in 80 AD, 80 MCI, and 80 NC from ADNI. Lower CSF Aβ42 protein levels were correlated with lateral ventricular expansion, and these studies show that ventricular expansion maps correlate with pathological CSF and cognitive measures in AD.

Combined Analysis of PIB, PET, CSF Biomarkers and Cognition in ADNI Subjects—Collaborations with the Jagust lab [*28] compared PIB-PET, FDG-PET and CSF measures of Aβ42, T-tau and P-tau$_{181}$ in 10 AD, 11 NC and 34 MCI ADNI subjects. There was substantial agreement between PIB-PET and CSF Aβ42 measures (91% agreement, kappa = 0.74), modest agreement between PIB-PET and P-tau (76% agreement, kappa = 0.50) and minimal agreement for other comparisons (kappa < 0.3). The MMSE score was significantly correlated with FDG-PET but not with PIB-PET or CSF Aβ42. Regression models showed that PIB-PET was significantly correlated with Aβ42, T-tau and P-tau$_{181}$, while FDG-PET only correlated with Aβ42. Thus, PET and CSF biomarkers of Aβ agree with one another while FDG-PET is modestly related to other biomarkers, but is better related to cognition.

Tensor Based Imaging, CSF Biomarkers and Cognition in ADNI Subjects—In a collaborative study with Leow et al. [*29], the anatomical distribution of longitudinal brain structural changes were compared over 12 months in 20 AD, 40 NCs and 40 MCI ADNI subjects. We detected widespread cerebral atrophy in AD, and a more restricted atrophic pattern in MCI. In MCI, temporal lobe atrophy rates were correlated with changes in MMSE scores, CDR, and logical/verbal learning memory scores. In AD, temporal atrophy rates were correlated with higher CSF P-tau levels, and a greater CSF tau/Aβ42 ratio. Temporal lobe atrophy was significantly faster in MCI subjects who converted to AD than in non-converters. Thus, serial MRI scans relate ongoing neurodegeneration to CSF biomarkers, cognitive changes, and conversion from MCI to AD.

Hippocampal Volume Loss, ApoE Genotype and CSF Biomarkers in early AD—A collaborative study with the Weiner lab [*30] included 112 NC, 226 MCI and 96 AD subjects who had at least three successive MRI scans at 47 ADNI sites. The MCI and AD groups showed hippocampal volume loss over 6 months and accelerated loss over 1 year while increased rates of hippocampal loss were associated with the ApoEε4 allele in AD and lower CSF Aβ42 in MCI irrespective of ApoE genotype. These data supports the concept that increased hippocampal volume loss is an indicator of AD pathology and a potential marker for efficacy of therapeutic interventions in AD.

Comparing MRI and CSF Biomarkers in NC, MCI and AD Subjects In ADNI—Collaborative studies with Cliff Jack's lab [*31,*32] correlated MRI and CSF biomarkers with clinical diagnosis and cognitive performance in NC, MCI and AD. Baseline CSF (T-tau, Aβ1-42 and P-tau$_{181}$) and MRI scans were obtained in 399 subjects (109 NC, 192 MCI, 98

AD). STAND-scores (STructural Abnormality iNDexscore) were computed, and correlated with CDR-SB and MMSE in MCI and AD ($p \leq 0.01$). STAND and all CSF biomarkers were predictors of clinical group (NC, MCI or AD) univariately ($p<0.001$). These studies show that CSF and MRI biomarkers independently contribute to intergroup diagnostic discrimination and the combination of CSF and MRI provides better prediction than either source of data alone. Studies of these subjects were extended, and single-predictor Cox proportional hazard models for time to conversion from MCI to AD showed that STAND and log T-tau/Aβ42 were predictive of future conversion. Thus, these studies show that MRI and CSF tau/Aβ42 provide complimentary predictive information about time to conversion from MCI to AD and combination of the two provides better prediction than either source alone.

Rules Based Medicine (RBM) Studies of CSF and Plasma—ADNI RBM "add-on" studies are in progress now using plasma and CSF samples from the entire ADNI cohort, but data are not yet available. Briefly, the plan is to interrogate these ADNI samples with an RBM panel of ~190 analytes using the Luminex® bead-based immunoassay system to identify novel sets of CSF and plasma AD biomarkers associated with definite AD versus MCI or NC status. Preliminary data from RBM studies of non-ADNI Penn samples identified several potential CSF and plasma AD biomarkers for further investigation in ADNI-1 [Hu et al, submitted] and completion of the RBM interrogation of the ADNI-1 CSF and plasma samples in the coming months will enable independent confirmation or rejection of these analytes for further study in the ADNI competing renewal period (ADNI-2) the application for which was submitted last November.

Round Robin Studies to Determine the Role of Plasma Aβ40/42 as AD Biomarkers—We recently completed our Aβ plasma round robin study that includes 12 academic and industrial laboratories, including ISAB sites. Each site used INO-BIA plasma Aβ kits (Innogenetics, Belgium). Notably, these studies now establish that we can reliably assess the utility of measuring plasma Aβ to predict conversion from MCI to AD and/or to monitor AD progression in studies of plasma Aβ proposed for ADNI-2. Detailed statistical analyses of the full data set are underway, but the reproducibility data achieved by the Penn ADNI Biomarker Core using the Innogenetics multiplex assay for plasma Aβ42/40 in this interlab round robin study look very promising. For example, we were able to achieve within and between day %CVs ranging between 0.9 -4.9% for plasma Aβ42, 1.2 – 6.6% for Aβ40 and up to 7.6% for the ratio of Aβ42/Aβ40.

A Model for the Temporal Ordering of Biomarkers of AD Pathology—The model proposed here and illustrated in Figure 1 is based on the view that AD begins with abnormal processing of amyloid precursor protein (APP) thereby increasing brain Aβ which leads to neuron dysfunction and death [*33-*37]. The model also assumes a lag phase between Aβ deposition and neuron loss, and differences in brain resiliency, plasticity, cognitive reserve or other factors likely account for the variable duration of this lag phase. The presence of additional brain pathologies (e.g. alpha-synuclein, TDP-43 lesions) also may contribute to clinical variations in AD patients. Briefly, the hypothetical model proposed by ADNI investigators in Figure 1 relates AD onset and progression to AD biomarkers based on the following assumptions: 1) these biomarkers become abnormal before clinical symptoms appear; 2) Aβ biomarkers become abnormal before tau and neurodegenerative biomarkers; 3) tau and neurodegenerative biomarkers correlate with clinical disease severity; 4) these biomarkers are temporally ordered. Growing evidence supports these assumptions as reviewed recently in Jack et al. [*36].

NIH-PA Author Manuscript NIH-PA Author Manuscript NIH-PA Author Manuscript

NIH-PA Author Manuscript

NIH-PA Author Manuscript

NIH-PA Author Manuscript

ADNI-2: The Future

Consistent with the goals of ADNI-1 and building on our progress in the first ADNI funding period, the Specific Aims of the Biomarker Core in the ADNI renewal (ADNI-2) are to test our new AD biomarker hypotheses. To do this, we: 1) bank and curate biofluids from ADNI-1, ADNI GO and ADNI-2 subjects; 2) distribute ADNI samples to investigators qualified by the ADNI RARC; 3) study promising AD biomarkers. Selection of biomarkers for study in ADNI-2 is based on: 1) ADNI-1 Biomarker Core studies; 2) our studies of AD biomarkers in non-ADNI cohorts; 3) other AD biomarker research. For example, our ADNI-1 data on CSF Aβ and tau established the importance of longitudinal studies of these analytes, while our Aβ plasma round robin data enable us to determine the utility of measuring plasma Aβ to predict conversion from MCI, including early MCI (eMCI) to AD or to monitor AD progression. Thus, the Specific Aims of the ADNI-2 Biomarker Core are designed to test the following hypotheses:

- **A.** A panel of CSF/plasma biomarkers (rather than any single analyte) will predict conversion from normal to MCI (including early MCI) or to AD and conversion from MCI to AD as well as identify MCI subjects who have stable MCI and do not convert to AD.
- **B.** A panel of CSF/plasma biomarkers will reflect the progression of AD from its prodromal phase through to moderate or severe stages of AD
- **C.** A panel of CSF/plasma biomarkers will predict the likelihood of healthy brain aging or resistance to AD in the NC population.

To accomplish this, the Specific Aims of the Penn Biomarker Core in ADNI-2 are to:

1. Continue to collect, aliquot, store, curate, track all samples collected from subjects in ADNI-1, ADNI GO and ADNI-2.
2. Continue biomarker studies of CSF Aβ42, T-tau and P-tau$_{181p}$ as well as plasma Aβ42 and Aβ40.
3. Validate promising new biomarkers including BACE, and analytes identified by the RBM panel of ~100 analytes.
4. Partner with the ADNI ISAB members and other investigators outside ADNI in RARC approved “add-on” biomarker studies that may include proteomic, metabalomic and lipidomic methodologies.
5. Collaborate with all ADNI Cores/Investigators in analyses of biomarker, clinical, imaging and autopsy data.
6. Collaborate with WW-ADNI Sites in Europe, Japan, Korea, China and Australia in joint studies and comparative analyses of previously collected and new biomarker data.

By implementing these Aims to test our new ADNI-2 hypotheses, we will advance understanding of the applications of validated and new AD biomarkers as predictive, diagnostic and progression markers from NC to early MCI/MCI and thence to AD thereby contributing to the mission of ADNI-2.

Conclusions

The ADNI Biomarker Core at Penn has made substantial progress since ADNI-1 was launched in 2004, as reviewed here. Significantly, as a result of the intense collaborations across the ADNI Cores, we have generated a compelling model of the temporal ordering of AD biomarkers (Fig. 1) that will guide our studies of specific AD biomarkers for diagnosis and disease staging in ADNI-2 [*36]. The staging biomarkers and their temporal relationships with

NIH-PA Author Manuscript NIH-PA Author Manuscript NIH-PA Author Manuscript

the phases of AD discussed here present opportunities to test AD biomarker hypotheses by ADNI investigators in ADNI-2 and by other investigators who mine ADNI data or develop their own biomarker data sets in WW-ADNI or other biomarker initiatives. In addition to their use in diagnostic tests, CSF biomarkers may be valuable in clinical trials, for enrichment of the patient sample with pure AD cases, for patient stratification, as safety markers, and to detect and monitor the biochemical effects of drugs [*37,38]. Thus, in the very near future, we expect that data emerging from North American ADNI, WW-ADNI and other biomarker initiatives will deliver on the promise to provide validated AD biomarkers for a wide variety of applications including predictive testing for AD, diagnosis of AD and for use in clinical trials to assess the response of disease modifying therapies in AD patients.

Acknowledgments

We thank our ADNI colleagues for their contributions to the work summarized here. Data collection and sharing for this project was funded by the Alzheimer's Disease Neuroimaging Initiative (ADNI) (National Institutes of Health Grant U01 AG024904). ADNI is funded by the National Institute on Aging, the National Institute of Biomedical Imaging and Bioengineering, and through generous contributions from the following: Abbott, AstraZeneca AB, Bayer Schering Pharma AG, Bristol-Myers Squibb, Eisai Global Clinical Development, Elan Corporation, Genentech, GE Healthcare, GlaxoSmithKline, Innogenetics,Johnson and Johnson, Eli Lilly and Co., Medpace, Inc., Merck and Co., Inc., Novartis AG, Pfizer Inc, F. Hoffman-La Roche, Schering-Plough, Synarc, Inc., and Wyeth, as well as non-profit partners the Alzheimer's Association and Alzheimer's Drug Discovery Foundation, with participation from the U.S. Food and Drug Administration. Private sector contributions to ADNI are facilitated by the Foundation for the National Institutes of Health (www.fnih.org <http://www.fnih.org/> <http://www.fnih.org <http://www.fnih.org/> >). The grantee organization is the Northern California Institute for Research and Education, and the study is coordinated by the Alzheimer's Disease Cooperative Study at the University of California, San Diego. ADNI data are disseminated by the Laboratory for Neuro Imaging at the University of California, Los Angeles. This research was also supported by NIH grants P30 AG010129, K01 AG030514, and the Dana Foundation.Other support has come from AG10124 and the Marian S. Ware Alzheimer Program. VMYL is the John H. Ware 3rd Professor for Alzheimer's Disease Research and JQT is the William Maul Measy-Truman G. Schnabel Jr. M.D. Professor of Geriatric Medicine and Gerontology. We thank Michal Figurski for help with the statistical analyses. We are grateful to Christopher M Clark, University of Pennsylvania Medical Center, Anne Fagan, Washington University, Hiroyuki Arai, Tohuku University and Holly Soares, Pfizer Global Research and Development, for providing aliquots of non-ADNI CSF samples to prepare the CSF quality control pools used in the immunoassay system employed in this investigation. We thank Donald Baldwin and the Molecular Diagnosis Genotyping Facility at the University of Pennsylvania Medical Center for provision of the APOε genotyping data.

References

1. Schneider JA, Arvanitakis Z, Bang W, Bennett DA. Mixed brain pathologies account for most dementia cases in community-dwelling older persons. Neurol 2007;69:2197–204.
2. White L, Small BJ, Petrovitch H, Ross GW, Masaki K, Abbott RD, Hardman J, Davis D, Nelson J, Markesbery W. Recent clinical-pathologic research on the causes of dementia in late life: update from the Honolulu-Asia Aging Study. J Geriatr Psychiatry Neurol 2005;18:224–7. [PubMed: 16306244]
3. Gomez-Isla T, Hollister R, West H, Mui S, Growdon JH, Petersen RC, Parisi JE, Hyman BT. Neuronal loss correlates with but exceeds neurofibrillary tangles in Alzheimer's disease. Ann Neurol 1997;41:17–24. [PubMed: 9005861]
4. Savva GM, Wharton SG, Ince PG, Forster G, Matthews FE, Brayne C. Age, neuropathology, and dementia. N Engl J Med 2009;360:2302–9. [PubMed: 19474427]
5. Terry RD, Masliah E, Salmon DP, Butters N, DeTeresa R, Hill R, Hansen LA, Katzman R. Physical basis of cognitive alterations in Alzheimer's disease: synapse loss is the major correlate of cognitive impairment. Ann Neurol 1991;30:572–80. [PubMed: 1789684]
6. Knopman DS, Parisi JE, Salviati A, Floriach-Robert M, Boeve BF, Ivnik RJ, Smith GE, Dickson DW, Johnson KA, Petersen LE, McDonald WC, Braak H, Petersen RC. Neuropathology of cognitively normal elderly. J Neuropathol Exp Neurol 2003;62:1087–95. [PubMed: 14656067]
7. Price JL, Morris JC. Tangles and plaques in nondemented aging and “preclinical” Alzheimer's disease. Ann Neurol 1999;45:358–68. [PubMed: 10072051]
8. Petersen RC. Mild cognitive impairment as a diagnostic entity. J Intern Med 2004;256:183–94. [PubMed: 15324362]

NIH-PA Author Manuscript NIH-PA Author Manuscript NIH-PA Author Manuscript

9. Dubois B, Feldman HH, Jacova C, Dekosky ST, Barberger-Gateau P, Cummings J, Delacourte a, Galasko d, Gauthier S, Jicha G, Meguro K, O'Brien J, Pasquier F, Robert P, Rossor M, Salloway S, Stern Y, Visser PJ, Scheltens P. Research criteria for the diagnosis of Alzheimer's disease: revising the NINCDS-ADRDA criteria. Lancet Neurol 2007;6:734–46. [PubMed: 17616482]
10. Chetelat G, Desgranges B, de la Sayette V, Viader F, Eustache F, Baron JC. Mild cognitive impairment: Can FDG-PET predict who is to rapidly convert to Alzheimer's disease? Neurol 2003;60:1374–7.
11. Dickerson BC, Goncharova I, Sullivan MP, Forchetti C, Wilson RS, Bennett DA, Beckett LA, deToledo-Morrell L. MRI-derived entorhinal and hippocampal atrophy in incipient and very mild Alzheimer's disease. Neurobiol Aging 2001;22:747–54. [PubMed: 11705634]
12. Drzezga A, Lautenschlager N, Siebner H, Riemenschneider M, Willoch F, Minoshima S, Schwaiger M, Kurz A. Cerebral metabolic changes accompanying conversion of mild cognitive impairment into Alzheimer's disease: a PET follow-up study. Eur J Nucl Med Mol Imaging 2003;30:1104–13. [PubMed: 12764551]
13. Jack CR Jr, Petersen RC, Xu YC, O'Brien PC, Smith GE, Ivnik RJ, Boeve BF, Waring SC, Tangalos EG, Kokmen E. Prediction of AD with MRI-based hippocampal volume in mild cognitive impairment. Neurol 1999;52:1397–403.
14. Killiany RJ, Gomez-Isla T, Moss M, Kikinis R, Sandor T, Jolesz F, Tanzi R, Jones K, Hyman BT, Albert MS. Use of structural magnetic resonance imaging to predict who will get Alzheimer's disease. Ann Neurol 2000;47:430–9. [PubMed: 10762153]
15. Petersen RC, Smith GE, Ivnik RJ, Tangalos EG, Schaid DJ, Thibodeau SN, Kokmen E, Waring SC, Kurland LT. Apolipoprotein E status as a predictor of the development of Alzheimer's disease in memory-impaired individuals. JAMA 1995;273:1274–8. [PubMed: 7646655]
16. Shaw LM, Vanderstichele H, Knapik-Czajka M, Clark CM, Aisen PS, Petersen RC, Blennow K, Soares H, Simon A, Lewczuk P, Dean R, Siemers E, Potter W, Lee VMY, Trojanowski JQ. Cerebrospinal fluid biomarker signature in Alzheimer's disease neuroimaging initiative subjects. Ann Neurol 2009;65:403–13. [PubMed: 19296504]
17. Visser PJ, Scheltens P, Verhey FR, Schmand B, Launer LJ, Jolles J, Jonker C. Medial temporal lobe atrophy and memory dysfunction as predictors for dementia in subjects with mild cognitive impairment. J Neurol 1999;246:477–85. [PubMed: 10431775]
18. Mattsson N, Zetterberg H, Hansson O, Andreasen N, Parnetti L, Jonsson M, Herukka SK, van der Flier WM, Blankenstein MA, Ewers M, Rich K, Kaiser E, Verbeek M, Tsolaki M, Mulugeta E, Rosén E, Aarsland D, Visser PJ, Schröder J, Marcusson J, de Leon M, Hampel H, Scheltens P, Pirttilä T, Wallin A, Jönhagen ME, Minthon L, Winblad B, Blennow K. CSF biomarkers incipient Alzheimer disease in patients with mild cognitive inpairment. JAMA 2009;302:385–393. [PubMed: 19622817]
19. Vemuri P, Wiste HJ, Weigand SD, Shaw LM, Trojanowski JQ, Weiner MW, Knopman DS, Petersen RC, Jack CR Jr. MRI and CSF biomarkers in normal, MCI, and AD subjects: predicting future clinical change. Neurol 2009;73:294–301.
20. McKhann G, Drachman D, Folstein M, Katzman R, Price D, Stadlan EM. Clinical diagnosis of Alzheimer's disease: report of the NINCDS-ADRDA Work Group under the auspices of Department of Health and Human Services Task Force on Alzheimer's Disease. Neurol 1984;34:939–44.
21. Vanderstichele, H.; De Meyer, G.; Shapiro, F.; Engelborghs, B.; DeDeyn, PP.; Shaw, LM.; Trojanowski, JQ. Alzheimer's disease biomarkers: From concept to clinical utility. In: Galimberti, D.; Scarpini, E., editors. Biomarkers For Early Diagnosis Of Alzheimer's Disease. Nova Science Publishers Inc; Hauppauge, NY: 2008. p. 81-122.
22. De Meyer G, Shapiro F, Vanderstichele H, Vanmechelen E, Engleborghs B, De Deyn PP, Hanson O, Minthon L, Zetterberg H, Blennow K, Shaw LM, Trojanowski JQ, Alzheimer's Disease Neuroimaging Initiative. A mixture modeling approach to biomarker assessment reveals an Alzheimer's disease signature in more than a third of cognitively normal elderly people. Arch Neurol. 2010 In press.
23. Okonkwo OC, Alosco ML, Griffith HR, Mielke MM, Shaw LM, Trojanowski JQ, Tremont G, Alzheimer's Disease Neuroimaging Initiative. CSF abnormalities and rate of decline in everyday function. Arch Neurol. 2010 In press.
24. Shaw LM, Korecka M, Clark CM, Lee VMY, Trojanowski JQ. Biomarkers of neurodegenertaion for diagnosis and monitoring therapeutics. Nat Rev Drug Discovery 2007;6:295–303.

NIH-PA Author Manuscript

NIH-PA Author Manuscript

NIH-PA Author Manuscript

25. Haschke M, Zhang YL, Kahle C, Klawitter J, Korecka M, Shaw LM, Christians U. HPLC-atmospheric pressure chemical ionization MS/MS for quantification of 15-F2t-isoprostane in human urine and plasma. Clin Chem 2007;53:489–497. [PubMed: 17259231]
26. Petersen RC, Aisen PS, Beckett LA, Donahue MJ, Gamst AC, Harvey DJ, Jack CR Jr, Jagust WJ, Shaw LM, Toga AW, Trojanowski JQ, Weiner MW, Alzheimer's Disease Neuroimaging Initiative. Alzheimer's Disease Neuroimaging Initiative (ADNI): Clinical characterization. Neurol 2010;74:201–209.
27. Chou YY, Leporé N, Avedissian C, Madsen SK, Parikshak N, Hua X, Shaw LM, Trojanowski JQ, Weiner MW, Toga AW, Thompson PM, Alzheimer's Disease Neuroimaging Initiative. Mapping correlations between ventricular expansion, and CSF amyloid and tau biomarkers in 240 subjects with Alzheimer's disease, mild cognitive impairment and elderly controls. Neuroimage 2009;46:394–410. [PubMed: 19236926]
28. Jagust WJ, Landau SM, Shaw LM, Trojanowski JQ, Koeppe RA, Reiman EM, Foster NL, Petersen RC, Weiner MW, Price JC, Mathis CA, Alzheimer's Disease Neuroimaging Initiative. Relationships between biomarkers in aging and dementia. Neurol 2009;73:1193–1199.
29. Leow AD, Yanovsky I, Parikshak N, Hua X, Lee S, Toga AW, Jack CR Jr, Bernstein MA, Britson PJ, Gunter JL, Ward CP, Borowski B, Shaw LM, Trojanowski JQ, Fleisher AS, Harvey D, Kornak J, Schuff N, Alexander GE, Weiner MW, Thompson PM, Alzheimer's Disease Neuroimaging Initiative. Alzheimer's Disease Neuroimaging Initiative: A one-year follow up study using tensor-based morphometry correlating degenerative rates, biomarkers and cognition. NeuroImage 2009;45:645–455. [PubMed: 19280686]
30. Schuff N, Woerner N, Boreta L, Kornfeld T, Shaw LM, Trojanowski JQ, Thompson PM, Jack CR Jr, Weiner MW, Alzheimer's Disease Neuroimaging Initiative. Hippocampal volume loss in early Alzheimer's disease in relation to ApoE genotype and biomarkers. Brain 2009;132:1067–1077. [PubMed: 19251758]
31. Vemuri P, Wiste HJ, Weigand SD, Shaw LM, Trojanowski JQ, Weiner M, Knopman DS, Petersen RC, Jack CR Jr, Alzheimer's Disease Neuroimaging Initiative. MRI and CSF biomarkers in normal, MCI, AD: Diagnostic discrimination and cognitive correlations. Neurol 2009;73:287–293.
32. Vemuri P, Wiste HJ, Weigand SD, Shaw LM, Trojanowski JQ, Weiner M, Knopman DS, Petersen RC, Jack CR Jr, Alzheimer's Disease Neuroimaging Initiative. MRI and CSF biomarkers in normal, MCI, AD: Predicting future clinical change. Neurol 2009;73:294–301.
33. Dean RA, Shaw LM. Use of cerebrospinal fluid biomarkers for diagnosis of incipient Alzheimer disease in patients with mild cognitive impairment. Clin Chem 2010;56:7–9. [PubMed: 19926774]
34. Hampel H, Shen Y, Walsh DM, Aisen P, Shaw LM, Zetterberg H, Trojanowski JQ, Blennow K. Biological markers of β-amyloid related mechanisms in Alzheimer's disease. Exper Neurol. 2010 In press.
35. Hampel H, Blennow K, Shaw LM, Hoessler YC, Zetterberg H, Trojanowski JQ. Total and phosphorylated tau protein as biological markers of Alzheimer's Disease. Exper Gerontol 2010;45:30–40. [PubMed: 19853650]
36. Jack CR Jr, Knopman DS, Jagust WJ, Shaw LM, Aisen PS, Petersen RC, Trojanowski JQ. Modeling dynamic biomarkers of the Alzheimer's pathological cascade. Lancet Neurol 2010;9:119–128. [PubMed: 20083042]
37. Petersen RC, Trojanowski JQ. Time for Alzheimer's disease biomarkers? Potentially yes for clinical trials, but not yet for clinical practice. JAMA 2009;302:436–7. [PubMed: 19622825]
38. Blennow K, Hampel H, Weiner M, Zetterberg H. Cerebrospinal fluid and plasma biomarkers in Alzheimer's disease. Nat Rev Neurol. 2010 In press.

NIH-PA Author Manuscript
NIH-PA Author Manuscript
NIH-PA Author Manuscript

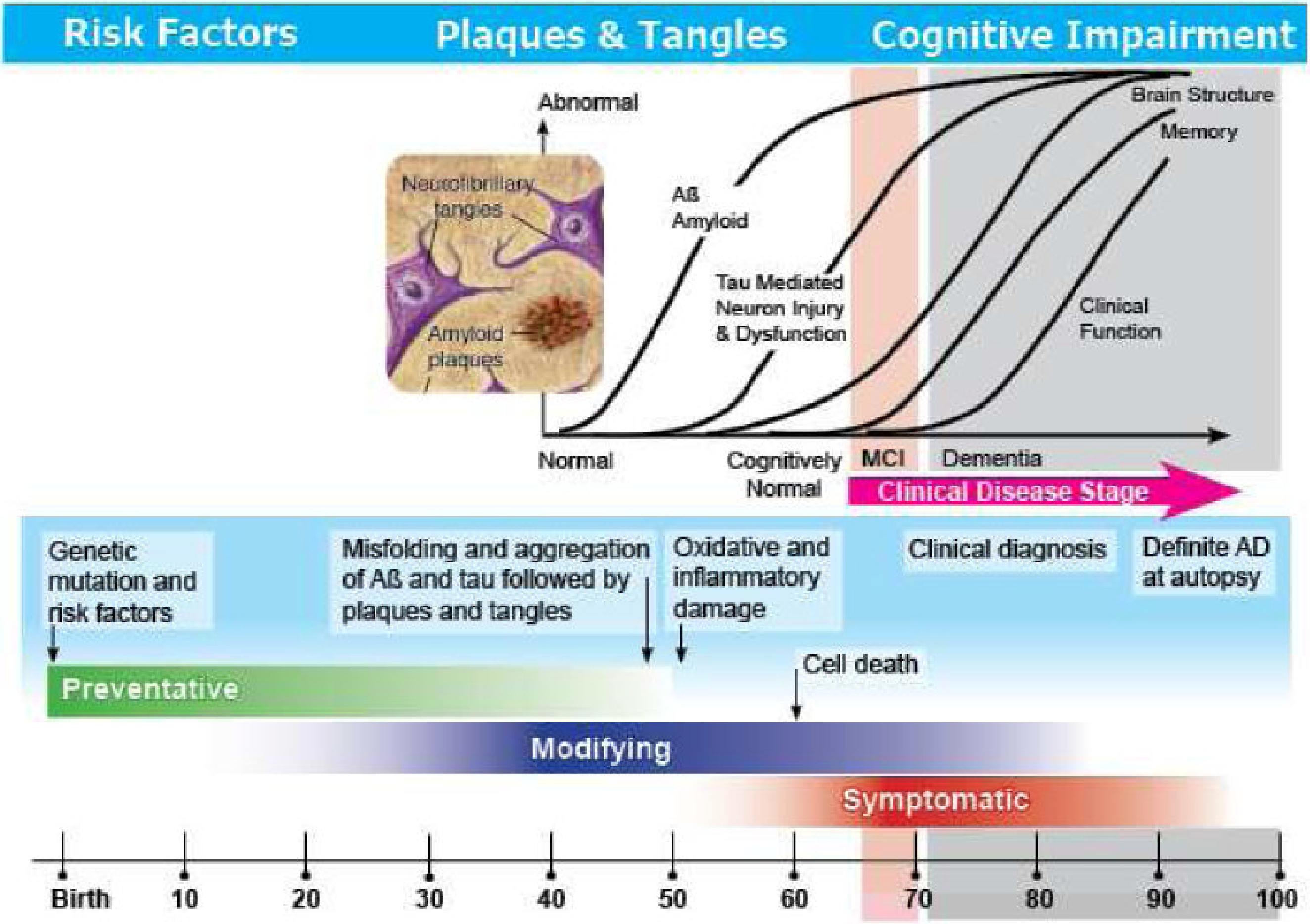

Figure 1.

This figure shows a hypothetical time line for the onset and progression of AD neurodegeneration and cognitive impairments progressing from NC to MCI and on to AD. The only highly predictive biomarkers for AD years before disease onset are genetic mutations that are pathogenic for familial AD (FAD), and these can be detected from birth onwards to identify those individuals in FAD kindreds who will go on to develop AD later in life. However, the emphasis in this review is on promising AD biomarkers studied in ADNI for the diagnosis of AD and predicting conversion from NC and/or MCI status to AD. Age from birth onwards is indicated in the timeline at the bottom of the figure and the green, blue and red bars indicate the time points at which preventive, disease modifying and symptomatic interventions, respectively, are likely to be most effective, and the aqua bar identifies milestones in the pathobiology of AD that culminate in death and autopsy confirmation of AD. However, AD biomarkers are needed to accelerate efforts to test the efficacy of preventive and disease modifying therapies for AD. To do this, it is important to determine the temporal ordering of AD biomarkers, and the proposed ADNI model illustrating the ordering of biomarkers of AD pathology relative to stages in the clinical onset and progression of AD is shown in the insert at the upper right of the figure adjacent to a depiction to the left of the defining pathologies of AD, i.e. plaques and tangles. In the insert on the right, clinical disease is on the horizontal axis and it is divided into three stages; cognitively normal, MCI and dementia. The vertical axis indicates the range from normal to abnormal for each of the biomarkers as well as for measures of memory and functional impairments. Amyloid imaging and CSF Aβ are biomarkers of brain

Aβ amyloidosis. CSF tau and FDG PET are biomarkers of neuron injury and degeneration while structural MRI is a biomarker of abnormal brain morphology.

NIH-PA Author Manuscript

NIH-PA Author Manuscript

NIH-PA Author Manuscript

Statistics of highlighted entities of class BIM (page 1)

Entity	Frequency
Manuscript	37
manuscript	15
collection	9
website	5
groups	4
performance	3
brief	3
standard	2
code	2
report	2
group	2
Collection	1
Resource	1
person	1
Group	1
version	1
manual	1
journal	1
organization	1
event	1
request	1
Access	1
license	1
note	1

Statistics of highlighted entities of class NIFT (page 1)

Entity	Frequency
CSF	103
tau	19
T-tau	15
MRI	12
FDG-PET	6
cerebrospinal fluid	4
ADAS-Cog	3
CDR	3
scores	3
hippocampal volume	3
ventricular expansion	3
MMSE	3
Alzheimer disease	2
P-tau	2
Cerebrospinal fluid	2
Tau	2
STAND	2
Clinical Dementia Rating	1
structural magnetic resonance imaging	1
temporal atrophy	1
PIB	1
cerebral atrophy	1
Hippocampal Volume	1
Hippocampal volume	1
T- tau	1
Ventricular Expansion	1
tensor- based morphometry	1
hippocampal atrophy	1
ADAS- Cog	1
algorithms	1
Medial temporal lobe atrophy	1
temporal lobe atrophy	1
hippocampal	1
Temporal lobe atrophy	1

Statistics of highlighted entities of class QIBO (page 1)

Entity	Frequency
disease	29
Disease	19
model	11
diagnosis	10
brain	8
chemical	5
volume	5
hippocampal	5
Hippocampal	2
enzyme	2
Diagnosis	2
blood	2
Model	1
Cerebral	1
drug development	1
preclinical	1
area	1
length	1
disease staging	1
Screening	1
staging	1
cerebral	1
Volume	1
cell	1
Brain	1

Key for highlighting of entities

Class	Color	Overall Frequency
BIM		97
NIFT		204
QIBO		113
