# Supplementary material for: Neuroimaging Feature Terminology: A Controlled Terminology for the Annotation of Brain Imaging Features
Source: J Alzheimers Dis. 2017 Aug 14;59(4):1153–69. doi: 10.3233/JAD-161148 (PMC5611802; doi:10.3233/JAD-161148)
Supplement: Supplementary file 3 [file jad-59-jad161148-s003.zip › Supplementary_File3/002_S_4171_MCI/Output_002_S_4171_FDG/spmT_report.pdf]

SPMgrid Report

Subject info

Patient ID: 002_S_4171_FDG **Sex:** M **Age:** 70

Pre-processing and registration step[1]

Linear {affine} component

X1 = 0.994*X −0.006*Y −0.011*Z −0.247

Y1 = 0.005*X +0.994*Y −0.020*Z +1.225

Z1 = 0.003*X +0.000*Y +1.022*Z −0.667

16 nonlinear iterations

7 x 9 x 8 basis functions

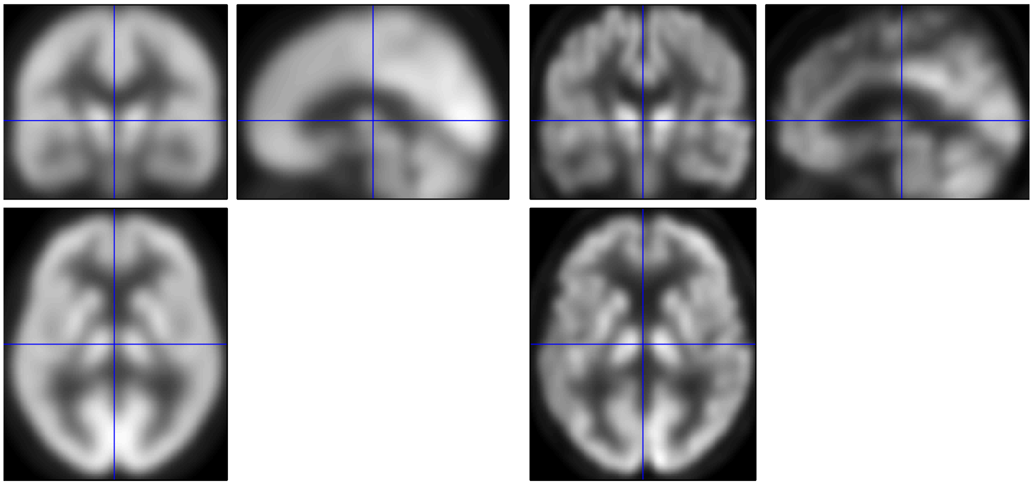

[1] Registration of the subject's brain to the ICBM152 space. The Template used is an average of 100 subjects (50 Healthy Elderly Controls and 50 patients matched per sex and age).

Hypometabolism

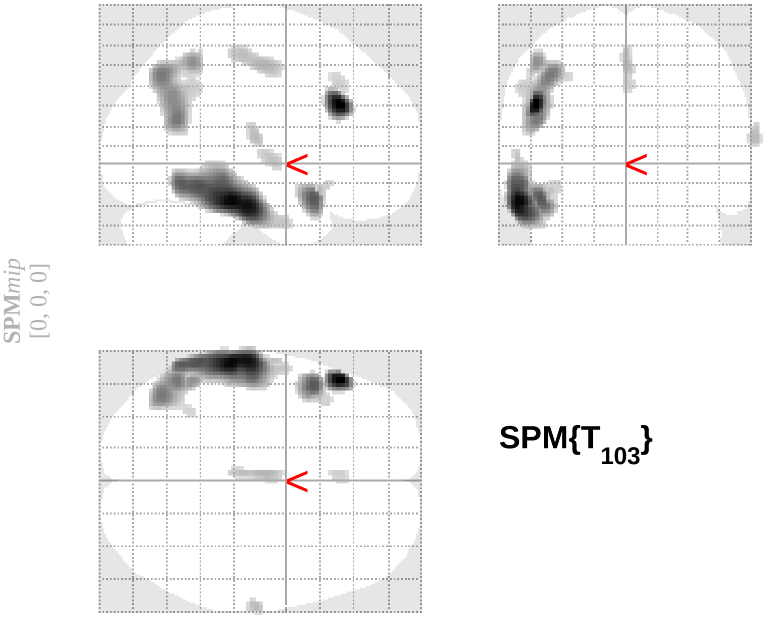

SPM{T_{103}}

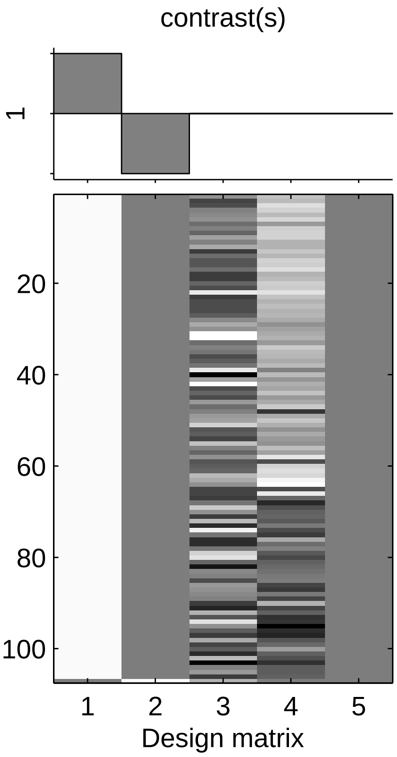

SPMresults: ./CREAM631855649/COMMON_FILES

Height threshold T = 3.171250 {p<0.001 (unc.)}

Extent threshold k = 10 voxels

Statistics: *p−values adjusted for search volume*

set−level		cluster−level				peak−level					mm mm mm
p	c	$p_{FWE-corr}$	$q_{FDR-corr}$	k_E	p_{uncorr}	$p_{FWE-corr}$	$q_{FDR-corr}$	T	$(Z_{\equiv})$	p_{uncorr}	
**0.040**	**8**	**0.356**	**0.148**	**141**	**0.074**	**0.002**	**0.004**	**5.73**	**5.33**	**0.000**	**−50 26 28**
		**0.000**	**0.000**	**1465**	**0.000**	**0.003**	**0.004**	**5.59**	**5.21**	**0.000**	**−58 −32 −22**
						0.004	0.004	5.51	5.14	0.000	−60 −22 −24
						0.055	0.050	4.76	4.52	0.000	−60 −48 −14
		**0.243**	**0.125**	**180**	**0.047**	**0.073**	**0.052**	**4.68**	**4.44**	**0.000**	**−48 12 −20**
						0.934	0.643	3.49	3.39	0.000	−42 16 −16
		**0.001**	**0.001**	**835**	**0.000**	**0.214**	**0.108**	**4.33**	**4.14**	**0.000**	**−50 −60 22**
						0.217	0.108	4.33	4.14	0.000	−42 −68 44
						0.384	0.191	4.11	3.94	0.000	−48 −62 32
		**0.508**	**0.191**	**104**	**0.120**	**0.830**	**0.570**	**3.66**	**3.54**	**0.000**	**−2 −12 48**
						0.896	0.617	3.56	3.45	0.000	−2 −26 54
		**0.890**	**0.421**	**33**	**0.372**	**0.882**	**0.617**	**3.59**	**3.47**	**0.000**	**68 −18 12**
		**0.918**	**0.421**	**27**	**0.421**	**0.930**	**0.643**	**3.50**	**3.40**	**0.000**	**−60 −10 0**
		**0.885**	**0.421**	**34**	**0.365**	**0.975**	**0.685**	**3.37**	**3.28**	**0.001**	**−2 26 40**

table shows 3 local maxima more than 8.0mm apart

Height threshold: T = 3.17, p = 0.001 (0.997)
Extent threshold: k = 10 voxels, p = 0.640 (0.978)
Expected voxels per cluster, <k> = 44.679
Expected number of clusters, <c> = 3.80
FWEp: 4.792, FDRp: 5.505, FWEc: 835, FDRc: 835

Degrees of freedom = [1.0, 103.0]
FWHM = 13.9 15.2 15.4 mm mm mm; 6.9 7.6 7.7 {voxels}
Volume: 1631952 = 203994 voxels = 452.8 resels
Voxel size: 2.0 2.0 2.0 mm mm mm; (resel = 404.48 voxels)

Spatial Data Visualization[2]

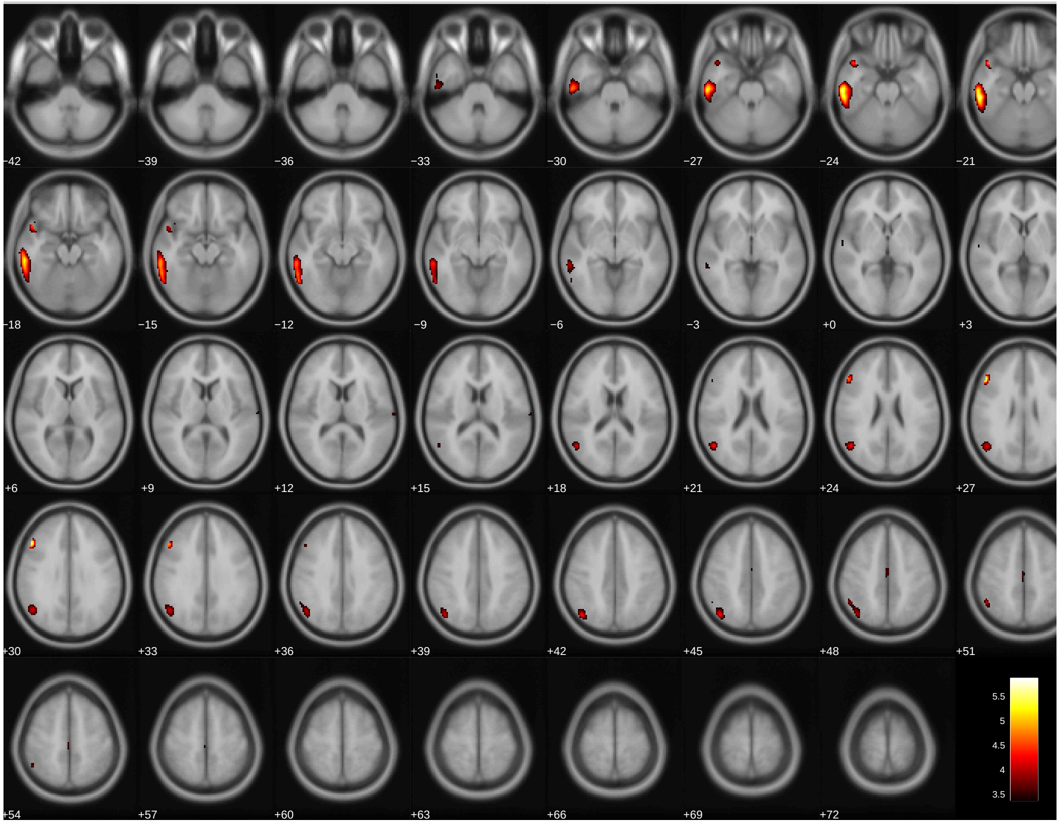

[2]In SPM the spatially normalised images are in neurological convention (with the right side of the brain being at the right side of the image).
