# Supplementary material for: Neuroimaging Feature Terminology: A Controlled Terminology for the Annotation of Brain Imaging Features
Source: J Alzheimers Dis. 2017 Aug 14;59(4):1153–69. doi: 10.3233/JAD-161148 (PMC5611802; doi:10.3233/JAD-161148)
Supplement: Supplementary file 3 [file jad-59-jad161148-s003.zip › Supplementary_File3/016_S_4952_Control/Output_016_S_4952_FDG/spmT_report.pdf]

# SPMgrid Report

## Subject info

**Patient ID:** 016_S_4952_FDG **Sex:** F **Age:** 70

## Pre-processing and registration step[1]

**Linear {affine} component**

X1 = 0.985*X −0.008*Y +0.001*Z −0.205

Y1 = 0.015*X +0.997*Y −0.012*Z +0.552

Z1 = −0.007*X +0.016*Y +1.083*Z +0.804

16 nonlinear iterations

7 x 9 x 8 basis functions

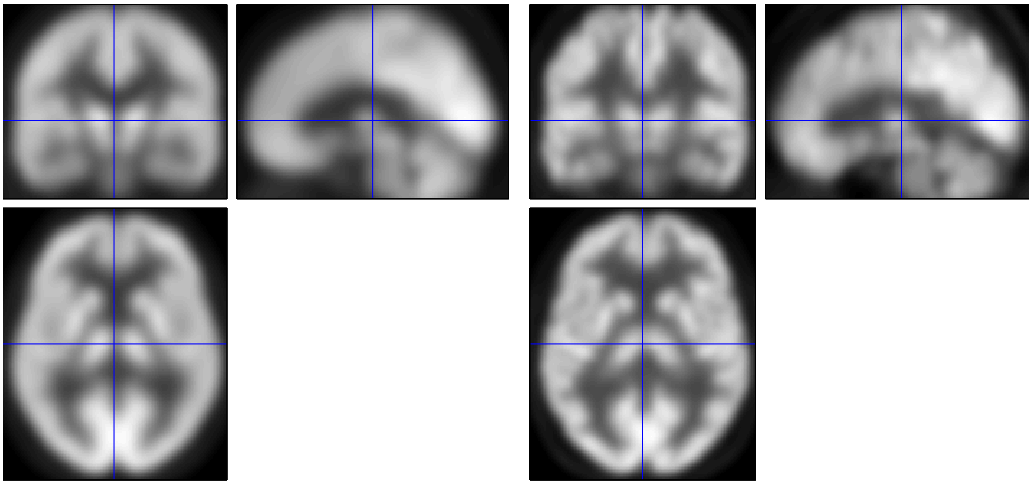

[1] Registration of the subject's brain to the ICBM152 space. The Template used is an average of 100 subjects (50 Healthy Elderly Controls and 50 patients matched per sex and age).

# Hypometabolism

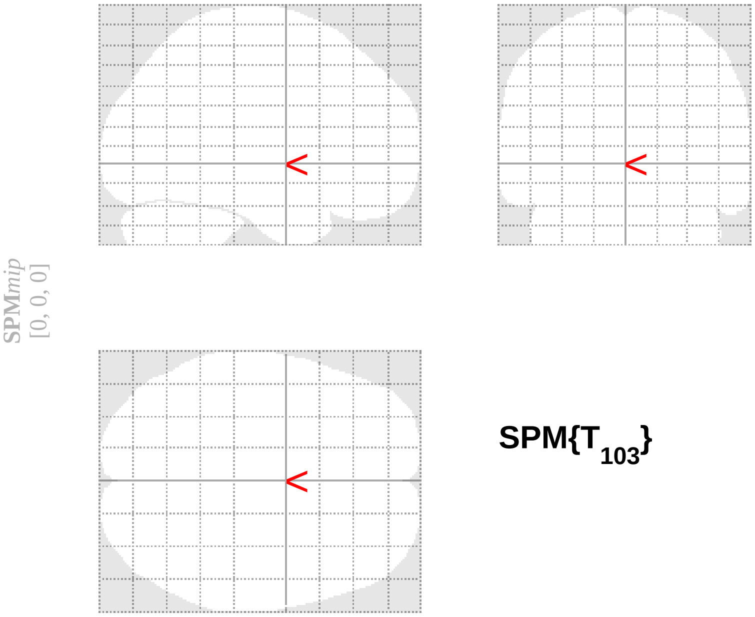


SPM{T$_{103}$}

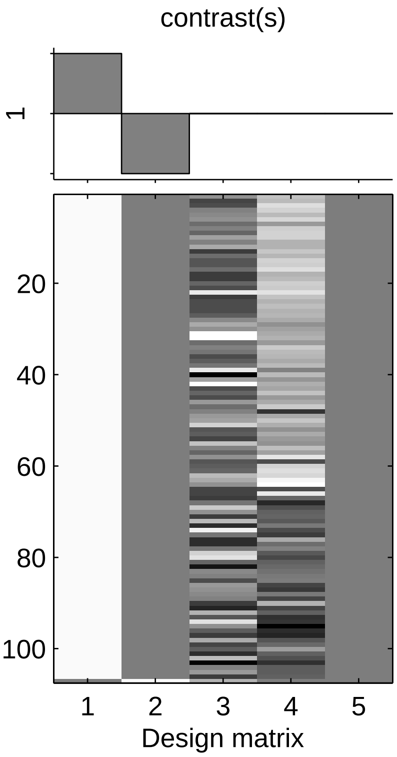


**SPMresults:** ./CREAM690209757/COMMON_FILES

Height threshold T = 3.171250 {p<0.001 (unc.)}

Extent threshold k = 0 voxels

**Statistics:** ***p−values adjusted for search volume***

| set−level | | cluster−level | | | | peak−level | | | | | mm mm mm |
|---|---|---|---|---|---|---|---|---|---|---|---|
| $p$ | $c$ | $p_{FWE-corr}$ | $q_{FDR-corr}$ | $k_E$ | $p_{uncorr}$ | $p_{FWE-corr}$ | $q_{FDR-corr}$ | $T$ | $(Z_{\equiv})$ | $p_{uncorr}$ | |

***no suprathreshold clusters***

*table shows 3 local maxima more than 8.0mm apart*

Height threshold: T = 3.17, p = 0.001 (0.997)
Extent threshold: k = 0 voxels, p = 1.000 (0.997)
Expected voxels per cluster, <k> = 44.683
Expected number of clusters, <c> = 5.93
FWEp: 4.792, FDRp: Inf, FWEc: Inf, FDRc: Inf

Degrees of freedom = [1.0, 103.0]
FWHM = 13.9 15.2 15.4 mm mm mm; 6.9 7.6 7.7 {voxels}
Volume: 1633016 = 204127 voxels = 453.3 resels
Voxel size: 2.0 2.0 2.0 mm mm mm; (resel = 404.53 voxels)

## Spatial Data Visualization[2]

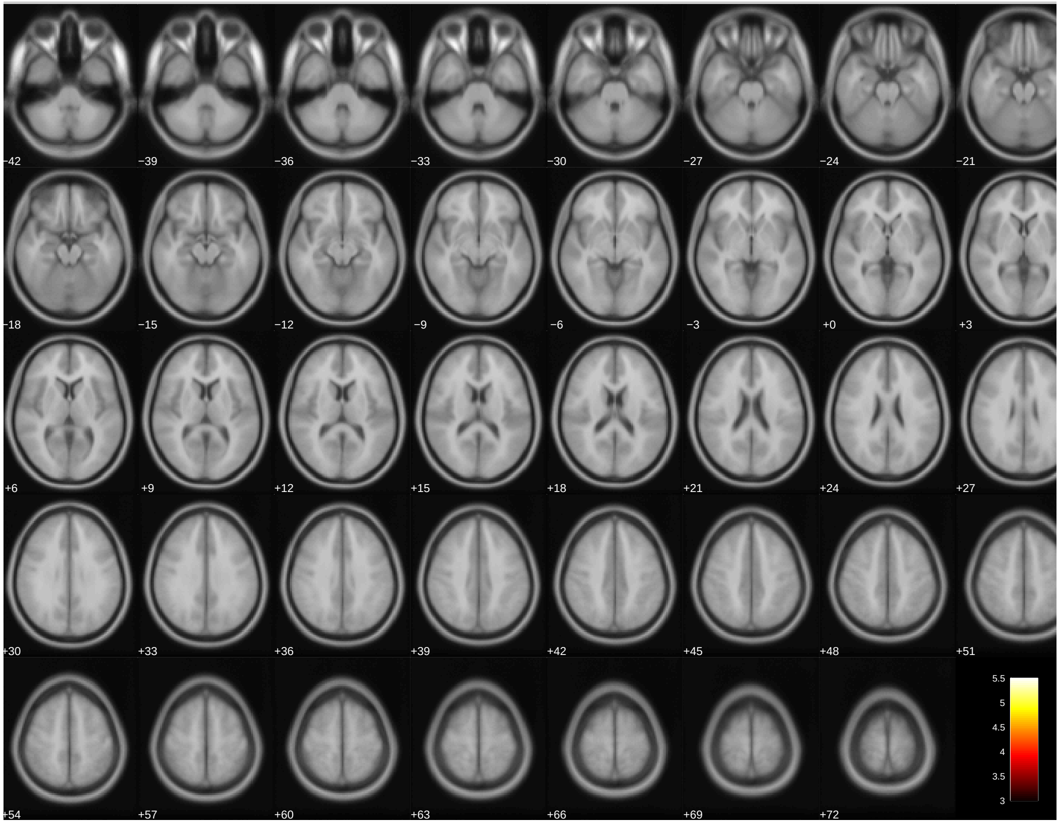


[2] In SPM the spatially normalised images are in neurological convention (with the right side of the brain being at the right side of the image).
